# Controlling Photophysical Properties in Bis-Cyclometalated Ir(III)-Terpyridine Complexes through Photoinduced Intraligand Charge Transfer

**DOI:** 10.1021/acs.inorgchem.6c01548

**Published:** 2026-06-15

**Authors:** Joanna Palion-Gazda, Aleksandra Kwiecień, Mateusz Penkala, Anna Kryczka, Barbara Machura, Patrycja Rawicka, Mariola Siwy, Dorota Kowalska, Sebastian Maćkowski, Ewa Schab-Balcerzak, Karol Erfurt

**Affiliations:** † Institute of Chemistry, 431562 University of Silesia, 9 Szkolna Str., Katowice 40-006, Poland; ‡ Institute of Physics, Faculty of Science and Technology, University of Silesia, 75 Pułku Piechoty 1a, Chorzów 41-500, Poland; § Centre of Polymer and Carbon Materials, Polish Academy of Sciences, Zabrze 41-819, Poland; ∥ Institute of Physics, Faculty of Physics, Astronomy and Informatics, Nicolaus Copernicus University in Toruń, ul. Grudziądzka 5, Toruń 87-100, Poland; ⊥ Department of Chemical Organic Technology and Petrochemistry, Silesian University of Technology, Krzywoustego 4, Gliwice 44-100, Poland

## Abstract

This work is the study of the role of a remote amine substituent on the terpyridine ligand in controlling the photophysical and electroluminescent behavior of complexes [Ir­(Ph-btz)_2_(R-C_6_H_4_-terpy-κ^2^N)]­PF_6_. Introduction of the amine group into the Ph-terpy framework induced perturbations in the absorption and luminescent characteristics of [Ir­(Ph-btz)_2_(R-C_6_H_4_-terpy-κ^2^N)]­PF_6_ relative to the parent chromophores [Ir­(Ph-btz)_2_(terpy-κ^2^N)]­PF_6_ and [Ir­(Ph-btz)_2_(C_6_H_5_-terpy-κ^2^N)]­PF_6_. Consistent with the presence of vibronic structure, negligible emission solvatochromism, weak rigidochromic behavior, and substantial overlap with the phosphorescence of 2-phenylbenzothiazole, the emissive triplet excited state of the model complexes was assigned to ^3^IL_Ph‑btz_ with a minor ^3^MLCT contribution. The amine-substituted Ir­(III) complexes were found to exhibit altered emission characteristics, manifested by a structureless emission band impacted by the amine group and solvent polarity. It was rationalized by a switch from the ^3^IL_Ph‑btz_/MLCT excited state in model chromophores to a configurationally mixed ^3^ILCT/IL/MLCT_R‑terpy_ in [Ir­(Ph-btz)_2_(R-C_6_H_4_-terpy-κ^2^N)]­PF_6_. With respect to the preliminary electroluminescence investigations, it was found that the introduction of the triphenylamine moiety enhances the stability of O/PEDOT/PSS/complex/Al devices. A deep insight into ground- and excited-state characters and photophysical properties of [Ir­(Ph-btz)_2_(R-C_6_H_4_-terpy-κ^2^N)]­PF_6_ was obtained by applying a wide range of experimental techniques, including cyclic voltammetry, UV–vis spectroscopy, photoluminescence spectroscopy, and transient absorption, complemented by DFT and TD-DFT calculations.

## Graphical abstract

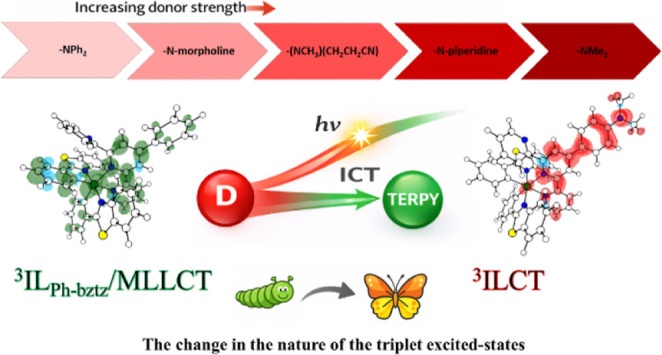

## Introduction

2,2′:6′,2″-Terpyridine derivatives (R-terpy) constitute a unique class of nitrogen-donor ligands that find widespread applications in coordination chemistry.^1–6^ Their transition metal complexes occupy a prominent position among systems suitable for optoelectronics,^7–12^ catalysis and photocatalysis,^13–20^ bioimaging, chemotherapy, and photodynamic therapy.^1,2,6,21–26^ Owing to a broad range of available structural modifications of the terpy backbone, terpyridine-based transition metal complexes also play a key role in elucidating light-induced electron- and energy-transfer processes, as well as in establishing and understanding structure–properties relationships. These aspects are particularly important for developing advanced materials with an optimal set of functional parameters.^5,27–38^

Over the past decade, the photophysical properties of carbonyl Re­(I) complexes have been extensively investigated as a function of terpy structural modifications.^38–50^ In particular, two strategies for achieving terpyridine-based Re­(I) chromophores with prolonged excited-state lifetimes have been demonstrated. First, long-lived excited states in [ReCl­(CO)_3_(R-terpy-κ^2^N)] systems were obtained by incorporating π-conjugated organic substituents into the terpyridine core, thereby enabling access to low-lying triplet intraligand (^3^IL) excited states of the aryl chromophore in the resulting Re­(I) complexes.^15,51^ Second, the introduction of strong electron-donating groups was shown to induce a switch in the nature of the lowest triplet excited state from metal-to-ligand charge transfer (^3^MLCT) to intraligand charge transfer (^3^ILCT).^44,49,50^ The extended excited-state lifetimes made [ReCl­(CO)_3_(Me_2_N-C_6_H_4_-terpy-κ^2^N)], [ReCl­(CO)_3_(4′-(9-anthryl)-terpy-κ^2^N)], and [ReCl­(CO)_3_(4′-(2-anthryl)-terpy-κ^2^N)] suitable for energy transfer from the triplet excited state to molecular oxygen (^3^O_2_) and the formation of singlet oxygen (^1^O_2_). Importantly, the Re­(I) complex bearing the NMe_2_ group was successfully employed as the photosensitizer in hydrogen evolution experiments,^44^ and its excellent sonocytotoxicity toward normoxic and hypoxic cancer cells was evidenced in in vitro and in vivo experiments.^52^

In our latest works,^53,54^ we have reported the effect of the electron-donating morpholinyl (morph) group on the optical properties of [IrCl_3_(R-C_6_H_4_-terpy-κ^3^N)], [Ir­(R-C_6_H_4_-terpy-κ^3^N)_2_]­(PF_6_)_3_, [IrCl­(Ph-py)­(R-C_6_H_4_-terpy-κ^3^N)]­PF_6_, and [Ir­(Ph-py)_2_(R-C_6_H_4_-terpy-κ^2^N)]­PF_6_ systems, demonstrating, among other things, a positive impact of the terpy decoration with the electron-rich group on photoluminescence lifetimes of bis-cyclometalated complexes [Ir­(Ph-py)_2_(R-C_6_H_4_-terpy-κ^2^N)]­PF_6_ (HPh-py = 2-phenypyridine). Notably, the excited-state lifetime of [Ir­(Ph-py)_2_(morph-C_6_H_4_-terpy-κ^2^N)]­PF_6_ was shown to exceed that of the model chromophore [Ir­(Ph-py)_2_(Ph-terpy-κ^2^N)]­PF_6_ by an order of magnitude.

In order to gain insight into the role of the remote substituent on the ground- and excited-state properties of bis-cyclometalated Ir­(III) systems, we designed a series of novel complexes [Ir­(Ph-btz)_2_(R-terpy-κ^2^N)]­PF_6_ and carried out systematic investigations of their electrochemical and photophysical properties (Scheme 1).

**Scheme 1:**
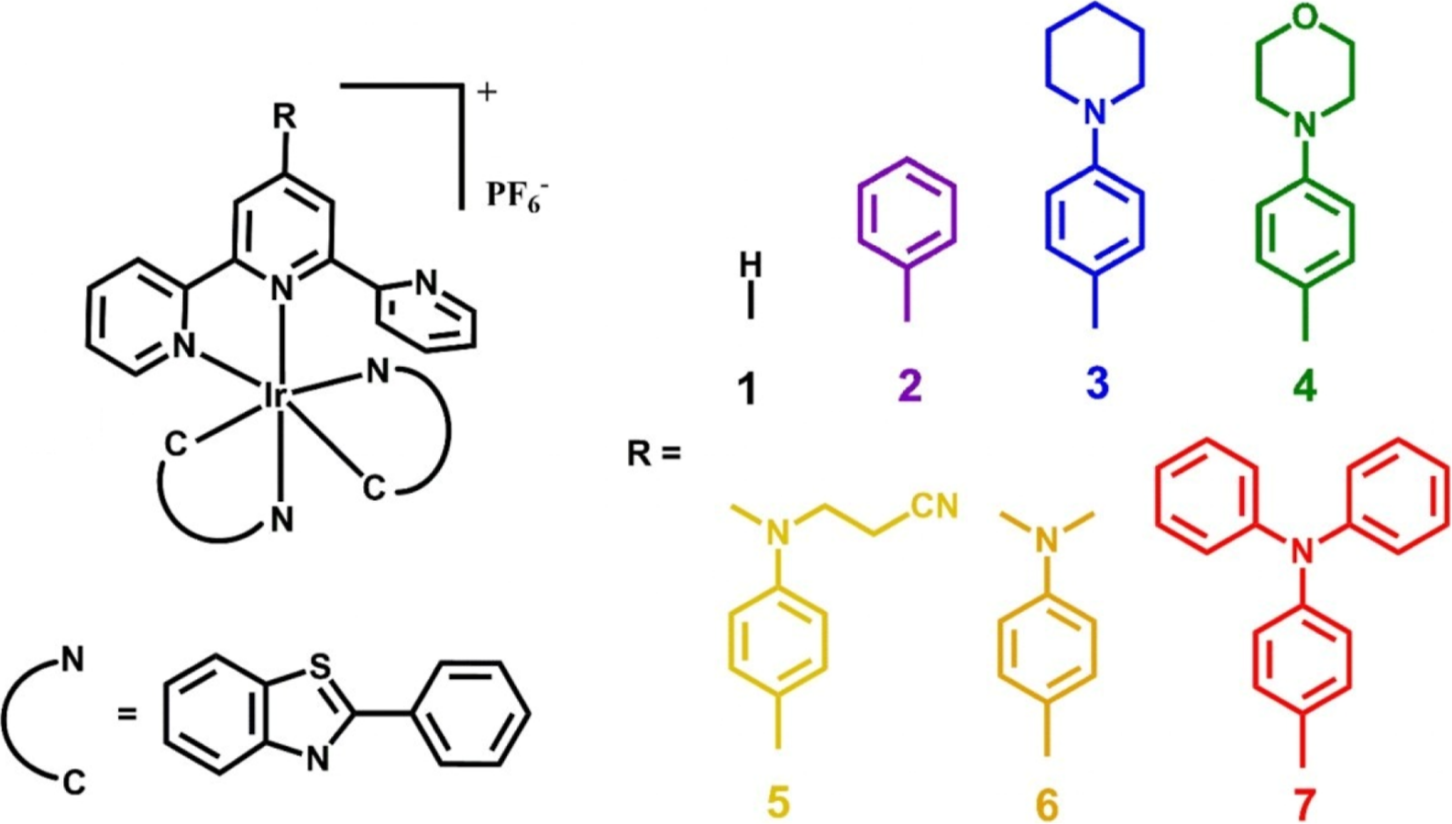
Iridium­(III) Complexes Studied in This Work

To the best of our knowledge, this is the first such comprehensive study focused on the role of the remote amine group of the terpy ancillary ligand in determining the photophysics of bis-cyclometalated Ir­(III) systems. The choice of 2-phenylbenzothiazole (HPh-btz) as a cyclometalating ligand was motivated by previous results, where Ir­(III) complexes containing this ligand exhibited significantly longer excited-state lifetimes compared to those bearing 2-phenylpyridine.^50,55,56^ Moreover, Ir­(III)-based materials obtained by the combination of Ph-btz as cyclometalating and terpy-based as ancillary ligands are extremely underexplored. To date, only the photophysical properties of [Ir­(Ph-btz)_2_(terpy-κ^2^N)]­PF_6_ have been very recently reported.^56^ In this work, substantial insight into ground- and excited-state characters and photophysical properties of [Ir­(Ph-btz)_2_(R-terpy-κ^2^N)]­PF_6_ has been achieved through a wide range of experimental techniques, including cyclic voltammetry (CV), UV–vis spectroscopy, static and time-resolved photoluminescence spectroscopy, and ultrafast transient absorption. The experimental results were corroborated with DFT and TD-DFT calculations. Finally, the designed complexes have been preliminarily tested as luminophores in light-emitting diode configurations.

## Results and Discussion

### Synthesis and Molecular Structures

Bis-cyclometalated heteroleptic iridium­(III) complexes [Ir­(Ph-btz)_2_(R-terpy-κ^2^N)]­PF_6_ (**1**–**7**) were obtained by a standard synthetic methodology,^55,57,58^ based on a bridge-splitting reaction of the dinuclear precursor [Ir­(μ-Cl)_2_(Ph-btz)_4_] with 2 equiv of the appropriate R-terpy derivative in a mixture of CHCl_3_–MeOH, followed by the anion exchange of Cl^–^ with PF_6_^–^ (Scheme 2).

**Scheme 2:**
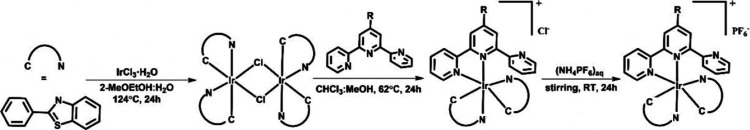
Synthetic Methodology of Complexes **1**–**7**

The formation of the mononuclear systems [Ir­(Ph-btz)_2_(R-terpy-κ^2^N)]­PF_6_ was confirmed by elemental analysis, high-resolution mass spectrometry (Figure S1), ^1^H and ^13^C NMR spectroscopy (Figure S2), and the FTIR technique (Figure S3).

HR-MS spectra of **1**–**7** recorded in a positive ionization mode display a signal corresponding to the appropriate [Ir­(Ph-btz)_2_(R-terpy-κ^2^N)]^+^ cation, along with the characteristic isotopic pattern of iridium. As shown in Figure S2, the proton resonances of the distal pyridines of terpy in [Ir­(Ph-btz)_2_(R-terpy-κ^2^N)]^+^ cations are magnetically nonequivalent, in agreement with a bidentate coordination mode of this ligand.

The presence of the hexafluorophosphate counteranion (PF_6_^–^) is unambiguously confirmed by a distinct PF_6_^–^ peak in the high-resolution mass spectra acquired in negative ionization mode (Figure S1), as well as by intense absorption bands at approximately 840 cm^–1^ and 555 cm^–1^ in the FTIR spectra of compounds **1–7** (Figure S3), in agreement with literature data.^59^

The molecular structures of **2** and **6** were confirmed by single-crystal X-ray analysis (Figure 1).

**Figure 1:**
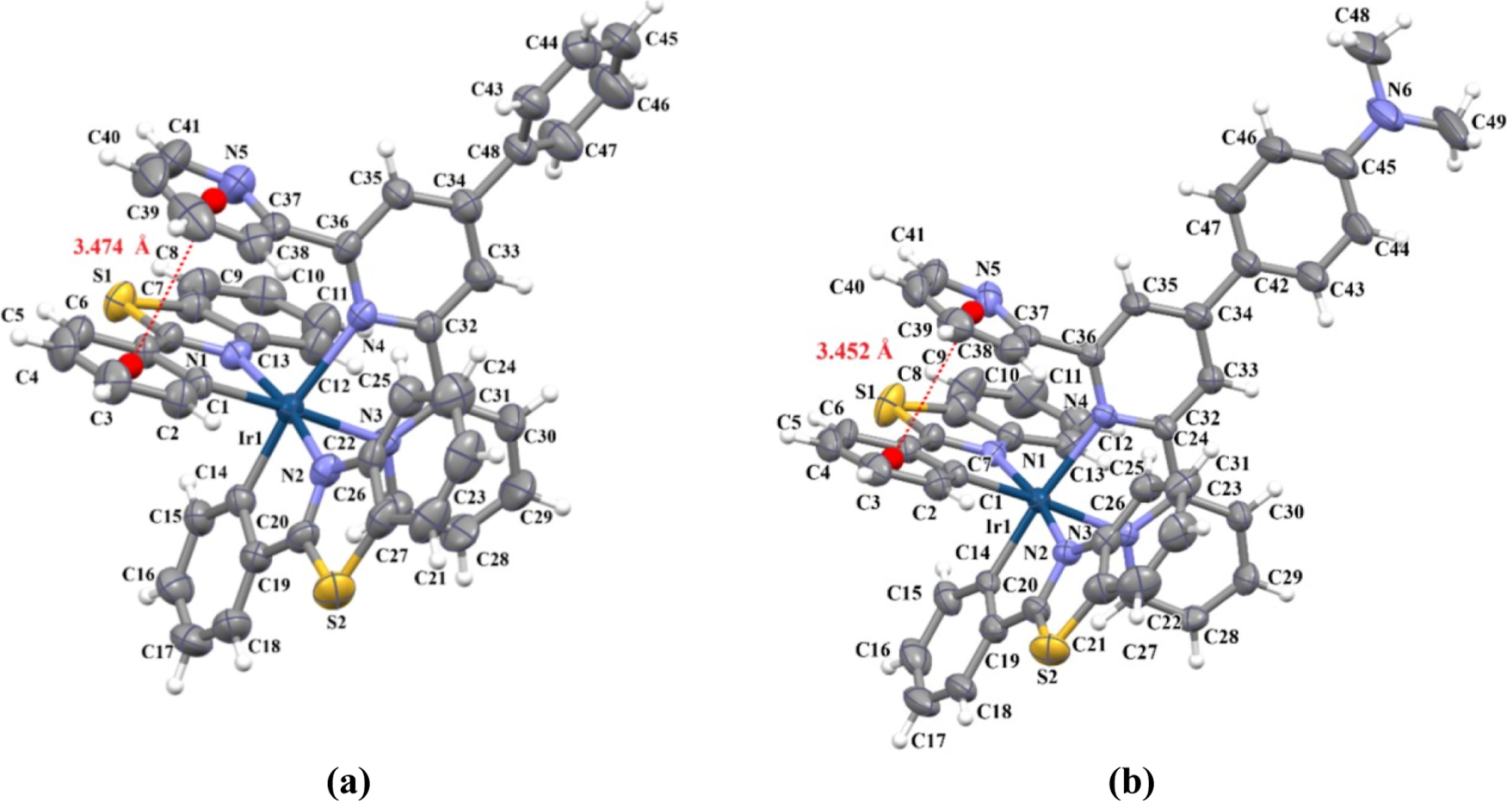
Molecular structures of **2** (a) and **6** (b) with atom numbering and thermal ellipsoids set at 50% probability for non-hydrogen atoms; (PF_6_^–^) counterions, present in **2** and **6**, and the methanol molecule, present in **6**, are omitted for clarity.

In bis-cyclometalated heteroleptic iridium­(III) complexes,^60^ the metal center of **2** and **6** typically shows a distorted octahedral environment, and two cyclometalating 2-phenylbenzothiazole ligands are arranged in a *cis*-C,C and *trans*-N,N configuration relative to each other. The Ph-terpy (**2**) and Me_2_N-C_6_H_4_-terpy (**6**) ligands coordinate to the Ir­(III) ion in a bidentate mode (κ^2^N), and their nitrogen atoms N3 and N4 are trans-arranged to the carbon atoms of Ph-btz ligands. In agreement with the trans influence of carbon donors of cyclometalating ligands, the Ir–N_terpy_ bond lengths [2.145(4) and 2.218(4) Å in **2**, 2.129(4) and 2.206(4) Å in **6**] are noticeably elongated relative to Ir–N_Ph‑btz_ [2.051(4) and 2.064(4) Å in **2**, 2.058(4) and 2.049(4) Å in **6**]. The Ir–C_Ph‑btz_ bond lengths fall in a narrow range of 2.006(5)–2.037(5) Å (Table S2). The noncoordinated pyridine ring of **2** and **6** is engaged in face-to-face π–π stacking interactions with the phenyl ring of the cyclometalating ligand (Figure 1 and Table S4). The considerable angular distortion of **2** and **6** from the octahedral geometry, reflected in the N–Ir–N and N–Ir–C bite angles (Table S2), is assigned to the formation of five-membered metallocycles upon chelating coordination of Ph-btz and terpy-based ligands. Additional structural data, including the analysis of short intra- and intermolecular contacts detected in **2** and **6**, are provided in ESI (Tables S1–S6 and Figure S4).

### Electrochemical Characterization

The role of electron-rich remote amine groups in controlling the redox behavior of [Ir­(Ph-btz)_2_(R-terpy-κ^2^N)]­PF_6_ was investigated using cyclic voltammetry (CV) and differential pulse voltammetry (DPV) on a glassy carbon working electrode in CH_3_CN, with the ferrocene/ferrocenium redox couple as the reference. The electrochemical results, summarized in Table 1, were utilized to determine ionization potentials (IP) and electron affinities (EA) of **1**–**7**, associated with the highest occupied molecular orbital (HOMO) and LUMO energy levels, respectively.^61^

**Table 1:** Electrochemical Data of **1–7**

complex	*E*_pa_^ox1^	*E*_pc_^ox1^	Δ*E* ^ox1^	*E*_1/2_^ox1^	*E*_pa_^ox2^	*E*_pc_^ox2^	Δ*E* ^ox2^	*E*_1/2_^ox2^	*E*_pa_^ox3^	*E*_pc_^ox3^	ΔE^ox3^	*E*_1/2_^ox3^	*E*_pc_^red1^	*E*_pa_^red1^	*E*_pc_^red2^	*E*_pc_^red2^	*E*_onset_^ox1^	*E*_onset_^red1^	IP	EA
**1**	1.00				1.18	1.07	0.11	1.12					–1.73	–1.80			0.92	–1.69	6.02	3.41
**2**	1.01				1.17	1.08	0.09	1.12					–1.64	–1.70			0.92	–1.61	6.02	3.49
**3**	0.59	0.53	0.06	0.56	1.03				1.21	1.10	0.14	1.11	–1.70	–1.77	–2.21	–2.28	0.48	–1.67	5.58	3.43
**4**	0.71	0.67	0.04	0.69	1.01				1.15	1.04	0.11	1.10	–1.67	–1.73	–2.14	–2.24	0.58	–1.62	5.68	3.48
**5**	0.76	0.72	0.04	0.74	1.00				1.17	1.08	0.09	1.12	–1.69	–1.76	–2.18	–2.27	0.62	–1.66	5.72	3.44
**6**	0.63	0.58	0.05	0.58	1.02				1.18	1.07	0.11	1.12	–1.71	–1.78	–2.18	–2.28	0.50	–1.68	5.60	3.42
**7**	0.72	0.52	0.20	0.62	1.02				1.19	1.10	0.09	1.14	–1.66	–1.74	–2.15	–2.29	0.59	–1.62	5.69	3.48

As demonstrated in Figures 2a and S5, all investigated Ir­(III) complexes show multistage redox characteristics within the applied potential range. A striking difference between the non- and amine-substituted systems concerns the oxidation processes. Complexes **1** and **2** display oxidation behavior typical of bis-cyclometalated Ir­(III) complexes, possessing a characteristic irreversible oxidation wave at 0.94 V, attributable to the Ir^III^/Ir^IV^ redox couple with a contribution from the phenyl group of the Ph-bztz ligand.^62–66^ A quasi-reversible first oxidation peak of **3**–**7** appears at much less positive potentials, falling in the range of 0.56–0.69 V. The piperidine and dimethylamine groups make compounds **3** and **6** easier to oxidize than **4**, **5**, and **7**. With reference to electrochemical properties of the free ligands,^67^ this behavior can be easily assigned to oxidation of the appended amine group. The values of *E*_onset_^ox1^ of investigated complexes show linear correlation with the substituent descriptors compatible with the Hammett σ constants, calculated using a free web tool (Figure 2b).^68^ The second irreversible oxidation peak of **3**–**7** is located at potentials related to the first oxidation state of model chromophores and thus represents an iridium-based oxidation process. It is independent of the appended amine groups.

**Figure 2:**
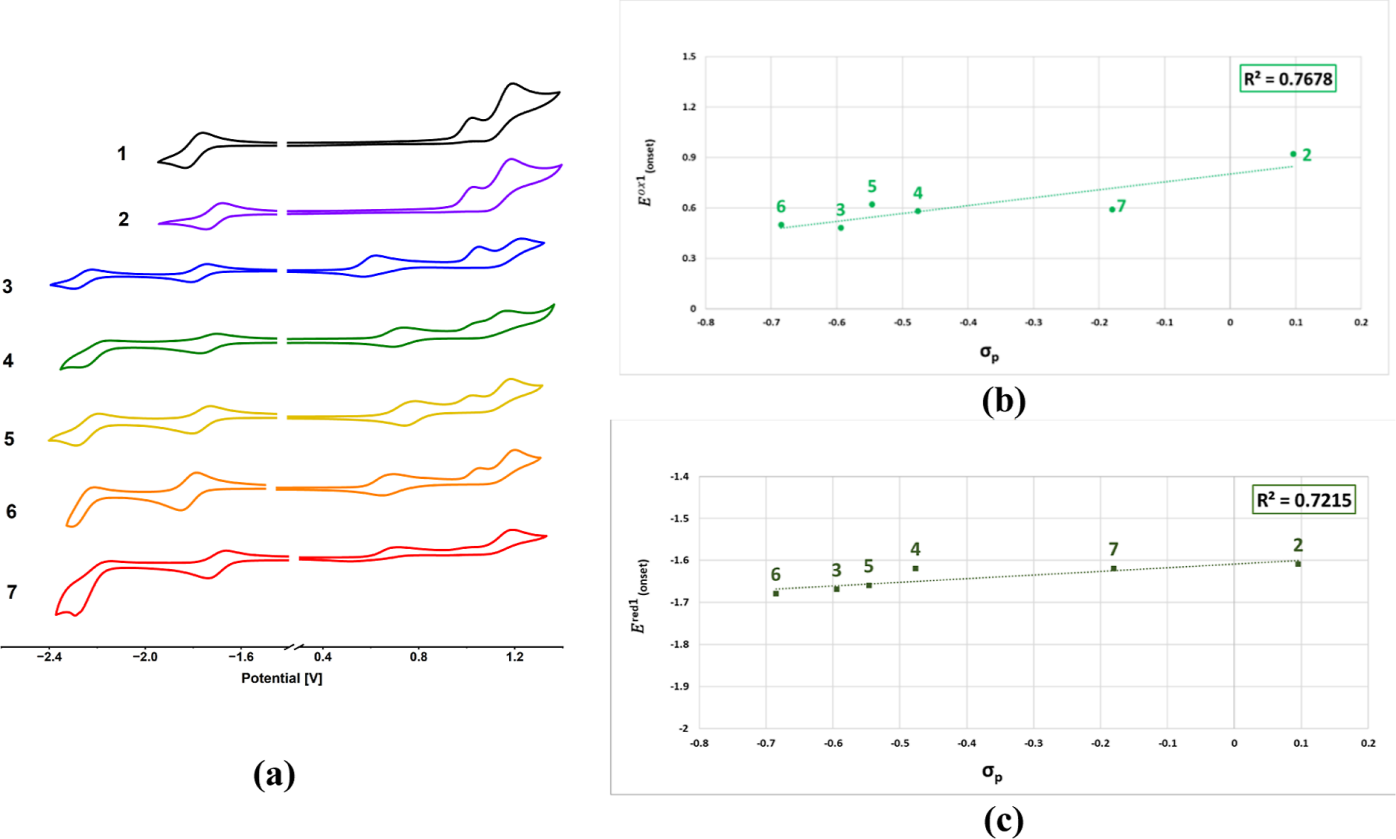
CV curves of complexes **1–7** (a) and linear correlation of *E*_onset_^ox1^ (b) and *E*_onset_^red1^ (c) of **2–7** with the calculated σ_p_ parameter of the R-group (b).

Typical of heteroleptic iridium­(III) complexes with diimines as ancillary ligands,^63,64,66^ the first quasi-reversible reduction can be safely assigned to the reduction of the terpy core, coordinated in a bidentate mode. The reduction of cyclometalating ligand (Ph-bztz) occurs at lower potentials. As shown in Figure 2 and Table 1, incorporation of the amine electron-donating group induces a slight cathodic shift in the reduction peak potential relative to the model complex **2**, consistent with increased electron density on the terpy core. In conclusion, functionalization of Ph-terpy with acyclic and cyclic amine groups leads to noticeable destabilization of the HOMO level, being the largest in the case of piperidine and dimethylamine, and thus results in a decrease in the HOMO–LUMO gap of amine-substituted Ir­(III) complexes compared to the model chromophores. The variations in LUMO energy levels of **3**–**7** are considerably less significant compared to the model chromophores.

### Absorption and Photo- and Electroluminescence Properties

The absorbance behavior of **1**–**7** was investigated in two solvents of different polarities (CH_3_CN and CHCl_3_) and in the solid state as films on a glass substrate. The data are presented in Figure 3 and in ESI (Table S7 and Figures S6–S9). The solid-state spectral profiles of **1**–**7** closely match those in solution, with only small differences in the position of the absorption band maximum (λ_max_) (Table S7 and Figure S6b).

**Figure 3:**
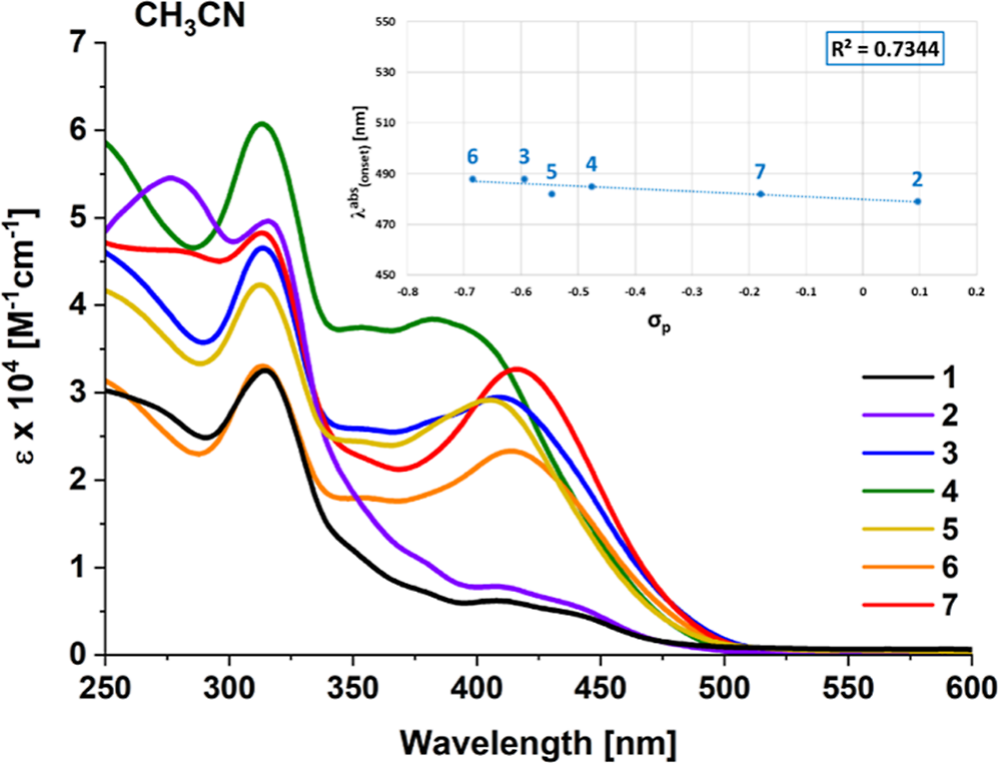
UV–vis spectra of [Ir­(Ph-btz)_2_(R-terpy-κ^3^N)]­PF_6_ complexes (**1**–**7**) in CH_3_CN solution and linear correlation of λ_onset_^abs^ of **2–7** with the calculated σ_p_ parameter of the R-group (inset).

The high-energy region (225–350 nm) of the UV–vis spectra of **1**–**7** shows strong absorptions attributed to spin-allowed π → π* transitions within the R-terpy and Ph-btz ligands (Figure S6). The weaker bands in the range 350–525 nm originate predominantly from charge transfer (CT) transitions, tentatively assigned to ^1^MLCT and ^1^LLCT (frequently assigned as ^1^MLLCT) for model chromophores (**1** and **2**) and to a combination of ^1^MLCT, ^1^LLCT, and ^1^ILCT for the others.^56,57,69–72^

Distinctions among the complexes **2**–**7** become apparent upon analysis of the onset of the lowest-energy absorption band in acetonitrile as a function of the calculated σ_p_ parameter of the R-group.^68^ As shown in Figure 3, decreasing σ_p_ values are associated with bathochromic shifts of onsets of lowest-energy absorption bands, supporting the contribution of ^1^ILCT to the lowest-energy absorption maxima of **3**–**7**. Consistent with the contribution of ^1^ILCT transitions associated with charge flow from the donor substituent to the terpy acceptor core, the complexes bearing appended amine groups (**3**–**7**) exhibit markedly enhanced molar visible absorptivity relative to **1** and **2**. Exemplarily, the molar extinction coefficient of **4** (4.08 × 10^4^ dm^3^·mol^–1^·cm^–1^) exceeds about 8 times the values for the model chromophores (0.47 and 0.56 dm^3^·mol^–1^·cm^–1^).

Relative to the parent complexes, the lowest-energy absorption maxima of **3**–**7** experience either a slight hypsochromic shift or remain almost unchanged (Table S7). The most pronounced blue shift is observed for **4** in CH_3_CN (Figure 3 and Table S7). This behavior is in contrast to that observed for Re­(I) carbonyl complexes of the type [ReCl­(CO)_3_(R-C_6_H_4_-terpy-κ^2^N)], in which introduction of electron-donating amine substituents into Ph-terpy produces a clear red shift of the longest-wavelength absorption band.^40,41,49^ Such findings may be indicative of a smaller contribution of the ^1^ILCT component to the lowest energy absorption of [Ir­(Ph-btz)_2_(R-C_6_H_4_-terpy-κ^2^N)]­PF_6_ compared to [ReCl­(CO)_3_(R-C_6_H_4_-terpy-κ^2^N)] systems.

For all Ir­(III) complexes with appended remote amine substituents (**3**–**7**), ^1^MLCT/ILCT absorptions in CH_3_CN occur at shorter wavelengths than those recorded in less polar CHCl_3_, supporting a negative solvatochromism in these systems (Figure S8). The largest difference between the visible absorption maxima in CHCl_3_ and CH_3_CN (∼25 nm) was revealed in the case of **4** and **7**. On the contrary, the parent complexes (**1** and **2**) display negligible solvatochromic effects. The incorporation of the phenyl into the terpy core invokes minor changes in both the absorption maxima and molar extinction coefficients of **2** in relation to **1**.

All of the designed compounds show stability and photostability in solution. As demonstrated in Figure S9, there are no noticeable changes in the absorbance profiles of these systems in UV–vis spectra recorded at regular time intervals for 12 h and after light irradiation at 420 nm.

Photoluminescence properties of **1**–**7** were investigated in solution at room temperature and in the solid state and rigid-glass matrix (77 K). The emission spectral data are presented in Table 2 and Figures 4–6, as well as provided in ESI (Figures S10–S23). Excited-state lifetimes in the microsecond or submicrosecond domain (Table 2) along with a substantial decrease in the photoluminescence intensities and lifetimes in the presence of molecular oxygen (Figure S17) support the triplet excited state as the origin of the emission in all systems. The inclusion of the amine group into the Ph-terpy core induced noticeable perturbations in the emission characteristics of [Ir­(Ph-btz)_2_(R-C_6_H_4_-terpy-κ^2^N)]­PF_6_ (**3**–**7**) relative to the parent complexes (**1** and **2**). The differences in the emission behavior between non- and amine-substituted systems concern the phosphorescence energies, lifetimes, quantum yields (QYs), rigidochromic effect, and sensitivity to solvent polarity, as shown in Figures 4–6 and Table 2.

**Figure 4:**
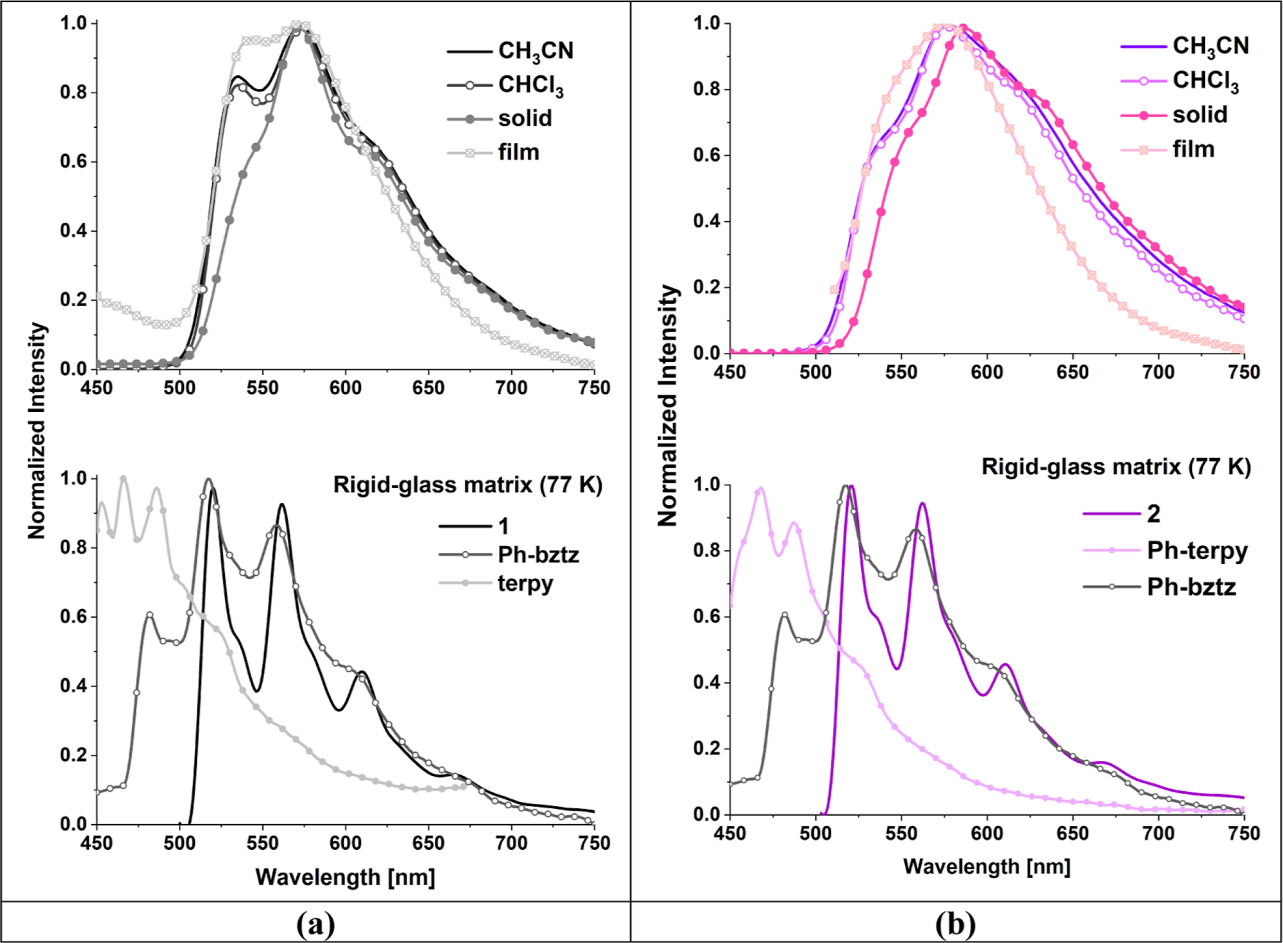
Emission spectra of **1** (a) and **2** (b) in solution at room temperature, the solid state as powder and film, and the rigid-glass matrix (77 K) alongside the phosphorescence of 2-phenylbenzothiazole and terpy and Ph-terpy. The free ligands were sensitized toward phosphorescence with addition of 10% (v/v) of ethylene iodide.

**Figure 5:**
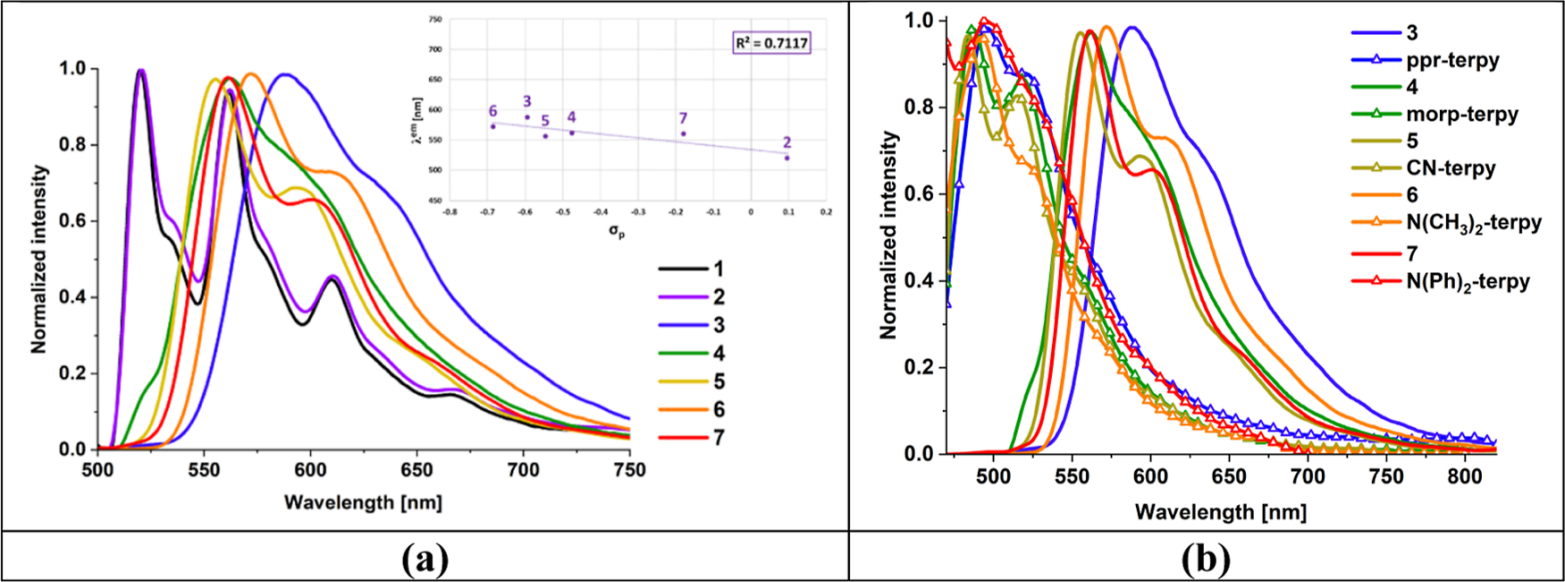
(a) Phosphorescence spectra at 77 K of the amine-substituted systems (**3**–**7**) relative to those of the model chromophores (**1** and **2**) and solution and linear correlation of λ^em^ of **2–7** with the calculated σ_p_ parameter of the R- group (inset). (b) The frozen-state phosphorescence spectra of the amine-substituted systems (**3–7**), along with the phosphorescence spectra of R-C_6_H_4_-terpy at 77 K. The free ligands R-C_6_H_4_-terpy were sensitized toward phosphorescence with addition of 10% (v/v) of ethylene iodide.

**Figure 6:**
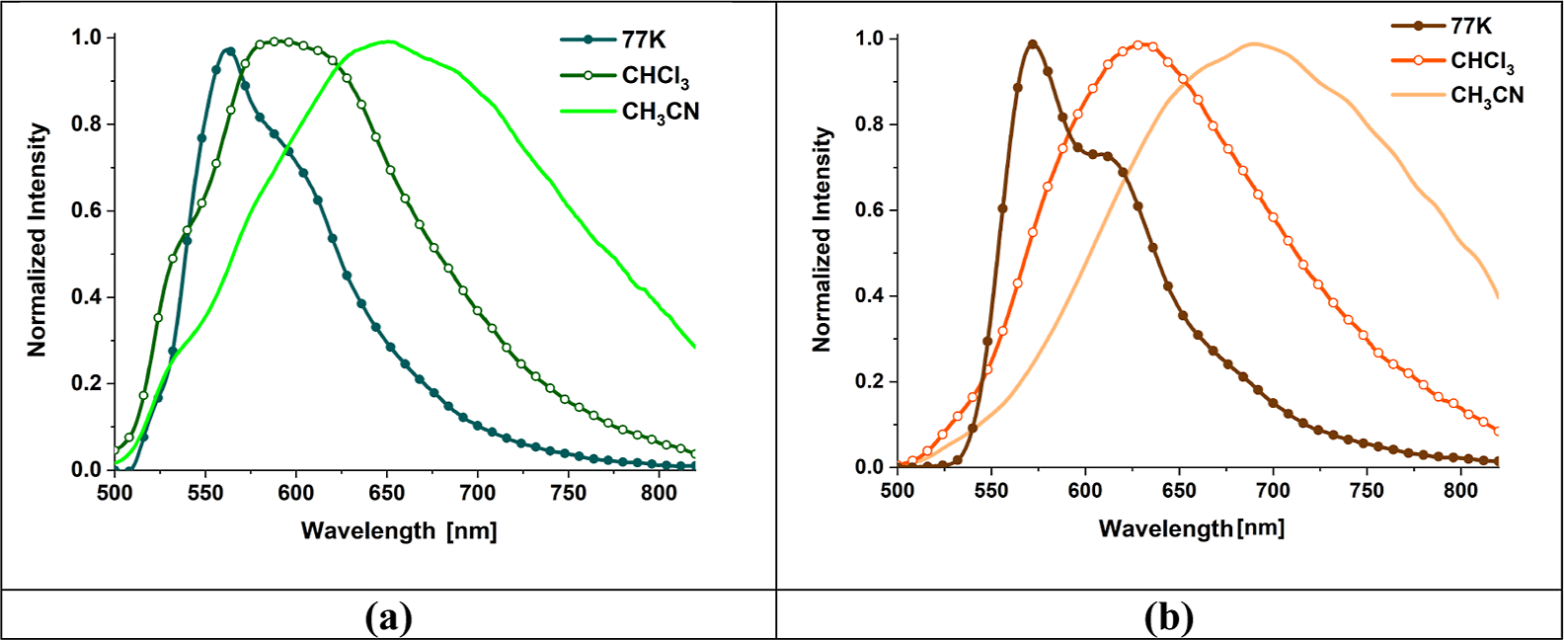
Normalized emission spectra of **4** (a) and **6** (b) in the matrix at 77 K and solution at room temperature. For other complexes, see Figure S19.

**Table 2:** Summary of Photoluminescent Properties of **1**–**7**

compound	medium	λ_exc_	λ_em_	PL lifetime (μs)	Χ^2^	QY (%)	kr (10^4^ s–1)^b^	knr (10^4^ s–1)^b^
**1**	CH_3_CN	410	535, 573, 620(sh)	Ar^c^: 0.66 (93.6%), 1.65 (6.4%)/τ_av_ = 0.72	1.086	7.25	10.07	128.82
				air: 0.44	0.958			
	CHCl_3_	410	535, 572, 620(sh)	Ar: 0.71 (79.0%), 1.61 (21.0%)/τ_av_ = 0.90	0.942	13.61	15.12	95.99
				air: 0.45 (34.74%), 0.79 (65.26%)/τ_av_ = 0.67	1.031			
	77 K^a^	430	520, 560, 610, 668	5.18 (94.3%), 17.66 (5.7%)/τ_av_:5.89	1.036			
	solid	470	540, 572, 618(sh)	1.25 (28.1%), 2.48 (71.9%)/τ_av_:2.14	1.101	0.05	0.02	46.71
	film	413	540, 571					
**2**	CH_3_CN	410	534, 578, 624(sh)	Ar: 0.51 (86.1%), 1.21 (13.9%)/τ_av_:0.61	1.131	7.82	12.82	151.11
				air: 0.36	1.11			
	CHCl_3_	410	534, 576, 617(sh)	Ar: 0.73 (84.4%), 2.53 (15.6%)/τ_av_:1.01	1.054	12.65	12.52	86.49
				air: 0.51 (61.7%), 0.85 (38.3%)/τ_av_:0.64	0.94			
	77 K^a^	430	520, 560, 610, 669	5.04	1.178			
	solid	470	550, 586, 630(sh)	0.42 (51.2%), 1.14 (48.8%)/τ_av_:0.77	1.125	4.69	6.09	123.78
	film	410	572					
**3**	CH_3_CN	425	680	Ar: 1.15	1.006	0.4	0.34	86.06
				air: 0.28	1.033			
	CHCl_3_	425	638	Ar: 1.08 (5.2%), 4.49 (94.8%)/τ_av_:4.31	1.169	2.44	0.57	22.64
				air: 0.79	1.35			
	77 K^a^	430	588, 640(sh)	36.64 (33.4%), 89.66 (66.6%)/τ_av_:71.96	1.117			
	solid	470	595, 620	0.67 (82.8%), 3.73 (13.8%), 21.84 (3.4%)/τ_av_:1.81	1.055	0.6	0.33	54.92
	film	422	574					
**4**	CH_3_CN	405	656	Ar: 1.22	1.033	4.47	3.66	78.3
				air: 0.30	1.04			
	CHCl_3_	405	595	Ar: 1.35 (6.5%), 4.52 (93.5%)/τ_av_:4.31	1.086	2.16	0.5	22.7
				air: 0.98	1.22			
	77 K^a^	430	562, 605	16.56 (35.9%), 54.49 (64.1%)/τ_av_:40.88	1.073			
	solid	470	580, 622(sh)	0.37 (71.8%), 1.35 (28.2%)/τ_av_:0.64	1.075	4.59	7.17	149.08
	film	422	537, 570					
**5**	CH_3_CN	410	614	Ar: 1.86	1.018	2.34	1.26	52.51
				air: 0.35	0.981			
	CHCl_3_	410	578	Ar: 0.93 (7.8%), 2.83 (92.2%)/τ_av_:2.68	1.127	11.61	4.33	32.98
				air: 1.23	1.17			
	77 K^a^	430	556, 595, 655(sh)	17.86 (41.5%), 49.94 (58.5%)/τ_av_:36.63	1.177			
	solid	470	564, 635(sh)	0.34 (37.1%), 2.61 (62.9%)/τ_av_:1.77	1.171	0.24	0.14	56.36
	film	416	585					
**60**	CH_3_CN	415	690	Ar: 1.15	1.053	0.8	0.7	86.26
				air: 0.21	1.091			
	CHCl_3_	415	630	Ar: 5.51	1.219	8.39	1.52	16.63
				air: 0.87	0.937			
	77 K^a^	445	572, 615, 685(sh)	31.75 (29.9%), 71.72 (70.1%)/τ_av_:59.75	1.029			
	solid	505	582, 630(sh)	0.80 (52.7%), 3.05 (47.3%)/τ_av_:1.87	1.205	3.94	2.11	51.37
	film	422	600					
**7**	CH_3_CN	415	516, 690	Ar (λe0.08390,0m = 510 nm): 0.02 (87.4%), 0.44 (12.6%)/τ_av_ = 0.07	0.896	2.16	30.86	1397.71
				(λem = 695 nm): 0.41	1.048		5.27	238.63
				air (λem = 510 nm): 0.01 (93.99%), 0.18 (6.01%)/τ_av_ = 0.02	0.989			
				(λem = 695 nm): 0.20	0.937			
	CHCl_3_	440	535(sh), 605	Ar (λem = 610 nm): 4.34	1.023	11.48	2.65	20.4
				air (λem = 610 nm): 0.95	0.907			
	77 K^a^	445	560, 600, 665	24.82 (46.3%), 68.22 (53.7%)/τ_av_:48.14	1.102			
	solid	470	580(sh), 600	2.87 (57.9%), 13.21 (42.1%)/τ_av_:7.22	1.062	11.13	1.54	12.31
	film	430	588					

a 77 K—measurement in the EtOH/MeOH (4:1 v/v) rigid matrix.

b Correlations between emission lifetimes (τ), quantum yields (QYs), and their radiative and nonradiative constants (*k*_r_ and *k*_nr_) were calculated according to the following equations: $\tau =\frac{1}{k_{r}+k_{nr}}$ and $QY=\frac{k_{r}}{k_{r}+k_{nr}}$ . *k*_r_ and *k*_nr_ were calculated: $k_{r}=\frac{QY}{\tau }$ and $k_{nr}=\frac{1-QY}{\tau }$ .

c Ar—deaerated conditions, air—deaerated conditions.

The excitation-independent emission bands of the model chromophores (**1** and **2**) in solution are nearly superimposable, display weak vibronic progression, and are marginally affected by the solvent polarity (Figures 4 and S22). With a decrease in temperature, a much sharper vibronic progression of the emission profile is developed, and the phosphorescence energy undergoes a slight blue shift. Weak rigidochromic behavior and large overlap with the phosphorescence of 2-phenylbenzothiazole suggest the predominant ^3^IL_Ph‑btz_-dominated nature of the triplet excited states of **1** and **2**.

As demonstrated in Figure S18, the replacement of Ph-py by Ph-btz in [Ir­(N^∩^C)_2_(Ph-terpy)]­(PF_6_) results in the change of the emission spectral profile and elongation of the lifetime of [Ir­(Ph-btz)_2_(Ph-terpy)]­(PF_6_) (5.05 μs at 77 K and 1.01 μs in CHCl_3_) relative to [Ir­(Py-py)_2_(Ph-terpy)]­(PF_6_) (4.24 μs at 77 K and 0.21 μs in CHCl_3_),^54^ indicating the switch of the triplet excited-state character from ^3^MLCT/IL in [Ir­(Ph-py)_2_(Ph-terpy)]­(PF_6_) to ^3^IL_Ph‑btz_^–^-dominated in [Ir­(Ph-btz)_2_(Ph-terpy)]­(PF_6_).

The phosphorescence spectra of the amine-substituted systems (**3**–**7**) measured at 77 K occur at noticeably longer wavelengths in relation to the parent complexes, and the emission spectral profile becomes less structured. At 77 K, their excited-state lifetimes are approximately 1 order of magnitude longer than those observed for complexes **1** and **2**. Furthermore, the emission energy is affected by the remote amine group, exhibiting a progressive bathochromic shift in the order **5** < **7** < **4** < **6** < **3** (Figure 5a and Table 2). The similarity between the emission profiles of the amine-substituted complexes (**3**–**7**) and the phosphorescence spectra of the corresponding R-C_6_H_4_-terpy ligands allows us to assume a large contribution of ^3^IL/ILCT_R‑terpy_ in the triplet excited states of **3**–**7** at 77 K (Figure 5b). A noticeable bathochromic shift of the emission of **3**–**7** relative to the phosphorescence of the appropriate R-C_6_H_4_-terpy is consistent with the enhanced electron-withdrawing ability of terpy upon coordination to the Ir­(III) center. Nevertheless, given the close energetic proximity of ^3^IL/ILCT_R‑terpy_ and ^3^IL_Ph‑btz_, contributions from ^3^IL_Ph‑btz_, as well as mixed ^3^LLCT and ^3^MLCT (assigned as ^3^MLLCT) excited states, cannot be excluded.

Upon transition from a 77 K matrix to room-temperature solution, the emission of the amine-substituted Ir­(III) complexes undergoes a pronounced bathochromic shift and becomes structureless (Figures 6 and S19). This behavior is characteristic of emission occurring from the excited state of a predominate ^3^CT character.

In analogy to the frozen-state phosphorescence spectra, the room-temperature emission of **3**–**7** is strongly impacted by the remote amine group, as illustrated in Figure 7.

**Figure 7:**
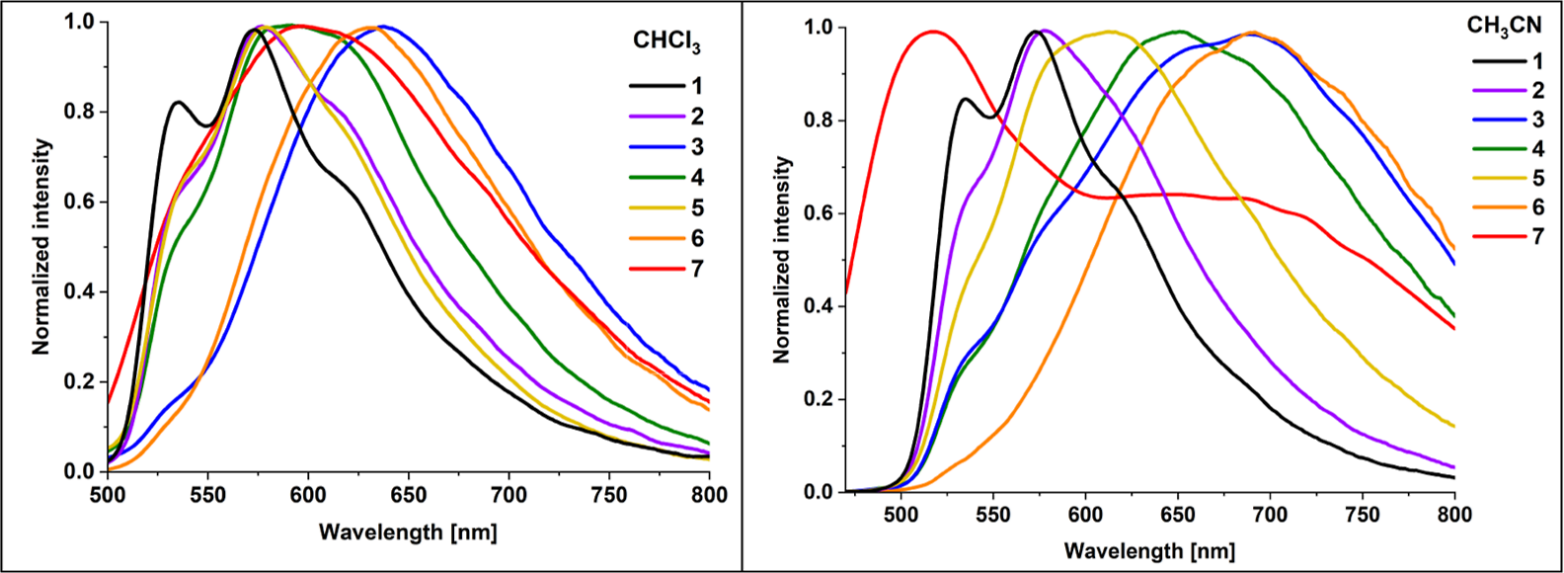
Room-temperature emission spectra of the amine-substituted systems (**3**–**7**) in CHCl_3_ (a) and CH_3_CN (b) relative to those of the model chromophores (**1** and **2**), recorded upon excitation at 440 nm.

Unlike the parent complexes but consistent with the predominate CT nature of the emissive triplet excited state, the RT emission of **3**–**7** shows pronounced solvent-polarity dependence. Upon going from CHCl_3_ to MeCN, the emission band becomes broader and red-shifted by approximately 40 nm for **3** and **5**, ∼60 nm for **4** and **6**, and ∼80 nm for **7**. In CHCl_3_, the emission maxima appear in the range of 590–640 nm, whereas in CH_3_CN, they shift to 638–690 nm. Except for **7**, which exhibits a dual fluorescence-phosphorescence emission and is discussed separately below, the emission energies of these systems correlate with the calculated σ_p_ parameters of the R-substituents,^68^ further highlighting the key role of the amine-substituted terpy ligands in determining the photophysics of [Ir­(Ph-btz)_2_(R-C_6_H_4_-terpy-κ^2^N)]­PF_6_. As shown in Figure S20, decreasing σ_p_ values are associated with bathochromic shifts of emission maxima.

Also, comparison of the photoluminescent data of **4** with those obtained for its analogue [Ir­(Ph-py)_2_(morph-C_6_H_4_-terpy-κ^2^N)]­PF_6_ ^54^ indicates a significant role of the amine-substituted terpyridine ancillary ligand in governing the photophysical behavior of [Ir­(Ph-btz)_2_(R-C_6_H_4_-terpy-κ^2^N)]­PF_6_. In contrast to the model chromophores (Figure S18), the replacement of Ph-py by Ph-btz leads to noticeably smaller variations in the emission energies (Figure S21). However, by analogy with **1** and **2**, the excited-state lifetimes are longer than those of [Ir­(Ph-py)_2_(morph-C_6_H_4_-terpy-κ^2^N)]­PF_6_.^54^

With respect to environmental polarity, noticeable differences between CHCl_3_ and CH_3_CN are also seen in the excited-state lifetimes and quantum yields of **3**–**7** relative to parent complexes (Table 2). Except for complex **7** in CH_3_CN, the appended electron-donating amine groups in [Ir­(Ph-btz)_2_(R-C_6_H_4_-terpy-κ2N)]­PF_6_ lead to an extended excited-state lifetime of **3**–**6** relative to the model chromophores, both in nonpolar and polar solvents. However, the extent of lifetime enhancement is markedly greater in the less polar solvent. Exemplarily, the excited-state lifetime of **6** (5.51 μs in CHCl_3_) was found to be approximately five times longer than those of parent complexes (0.90 μs for **1** and 1.01 μs for **2**). In contrast, all chromophores bearing amine-functionalized terpys exhibit reduced emission quantum yields compared to the unsubstituted systems, with the decrease being more pronounced in acetonitrile. These results may suggest the presence of two ^3^CT and ^3^IL triplet excited states in energy proximity, whose relative contributions to the emissive triplet state strongly depend on the solvent polarity.^73^ As can be expected, the charge transfer component, encompassing predominately ^3^ILCT_R‑terpy_ and ^3^MLCT_R‑terpy_ in complexes **3**–**7**, is preferentially stabilized in the polar environment, thereby gaining a significantly larger contribution in acetonitrile. The reduced lifetimes of **3**–**6** in polar acetonitrile may, to some extent, be rationalized by the pronounced emission bathochromic shift observed with increasing solvent polarity.

Taking into consideration the excitation wavelength emission dependence of amine-substituted Ir­(III) complexes in solution (Figures 8a and S22), complex **7** exhibits photoluminescence behavior that is distinctly different from the other systems studied. Excitation in the range of 350–420 nm of **7** in acetonitrile leads to the population of two well-separated excited states, resulting in a dual emission. The higher energy component of **7** is superimposed with the R-C_6_H_4_-terpy fluorescence (^1^IL), but its intensity is noticeably quenched compared to that of the corresponding free ligand (Figure S23). This behavior is consistent with incomplete energy transfer from ^1^IL_R‑terpy_ to lower energy ^1^MLLCT/ILCT_R‑terpy_ excited states via the Förster resonance energy transfer (FRET) mechanism.^74–78^ The high purity of **7**, confirmed by ultra-performance liquid chromatography (UPLC) analysis (Figure S24), rules out impurity-related emission, while its photostability in solution excludes any ligand dissociation under irradiation. The coexistence of fluorescence and phosphorescence was further confirmed by the emission spectra recorded in BuCN at temperatures from 80 to 300 K (Figure 8b), Notably, the unique photophysical behavior of **7** correlates with the fact that Ph_2_N-C_6_H_4_-terpy is a rare example of a strongly emissive amine-functionalized R-C_6_H_4_-terpy ligand.^67,79^

**Figure 8:**
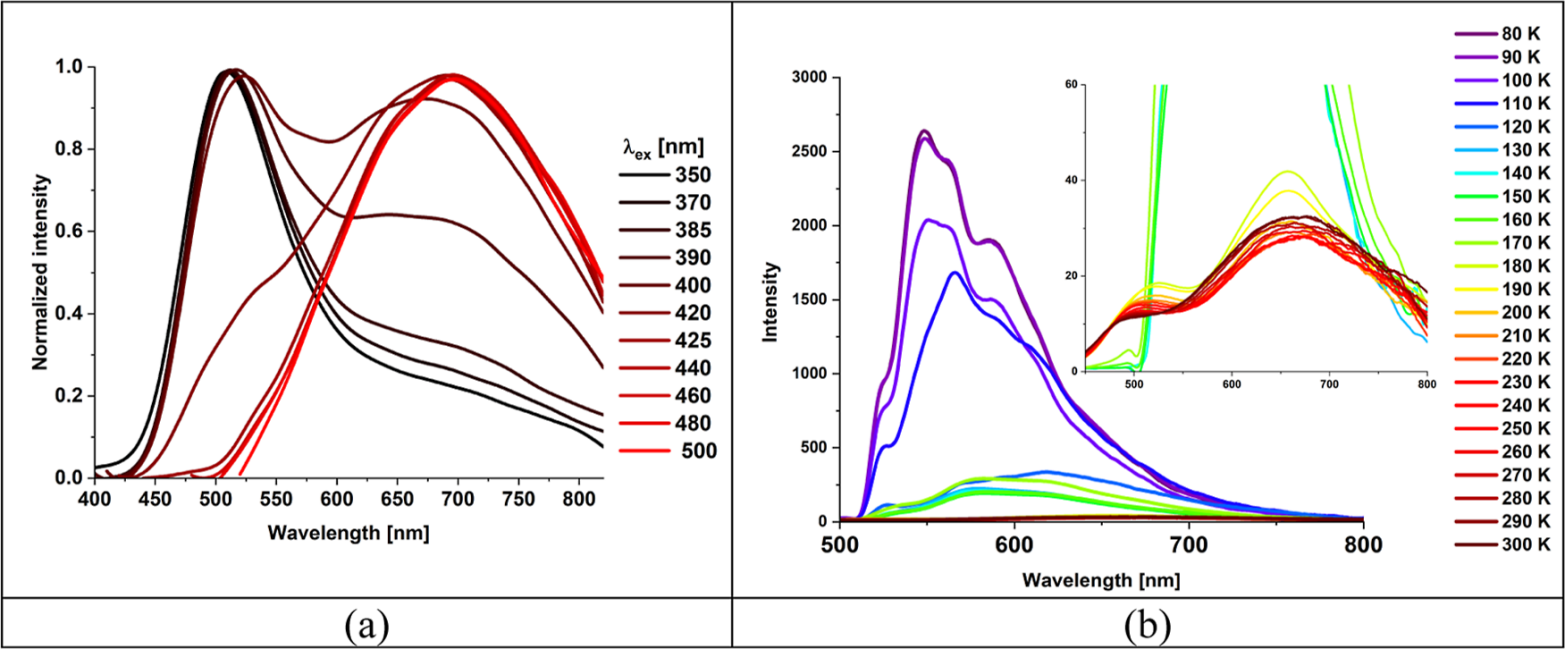
Emission spectra of **7** in CH_3_CN upon different excitation wavelengths (a); emission spectra of **7** in BuCN at different temperatures from 80 to 300 K (b).

Taken together, both static and time-resolved emission spectroscopy data for complexes **1**–**7** in solution allow us to conclude that the altered emission behavior of the amine-substituted Ir­(III) complexes relative to the parent chromophores arises from a change in triplet excited-state character, from ^3^IL_Ph‑btz_/MLCT in **1**–**2** to ^3^ILCT/IL/MLCT_R‑terpy_ in **3**–**7**.

All Ir­(III) complexes are also emissive in the solid state, both as powders and as thin films on glass substrates (Table 2 and Figure S25). The model chromophores exhibit solid-state emission maxima comparable to those observed in solution (MeCN and CHCl_3_). By contrast, the amine-substituted Ir­(III) complexes (**3**–**7**) display a pronounced hypsochromic shift in the solid state relative to their emission in acetonitrile, having emission energy more closely resembling that recorded in CHCl_3_. Remarkably, incorporation of the triphenylamine moiety results in a significant enhancement of the photoluminescence quantum yield for **7** compared with the model chromophores (Table 2 and Figure S25).

In the following stage of the study, the impact of the remote substituent in bis-cyclometalated complexes [Ir­(Ph-btz)_2_(R-C_6_H_4_-terpy-κ_2_N)]­PF_6_ on their electroluminescent (EL) performance was preliminarily investigated. Experiments included the registration of EL spectra from simple diode architectures in fixed geometry, with the neat complex forming the emissive layer (ITO/PEDOT/PSS/complex/Al), as well as from guest–host devices, in which the complex was molecularly dispersed (2 and 15 wt %) in a binary matrix composed of poly­(9-vinylcarbazole) (PVK) and 2-(4-*tert*-butylphenyl)-5-(4-biphenyl)-1,3,4-oxadiazole (PBD) (PVK/PBD, 50:50 wt %) (ITO/PEDOT/PSS/PVK/PBD/complex/Al). At this exploratory stage, quantitative OLED performance parameters were not determined. Representative EL spectra are shown in Figures 9 and S26, while the electroluminescence maxima (λEL) and corresponding peak intensities are summarized in Table S8.

**Figure 9:**
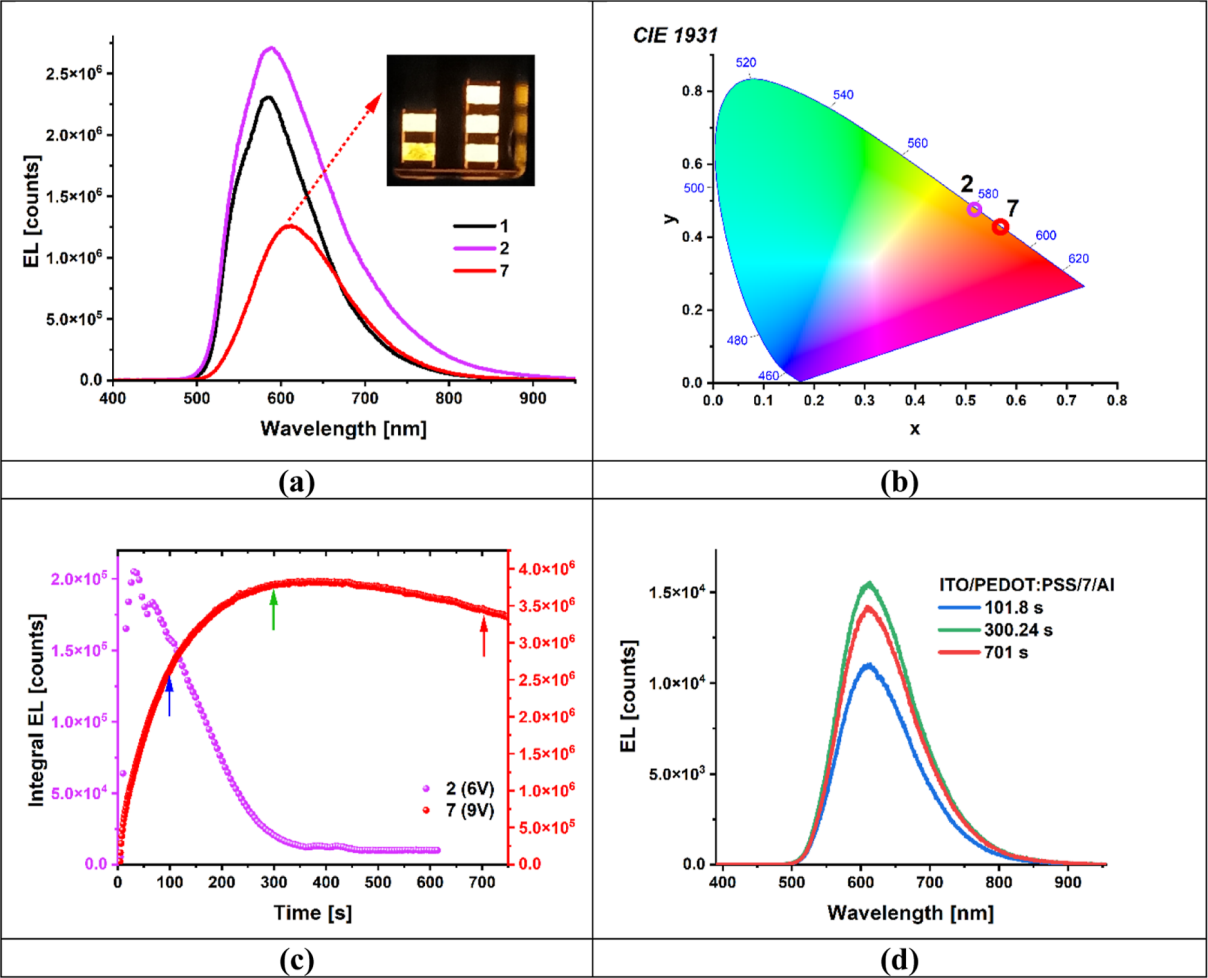
Electroluminescence spectra of exemplary diodes with structure ITO/PEDOT/PSS/complex/Al under an external voltage of 11 V and photo of devices (inset) (a); chromaticity diagrams of diodes ITO/PEDOT/PSS/2/Al and ITO/PEDOT/PSS/7/Al (model CIE 1931) (b); and the kinetics of EL integral intensity recorded as a function of time for ITO/PEDOT/PSS/2/Al and ITO/PEDOT/PSS/7/Al (c) and corresponding kinetics EL spectra of ITO/PEDOT/PSS/7/Al (d) (see also Figure S25).

All fabricated diodes exhibit light emission. As summarized in Table S8, the highest EL intensities were obtained from devices with the architecture ITO/PEDOT/PSS/complex/Al. A pronounced reduction in EL output was observed for the corresponding guest–host configurations (ITO/PEDOT/PSS/PVK/PBD/complex/Al), where the complexes were molecularly dispersed within the polymeric matrix.

**Table 3:** Calculated Phosphorescence Emission Energies alongside the Experimental Values

		TD-DFT		
complex	DFT Δ*E*_*T*1–*S*0_	main contribution	(eV)/(nm)	λ_exp_ [nm]
**1**	2.02 eV/613 nm	H → L (85%)	1.86/664	535, 573, 620
**2**	2.00 eV/620 nm	H → L (87%)	1.92/647	534, 578, 624
**3**	1.87 eV/662 nm	H → L (84%)	1.84/674	680
**4**	2.05 eV/603 nm	H → L (83%)	1.90/652	656
**5**	2.05 eV/603 nm	H → L (83%)	1.97/628	638
**6**	1.96 eV/634 nm	H → L (84%)	1.91/646	690
**7**	1.91 eV/648 nm	H → L (80%)	1.84/674	690

Concerning the devices based on the ITO/PEDOT/PSS/complex/Al configuration, the most favorable—in terms of intensity—electroluminescent performance was observed for those incorporating model chromophores and complex **7**, exhibiting emission in the yellow–orange and orange–red spectral regions, respectively. The investigations of the impact of applied external voltage on the EL performance revealed maximum emission intensities at approximately 11 V for devices incorporating complexes **1**, **2**, **4**, and **6**, whereas higher operating voltages of 15–16 V were required for diodes based on complexes **3** and **7** and approximately 18 V for the device containing complex **5** (Figure S26c). The temporal stability of light emission was examined for diodes ITO/PEDOT/PSS/complex/Al based on 2 and 7 (Figures 9 and S26). Notably, the diode incorporating complex **7** displayed markedly enhanced operational stability relative to the device containing complex **2**.

Taken together, the electroluminescent characteristics identify the triphenylamine-functionalized Ir­(III) complex as a promising luminophore for OLED applications, warranting further optimization and comprehensive performance evaluation.

### Femto- and Nanosecond Transient Absorption

The character of the excited states of **1**–**7** was further investigated using femtosecond transient absorption (fs-TA) spectroscopy (Figures 10 and S27) and laser flash photolysis (Figures S28 and S29). All measurements were performed in degassed acetonitrile solutions under 355 nm laser excitation. Photostability of complexes **1**–**7** during the fs-TA experiments was verified by comparing their UV–Vis absorption spectra recorded before and after laser irradiation (Figure S30). The fs-TA data sets were subjected to global analysis using a linear, unidirectional sequential kinetic model implemented in the Optimus software,^80^ enabling deconvolution of the TA data into evolution-associated spectra (EAS) and providing the decay-associated spectra (DAS). The results of the fs-TA experiments, along with the corresponding global fitting analyses, are summarized in Figure 10 and in ESI (Figure S27). Owing to the strong spin–orbit coupling induced by the iridium center,^73,81^ intersystem crossing occurs on a time scale shorter than the temporal resolution of our experimental setup. Consequently, all observed positive transient absorption bands are assigned to triplet excited states.

**Figure 10:**
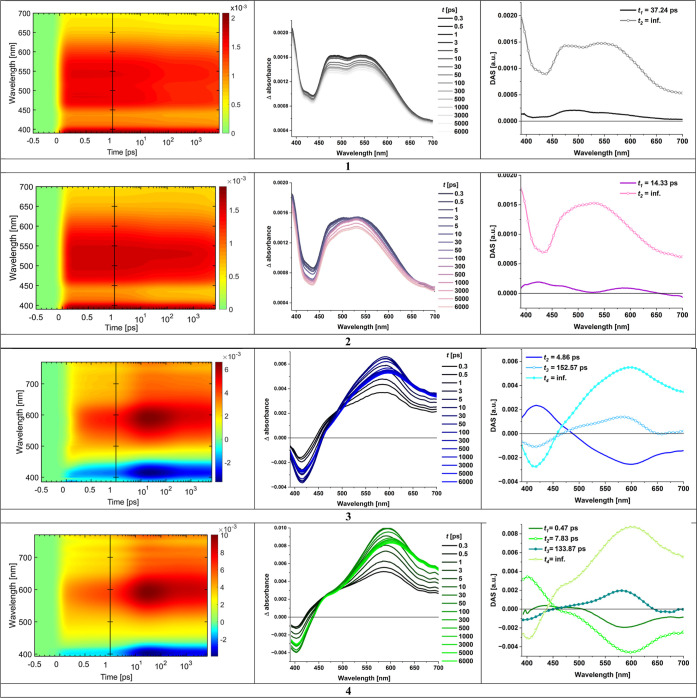

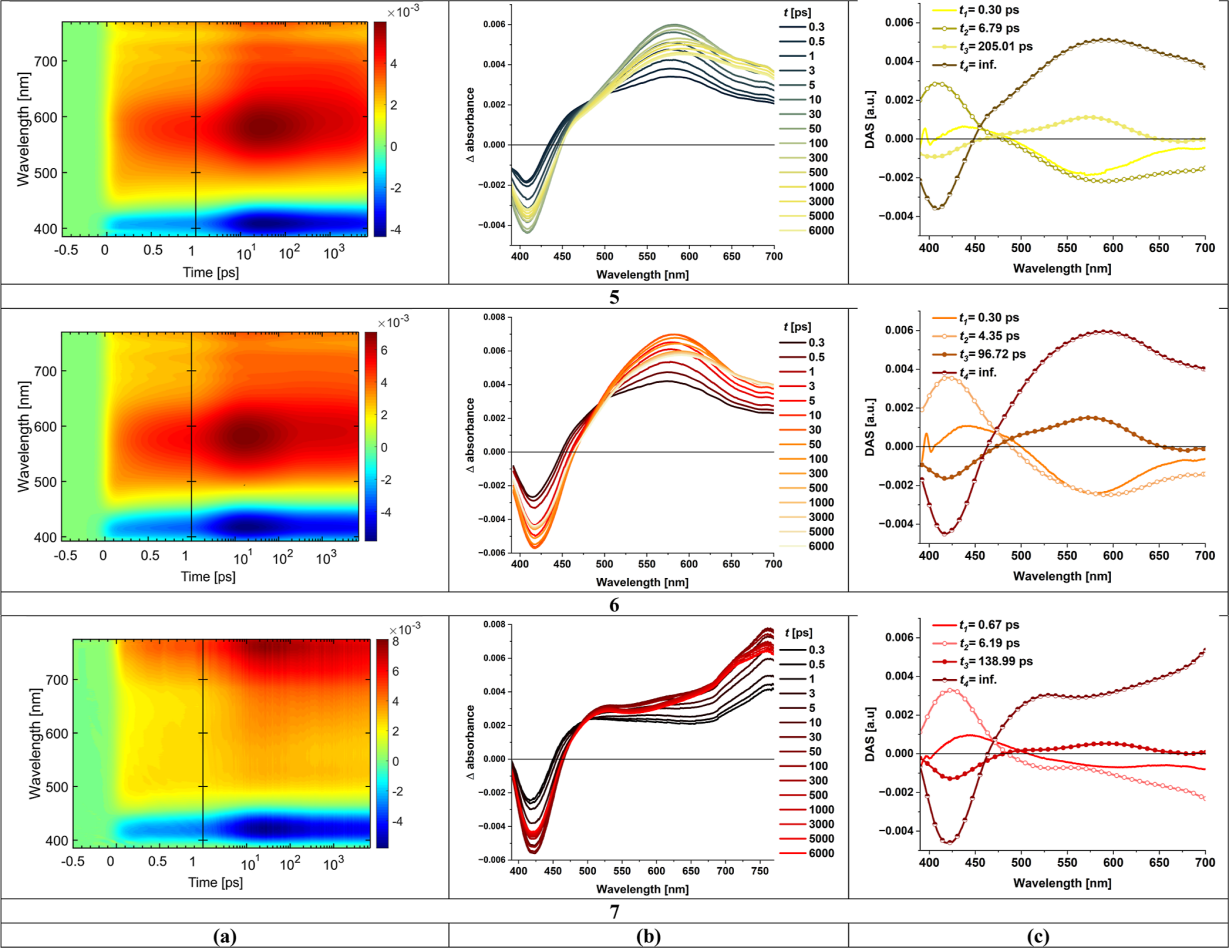
Fs-TA measurements for compounds **1–7** in MeCN upon 355 nm excitation: (a) time–wavelength 2D plots; (b) fs-TA spectra at selected picosecond delays; (c) decay-associated spectra (DAS_i_) with the corresponding time constants *t*_i_ (see Figure S27).

In analogy and agreement with the electrochemical and spectroscopic results discussed in the previous sections, transient absorption investigations also reveal a striking difference between the model chromophores and amine-substituted [Ir­(Ph-btz)_2_(R-C_6_H_4_-terpy-κ^2^N)]­PF_6_ complexes.

The TA spectra of **1** and **2** exhibit two excited-state absorption (ESA) bands with maxima in UV and Vis ranges (Figure 10). These bands appear after laser photoexcitation, and their spectral shapes and intensities undergo minimal changes over the applied time window (up to 7 ns). The pronounced dip in the range 405–460 nm, separating the ESA bands, coincides with the lowest-energy charge-transfer absorption of these systems and is therefore assigned to ground-state bleaching (GSB). In accordance with previous reports on bis-cyclometalated Ir­(III) complexes,^63^ such spectral features correspond to the population of triplet excited state ^3^IL_Ph‑btz_/MLCT. Global lifetime analysis (GLA) indicates that the fully relaxed ^3^IL_Ph‑btz_/MLCT is formed within the first several tens of picoseconds, 37.24 ps for **1** and 14.33 ps for **2**. This slow component in GLA is attributed to the complex geometrical relaxation and diffusive solvation response.^73^ Comparison of the fsTA profiles of **1** and **2** at 6 ns with those obtained by laser flash photolysis (20 ns) reveals a decrease in absorption intensity in the 525–650 nm region with increasing delay time. The ns-TA spectra of **1** and **2** show high similarity to ns-TA spectral features of the ^3^IL_Ph‑btz_ excited state, thereby supporting the key role of the cyclometalating ligand in controlling the photophysical properties of the model chromophores (Figure 11). The triplet excited-state lifetimes deduced from the decay of the ns-TA signals are in good agreement with those obtained from the decay of the emission signals, confirming that the transient absorbing excited state corresponds to the same triplet excited state responsible for the emission (Table S9). Complete laser flash photolysis data are provided in Figure S28.

**Figure 11:**
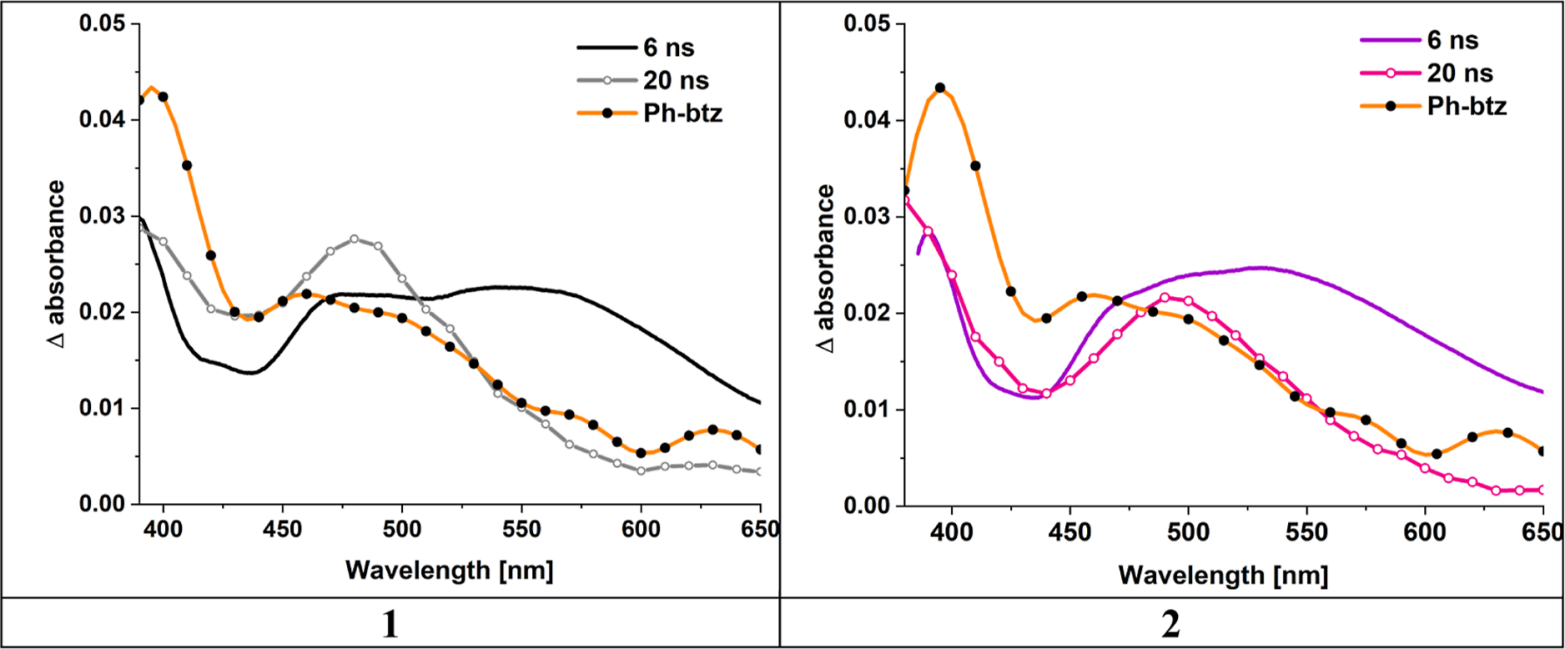
Comparison of the TA spectral profiles of the model chromophores recorded at 6 ns delay time in the fs-TA experiments with the earliest delay-time spectra of **1–2** and the free cyclometalating ligand obtained by laser flash photolysis.

In contrast to the model chromophores, which display only positive TA signals, the amine-substituted Ir­(III) complexes exhibit pronounced GSB in the region of their ^1^MLCT/ILCT charge-transfer absorptions in the UV–Vis spectra and a broad ESA band extending from 460 to 700 nm. Both negative and positive TA features appear after laser photoexcitation and persist throughout the delay window (up to 7 ns). With a reference to our previous reports on [ReCl­(CO)_3_(R-terpy-κ^2^N)] bearing strong electron-donating groups, transient excited-state absorption features of the amine-substituted Ir­(III) complexes can be assigned to the ^3^ILCT/IL_R‑terpy_ excited state admixed with ^3^MLCT_R‑terpy_ (Figure 12). The ILCT contribution is also clearly evident for the triphenylamine (TPA)-substituted complex (**7**). The TA spectra of **7** show excited-state absorptions typical of TPA^•+^ in the range of 700–800 nm.^50,82^ The characteristic excited-state absorption of the *N*,*N*-dimethylaniline radical cation is expected at ∼470 nm.^83^

**Figure 12:**
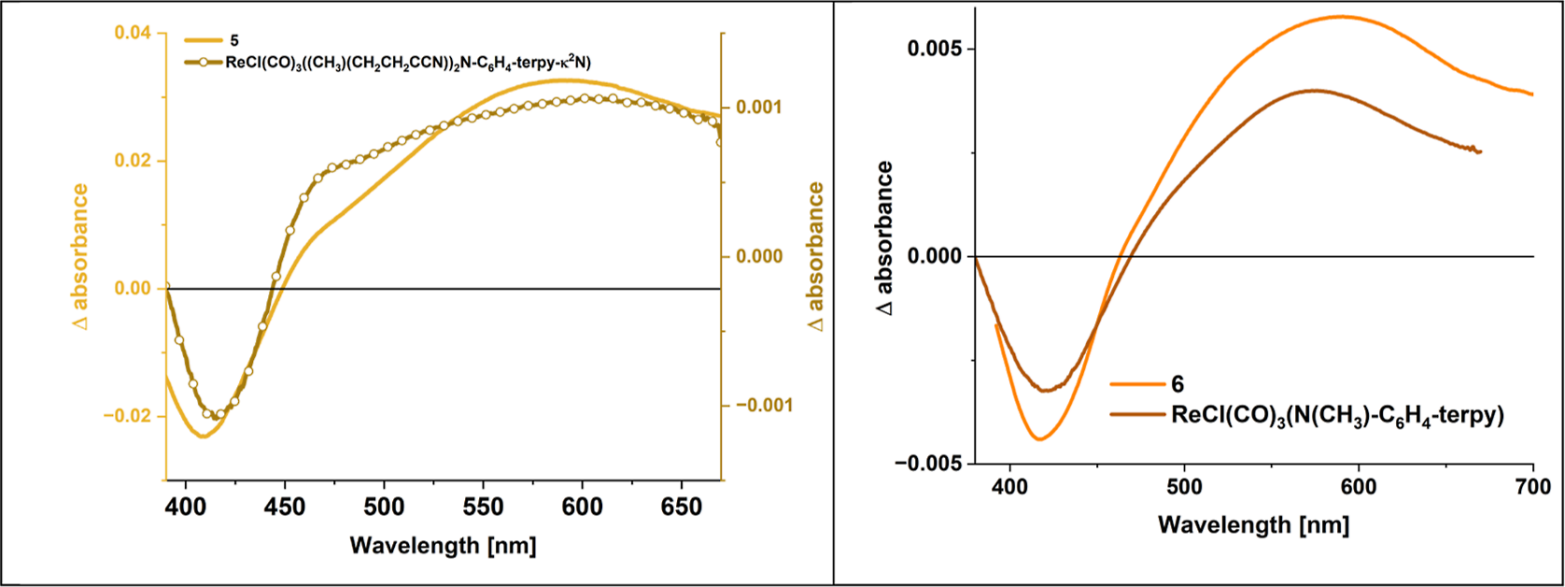
TA spectra of **5** and **6** recorded at 6 ns versus TA spectra of [ReCl­(CO)_3_((CH_3_)­(CH_2_CH_2_CN)­N-C_6_H_4_-terpy-κ^2^N)] and [ReCl­(CO)_3_(Me_2_N-C_6_H_4_-terpy-κ^2^N)] at 6 ns,^49^ respectively. Reproduced from ref 49. Available under a CC BY 4.0 license. Copyright © 2022 by the authors Joanna Palion-Gazda et al., published by MDPI, Basel, Switzerland.

Based on the early-time spectral evolution and previous reports on related systems,^47,49^ we assume that the final ^3^ILCT/IL/MLCT_R‑terpy_ excited state is formed via two paths. In analogy with [ReCl­(CO)_3_(R-C_6_H_4_-terpy-κ^2^N)] complexes bearing strong electron-donating substituents, 355 nm photoexcitation of **3**–**7** predominantly populates ^1^ILCT/IL_R‑terpy_ and ^1^MLCT, making possible the following processes: ^1^MLCT → ^3^MLCT → ^3^ILCT/IL and ^1^ILCT/IL → ^1^MLCT → ^3^MLCT → ^3^ILCT/IL to form ^3^ILCT/IL/MLCT_R‑terpy_. In agreement with the literature,^73,81^ ISC occurs in the time range shorter than the instrument response, so ESA features appearing just after photoexcitation correspond to triplet excited states.

In contrast to [ReCl­(CO)_3_(R-C_6_H_4_-terpy-κ^2^N)], the amine-substituted Ir­(III) complexes also access the ^3^ILCT/IL/MLCT_R‑terpy_ state via a ligand–ligand charge-transfer (LLCT) pathway, involving electron density transfer from the Ph-btz cyclometalating ligand to the R-C_6_H_4_-terpy ancillary ligand. It is reflected in the early-time spectral evolution. During the first few picoseconds, growth of the ESA band is accompanied by increased GSB negativity, indicating overlap of GSB with the UV ESA observed in the model chromophores. In the global lifetime analysis, this process is represented by the DAS_2_, characterized by positive signals at 380–450 nm and negative features above 450 nm.

As illustrated in Figure 13, the relaxed fs-TA spectral profiles of the amine-substituted Ir­(III) complexes generally match rather well those obtained with the laser flash photolysis technique. Complete laser flash photolysis data are provided in Figures S28 and S29.

**Figure 13:**
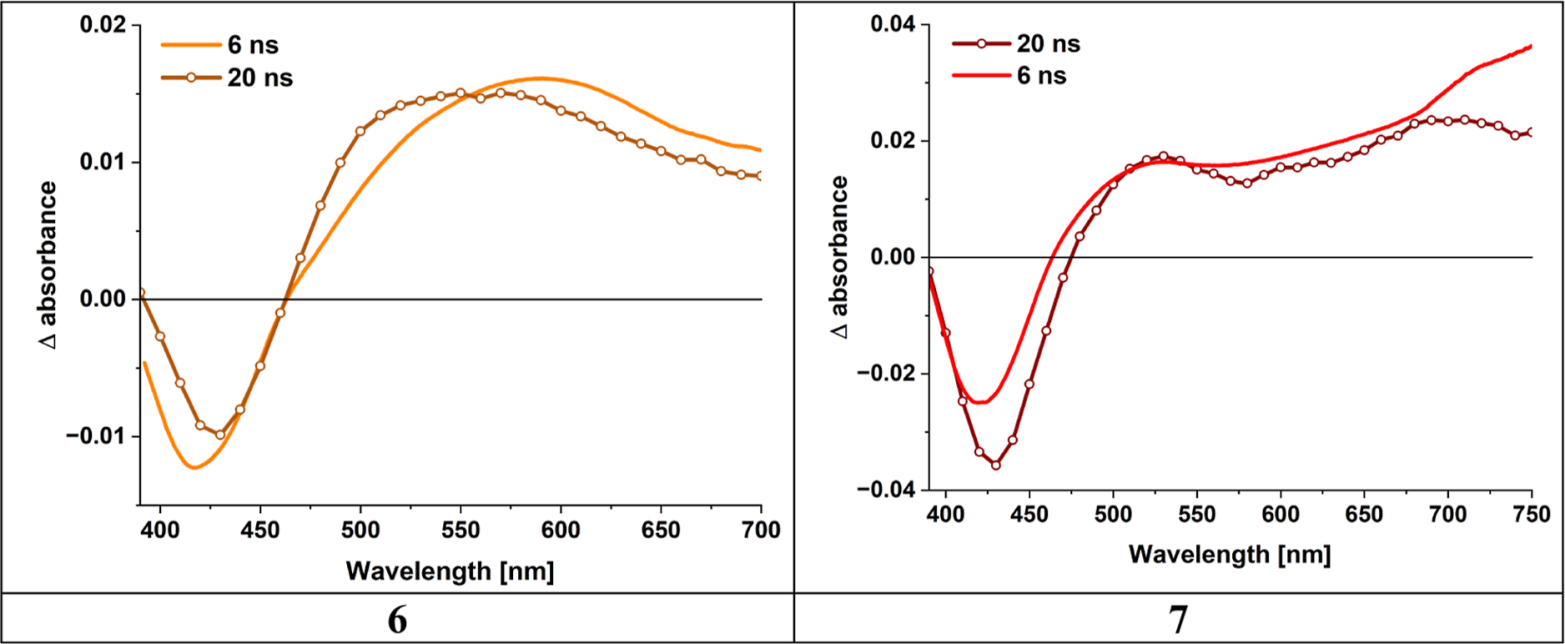
Comparison of the TA spectral profiles of the complexes **6** and **7** recorded at a 6 ns delay time in the fs-TA experiments with their earliest delay-time spectra obtained by laser flash photolysis. For other complexes, see Figure S29.

## Theoretical Calculations

The role of the amine group in controlling the photophysical properties of [Ir­(Ph-btz)_2_(R-C_6_H_4_-terpy-κ^2^N)]­PF_6_ was further evaluated theoretically at the DFT/PBE1PBE/SDD/def2-TZVP level. The results confirmed a profound difference between the unsubstituted (**1**–**2**) and amine-functionalized (**3**–**7**) chromophores and support the electrochemical and optical trends discussed above. Incorporation of the electron-donating amine groups alters the electron density distribution of the highest occupied molecular orbital (HOMO) and perturbs the energies of the frontier molecular orbitals relative to the parent chromophores. Calculated energy level diagrams and the frontier molecular orbitals for complexes **1**–**7** are presented in Figure 14.

**Figure 14:**
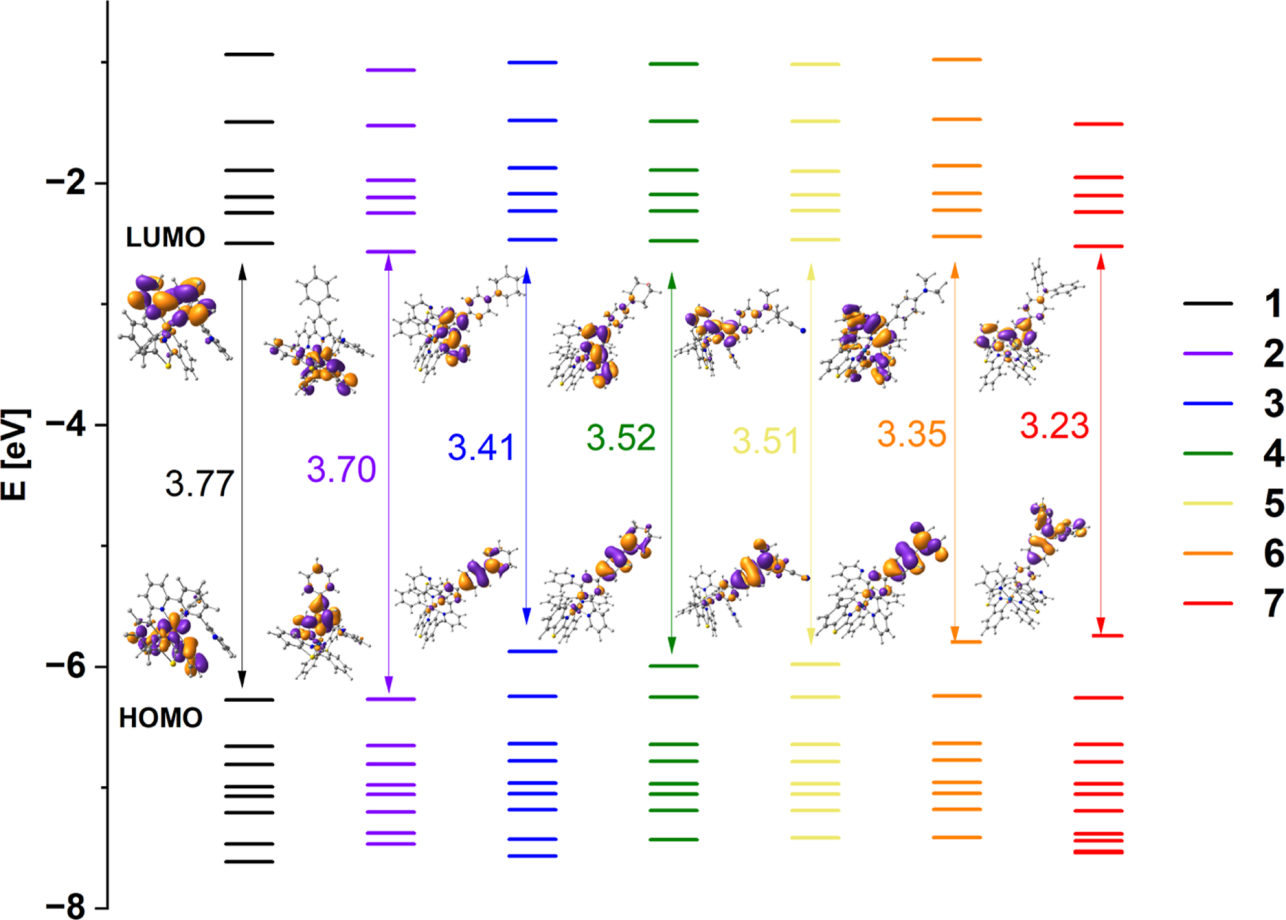
Calculated energy diagrams and frontier molecular orbitals of **1–7**.

For the model chromophores **1** and **2**, HOMO is primarily distributed over the iridium d orbitals and the C-part of the cyclometalating ligand (Ph-btz). In contrast, for the amine-substituted [Ir­(Ph-btz)_2_(R-C_6_H_4_-terpy-κ^2^N)]­PF_6_ complexes (**3**–**7**), HOMO becomes predominantly localized on the R-C_6_H_4_-terpy ligand, with major contributions from the amine and phenylene moieties (Figures 14 and S30). This redistribution of the HOMO electron density is accompanied by an increase in the HOMO energy level compared to complexes **1** and **2**. The HOMO energies follow the following trend: **1** = **2** (−6.27 eV) < **4** (−5.99 eV) < **5** (−5.98 eV) < **3** (−5.88 eV) < **6** (−5.80 eV) < **7** (−5.74 eV).

Notably, in the cases of the amine-substituted [Ir­(Ph-btz)_2_(R-C_6_H_4_-terpy-κ^2^N)]­PF_6_ complexes (**3**–**7**), the HOMO-1 reflects closely the HOMO of the parent complexes. It remains comparably distributed over the iridium d orbitals and the cyclometalated carbon fragment of the Ph-btz ligand and exhibits a similar energy level to the HOMO of **1** and **2** (Figure 14). The pronounced destabilization of orbitals localized on the amine and phenylene fragments of the R-C_6_H_4_-terpys, accompanied by a shift in HOMO character from predominantly metal-centered in unsubstituted systems to ligand-centered in derivatives bearing strongly electron-donating substituents, has also been consistently observed across the entire series of previously reported [ReCl­(CO)_3_(R-C_6_H_4_-terpy-κ^2^N)] complexes.^40,41,44,49,50^ This modulation of the frontier orbital distribution provides a clear rationale for the tunability of the photophysical properties, which arises from the increasing contribution of photoinduced intraligand charge-transfer (ILCT) transitions in the functionalized derivatives.

The LUMO in **3**–**7** complexes is predominantly associated with the π* orbitals of the pyridine rings of R-terpy coordinated to the metal ion and is slightly destabilized upon amine substitution, with a maximum shift of 0.13 eV observed for **6** (namely, from −2.57 eV for **2** to −2.44 eV for **6**). As a consequence, the HOMO–LUMO energy gaps of complexes **3**–**7** are reduced compared to those of the model chromophores. The spin allowed HOMO → LUMO electronic transition in complexes **3**–**7** corresponds to an intraligand charge transfer (^1^ILCT) from the electron-donating amine substituent toward the terpy acceptor, contrary to the model chromophores, for which the HOMO → LUMO excitation is of ^1^MLLCT nature. The contribution of ^1^ILCT to the lowest energy absorption band accounts for the markedly enhanced visible-light absorptivity observed in the amine-functionalized complexes (**3**–**7**). As shown in Tables S10–S16, the oscillator strengths of the ^1^ILCT transitions are substantially larger than those associated with the metal-to-ligand–ligand charge transfer (^1^MLLCT) transitions, which also contribute to the lowest-energy absorption band of **3**–**7**.

According to the theoretical calculations, the unsubstituted (**1**–**2**) and amine-functionalized chromophores (**3**–**7**) also differ in the nature of their lowest-energy triplet excited states. For complexes **3**–**7**, the spin density of the triplet state is predominantly localized on the appended amine group and Ph-terpy part, with a minor contribution from iridium d orbitals (Figure 15). The emission of these systems originates from the ^3^ILCT/IL/MLCT_R‑terpy_ excited state. In contrast, for complexes **1** and **2**, the triplet-state spin density is distributed over the {[Ir­(Ph-btz)_2_} and terpy moieties, indicating ^3^IL/MLCT character. In line with experimental findings, the emission of **3**–**7**, as theoretically predicted, should occur at longer wavelengths relative to the model chromophores **1** and **2** (Table 3).

**Figure 15:**
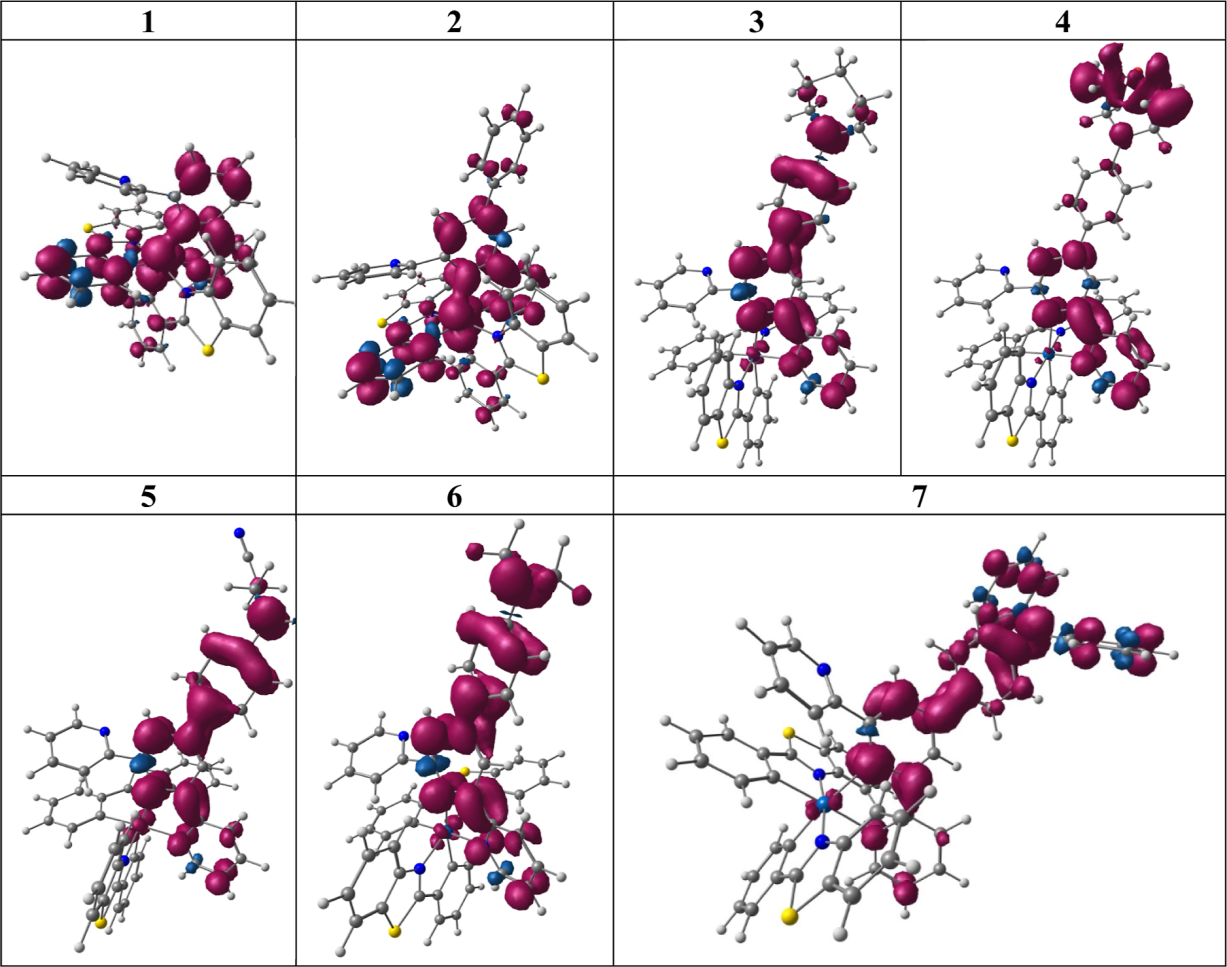
Spin density surface plots generated from the lowest energy optimized triplet states of [Ir­(Ph-btz)_2_(R-C_6_H_4_-terpy-κ^2^N)]­PF_6_ complexes. The isodensity surface plots of the LSOMO and HSOMO of **1**–**7** are shown in Figure S32.

## Conclusions

To investigate the impact of structural modifications of 2,2′:6′,2″-terpyridine-based ancillary ligands on the electrochemical and photophysical properties of bis-cyclometalated Ir­(III) complexes, a series of [Ir­(Ph-btz)_2_(R-terpy-κ^2^N)]­PF_6_ complexes were synthesized. The terpyridine ligands, which coordinate bidentately to the Ir­(III) center in these systems, were functionalized by strong electron-donating acyclic and cyclic amine groups attached via a phenylene spacer. The remote amine substituents were found to play a decisive role in controlling ground- and excited-state properties of [Ir­(Ph-btz)_2_(R-C_6_H_4_-terpy-κ^2^N)]­PF_6_. Compared to the parent chromophores [Ir­(Ph-btz)_2_(terpy-κ^2^N)]­PF_6_ (**1**) and [Ir­(Ph-btz)_2_(C_6_H_5_-terpy-κ^2^N)]­PF_6_ (**2**), the amine-substituted [Ir­(Ph-btz)_2_(R-C_6_H_4_-terpy-κ^2^N)]­PF_6_ complexes (**3**–**7**) undergo oxidation at significantly lower positive potentials, reflecting destabilization of the highest occupied molecular orbital (HOMO). As predicted theoretically, the HOMO of **3**–**7** is predominantly localized on the R-C_6_H_4_-terpy ligand, contrary to the model chromophores, where it is distributed over the iridium d orbitals and C-part of the cyclometalating ligand. Owing to the contribution of ^1^ILCT transitions, associated with charge flow from the donor substituent to the terpy acceptor core, the complexes bearing appended amine groups (**3**–**7**) exhibit substantially enhanced molar absorptivity in the visible range relative to **1** and **2**. Differences in emission behavior between the unsubstituted and amine-functionalized systems, including phosphorescence energies, lifetimes, quantum yields, rigidochromic effect, and sensitivity to solvent polarity, were interpreted by the change in the nature of the triplet excited states from ^3^IL_Ph‑btz_/MLLCT to ^3^ILCT/IL/MLCT_R‑terpy_. This conclusion was supported by both static and time-resolved photoluminescence spectroscopy and ultrafast transient absorption as well as theoretically by DFT and TD-DFT calculations. Remarkably, the room-temperature emission of **3**–**7** appears at longer wavelengths and is characterized by longer excited-state lifetimes relative to unsubstituted complexes (**1** and **2**). The complex incorporating the Ph_2_N-C_6_H_4_-terpy ancillary ligand uniquely exhibits dual fluorescence–phosphorescence emission upon higher-energy excitation, owing to incomplete Förster resonance energy transfer from the ^1^IL_R‑terpy_ state to the lower-energy ^1^MLLCT/ILCT_R‑terpy_ state. The electroluminescent studies identified the triphenylamine-functionalized Ir­(III) complex (**7**) as the most promising luminophore for OLED applications. Overall, the elucidation of the structure–property relationships provides insights into the origin and modulation of excited-state behavior in these systems, thereby offering a powerful foundation for the rational design and further development of phosphorescent materials.

## Experimental Section

### Materials

Commercially available iridium­(III) chloride hydrate, ammonium hexafluorophosphate, 2-phenylbenzothiazole (Ph-bztz), 2-acetylpyridine, benzaldehyde, 4-(1-piperidinyl)­benzaldehyde, 4-(4-morpholinyl)­benzaldehyde, 4-[(2-cyanoethyl)­methylamino]­benzaldehyde, 4-(dimethylamino)­benzaldehyde, and 4-(diphenylamino)­benzaldehyde were used as received without further purification. Reagent-grade solvents were used for synthesis, and HPLC-grade solvents were used for the spectroscopic measurements.

### Preparation of Ligands and Ir­(III) Complexes

The 2,2′:6′,2″-terpyridine derivatives were prepared via base-catalyzed Kröhnke condensation of 2-acetylpyridine with two equivalents of the corresponding aldehyde, according to published procedures.^40,67^

A mixture of [Ir_2_(μ-Cl)_2_(Ph-bztz)_4_] (0.17 g, 0.15 mmol) and the corresponding R-terpy ligand (0.32 mmol) was refluxed under an argon atmosphere in methanol/chloroform (1:3 v/v, 40 mL) for 24 h. After cooling the reaction mixture to room temperature, a saturated aqueous solution of (NH_4_)_2_PF_6_ was added dropwise, resulting in the formation of a yellow (**1** and **7**), orange (**2–4**), or brown (**5** and **6**) precipitate. The mixture was stirred for an additional 8 h, after which the precipitate was collected by filtration, washed with water and diethyl ether, and dried in air. The crude product was purified by column chromatography on neutral alumina using a CH_2_Cl_2_–MeOH mixture (2:1 v/v) as the eluent. Crystals suitable for X-ray analysis (**2** and **6**) were obtained by recrystallization from CH_2_Cl_2_/CH_3_CN.
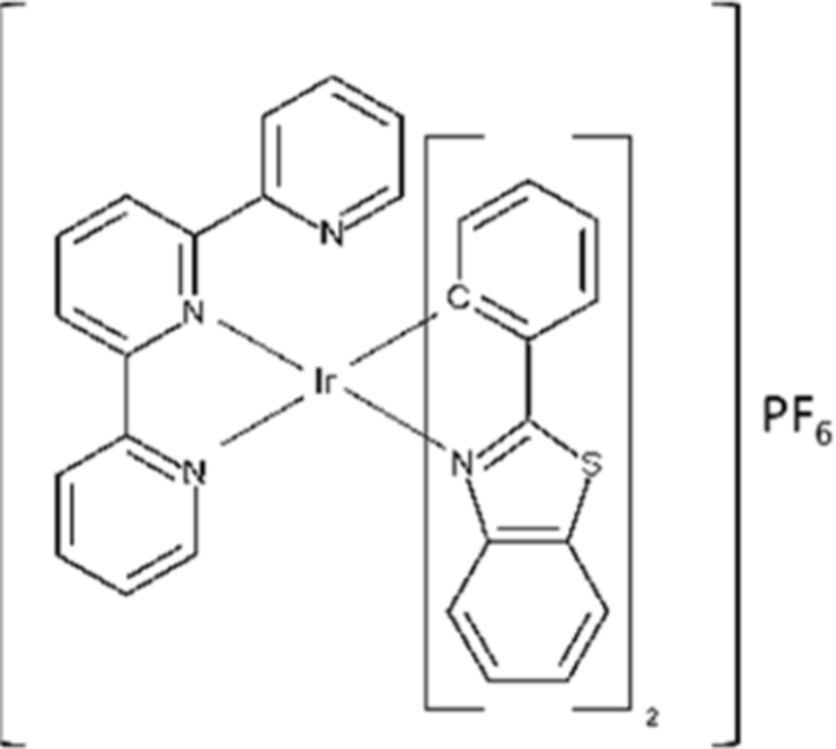


#### [Ir­(Ph-bztz)_2_(terpy-κ^2^N)]­PF_6_ (**1**)

Yield: 0.10 g, 66%. Anal. Calcd for C_41_H_27_F_6_IrN_5_PS_2_·CH_3_OH·CH_2_Cl_2_ (1107.97 g/mol): C, 46.61; H, 3.00; N, 6.32%. Found: C, 46.68; H, 3.24; N, 6.63. ^1^H NMR (500 MHz, DMSO-*d*_6_): δ 8.92–8.84 (m, 2H), 8.40 (t, *J* = 7.9 Hz, 1H), 8.31–8.22 (m, 3H), 8.01 (dt, *J* = 4.7, 1.4 Hz, 1H), 7.84 (dd, *J* = 7.8, 1.4 Hz, 1H), 7.73 (dd, *J* = 5.6, 1.6 Hz, 1H), 7.70–7.63 (m, 2H), 7.50 (ddd, *J* = 8.3, 7.1, 1.1 Hz, 1H), 7.43 (ddd, *J* = 8.2, 7.1, 1.1 Hz, 1H), 7.40–7.32 (m, 2H), 7.23–7.13 (m, 2H), 7.03 (td, *J* = 7.5, 1.1 Hz, 1H), 6.96–6.89 (m, 2H), 6.85–6.80 (m, 1H), 6.84 (td, *J* = 7.5, 1.4 Hz, 1H), 6.63 (td, *J* = 7.4, 1.1 Hz, 1H), 6.49 (td, *J* = 7.5, 1.4 Hz, 1H), 6.21 (d, *J* = 8.4 Hz, 1H), 5.93 (d, *J* = 7.9 Hz, 1H), 5.85 (d, *J* = 7.6 Hz, 1H) ppm. ^13^C­{H} NMR (126 MHz, DMSO-*d*_6_): δ 181.79, 180.29, 163.06, 163.04, 157.11, 157.01, 153.48, 151.49, 149.67, 149.05, 148.38, 148.35, 148.32, 146.67, 140.58, 140.31, 139.36, 139.31, 135.87, 132.37, 132.24, 131.10, 130.58, 130.51, 129.92, 128.34, 128.06, 127.87, 126.77, 126.41, 126.24, 125.73, 125.32, 124.70, 124.60, 124.51, 124.02, 123.40, 121.07, 118.00, 116.78 ppm. IR (KBr, cm^–1^) intensity: vs–very strong; s–strong, m–medium, w–weak: 3596 (w), 3422 (w), 3054 (w), 1734 (w), 1581 (m), 1568 (m), 1551 (m), 1531 (w), 1471 (m), 1448 (m), 1438 (m), 1407 (m), 1323 (w), 1298 (m), 1236 (w), 1167 (w), 1126 (w), 1050 (w), 1026 (w), 995 (w), 919 (w), 843 (s), 776 (m), 753 (s), 741 (m), 722 (m), 645 (w), 614 (w), 557 (s), 446 (w).
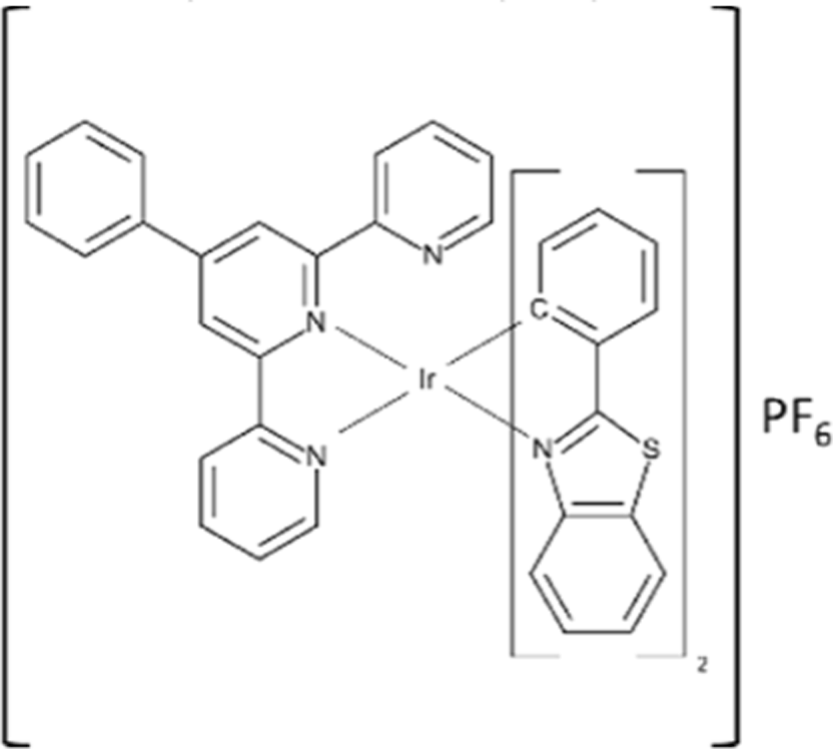


#### Ir­(Ph-bztz)_2_(Ph-terpy-κ^2^N)]­PF_6_ (**2**)

Yield: 0.12 g, 75%. Anal. Calcd for C_47_H_31_ F_6_IrN_5_PS_2_·CH_3_OH·H_2_O (1118.16 g/mol): C, 51.56; H, 3.43; N, 6.26%. Found: C, 51.85; H, 3.109; N, 5.92%. ^1^H NMR (500 MHz, DMSO-*d*_6_): δ 9.20–9.13 (m, 2H), 8.33–8.18 (m, 3H), 8.17–8.11 (m, 2H), 8.03–7.96 (m, 2H), 7.87–7.81 (m, 1H), 7.77–7.71 (m, 1H), 7.71–7.64 (m, 1H), 7.62–7.56 (m, 3H), 7.48 (td, *J* = 7.8, 2.4 Hz, 1H), 7.44–7.40 (m, 1H), 7.38–7.30 (m, 2H), 7.22 (t, *J* = 7.8 Hz, 1H), 7.19–7.13 (m, 1H), 7.06–6.98 (m, 2H), 6.97–6.88 (m, 2H), 6.89–6.81 (m, 1H), 6.67–6.59 (m, 1H), 6.48 (dt, *J* = 8.2, 4.7 Hz, 1H), 6.26 (dd, *J* = 8.6, 3.3 Hz, 1H), 5.97–5.91 (m, 1H), 5.88 (dd, *J* = 8.9, 3.5 Hz, 1H) ppm. ^13^C­{H} NMR (126 MHz, DMSO-*d*_6_): δ 181.79, 180.32, 163.74, 157.91, 157.13, 153.87, 151.44, 150.62, 149.68, 149.39, 149.17, 148.51, 148.35, 147.09, 140.13, 139.43, 139.36, 135.62, 134.28, 132.38, 132.25, 131.11, 130.60, 130.45, 129.44, 129.35, 128.33, 128.02, 127.91, 127.86, 127.47, 126.94, 126.76, 126.44, 126.41, 126.24, 125.80, 125.71, 124.58, 124.47, 123.94, 123.40, 123.34, 121.58, 121.02, 118.10, 116.91 ppm. IR (KBr, cm^–1^) intensity: 3666 (w), 3585 (w), 3049 (w), 3049 (w), 1614 (m), 1580 (m), 1567 (w), 1550 (w), 1470 (m), 1448 (m), 1437 (m), 1407 (m), 1322 (m), 1298 (m), 1267 (m), 1162 (w), 1124 (w), 1070 (w), 1050 (w), 1025 (w), 996 (w), 840 (s), 787 (m), 749 (s), 723 (m), 703 (m), 656 (w), 627 (w), 557 (m), 444 (m).
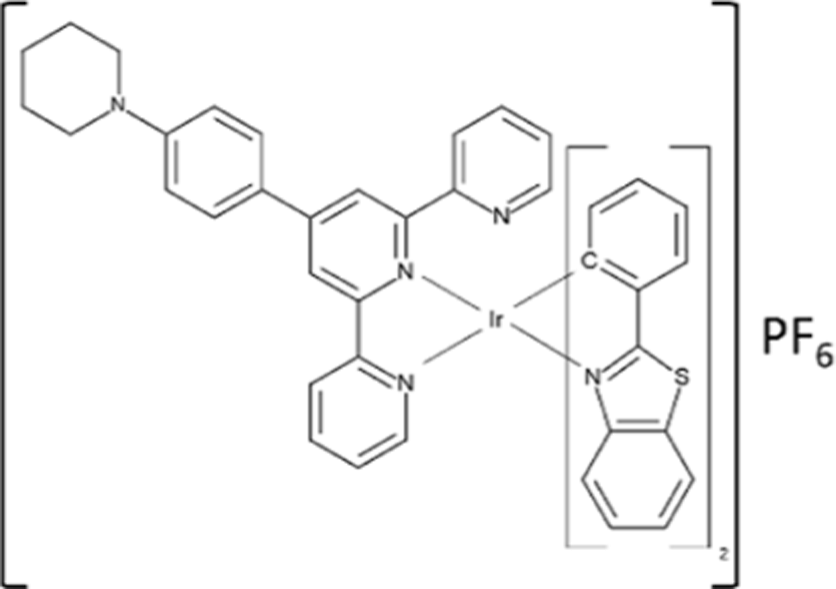


#### Ir­(Ph-bztz)_2_(ppr-C_6_H_4_-terpy-κ^2^N)]­PF_6_ (**3**)

Yield: 0.11 g, 65%. Anal. Calcd for C_52_H_40_F_6_IrN_6_PS_2_·0.5CH_3_CN (1170.7 g/mol): C, 54.37; H, 3.57; N, 7.78%. Found: C, 54.40; H, 3.52; N, 7.90%. ^1^H NMR (500 MHz, DMSO-*d*_6_): δ 9.14 (d, *J* = 8.3 Hz, 1H), 9.02 (d, *J* = 2.1 Hz, 1H), 8.30–8.23 (m, 2H), 8.22 (dd, *J* = 8.3, 1.2 Hz, 1H), 8.06–8.01 (m, 2H), 7.99 (dt, *J* = 4.9, 1.4 Hz, 1H), 7.85–7.80 (m, 2H), 7.71 (dd, *J* = 5.7, 1.6 Hz, 1H), 7.64 (ddd, *J* = 7.0, 5.5, 1.2 Hz, 1H), 7.47 (ddd, *J* = 8.2, 7.2, 1.1 Hz, 1H), 7.42 (ddd, *J* = 8.2, 7.2, 1.1 Hz, 1H), 7.38–7.28 (m, 2H), 7.22–7.12 (m, 2H), 7.09–6.98 (m, 4H), 6.91 (ddd, *J* = 7.7, 4.8, 1.2 Hz, 1H), 6.84 (td, *J* = 7.5, 1.4 Hz, 2H), 6.62 (td, *J* = 7.5, 1.1 Hz, 1H), 6.47 (td, *J* = 7.4, 1.4 Hz, 1H), 6.25 (d, *J* = 8.4 Hz, 1H), 5.93 (d, *J* = 7.6 Hz, 1H), 5.86 (d, *J* = 7.6 Hz, 1H), 3.37 (br s, 2H), 1.60 (br s, 4H) ppm. ^13^C­{H} NMR (126 MHz, DMSO-*d*_6_): δ 181.70, 180.34, 163.33, 157.43, 157.41, 154.19, 152.92, 151.66, 150.02, 149.58, 149.24, 148.43, 148.39, 147.63, 139.99, 139.48, 139.33, 135.53, 132.34, 132.21, 131.11, 130.61, 130.38, 129.01, 128.90, 128.20, 128.07, 127.94, 127.80, 126.72, 126.40, 126.19, 125.70, 125.66, 125.50, 124.56, 124.42, 124.06, 123.77, 123.29, 123.22, 121.52, 120.89, 119.58, 118.20, 116.92, 114.35, 47.97, 24.91, 23.94 ppm. IR (KBr, cm^–1^) intensity: 3447 (w), 3055 (w), 2935 (w), 1585 (s), 1525 (w), 1472 (w), 1448 (m), 1438 (m), 1407 (m), 1364 (w), 1322 (w), 1298 (w), 1265 (w), 1239 (m), 1216 (w), 1126 (w), 1070 (w), 1025 (w), 995 (w), 918 (w), 840 (s), 789 (w), 755 (m), 725 (w), 616 (w), 557 (m), 447 (w).
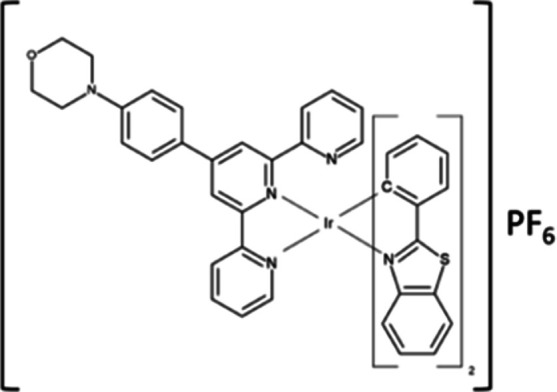


#### Ir­(Ph-bztz)_2_(morph-C_6_H_4_-terpy-κ^2^N)]­PF_6_ (**4**)

Yield: 0.13 g, 76%. Anal. Calcd for C_51_H_38_F_6_IrN_6_OPS_2_·0.5CH_3_CN (1172.72 g/mol): C, 53.26; H, 3.39; N, 7.76%. Found: C, 53.12; H, 3.29; N, 7.96%. ^1^H NMR (500 MHz, DMSO-*d*_6_): δ 9.15 (d, *J* = 8.5 Hz, 1H), 9.05 (d, *J* = 2.1 Hz, 1H), 8.30–8.24 (m, 2H), 8.22 (d, *J* = 7.5 Hz, 1H), 8.11–8.06 (m, 2H), 7.99 (dt, *J* = 4.6, 1.5 Hz, 1H), 7.87 (d, *J* = 2.0 Hz, 1H), 7.83 (dd, *J* = 7.6, 1.4 Hz, 1H), 7.73–7.70 (m, 1H), 7.65 (ddd, *J* = 7.0, 5.6, 1.2 Hz, 1H), 7.47 (ddd, *J* = 8.2, 7.2, 1.1 Hz, 1H), 7.42 (ddd, *J* = 8.2, 7.2, 1.1 Hz, 1H), 7.35 (dd, *J* = 7.6, 1.3 Hz, 1H), 7.31 (ddd, *J* = 8.5, 7.2, 1.3 Hz, 1H), 7.19 (td, *J* = 7.7, 1.8 Hz, 1H), 7.16 (ddd, *J* = 8.5, 7.3, 1.3 Hz, 1H), 7.10–7.06 (m, 2H), 7.06–7.03 (m, 1H), 7.02 (td, *J* = 7.5, 1.1 Hz, 1H), 6.91 (ddd, *J* = 7.7, 4.8, 1.1 Hz, 1H), 6.87–6.82 (m, 2H), 6.62 (td, *J* = 7.4, 1.1 Hz, 1H), 6.48 (td, *J* = 7.5, 1.4 Hz, 1H), 6.25 (dt, *J* = 8.5, 0.8 Hz, 1H), 5.93 (d, *J* = 7.6 Hz, 1H), 5.86 (d, *J* = 7.6 Hz, 1H), 3.78–3.72 (m, 4H), 3.31–3.28 (m, 4H) ppm. ^13^C­{H} NMR (126 MHz, DMSO-*d*_6_): δ 181.72, 180.35, 163.42, 157.51, 157.38, 154.13, 153.00, 151.62, 150.00, 149.62, 149.23, 148.44, 148.39, 147.55, 140.02, 139.48, 139.34, 135.57, 132.36, 132.23, 131.12, 130.61, 130.41, 129.26, 128.94, 128.12, 128.01, 127.95, 127.87, 127.82, 126.74, 126.43, 126.21, 125.78, 125.68, 125.57, 124.58, 124.44, 124.39, 123.82, 123.33, 122.94, 120.92, 119.83, 118.19, 116.93, 114.30, 65.89, 47.04 ppm. IR (KBr, cm^–1^) intensity: 3447 (w), 3055 (w), 2955 (w), 2845 (w), 1599 (m), 1587 (m), 1551 (w), 1523 (m), 1467 (w), 1447 (m), 1438 (m), 1404 (m), 1383 (w), 1346 (w), 1321 (w), 1298 (w), 1265 (w), 1232 (m), 1218 (m), 1162 (w), 1125 (w), 1134 (w), 1070 (w), 1052 (w), 1026 (w), 995 (w), 929 (m), 904 (w), 840 (s), 788 (w), 756 (m), 737 (w), 724 (w), 637 (w), 557 (m), 446 (w).
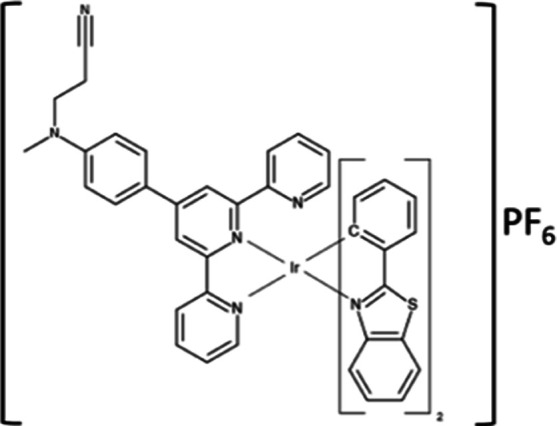


#### Ir­(Ph-bztz)_2_((CH_3_)­(CH_2_CH_2_CCN))_2_N-C_6_H_4_-terpy-κ^2^N)]­PF_6_ (**5**)

Yield: 0.08 g, 47%. Anal. Calcd for C_51_H_37_F_6_IrN_7_PS_2_·H_2_O (1167.21 g/mol): C, 52.48; H, 3.37; N, 8.40%. Found: C, 52.40; H, 3.16; N, 8.42%. ^1^H NMR (500 MHz, DMSO-*d*_6_): δ 9.15 (d, *J* = 8.5 Hz, 1H), 9.02 (d, *J* = 2.1 Hz, 1H), 8.30–8.24 (m, 2H), 8.22 (dd, *J* = 8.3, 1.2 Hz, 1H), 8.09–8.04 (m, 2H), 7.99 (dt, *J* = 4.9, 1.4 Hz, 1H), 7.84 (d, *J = 2.0 Hz, 1H), 7.82 (dd, *J* = 7.6, 1.4 Hz, 1H), 7.71 (dd, *J* = 5.7, 1.7 Hz, 1H), 7.64 (ddd, *J* = 7.1, 5.5, 1.2 Hz, 1H), 7.47 (ddd, *J* = 8.2, 7.2, 1.1 Hz, 1H), 7.42 (ddd, *J* = 8.2, 7.2, 1.1 Hz, 1H), 7.35 (dd, *J* = 7.7, 1.3 Hz, 1H), 7.31 (ddd, *J* = 8.5, 7.2, 1.2 Hz, 1H), 7.22–7.13 (m, 2H), 7.06 (d, *J* = 8.4 Hz, 1H), 7.01 (td, *J* = 7.5, 1.1 Hz, 1H), 6.94–6.89 (m, 3H), 6.84 (td, *J* = 7.5, 1.5 Hz, 2H), 6.62 (td, *J* = 7.5, 1.1 Hz, 1H), 6.47 (td, *J* = 7.5, 1.4 Hz, 1H), 6.25 (d, *J* = 8.3 Hz, 1H), 5.93 (d, *J* = 7.8 Hz, 1H), 5.86 (d, *J* = 7.7 Hz, 1H), 3.81 (t, *J* = 6.6 Hz, 2H), 3.06 (s, 3H), 2.77 (t, *J* = 6.6 Hz, 2H) ppm. ^13^C­{H} NMR (126 MHz, DMSO-*d*_6_): δ 181.69, 180.35, 163.33, 157.44, 157.41, 154.20, 151.66, 150.53, 150.12, 149.59, 149.25, 148.43, 148.40, 147.66, 140.00, 139.49, 139.34, 135.56, 132.35, 132.22, 131.12, 130.62, 130.39, 129.11, 128.06, 127.94, 127.81, 126.72, 126.42, 126.20, 125.67, 125.51, 124.56, 124.43, 123.95, 123.79, 123.30, 123.22, 120.89, 120.86, 119.49, 118.19, 116.94, 112.25, 47.14, 37.88, 15.09 ppm. IR (KBr, cm^–1^) intensity: 3442 (w), 3053 (w), 2250 (w), 1600 (m), 1585 (m), 1567 (w), 1551 (w), 1525 (m), 1473 (m), 1448 (m), 1437 (m), 1407 (m), 1368 (w), 1348 (w), 1323 (w), 1298 (w), 1266 (m), 1198 (m), 1158 (w), 1120 (w), 1071 (w), 1027 (w), 991 (w), 964 (w), 841 (s), 813 (m), 789 (m), 754 (m), 725 (m), 681 (w), 660 (w), 557 (m), 444 (w).
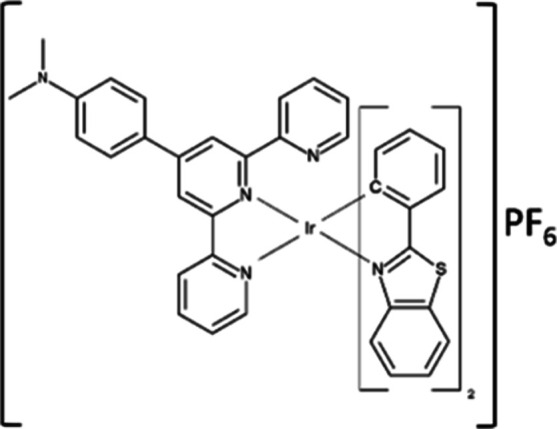


#### Ir­(Ph-bztz)_2_((CH_3_)_2_N-C_6_H_4_-terpy-κ^2^N)]­PF_6_ (**6**)

Yield: 0.12 g, 70%. Anal. Calcd for C_49_H_36_F_6_IrN_6_PS_2_·CH_3_OH (1142.20 g/mol): C, 52.58; H, 3.53; N, 7.36%. Found: C, 52.92; H, 3.16; N, 7.76%. ^1^H NMR (500 MHz, DMSO-*d*_6_): δ 9.14 (d, *J* = 8.3 Hz, 1H), 9.00 (d, *J* = 2.1 Hz, 1H), 8.30–8.24 (m, 2H), 8.22 (dd, *J* = 8.2, 1.4 Hz, 1H), 8.08–8.03 (m, 2H), 7.99 (dt, *J* = 4.6, 1.5 Hz, 1H), 7.84–7.79 (m, 2H), 7.73–7.69 (m, 1H), 7.64 (ddd, *J* = 7.0, 5.6, 1.2 Hz, 1H), 7.47 (ddd, *J* = 8.2, 7.2, 1.1 Hz, 1H), 7.42 (ddd, *J* = 8.3, 7.2, 1.1 Hz, 1H), 7.35 (dd, *J* = 7.8, 1.4 Hz, 1H), 7.31 (ddd, *J* = 8.5, 7.2, 1.3 Hz, 1H), 7.21–7.13 (m, 2H), 7.08 (d, *J* = 8.4 Hz, 1H), 7.01 (td, *J* = 7.5, 1.1 Hz, 1H), 6.91 (ddd, *J* = 7.7, 4.9, 1.2 Hz, 1H), 6.87–6.78 (m, 4H), 6.62 (td, *J* = 7.5, 1.1 Hz, 1H), 6.47 (td, *J* = 7.4, 1.4 Hz, 1H), 6.25 (d, *J* = 8.4 Hz, 1H), 5.93 (d, *J* = 7.4 Hz, 1H), 5.86 (d, *J* = 7.0 Hz, 1H), 3.04 (s, 6H) ppm. ^13^C­{H} NMR (126 MHz, DMSO-*d*_6_): δ 181.69, 180.34, 163.25, 157.48, 157.34, 154.22, 152.26, 151.69, 150.20, 149.58, 149.26, 149.18, 148.41, 147.71, 139.98, 139.50, 139.33, 135.56, 132.35, 132.22, 131.12, 130.62, 130.38, 128.92, 128.03, 127.81, 126.72, 126.42, 126.20, 125.67, 125.46, 124.56, 124.43, 123.77, 123.29, 120.88, 119.94, 119.30, 116.93, 111.97, 40.11 ppm. IR (KBr, cm^–1^) intensity: 3421 (w), 3053 (w), 1597 (s), 15,586 (s), 1533 (m), 1472 (m), 1448 (m), 1438 (m), 1407 (m), 1367 (m), 1322 (w), 1299 (w), 1266 (m), 1209 (m), 1170 (w), 1125 (w), 1069 (w), 1026 (w), 994 (w), 945 (w), 840 (s), 792 (m), 754 (m), 739 (w), 723 (w), 683 (w), 557 (m), 445 (w).
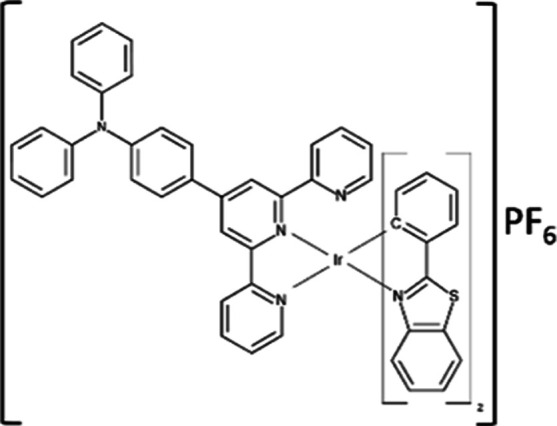


#### Ir­(Ph-bztz)_2_((Ph)_2_N-C_6_H_4_-terpy-κ^2^N)]­PF_6_ (**7**)

Yield: 0.13 g, 68%. Anal. Calcd for C_59_H_40_F_6_IrN_6_PS_2_·H_2_O (1252.31 g/mol): C, 56.59; H, 3.38; N, 6.71%. Found: C, 56.45; H, 3.27; N, 6.92%. ^1^H NMR (500 MHz, DMSO-*d*_6_): δ 9.12 (d, *J* = 8.4 Hz, 1H), 9.06 (d, *J* = 2.0 Hz, 1H), 8.30–8.25 (m, 2H), 8.23 (dt, *J* = 8.1, 1.0 Hz, 1H), 8.06–8.02 (m, 2H), 7.99 (dt, *J* = 4.7, 1.6 Hz, 1H), 7.85 (d, *J* = 2.0 Hz, 1H), 7.83 (dd, *J* = 7.5, 1.3 Hz, 1H), 7.74–7.70 (m, 1H), 7.65 (ddd, *J* = 6.9, 5.6, 1.2 Hz, 1H), 7.48 (ddd, *J* = 8.3, 7.2, 1.1 Hz, 1H), 7.42 (ddd, *J* = 8.1, 7.2, 1.0 Hz, 1H), 7.41–7.31 (m, 6H), 7.21–7.11 (m, 8H), 7.05–6.97 (m, 4H), 6.91 (ddd, *J* = 7.7, 4.9, 1.2 Hz, 1H), 6.84 (td, *J* = 7.6, 1.4 Hz, 2H), 6.62 (td, *J* = 7.5, 1.1 Hz, 1H), 6.48 (td, *J* = 7.4, 1.4 Hz, 1H), 6.26–6.22 (m, 1H), 5.93 (d, *J* = 7.7 Hz, 1H), 5.86 (d, *J* = 7.7 Hz, 1H) ppm. ^13^C­{H} NMR (126 MHz, DMSO-*d*_6_): δ 181.75, 180.33, 163.49, 157.61, 157.25, 154.01, 151.57, 150.12, 149.82, 149.64, 149.20, 148.49, 148.37, 147.38, 146.13, 140.08, 139.45, 139.33, 135.58, 132.34, 132.24, 131.12, 130.60, 130.42, 129.88, 129.14, 128.21, 128.02, 127.84, 126.75, 126.42, 126.23, 126.15, 125.70, 125.45, 125.11, 124.65, 124.59, 124.47, 123.87, 123.36, 120.95, 120.67, 120.50, 116.88 ppm. IR (KBr, cm^–1^) intensity: 3421 (w), 3056 (w), 1604 (m), 1587 (s), 1552 (w), 1415 (m), 1487 (m), 1448 (m), 1439 (w), 1409 (m), 1369 (w), 1331 (m), 1298 (m), 1266 (m), 1201 (m), 1182 (w), 1162 (w), 1126 (w), 1073 (w), 1051 (w), 1027 (w), 993 (w), 835 (s), 788 (m), 754 (m), 729 (w), 699 (w), 384 (w), 658 (w), 558 (m), 520 (w), 447 (w).

### Experimental Methods

Elemental analyses (C, H, N) were carried out on an Elementar Vario EL Cube analyzer. NMR spectra were recorded on a Bruker Avance 500 MHz spectrometer in DMSO-*d*_6_. IR spectra were acquired using a Nicolet iS5 FTIR spectrophotometer (4000–400 cm^–1^) with the KBr pellet method. X-ray diffraction data were collected at room temperature on a Gemini A Ultra diffractometer (Oxford Diffraction) with Mo Kα radiation (λ = 0.71073 Å); crystallographic data for complexes **2** and **6** were deposited in the Cambridge Crystallographic Data Centre (CCDC 2532314 and 2532313).

The GAUSSIAN-16 software package was used to perform theoretical calculations at both the DFT and TD-DFT levels by applying the PBE0 functional. For iridium, the Stuttgart/Dresden relativistic small-core ECP basis set was employed, and for all other elements, the def2-TZVP basis set was used with solvent effects included via the polarizable continuum model (PCM).

UV–vis absorption spectra were recorded on an Evolution 220 UV–vis spectrometer (ThermoScientific). Emission and excitation spectra, along with time-resolved measurements (TCSPC), were recorded using an FLS-980 fluorescence spectrophotometer (Edinburgh Instruments) in various media, including different solvents at room temperature, solid-state samples, and frozen matrices at 77 K (4:1 v/v).

Femtosecond transient absorption (fs-TA) spectra were recorded using a pump–probe system (Ultrafast Systems, Helios) with samples prepared in argon-saturated acetonitrile at concentrations giving absorbance ∼0.5. Measurements were done at 355 nm excitation for solutions in stirred quartz cells. Further detailed experimental procedures are provided in Electronic Supporting Information (ESI).

Electroluminescence (EL) spectra were acquired by mounting the sample on an XYZ stage and applying a constant voltage using a Gw Instek PSP-405 precision power supply. The light emitted from the OLED device was collected via a 30 mm lens and focused onto the 50 μm entrance slit of a Shamrock SR-303i monochromator. Detection was performed using an Andor iDus 12305 CCD detector with a typical integration time of 10 s. Optical alignment of the system was pre-established using a 405 nm laser.

## Supplementary Material



## Notes

Additional experimental details, IR and NMR data, X-ray analysis, UV–vis data, emission spectra, lifetime decay curves, electrochemistry data, nano- and femtosecond transient absorption analysis, and DFT calculations X-ray CIF data deposited at CCDC nos. 2532314 and 2532313 (PDF)
