# Supplementary material for: Controlling Photophysical Properties in Bis-Cyclometalated Ir(III)-Terpyridine Complexes through Photoinduced Intraligand Charge Transfer
Source: Inorg Chem. 2026 Jun 15;65(25):14112–33. doi: 10.1021/acs.inorgchem.6c01548 (PMC13321313; doi:10.1021/acs.inorgchem.6c01548)
Supplement: Supplementary file 1 [file ic6c01548_si_001.pdf]

SUPPORTING INFORMATION

Controlling photophysical properties in bis-cyclometalated Ir(III)–terpyridine complexes through photoinduced intraligand charge transfer

Joanna Palion-Gazda[1*], Aleksandra Kwiecień[1], Mateusz Penkala[1], Anna Kryczka[1], Barbara Machura[1*], Patrycja Rawicka[2] Mariola Siwy[3], Dorota Kowalska[4], Sebastian Maćkowski[4], Ewa Schab-Balcerzak[1,3], Karol Erfurt[5]

[1]*Institute of Chemistry, University of Silesia, 9 Szkolna Str., 40-006 Katowice, Poland, e-mail:* joanna.palion-gazda@us.edu.pl, barbara.machura@us.edu.pl
[2]*Institute of Physics, Faculty of Science and Technology, University of Silesia, 75 Pułku Piechoty 1a, 41-500 Chorzów, Poland*
[3]*Centre of Polymer and Carbon Materials, Polish Academy of Sciences.*
[4]*Institute of Physics, Faculty of Physics, Astronomy and Informatics, Nicolaus Copernicus University in Toruń, ul. Grudziądzka 5, 87-100 Toruń, Poland*
[5]*Department of Chemical Organic Technology and Petrochemistry, Silesian University of Technology, Krzywoustego 4, 44-100 Gliwice, Poland*

Table of contents

Experimental Section **3**
Crystal structure determination and refinement 3
Computational details 3
Physical measurements 3
Photoluminescence spectra 4
Device Preparation 4
Electroluminescence spectra and kinetics 4
Femtosecond transient absorption spectroscopy 4
HR-MS spectrometry **5**
Figure S1. HR-ESI-MS spectra of **1–7** (a–g). 16
NMR spectroscopy **16**
Figure S2. NMR spectra of **1**–**7** (a–g). 23
FT-IR spectroscopy **24**
Figure S3. FT-IR spectra of Ph-btz (a) and **1**–**7** along with those for the free ligands (b–h). 27
X-Ray analysis **27**
Table S1. Crystal data and structure refinement of complexes **2** and **6**. 27
Table S2. Experimental bond lengths [Å] and angles [°] for **2** and **6**. 28
Table S3. Short intra- and intermolecular contacts in the crystal structures of **2** and **6**. 29
Table S4. Short π•••π interactions (with Cg•••Cg distances shorter than 4 Å) in the crystal structures of **2** and **6**. 29
Table S5. X–H•••Cg(J)(π-ring) interactionsin the crystal structures of **2** and **6**. 29
Figure S4. Crystal packing arrangement of **2** (a) and **6** (b) along with the selected intermolecular contacts. 31
Electrochemical charcterization **32**
Figure S5. The cyclic voltammetry (CV) and differential pulse voltammetry (DPV) curves of complexes **1**–**7**. 32

UV-Vis spectroscopy **32**

Table S7. The absorption maxima and molar extinction coefficient values for complexes **1**–**7**. 32

Figure S6. UV-Vis spectra of **1**–**7** in chloroform solution (a) solid state as film on glass substrate (b). 33

Figure S7. UV-Vis spectra of **1–7** in CH_3CN alongside electronic absorption spectra of 2-phenylbenzothiazole and the appropriate terpy derivative. 34

Figure S8. Solvatochromic impact on the absorbance behavior of **1**–**7**. 35

Figure S9. UV-Vis photostability of Ir(III) complexes upon 420 nm light irradiation in CH_3CN and $CHCl_3$; $c = 10^{-5}$ M.38

Luminescence properties **39**

Figure S10. Summary of photoluminescence properties of complex **1**. 40

Figure S11. Summary of photoluminescence properties of complex **2**. 41

Figure S12. Summary of photoluminescence properties of complex **3**. 42

Figure S13. Summary of photoluminescence properties of complex **4**. 44

Figure S14. Summary of photoluminescence properties of complex **5**. 45

Figure S15. Summary of photoluminescence properties of complex **6**. 46

Figure S16. Summary of photoluminescence properties of complex **7**. 49

Figure S17. Emission spectra and decay curves of **1–7** in $CHCl_3$ and CH_3CN. 53

Figure S18. The phosphorescence spectra of $[Ir(Ph\text{-}btz)_2(Ph\text{-}terpy)](PF_6)$ (**2**) compared to those of $[Ir(Py\text{-}py)_2(Ph\text{-}terpy)](PF_6)$ 53

Figure S19. Normalized emission spectra of **3**, **5** and **7** in the matrix at 77 K and solution at room temperature. 54

Figure S20. Linear correlation of λ^{em} of **2–6** with the calculated σ_p parameter of the R–group. 54

Figure S21. Comparative analysis of photoluminescent data of **4** with those for its analogue $[Ir(Ph\text{-}py)_2(morph\text{-}C_6H_4\text{-}terpy\text{-}\kappa^2N)]PF_6$. 55

Figure S22. Emission spectra of **1**–**7** upon different excitation wavelengths. 57

Figure S23. Emission spectra of $N(Ph)_2\text{-}C_6H_5$-terpy and complex 7 in CH_3CN. 58

Figure S24. UPLC spectra for **7**. 58

Figure S25. Emission spectra of **1–7** in the solid state as thin films deposited on a glass substrate. 58

Figure S26. Photos of exemplary diodes **3**, **5** and **6** (a), the EL intensity recorded as a function of time and EL spectra of ITO/PEDOT:PSS/2/Al (b), effect of applied voltage on EL intensity of diode ITO/PEDOT:PSS/1/Al and ITO/PEDOT:PSS/5/Al (c) 59

Table S8. EL data of the fabricated diodes ITO/PEDOT:PSS/ complex/Al and ITO/PEDOT:PSS/PVK:PBD:complex/Al obtained under external voltage 11 V. 59

Transient absorption spectroscopy **60**

Figure S27. Summary of the global lifetime analysis of **1-7** (acetonitrile, pump wavelength 355 nm; pump power 0.24 μJ per pulse) containing, evolution associated spectra, residual map, time traces at several wavelength and transient spectra. 63

Figure S28. Nanosecond transient absorption (ns-TA) spectra obtained using laser flash photolysis (LFP) in degassed CH_3CN, together with the corresponding decay traces. 65

Figure S29. Comparison of the TA spectral profiles of the complexes **3–5** recorded at 6 ns delay time in the fs-TA experiments with their earliest delay-time spectra obtained by laser flash photolysis. 66

Figure S30. UV–Vis absorption spectra of complexes **1**–**7** recorded before and after laser irradiation. 67

Table S9. Triplet state maximum absorption (λ_{max}) and average lifetimes deaerated ($\tau T, N2$) environments obtained for all tested compounds dissolved in acetonitrile 67

Theoretical calculations **67**

Figure S31. Frontier molecular orbitals of and major electron density distributions of complexes **1–7** (a–g, respectively). 75

Table S10. Assignment of calculated singlet excited states to the UV–Vis spectra and TD-DFT parameters of electronic transitions for **1**........ 76

Table S11. Assignment of calculated singlet excited states to the UV–Vis spectra and TD-DFT parameters of electronic transitions for **2**........ 77

Table S12. Assignment of calculated singlet excited states to the UV–Vis spectra and TD-DFT parameters of electronic transitions for **3**........ 78

Table S13. Assignment of calculated singlet excited states to the UV–Vis spectra and TD-DFT parameters of electronic transitions for **4**........ 79

Table S14. Assignment of calculated singlet excited states to the UV–Vis spectra and TD-DFT parameters of electronic transitions for **5**........ 80

Table S15. Assignment of calculated singlet excited states to the UV–Vis spectra and TD-DFT parameters of electronic transitions for **6**........ 81

Table S16. Assignment of calculated singlet excited states to the UV–Vis spectra and TD-DFT parameters of electronic transitions for **7**........ 82

Figure S32. The isodensity surface plots of the LSOMO and HSOMO of **1**–**7**........ 84

References........ 85

Experimental Section

Crystal structure determination and refinement

The X-ray diffraction data for compounds **2** and **6** were obtained using Oxford Diffraction Gemini A Ultra four-circle diffractometer equipped with an Atlas CCD detector and graphite monochromated MoKα radiation (λ = 0.71073 Å). The data collection, unit cell refinement, and data reduction were carried out using the CrysAlis^{Pro} software (Agilent Technologies Ltd., 2014). The crystal structures of Ir(III) complexes were solved by direct methods using the SHELXS program and refined via least-squares minimization the SHELXL-2014 package[1]. Non-hydrogen atoms were refined anisotropically. Hydrogen atoms in each structure were positioned geometrically and refined using a riding model with fixed bond lengths: d(C–H) = 0.93 Å and thermal parameters: U_{iso}(H) = 1.2 U_{eq}(C). Detailed crystallographic data, structure determination procedures, and refinement parameters are summarized in Table S1.

Computational details

Theoretical calculations for all investigated compounds were performed based on fully optimized geometries using the GAUSSIAN 16 software package[2]. Calculations were carried out at both the DFT and TD-DFT levels, employing the PBE0 hybrid functional[3,4], which is well-regarded for its balanced accuracy in predicting ground- and excited-state properties. For the iridium atom, the Stuttgart/Dresden relativistic ECP[5,6] along with its corresponding basis set was used to incorporate scalar relativistic effects and reduce computational cost. For all other atoms, the def2-TZVP basis set[7–9] was applied to ensure accurate treatment of valence electron interactions. Solvent effects were accounted for using the polarizable continuum model (PCM)[10,11], which simulates the influence of the solvent as a continuous polarizable medium, thereby providing more realistic modelling conditions. The predicted structural parameters in the ground state fall within the expected error range associated with DFT calculations, indicating the reliability of the applied computational methods. All calculations were performed using high-performance computing resources provided by the Wroclaw Centre for Networking and Supercomputing (http://wcss.pl), ensuring the efficiency and scalability required for the geometry optimizations and property evaluations.

Physical measurements

NMR spectra (^{1}H, ^{13}C) were acquired on a Bruker Avance 500 MHz spectrometer at 298 K, with DMSO-d_6 used as the solvent. The operating frequencies were 500 MHz for 1H and 125 MHz for

13C. Chemical shifts were calibrated using the residual solvent peak. HRMS analyses were performed with use of Xevo G2 Q-TOF mass spectrometer (Waters) with an ESI ion source in positive and negative ion modes. The collection of data was performed from 100 to 1000 Da, scan time 0.5 s, in centroid mode, with the mass corrected using external reference (Lock-SprayTM leucine enkephalin solution, reference ion [M+H]+ at m/z 556.2771 Da). Data analysis was performed with the MassLynx software (Waters) incorporated with the instrument.
Fourier-transform infrared (FTIR) spectra were recorded in the 4000–400 cm^{-1} range using a Nicolet iS5 spectrometer, with samples prepared as potassium bromide (KBr) pellets. UV–Vis absorption spectra were measured in $CHCl_3$, and MeCN solutions ($5\cdot10^{-5}$ mol/dm^3). To evaluate kinetic stability, UV–Vis spectra of the compounds in each solvent were recorded at two-hour intervals over a total period of 12 hours. Photostability was examined by recording successive UV–Vis spectra following 20-minute irradiations of the samples using a xenon lamp at a low-energy excitation wavelength.

Photoluminescence spectra

Steady-state emission spectra were recorded for argon-saturated and air-equilibrated solutions in $CHCl_3$ and MeCN (concentration = 5×10^{-5} mol/dm^3) using an FLS-980 fluorescence spectrophotometer (Edinburgh Instruments). This instrument is equipped with a 450 W xenon lamp and a high-gain photomultiplier detector (PMT +500 nm, Hamamatsu R928P). To determine photoluminescence (PL) lifetimes in solution and solid state, two techniques were used: time-correlated single-photon counting (TCSPC) and multi-channel scaling (MCS). TCSPC measurements were conducted on optically diluted solutions using picosecond-pulsed diode lasers (EPLED 375 nm, EPLED 405 nm, and EPLED 470) as excitation sources, with detection performed using a PMT (Hamamatsu R928P, Japan). Decay curves were primarily analyzed using tail fitting and fitted with single- or double-exponential functions. For samples in a frozen matrix at 77 K, PL lifetime measurements were performed using the MCS technique, where excitation was provided by a 60 W microsecond xenon flash lamp. Quantum yields of emission were determined via the absolute method, utilizing an integrating sphere. For argon-saturated solutions, the corresponding solvent served as the blank, while for powdered samples, a Spectralon® reflectance standard was used. Temperature-dependent emission spectra were measured in BuCN solution (concentration = 5×10^{-5} mol/dm^3) with a liquid nitrogen cryostat (Optistat DN, Oxford Instruments) and a Mercury iTC temperature controller (Oxford Instruments).

Device Preparation

Devices based on the architectures ITO/PEDOT:PSS/complex/Al and ITO/PEDOT:PSS/PVK:PBD:complex/Al were fabricated according to the following procedure. Patterned ITO-coated OSSILA substrates were first coated with a thin PEDOT:PSS layer via spin coating at 5000 rpm for 60 s, followed by thermal treatment at 120 °C for 15 min. Subsequently, either the Ir(III) complex alone or a PVK:PBD:complex blend dissolved in chloroform was deposited by spin coating at 1000 rpm for 60 s. The resulting films were then annealed at 100 °C for 5 min. Finally, a 110 nm thick aluminum electrode was deposited under vacuum conditions.

Electroluminescence spectra and kinetics

Electroluminescence (EL) spectra were acquired by mounting the sample on an XYZ stage and applying a constant voltage using a Gw Instek PSP-405 precision power supply. The light emitted from the OLED device was collected via a 30 mm lens and focused onto the 50 μm entrance slit of a Shamrock SR-303i monochromator. Detection was performed using an Andor iDus 12305 CCD detector with a typical integration time of 10 s. Optical alignment of the system was pre-established using a 405 nm laser. The electroluminescence (EL) kinetics were investigated by continuously recording full spectra at 10 s intervals. These consecutive acquisition cycles were performed without interruption over a total duration of approximately 13 minutes.

Femtosecond transient absorption spectroscopy

Femtosecond transient absorption (TA) spectra were recorded using a pump–probe setup (Helios, Ultrafast Systems), as described in detail in our previous publications.[12–14] All samples were prepared in argon-saturated MeCN solutions, with the absorbance of the first electronic band adjusted

to 0.5 (corresponding to concentrations in the range of 2.5×10^{-4} to 1×10^{-4} mol·dm^{-3}), and introduced into quartz cuvettes (2 mm path length), equipped with magnetic stirring. Excitation was carried out at 355 nm, and the transient absorption was monitored in visible spectral regions.

The raw TA data were processed using Surface Xplorer software (Ultrafast Systems), followed by analysis using Optimus™ software.[15] Routine correction procedures, including probe chirp compensation and subtraction of solvent contributions, were performed prior to kinetic analysis. Coherent artifact analysis was used to estimate the instrument response function (IRF), with the full width at half maximum (FWHM) determined to be approximately 200 fs, and to refine the chirp correction. In the ΔA vs. λ spectra, positive features correspond to excited-state absorption (ESA), while negative signals are attributed to ground-state bleaching (GSB). The dataset was deconvoluted into species-associated spectra (SAS), and decay-associated spectra (DAS) were obtained as a linear combination of the SAS components.

Laser Flash Photolysis

Triplet state lifetimes of the investigated compounds were measured using an LKS 60 laser flash photolysis spectrometer (Applied Photophysics). Excitation was provided by the third harmonic (355 nm) of a nanosecond Nd:YAG laser (Brilliant), delivering pulses with a maximum energy of 100 mJ and a peak power of 20 MW. The probe light source was a 150 W xenon flash lamp (OSRAM XBO CR-OFR). The detection system incorporated a monochromator equipped with a 1/1200 mm diffraction grating, enabling spectral measurements across the 200–1000 nm range. Signal acquisition was performed using a photomultiplier tube. Samples were prepared in acetonitrile with absorbance values of 0.2 at 355 nm and introduced into 1 cm path length quartz cuvettes. Measurements were conducted at room temperature under near-anaerobic conditions. To remove dissolved oxygen, all solutions were purged with nitrogen for 30 minutes prior to measurement. Kinetic decay traces of the triplet states were recorded at the wavelength corresponding to the absorption maximum of the triplet–triplet transition for each compound. All decay curves were fitted using a monoexponential model via LKS Pro-Data software.

HR-MS spectrometry

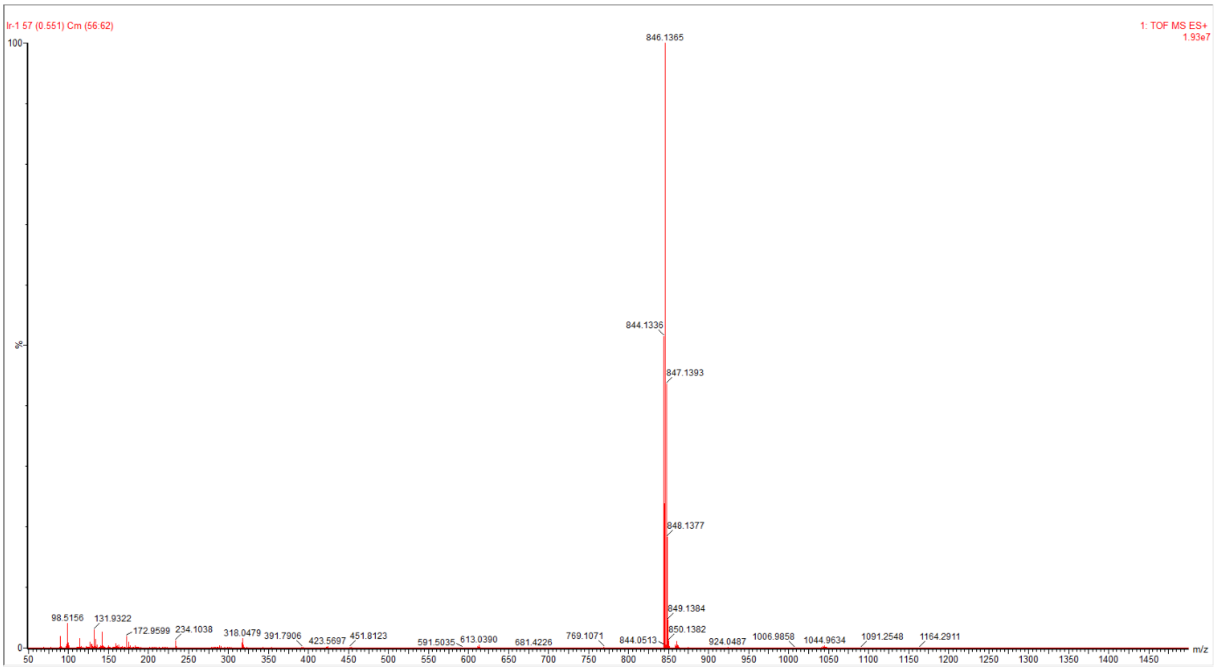

Multiple Mass Analysis: 3 mass(es) processed
Tolerance = 50.0 mDa / DBE: min = -1.5, max = 50.0
Element prediction: Off
Number of isotope peaks used for i-FIT = 3
Monoisotopic Mass, Odd and Even Electron Ions
24 formula(e) evaluated with 3 results within limits (up to 5 best isotopic matches for each mass)
Elements Used:

Mass	RA	Calc. Mass	mDa	PPM	DBE	Formula	i-FIT	i-FIT Norm	Fit Conf %	C	H	N	S	Ir
844.1336	51.46	844.1181	15.5	18.4	33.0	C41 H25 N5 S2 Ir	841.2	n/a	n/a	41	25	5	2	1
846.1365	100.00	846.1337	2.8	3.3	32.0	C41 H27 N5 S2 Ir	833.8	n/a	n/a	41	27	5	2	1
847.1393	43.65	847.1415	-2.2	-2.6	31.5	C41 H28 N5 S2 Ir	808.4	n/a	n/a	41	28	5	2	1

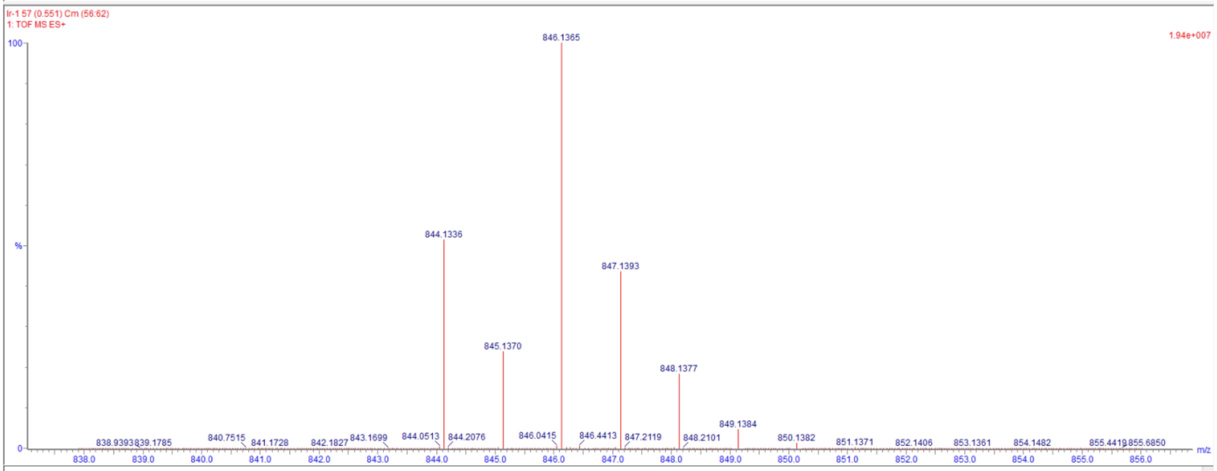

Tolerance = 50.0 mDa / DBE: min = -1.5, max = 50.0
Element prediction: Off
Number of isotope peaks used for i-FIT = 3
Monoisotopic Mass, Even Electron Ions
1 formula(e) evaluated with 1 results within limits (up to 5 best isotopic matches for each mass)
Elements Used:

Mass	RA	Calc. Mass	mDa	PPM	DBE	Formula	i-FIT	i-FIT Norm	Fit Conf %	F	P
144.9657	100.00	144.9642	1.5	10.3	-1.5	F6 P	-1.5	n/a	n/a	6	1

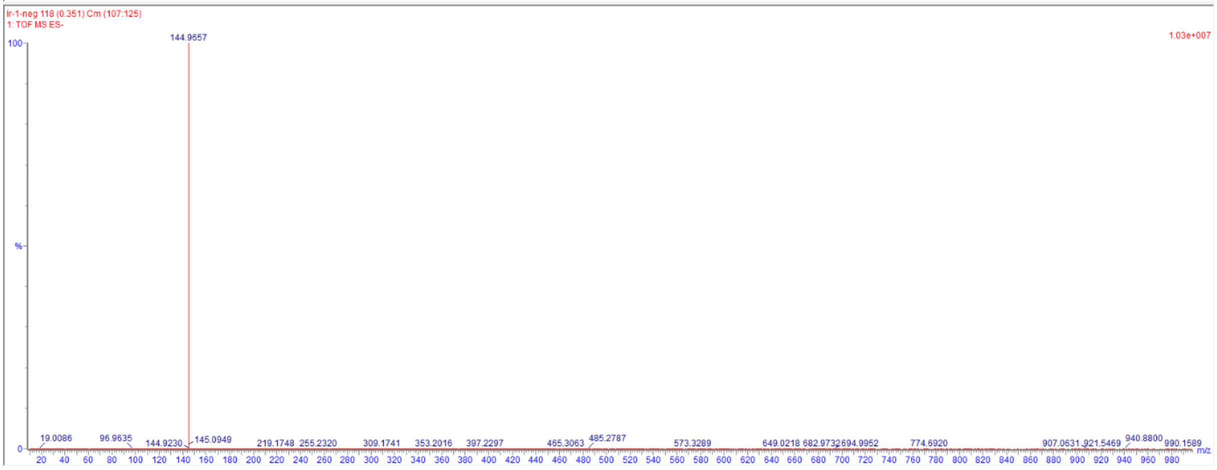

(a) **1** HRMS (ESI): calcd for $C_{41}H_{27}IrN_5S_2^+$ 846.1337 found 846.1365.

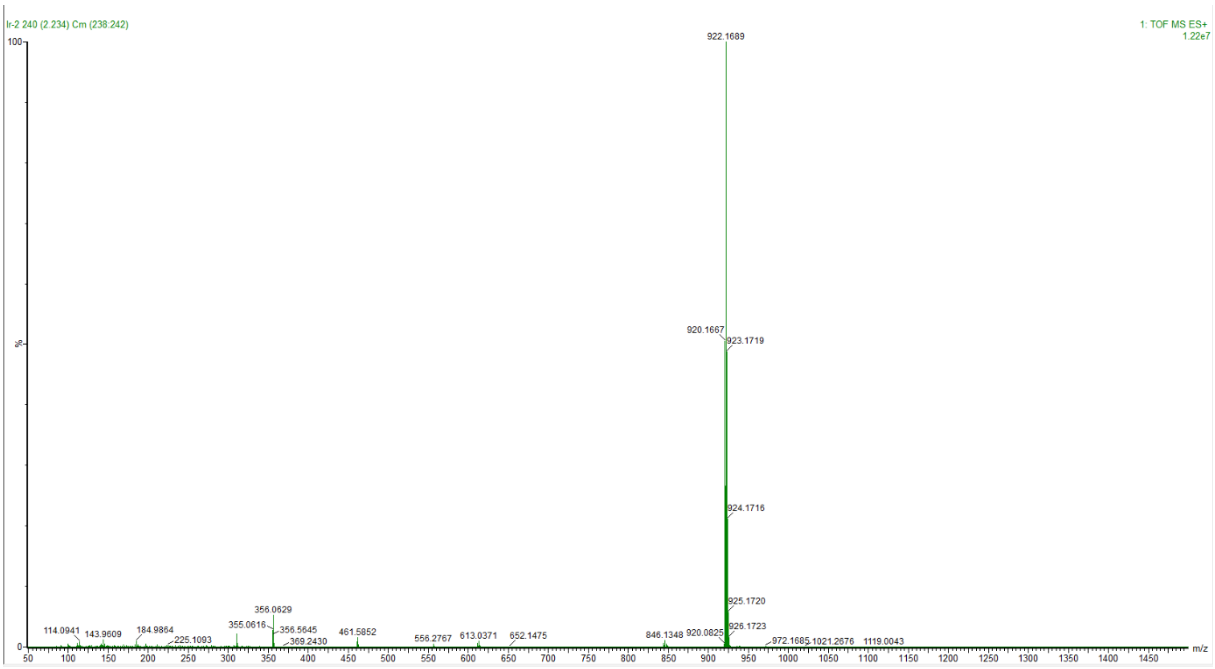
Ir-2 240 (2.234) Cm (238:242)
1: TOF MS ES+
1.22e7
922.1689
920.1667
923.1719
924.1716
925.1720
926.1723
920.0825
846.1348
972.1685
1021.2676
1119.0043
114.0941
143.9609
184.9864
225.1093
355.0616
356.0629
356.5645
369.2430
461.5852
556.2767
613.0371
652.1475
%
m/z

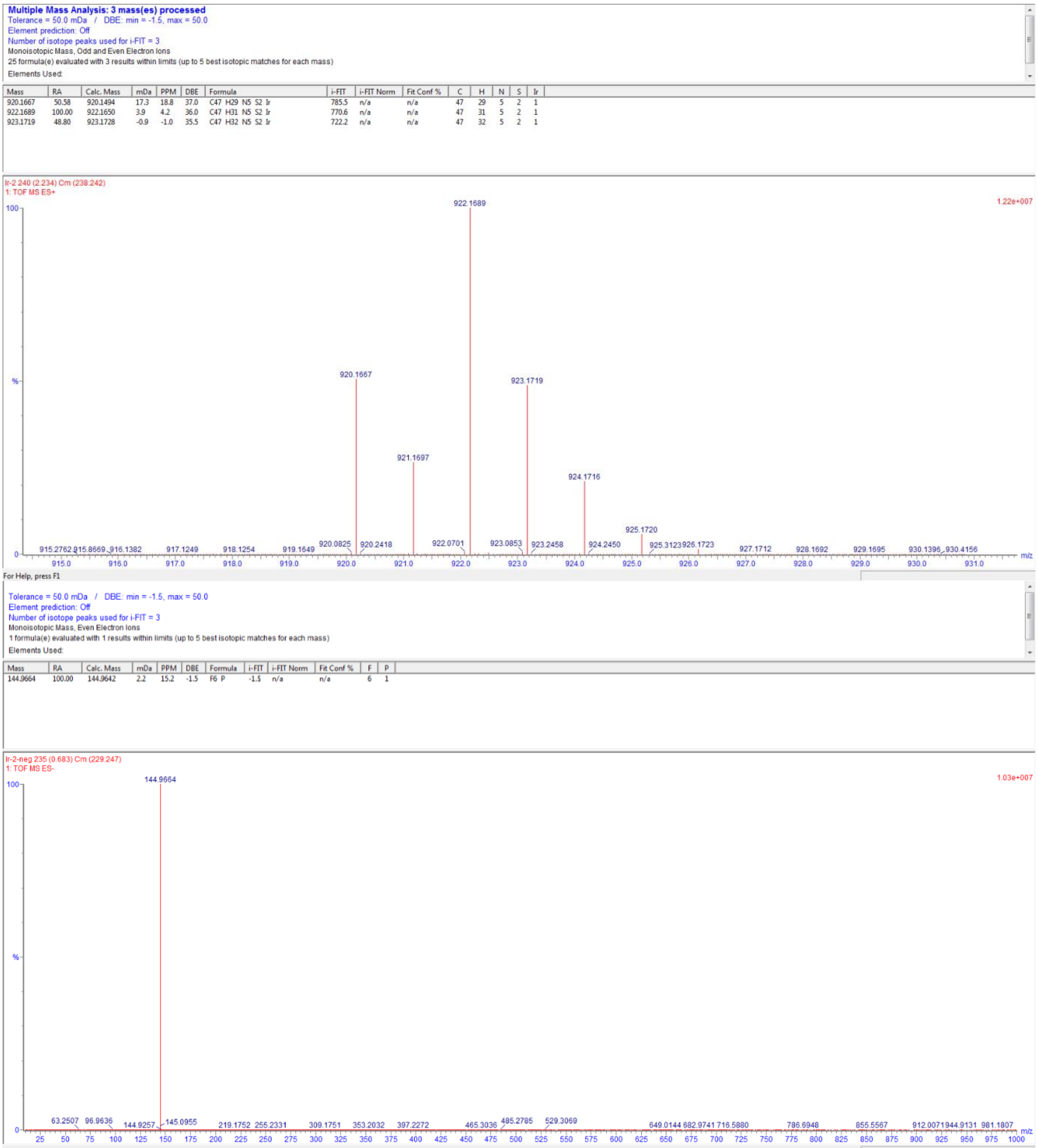

(b) **2** HRMS (ESI): calcd for $C_{47}H_{31}IrN_5S_2^+$ 922.1650 found 922.1689.

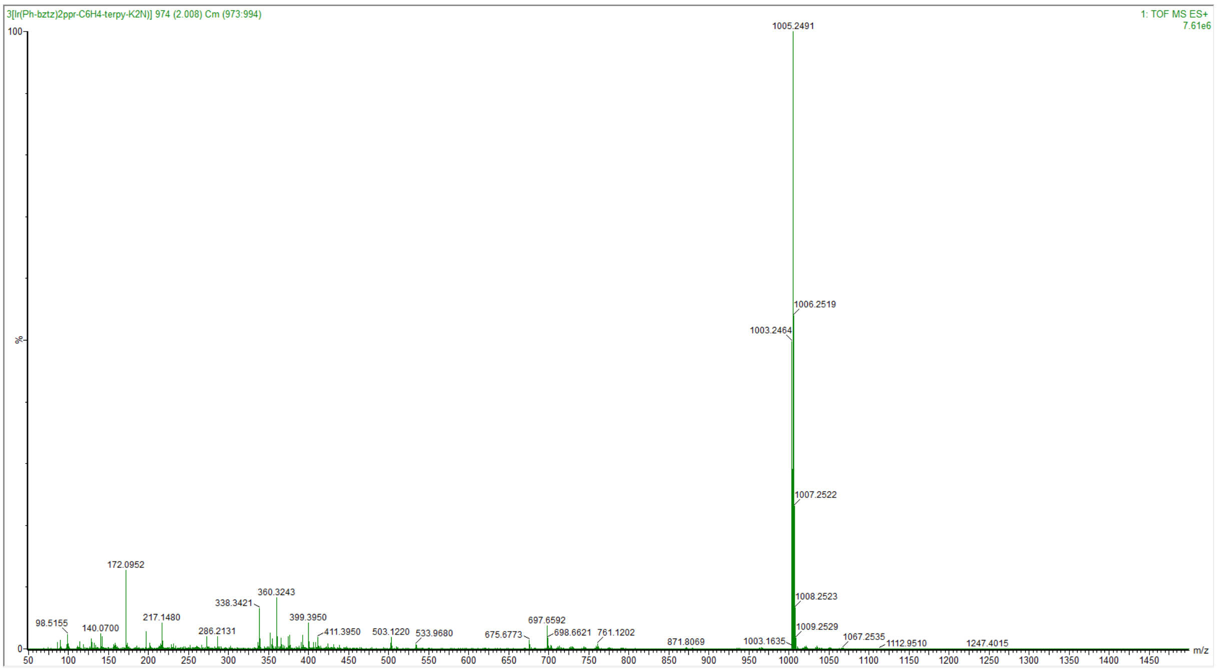

Multiple Mass Analysis: 6 mass(es) processed
Tolerance = 50.0 mDa / DBE: min = -1.5, max = 50.0
Element prediction: Off
Number of isotope peaks used for i-FIT = 3
Monoisotopic Mass, Odd and Even Electron Ions
51 formula(e) evaluated with 6 results within limits (all results (up to 1000) for each mass)
Elements Used:

Mass	RA	Calc. Mass	mDa	PPM	DBE	Formula	i-FIT	i-FIT Norm	Fit Conf %	C	H	N	S	Ir
1003.2464	49.82	1003.2229	23.5	23.4	38.0	C52 H38 N6 S2 Ir	872.9	n/a	n/a	52	38	6	2	1
1004.2494	29.09	1004.2307	18.7	18.6	37.5	C52 H39 N6 S2 Ir	874.5	n/a	n/a	52	39	6	2	1
1005.2491	100.00	1005.2385	10.6	10.5	37.0	C52 H40 N6 S2 Ir	858.2	n/a	n/a	52	40	6	2	1
1006.2519	53.98	1006.2463	5.6	5.6	36.5	C52 H41 N6 S2 Ir	797.7	n/a	n/a	52	41	6	2	1
1007.2522	23.17	1007.2542	-2.0	-2.0	36.0	C52 H42 N6 S2 Ir	734.5	n/a	n/a	52	42	6	2	1
1008.2523	6.64	1008.2620	-9.7	-9.6	35.5	C52 H43 N6 S2 Ir	708.6	n/a	n/a	52	43	6	2	1

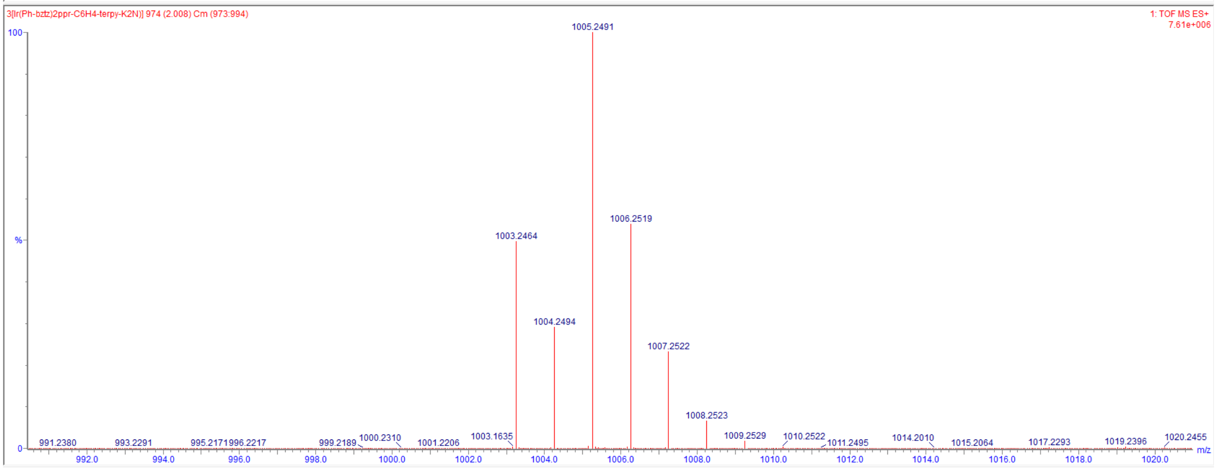

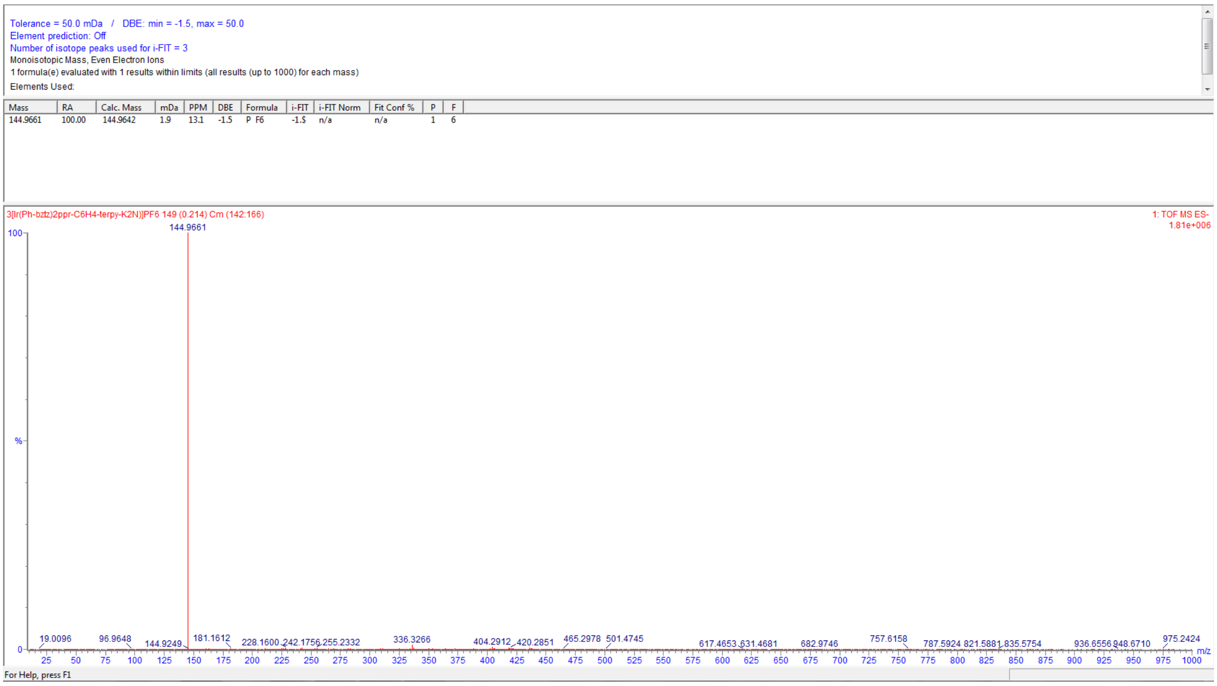

(c) **3** HRMS (ESI): calcd for $C_{52}H_{40}IrN_6S_2^+$ 1005.2491 found 1005.2385.

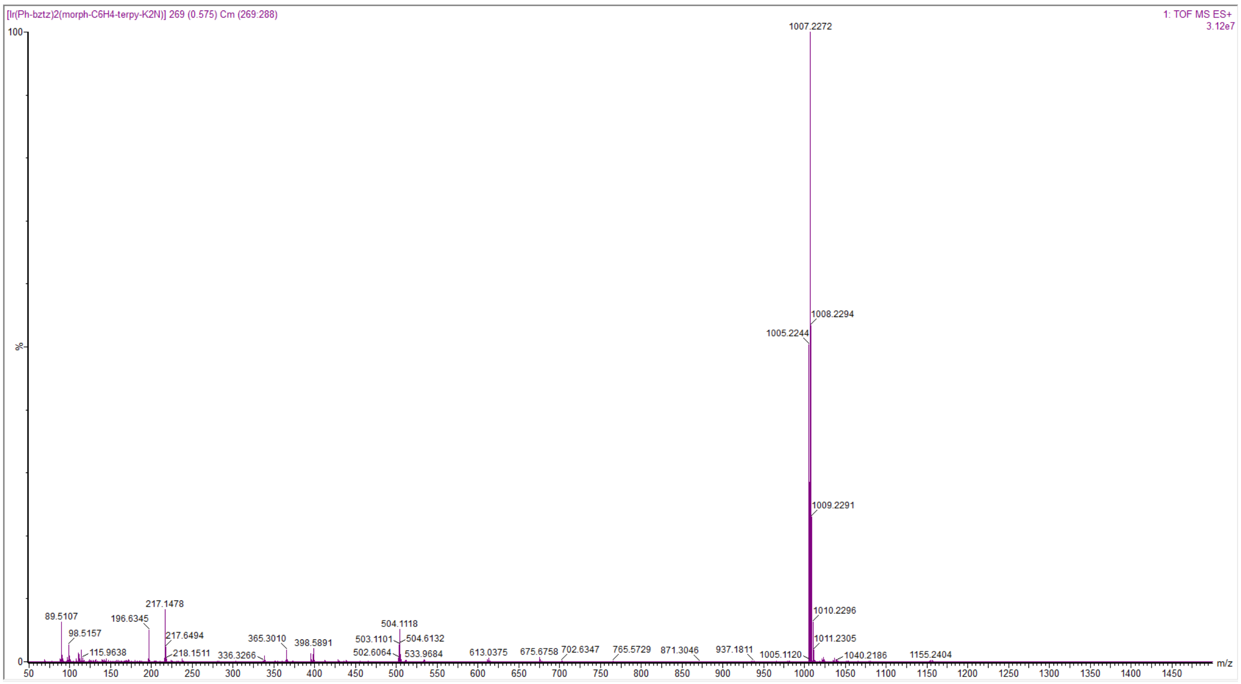

Multiple Mass Analysis: 6 mass(es) processed
Tolerance = 50.0 mDa / DBE: min = -1.5, max = 50.0
Element prediction: Off
Number of isotope peaks used for i-FIT = 3
Monoisotopic Mass, Odd and Even Electron Ions
53 formula(e) evaluated with 6 results within limits (up to 3 closest results for each mass)
Elements Used:

Mass	RA	Calc. Mass	mDa	PPM	DBE	Formula	i-FIT	i-FIT Norm	Fit Conf %	C	H	N	O	S	Ir
1005.2263	49.88	1005.2021	24.2	24.1	38.0	C51 H36 N6 O S2 Ir	634.1	n/a	n/a	51	36	6	1	2	1
1006.2291	28.89	1006.2099	19.2	19.1	37.5	C51 H37 N6 O S2 Ir	657.8	n/a	n/a	51	37	6	1	2	1
1007.2288	100.00	1007.2178	11.0	10.9	37.0	C51 H38 N6 O S2 Ir	625.9	n/a	n/a	51	38	6	1	2	1
1008.2317	54.48	1008.2256	6.1	6.1	36.5	C51 H39 N6 O S2 Ir	625.6	n/a	n/a	51	39	6	1	2	1
1009.2299	24.76	1009.2334	-3.5	-3.5	36.0	C51 H40 N6 O S2 Ir	625.7	n/a	n/a	51	40	6	1	2	1
1010.2289	8.04	1010.2412	-12.3	-12.2	35.5	C51 H41 N6 O S2 Ir	601.2	n/a	n/a	51	41	6	1	2	1

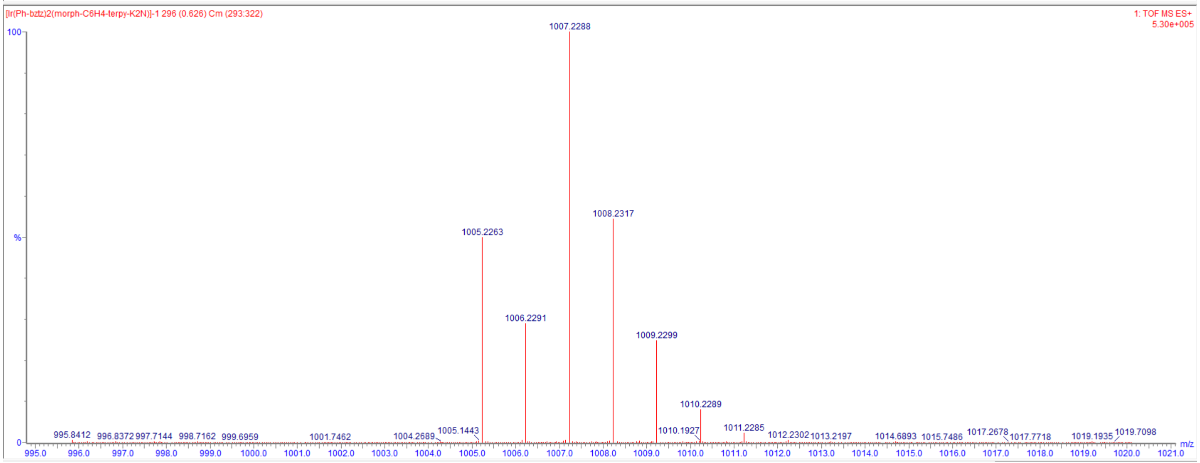

Tolerance = 50.0 mDa / DBE: min = -1.5, max = 50.0
Element prediction: Off
Number of isotope peaks used for i-FIT = 3
Monoisotopic Mass, Even Electron Ions
1 formula(e) evaluated with 1 results within limits (up to 3 closest results for each mass)
Elements Used:

Mass	RA	Calc. Mass	mDa	PPM	DBE	Formula	i-FIT	i-FIT Norm	Fit Conf %	F	P
144.9659	100.00	144.9642	1.7	11.7	-1.5	F6 P	-1.5	n/a	n/a	6	1

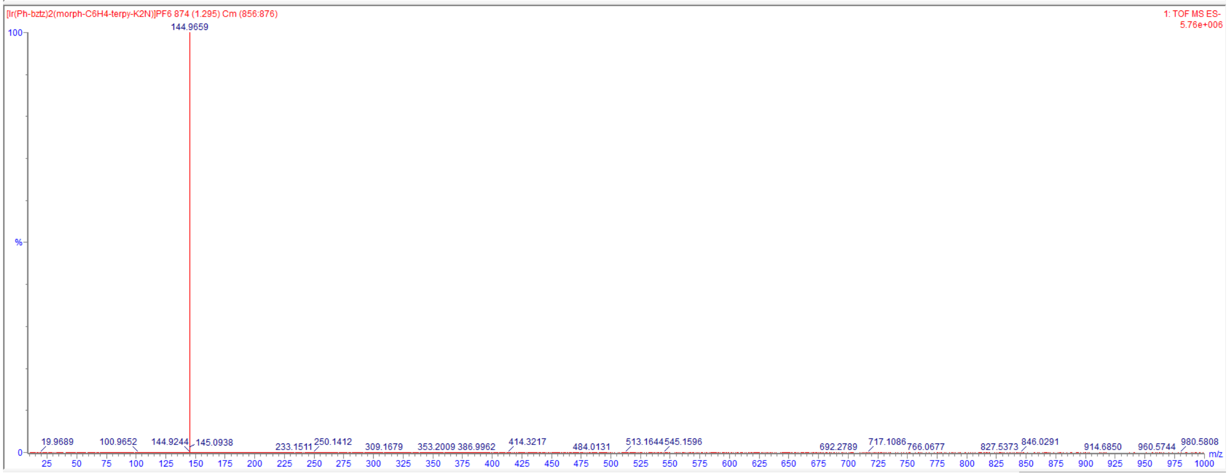

(d) **4** HRMS (ESI): calcd for $C_{51}H_{38}IrON_6S_2^+$ 1007.2178 found 1007.2288

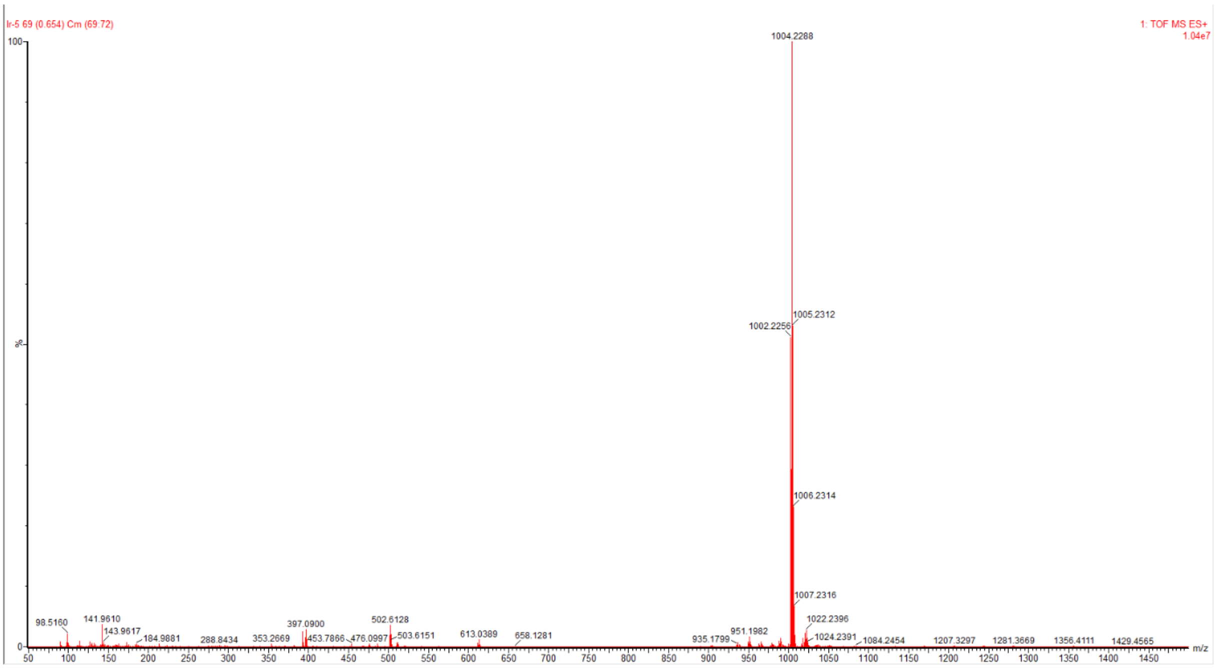

Multiple Mass Analysis: 6 mass(es) processed
Tolerance = 50.0 mDa / DBE: min = -1.5, max = 50.0
Element prediction: Off
Number of isotope peaks used for i-FIT = 3
Monoisotopic Mass, Odd and Even Electron Ions
54 formula(e) evaluated with 6 results within limits (up to 3 closest results for each mass)
Elements Used:

Mass	RA	Calc. Mass	mDa	PPM	DBE	Formula	i-FIT	i-FIT Norm	Fit Conf %	C	H	N	S	Ir
1002.2258	51.18	1002.2025	23.3	23.2	39.0	C51 H35 N7 S2 Ir	803.7	n/a	n/a	51	35	7	2	1
1003.2285	29.41	1003.2103	18.2	18.1	38.5	C51 H36 N7 S2 Ir	790.0	n/a	n/a	51	36	7	2	1
1004.2285	100.00	1004.2181	10.4	10.4	38.0	C51 H37 N7 S2 Ir	785.2	n/a	n/a	51	37	7	2	1
1005.2311	53.53	1005.2259	5.2	5.2	37.5	C51 H38 N7 S2 Ir	763.6	n/a	n/a	51	38	7	2	1
1006.2313	23.26	1006.2338	-2.5	-2.5	37.0	C51 H39 N7 S2 Ir	717.5	n/a	n/a	51	39	7	2	1
1007.2316	6.72	1007.2416	-10.0	-9.9	36.5	C51 H40 N7 S2 Ir	699.5	n/a	n/a	51	40	7	2	1

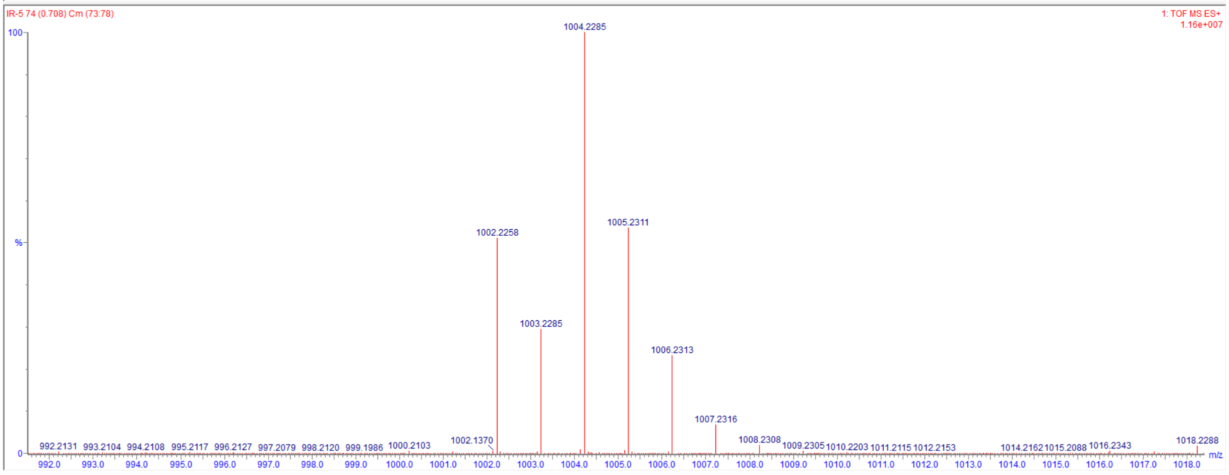

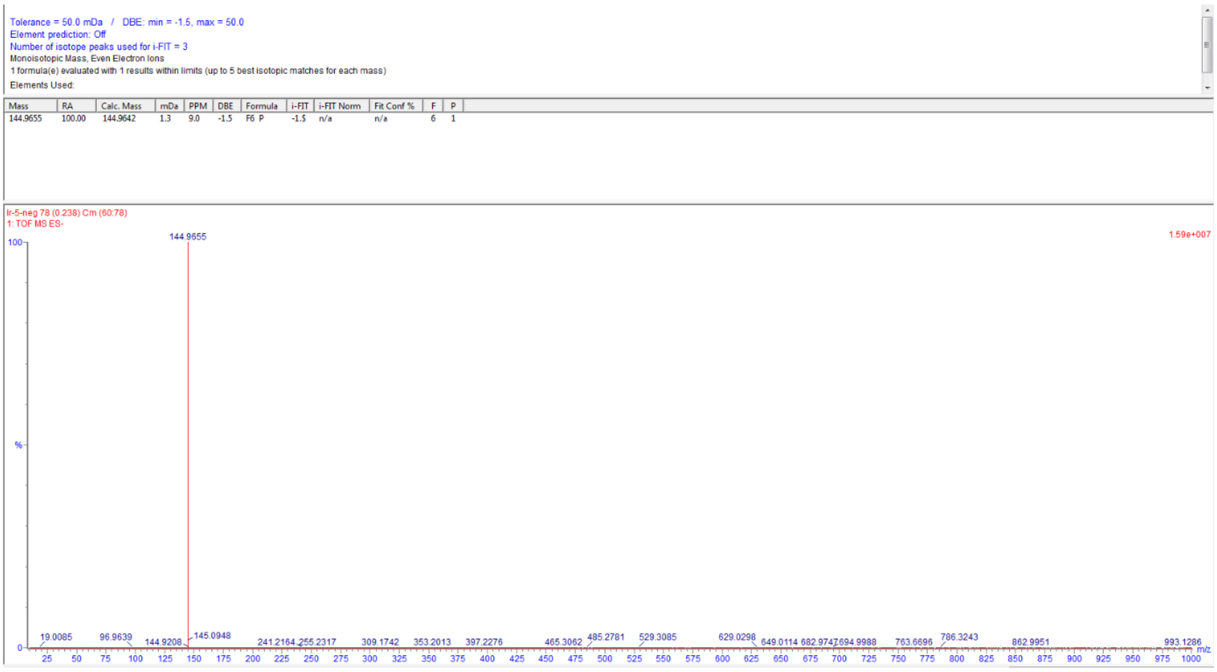

(e) **5** HRMS (ESI): calcd for $C_{52}H_{40}IrN_6S_2^{+}$ 1004.2181 found 1004.2285

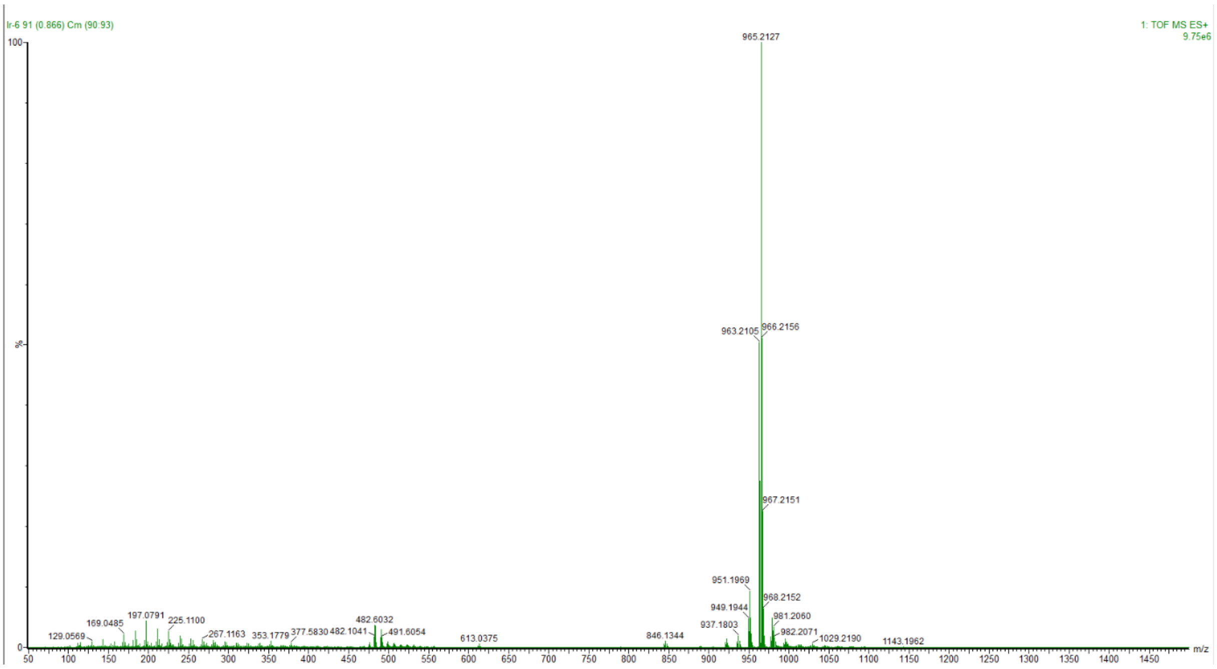

Multiple Mass Analysis: 3 mass(es) processed
Tolerance = 50.0 mDa / DBE: min = -1.5, max = 50.0
Element prediction: Off
Number of isotope peaks used for i-FIT = 3
Monoisotopic Mass, Odd and Even Electron Ions
26 formula(e) evaluated with 3 results within limits (up to 5 best isotopic matches for each mass)
Elements Used:

Mass	RA	Calc. Mass	mDa	PPM	DBE	Formula	i-FIT	i-FIT Norm	Fit Conf %	C	H	N	S	Ir
963.2105	50.43	963.1916	18.9	19.6	37.0	C49 H34 N6 S2 Ir	774.6	n/a	n/a	49	34	6	2	1
965.2127	100.00	965.2072	5.5	5.7	36.0	C49 H36 N6 S2 Ir	755.8	n/a	n/a	49	36	6	2	1
966.2156	51.09	966.2150	0.6	0.6	35.5	C49 H37 N6 S2 Ir	725.8	n/a	n/a	49	37	6	2	1

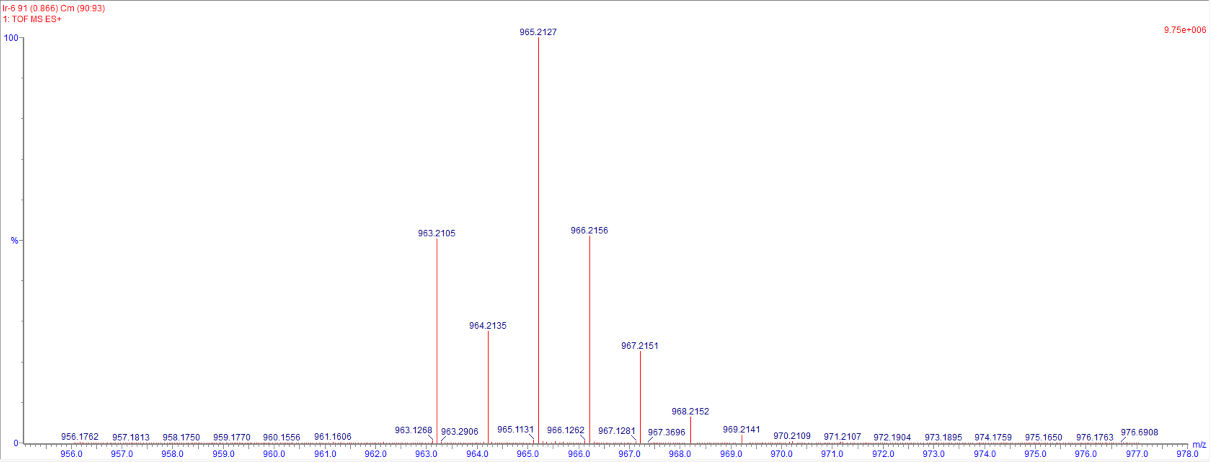

Tolerance = 50.0 mDa / DBE: min = -1.5, max = 50.0
Element prediction: Off
Number of isotope peaks used for i-FIT = 3
Monoisotopic Mass, Even Electron Ions
1 formula(e) evaluated with 1 results within limits (up to 5 best isotopic matches for each mass)
Elements Used:

Mass	RA	Calc. Mass	mDa	PPM	DBE	Formula	i-FIT	i-FIT Norm	Fit Conf %	F	P
144.9654	100.00	144.9642	1.2	8.3	-1.5	F6 P	-1.5	n/a	n/a	6	1

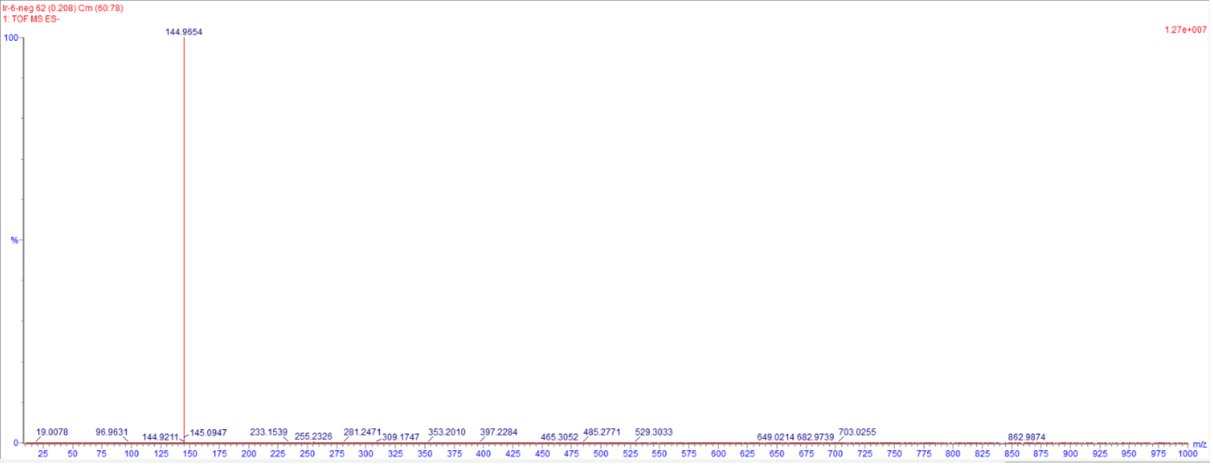

(f) **6** HRMS (ESI): calcd for $C_{49}H_{36}IrN_6S_2^+$ 965.2072 found 965.2127.

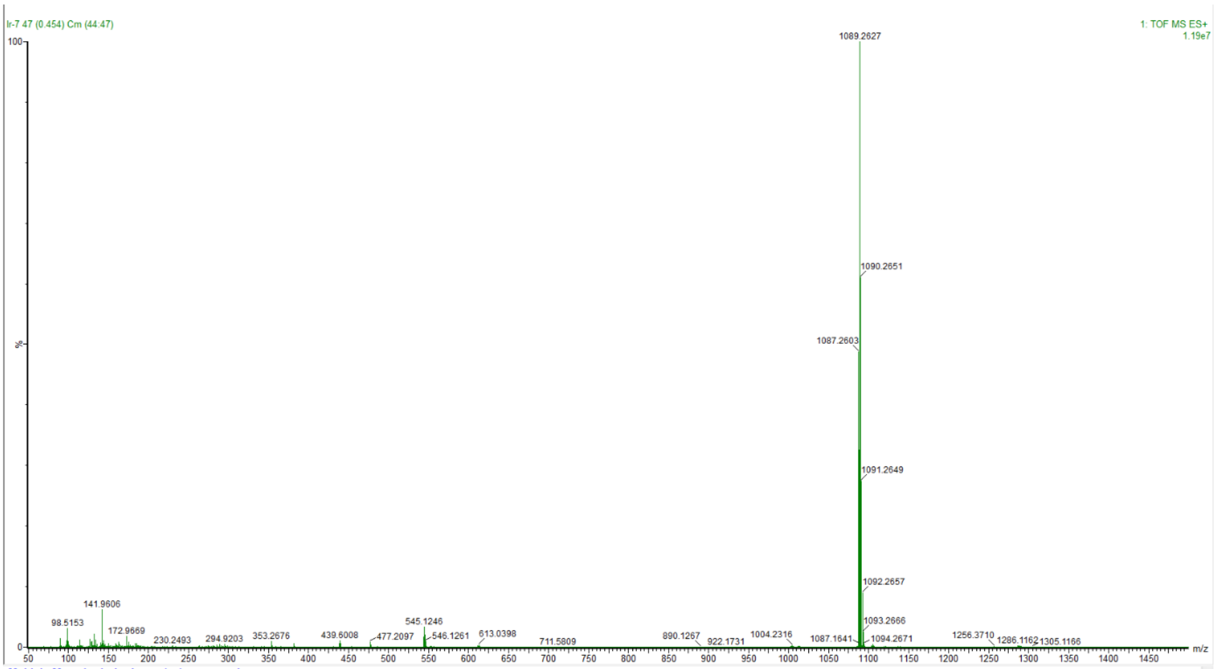

Multiple Mass Analysis: 4 mass(es) processed
Tolerance = 50.0 mDa / DBE: min = -1.5, max = 50.0
Element prediction: Off
Number of isotope peaks used for i-FIT = 3
Monoisotopic Mass, Odd and Even Electron Ions
35 formula(e) evaluated with 4 results within limits (up to 5 best isotopic matches for each mass)
Elements Used:

Mass	RA	Calc. Mass	mDa	PPM	DBE	Formula	i-FIT	i-FIT Norm	Fit Conf %	C	H	N	S	Ir
1087.2603	48.79	1087.2229	37.4	34.4	45.0	C59 H38 N6 S2 Ir	722.6	n/a	n/a	59	38	6	2	1
1088.2629	32.50	1088.2307	32.2	29.6	44.5	C59 H39 N6 S2 Ir	707.9	n/a	n/a	59	39	6	2	1
1089.2627	100.00	1089.2385	24.2	22.2	44.0	C59 H40 N6 S2 Ir	691.4	n/a	n/a	59	40	6	2	1
1090.2651	60.99	1090.2463	18.8	17.2	43.5	C59 H41 N6 S2 Ir	676.2	n/a	n/a	59	41	6	2	1

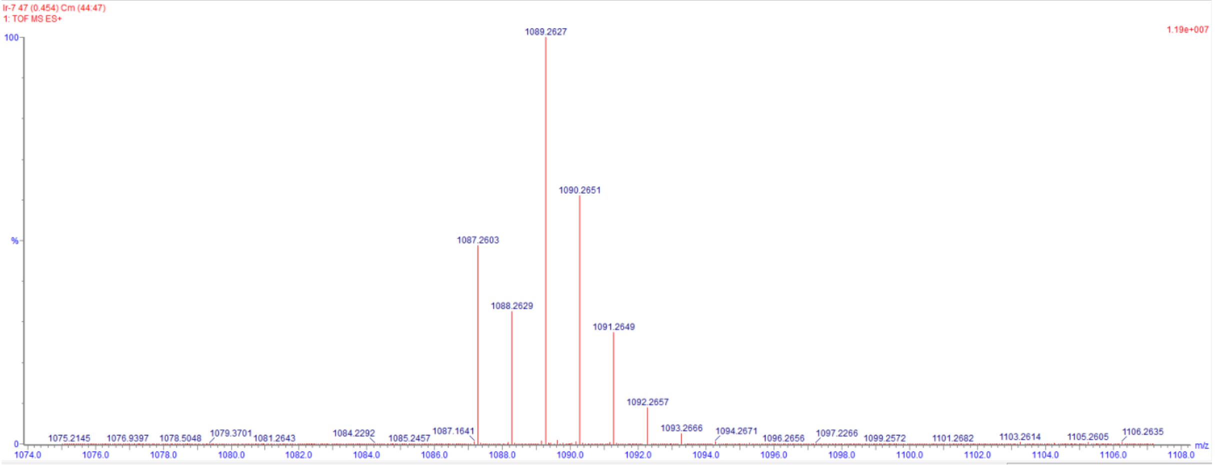

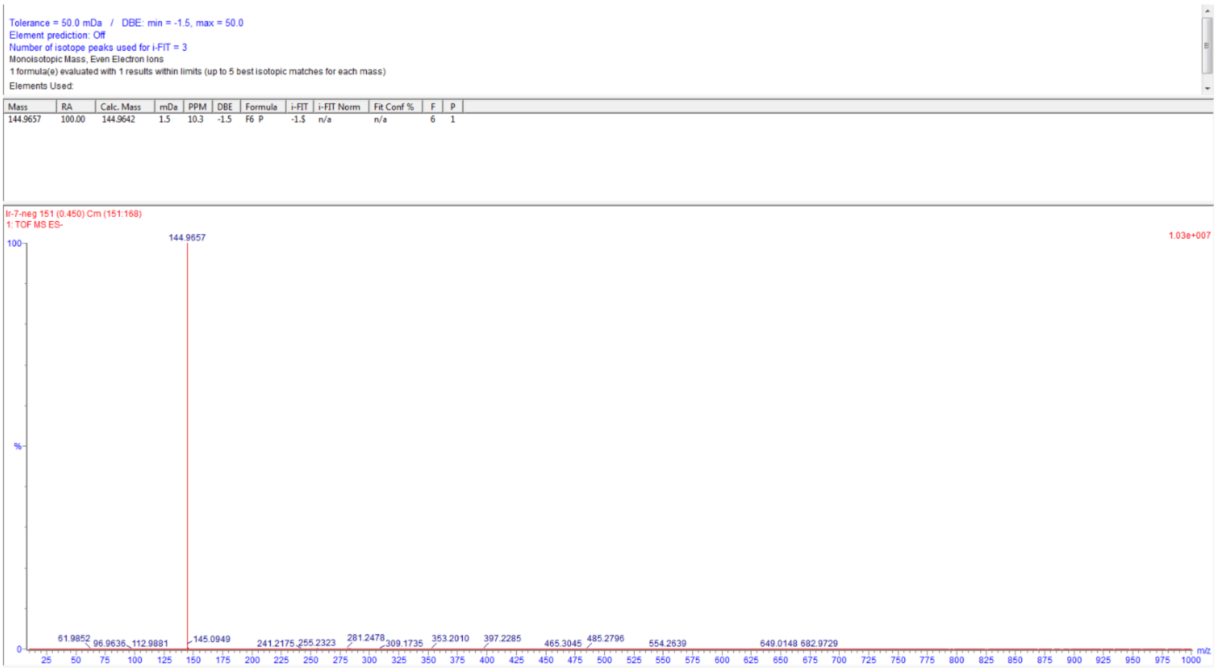

(g) **7** HRMS (ESI): calcd for $C_{59}H_{40}IrN_6S_2^{+}$ 1089.2385 found 1089.2627.

Figure S1. HR-ESI-MS spectra of **1–7** (a–g).

NMR spectroscopy

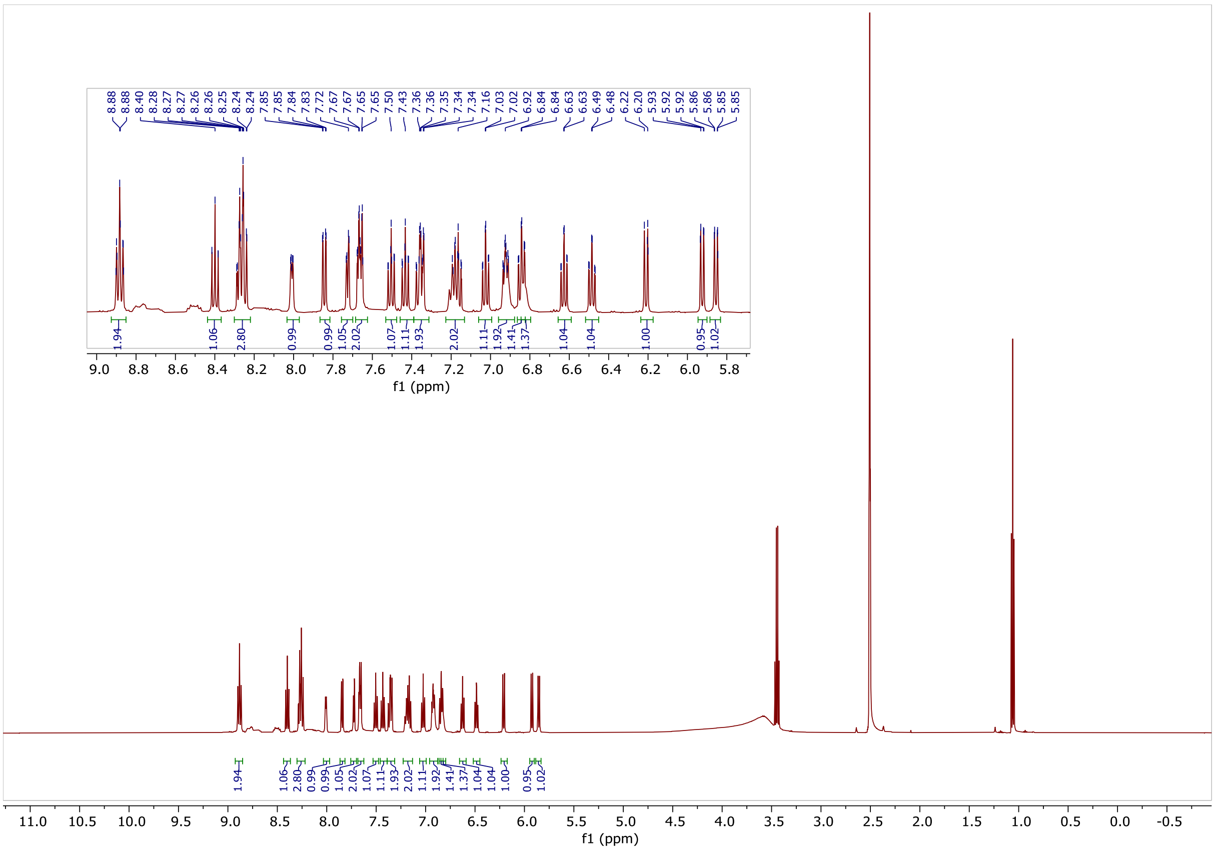

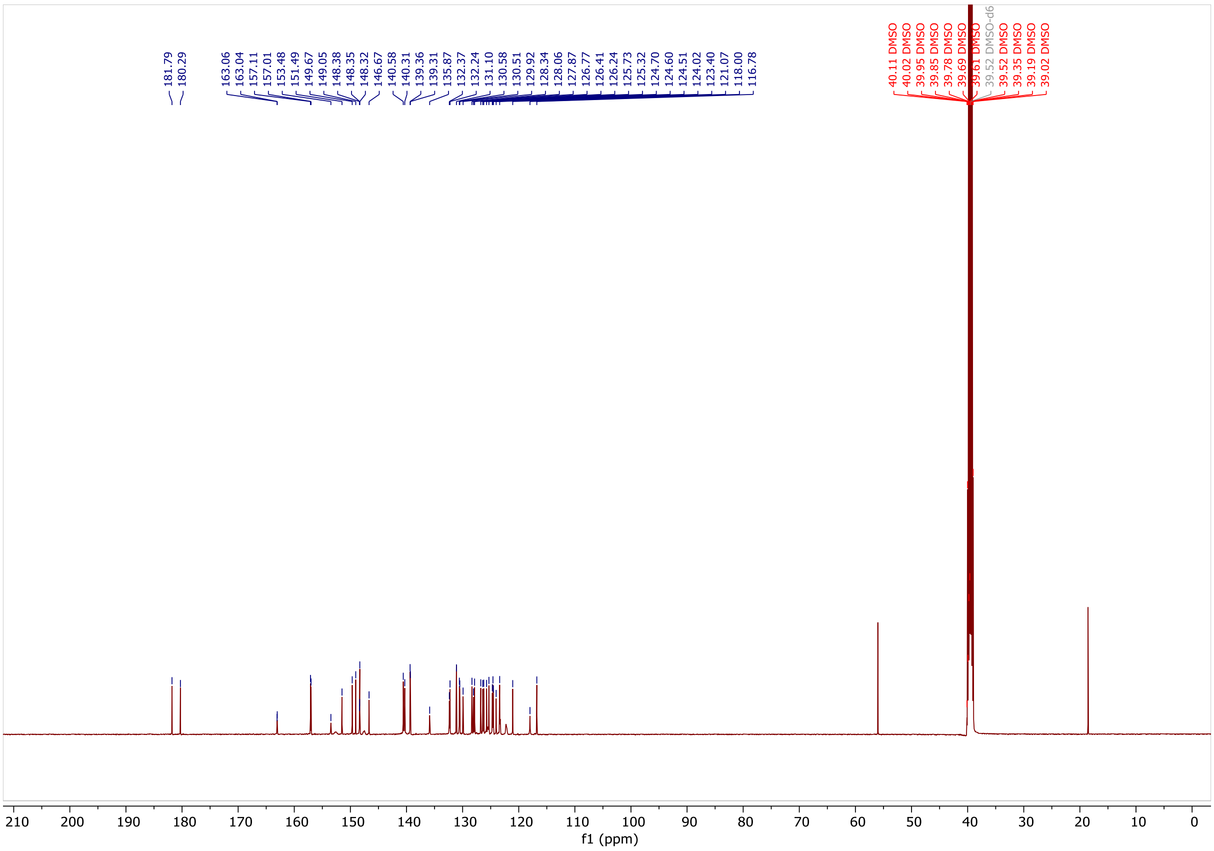

(a) ^{1}H and $^{13}C\{H\}$ NMR spectra of compound **1** in DMSO-d_6.

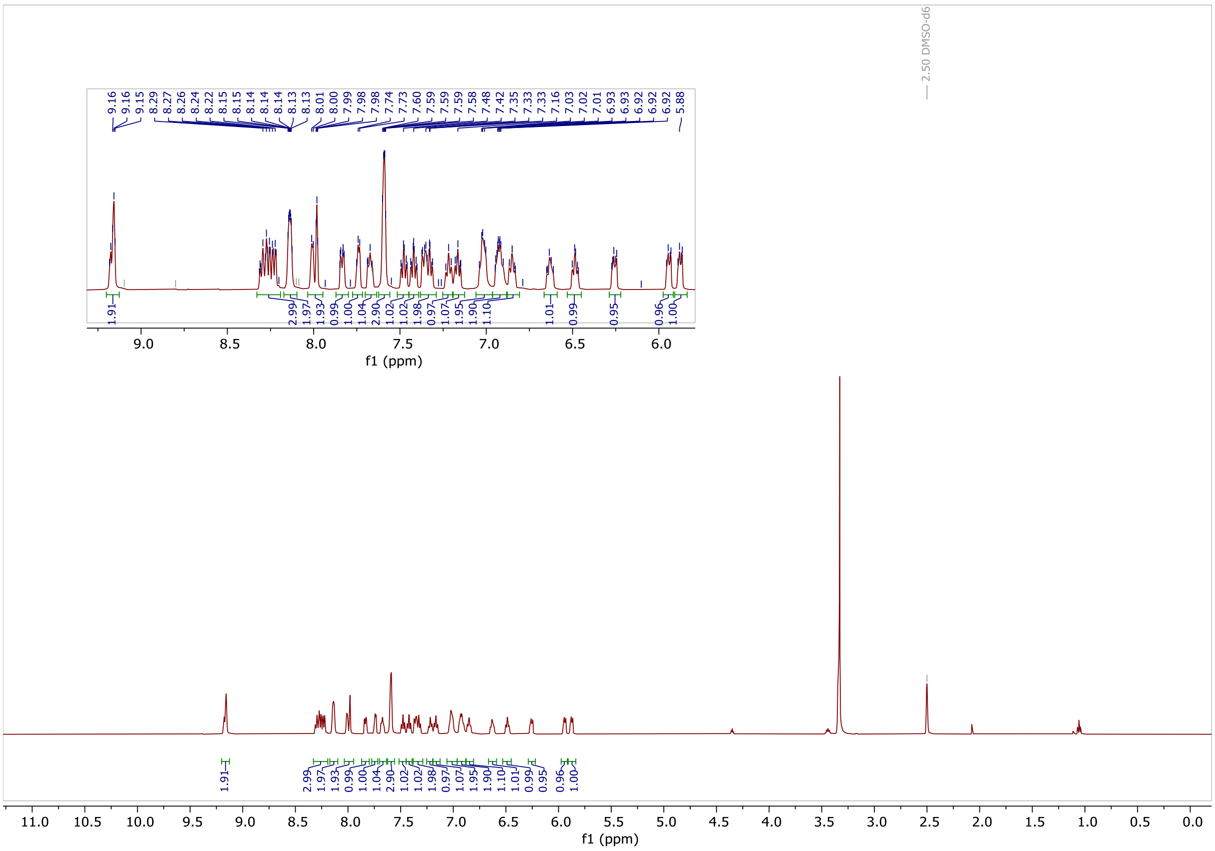

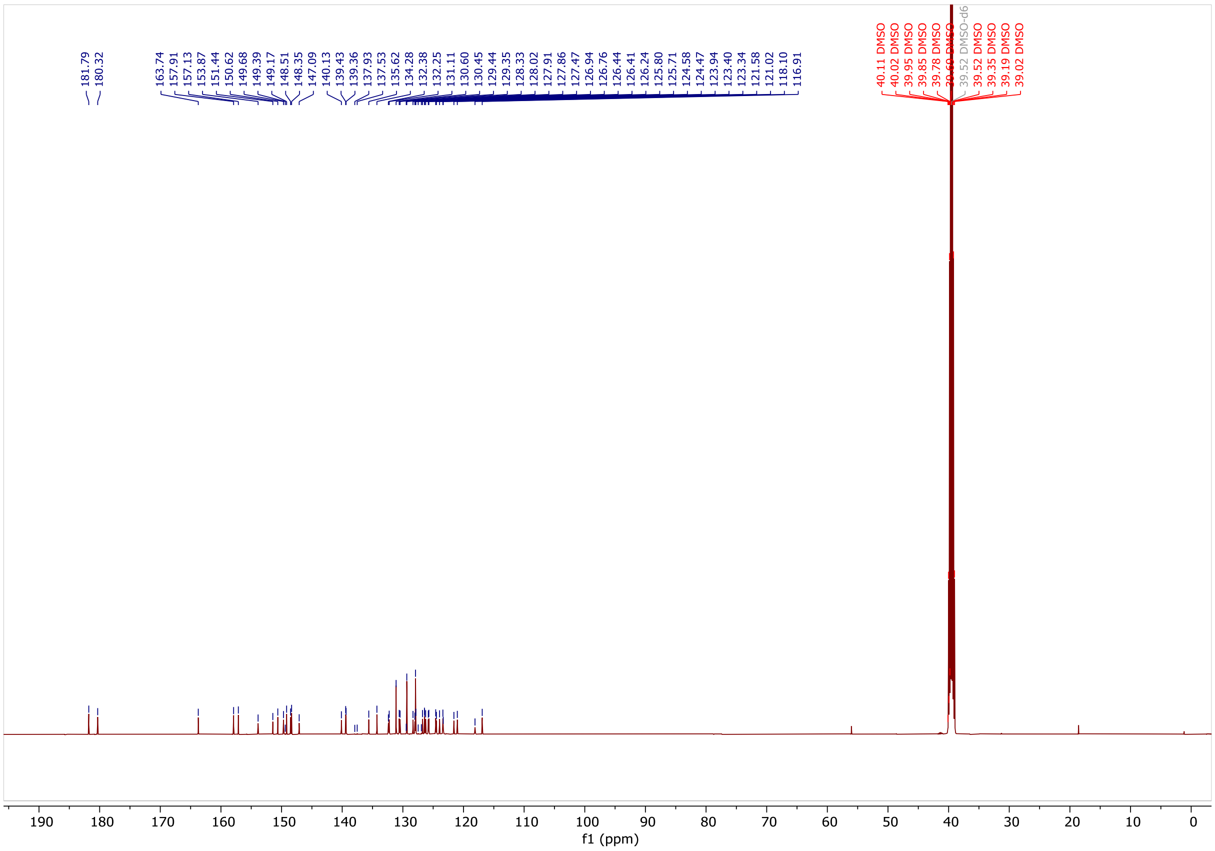

(b) ^{1}H and $^{13}C\{H\}$ NMR spectra of compound **2** in DMSO-d_6.

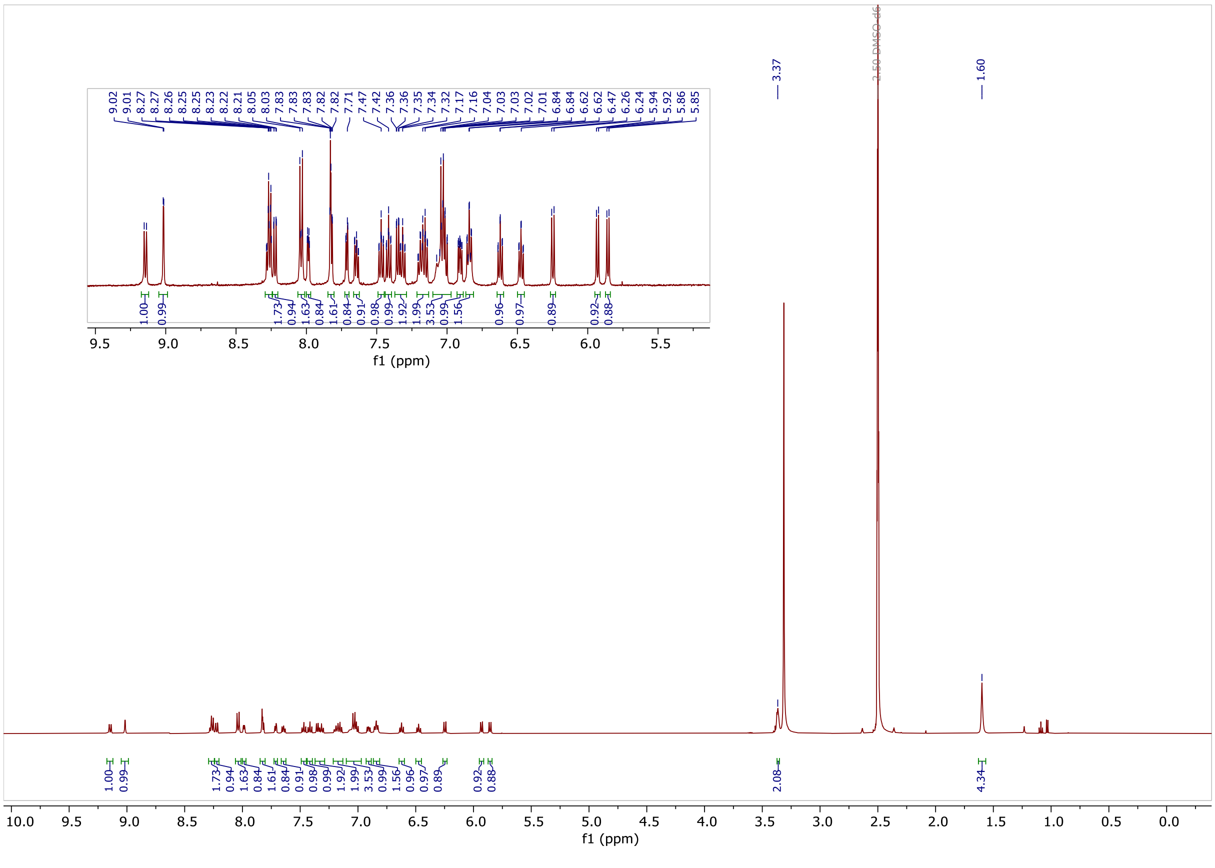

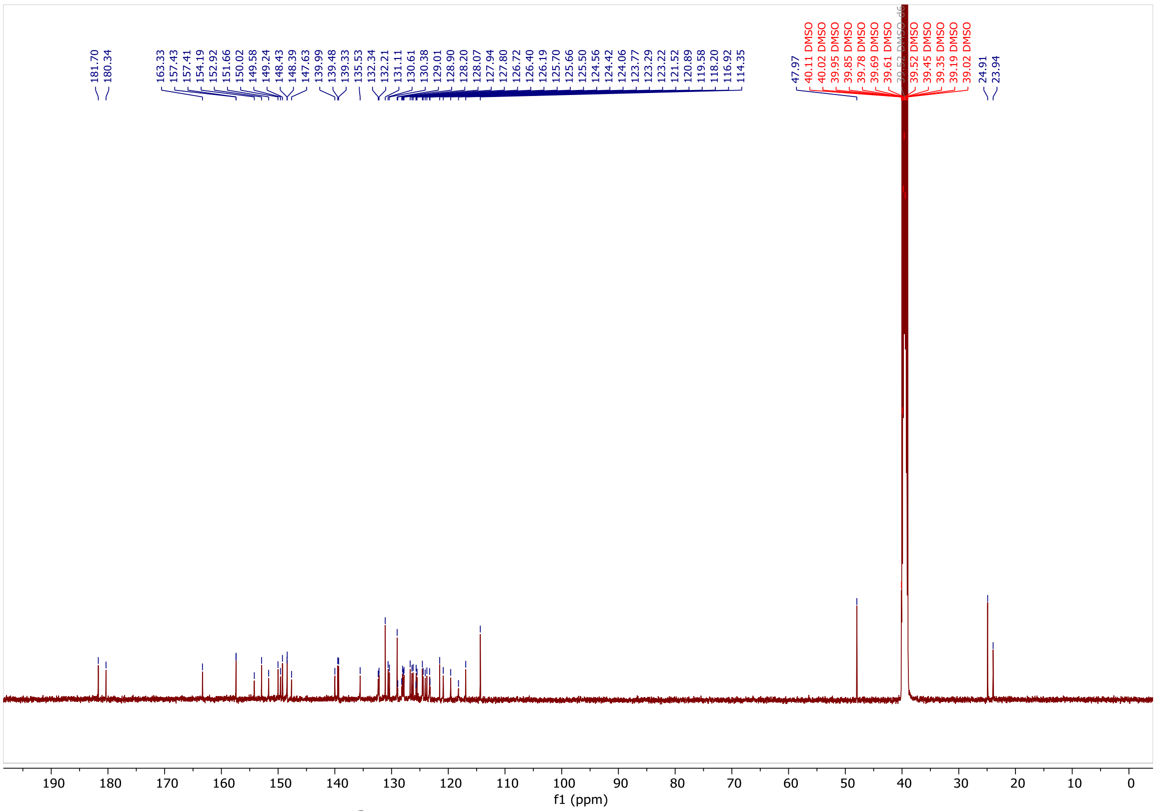

(c) ^{1}H and $^{13}C\{H\}$ NMR spectra of compound **3** in DMSO-d_6.

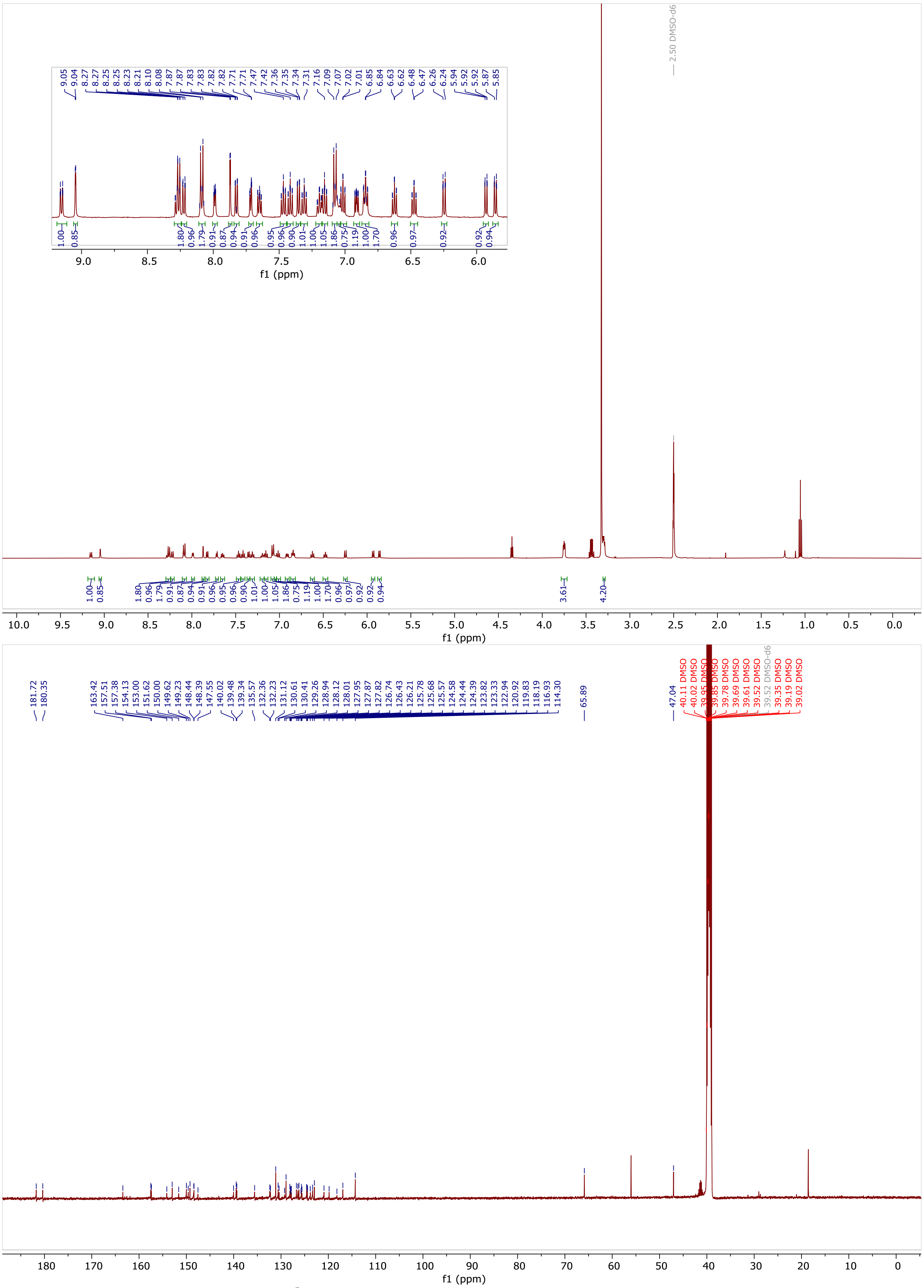

(d) ^{1}H and ^{13}C{H} NMR spectra of compound **4** in DMSO-d_6.

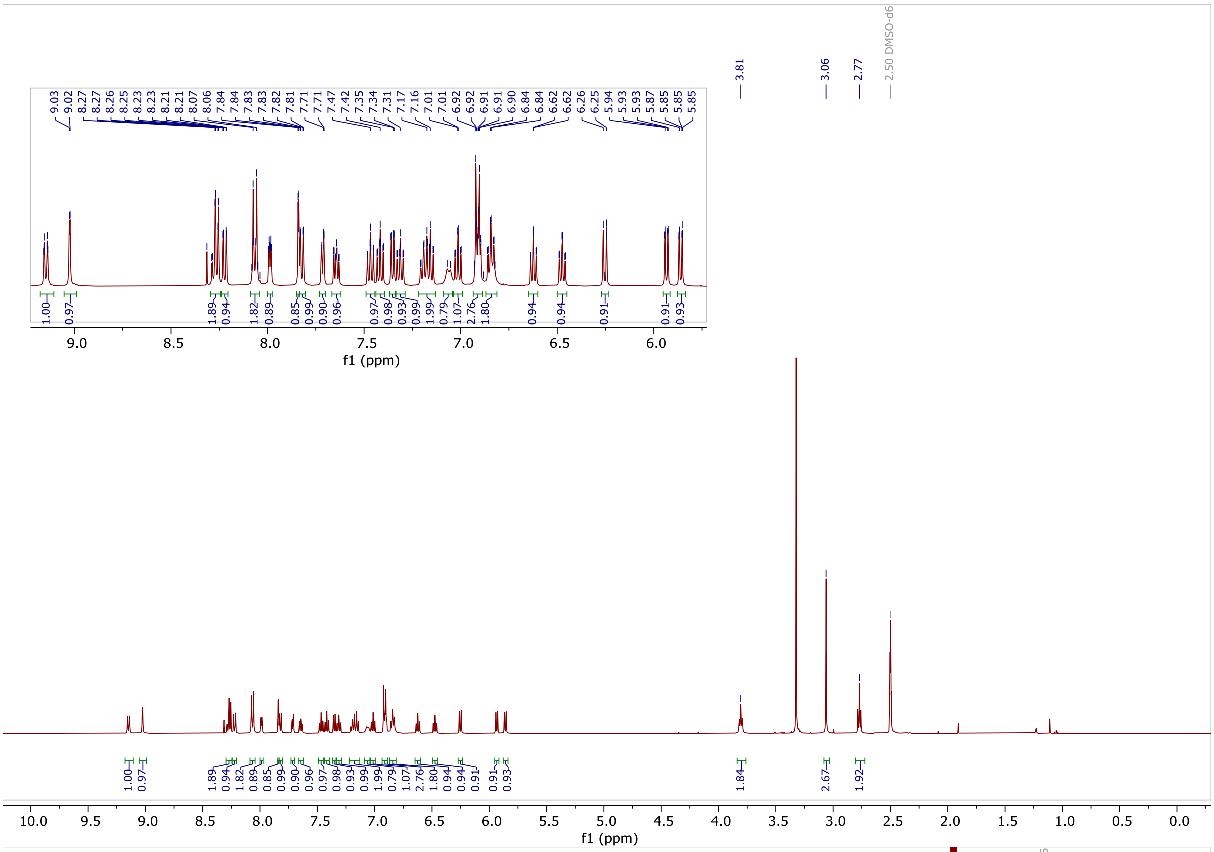

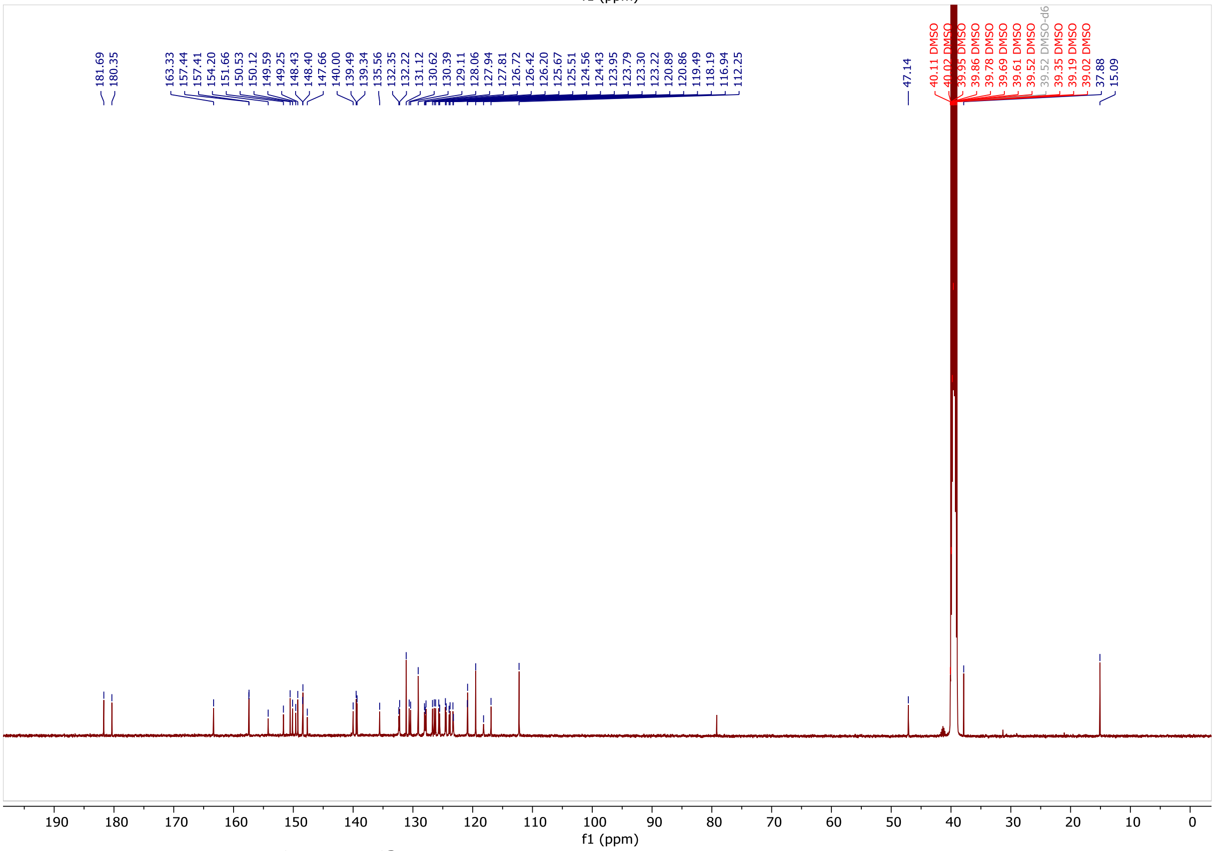

(e) ^{1}H and $^{13}C\{H\}$ NMR spectra of compound **5** in DMSO-d_6.

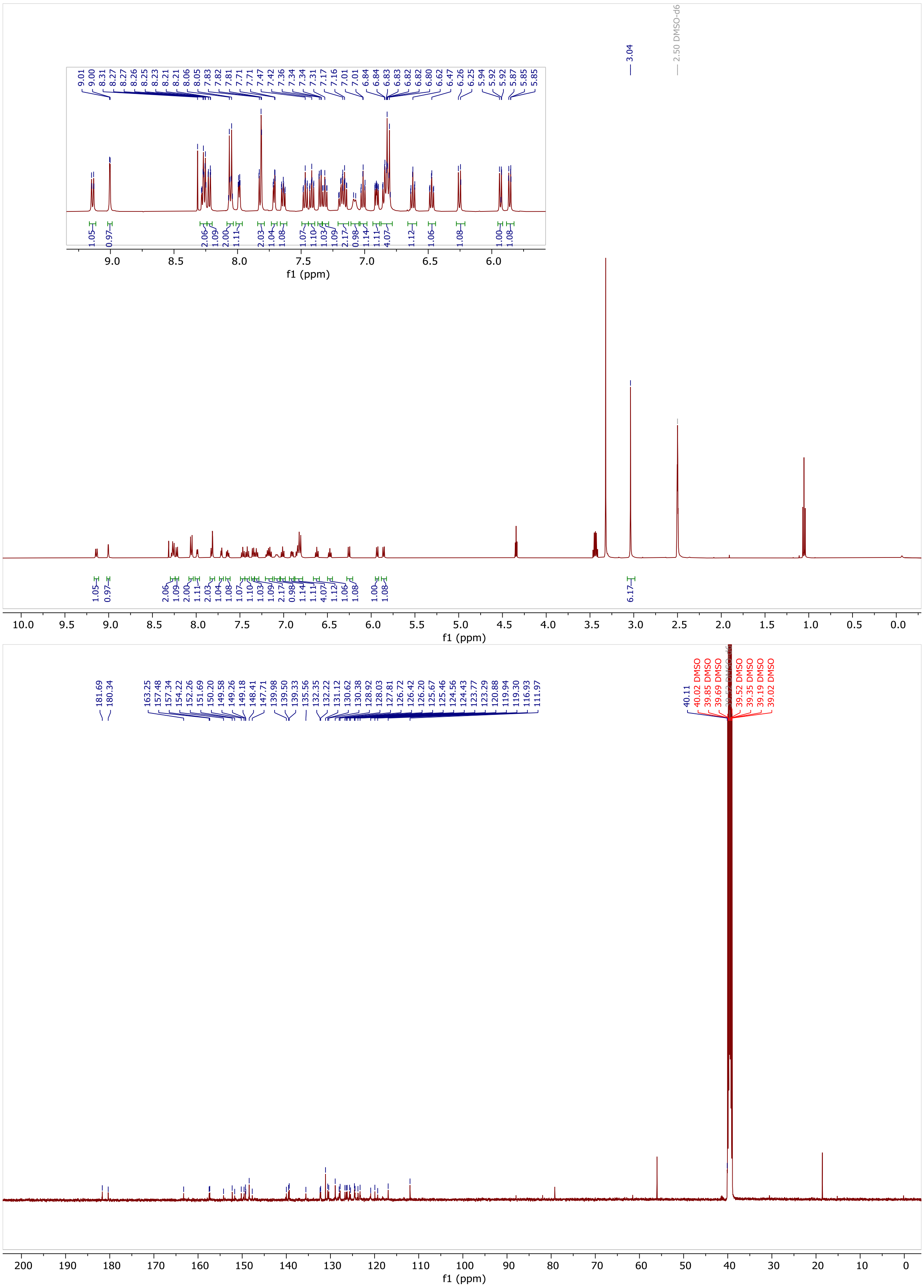

(f) ^{1}H and $^{13}C\{H\}$ NMR spectra of compound **6** in DMSO-d_6.

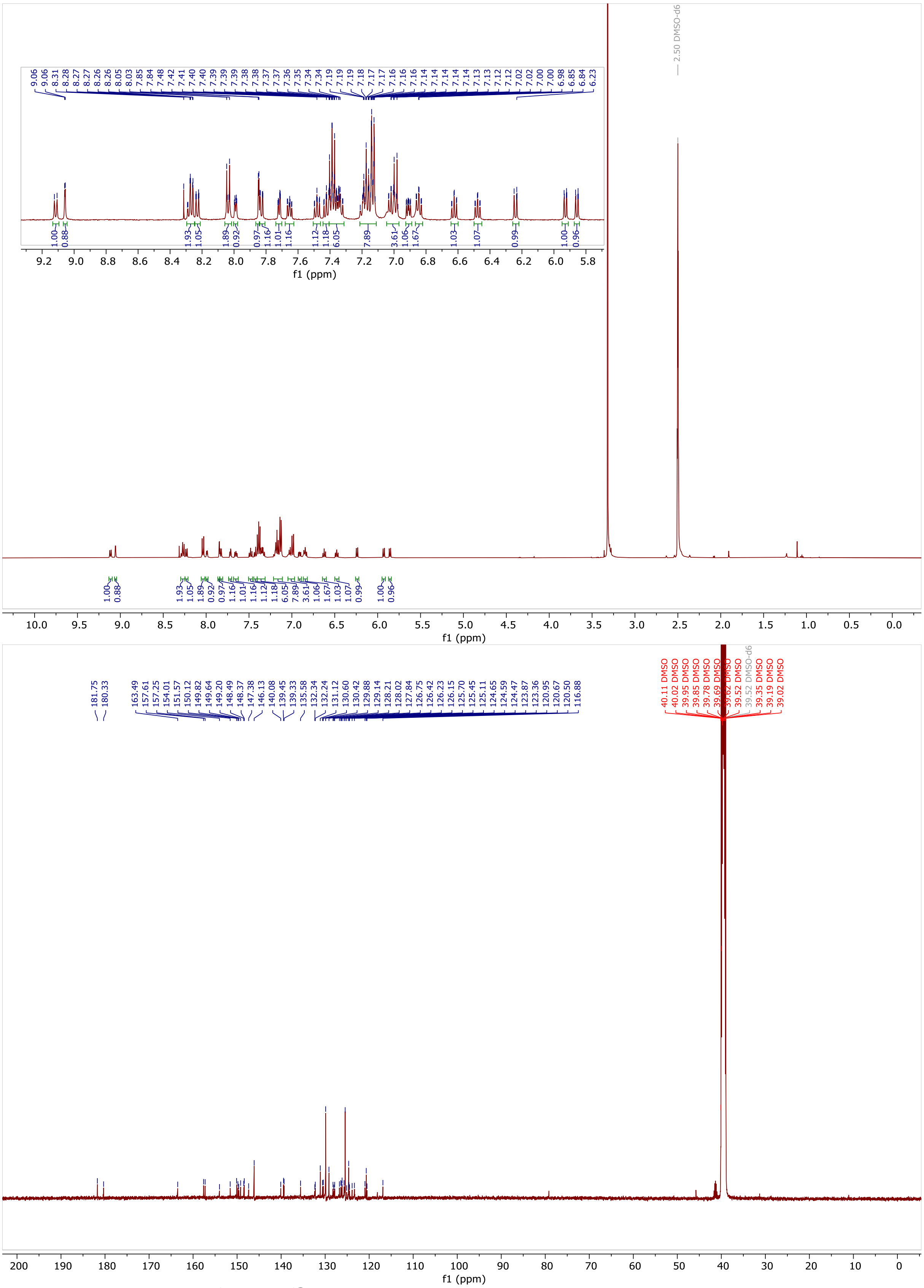

(g) ^{1}H and $^{13}C\{H\}$ NMR spectra ofcompound **7** in DMSO-d$_6$.

Figure S2. NMR spectra of **1**–**7** (a–g).

FT-IR spectroscopy

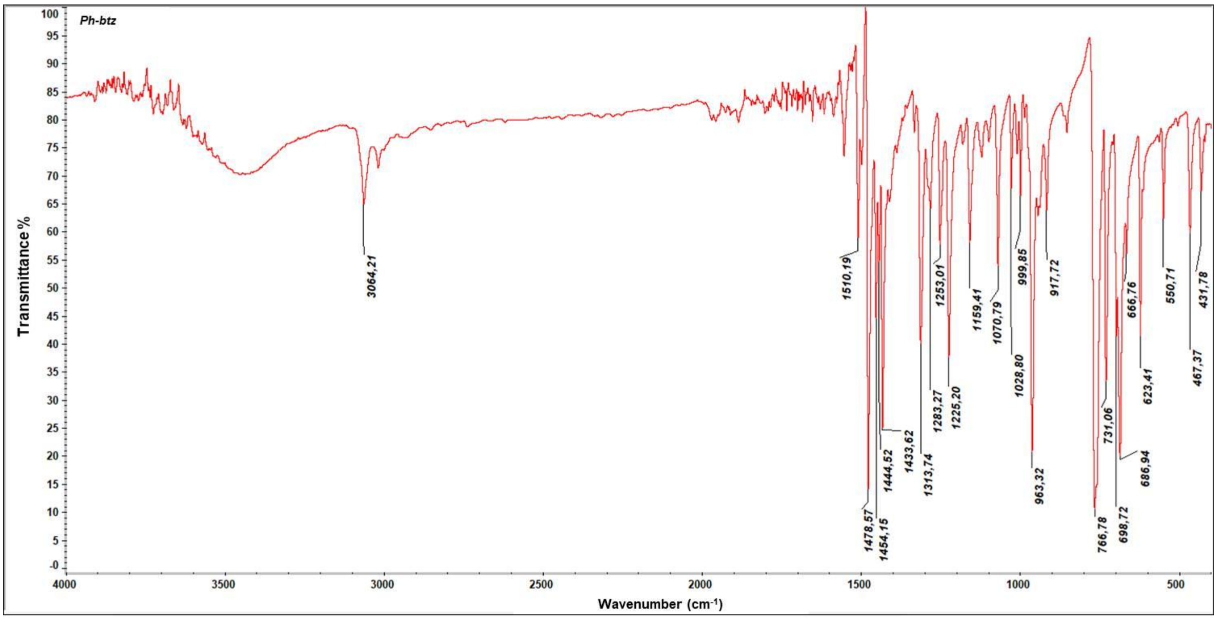

(a)

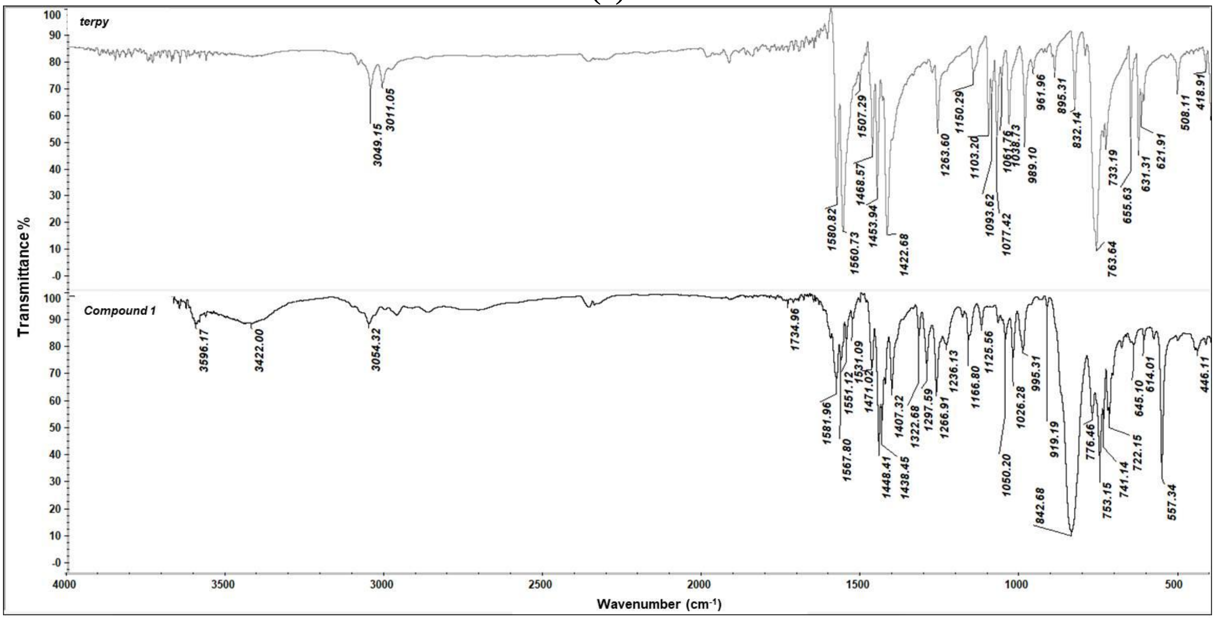

(b)

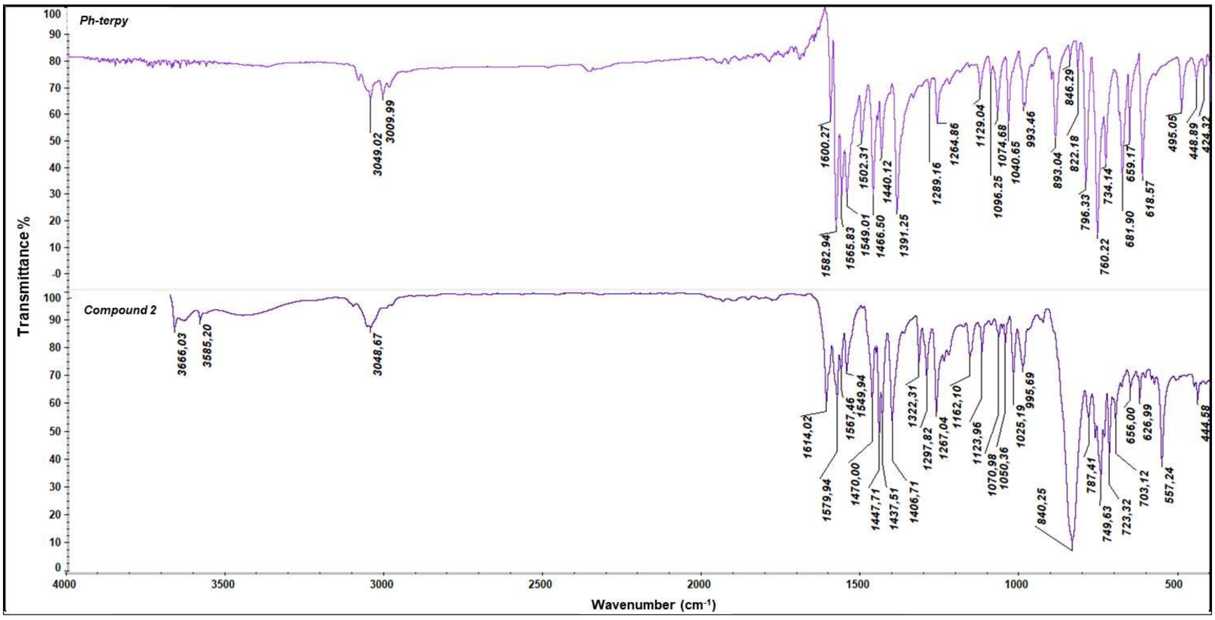

Ph-terpy
Compound 2
Transmittance %
Wavenumber (cm-1)
3049.02
3009.99
1600.27
1582.94
1565.83
1549.01
1502.31
1466.50
1440.12
1391.25
1289.16
1264.86
1129.04
1096.25
1074.68
1040.65
993.46
893.04
846.29
822.18
796.33
760.22
734.14
681.90
659.17
618.57
495.05
448.89
424.32
3666,03
3585,20
3048,67
1614,02
1579,94
1567,46
1549,94
1470,00
1447,71
1437,51
1406,71
1322,31
1297,82
1267,04
1162,10
1123,96
1070,98
1050,36
1025,19
995,69
840,25
787,41
749,63
723,32
703,12
656,00
626,99
557,24
444,58

(c)

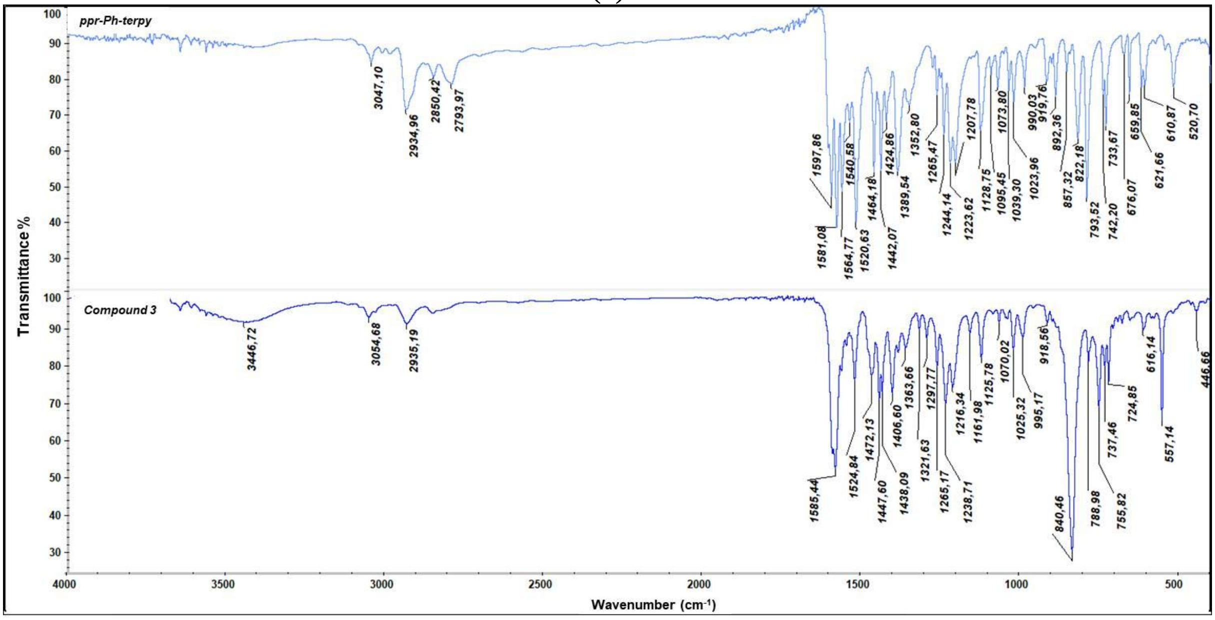

ppr-Ph-terpy
Compound 3
Transmittance %
Wavenumber (cm-1)
3047,10
2934,96
2850,42
2793,97
1597,86
1581,08
1564,77
1540,58
1520,63
1464,18
1442,07
1424,86
1389,54
1352,80
1265,47
1244,14
1223,62
1207,78
1128,75
1095,45
1073,80
1039,30
1023,96
990,03
919,76
892,36
857,32
822,18
793,52
742,20
733,67
676,07
659,85
621,66
610,87
520,70
3446,72
3054,68
2935,19
1585,44
1524,84
1472,13
1447,60
1438,09
1406,60
1363,66
1321,63
1297,77
1265,17
1238,71
1216,34
1161,98
1125,78
1070,02
1025,32
995,17
918,56
840,46
788,98
755,82
737,46
724,85
616,14
557,14
446,66

(d)

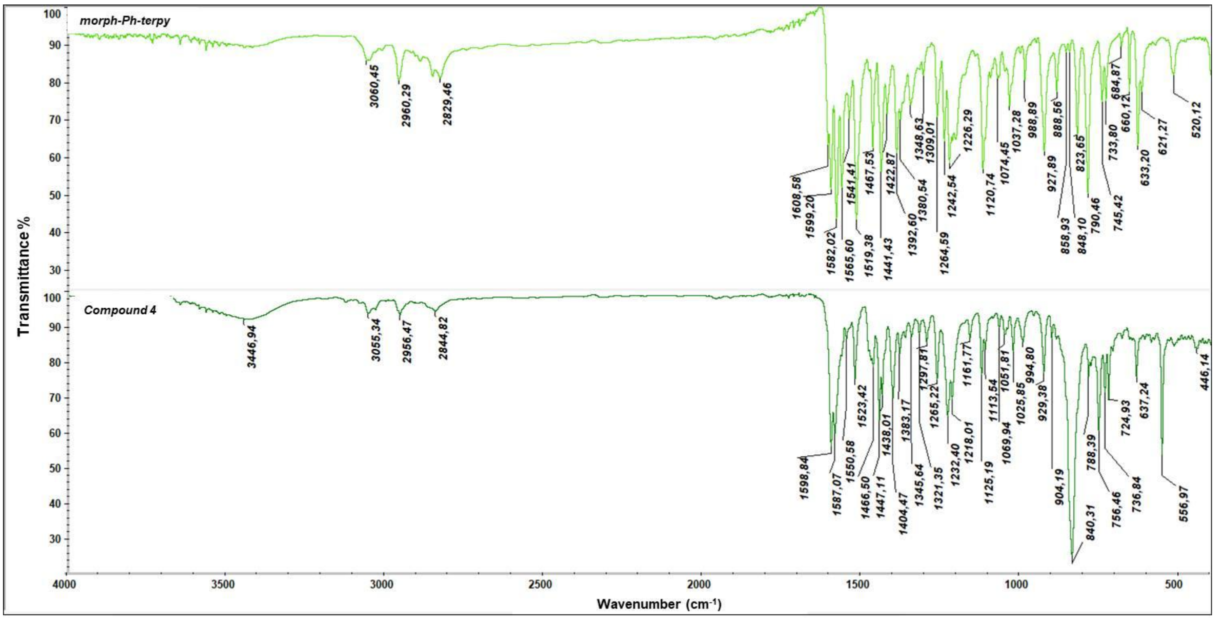
morph-Ph-terpy
Compound 4
Transmittance %
Wavenumber (cm-1)

(e)

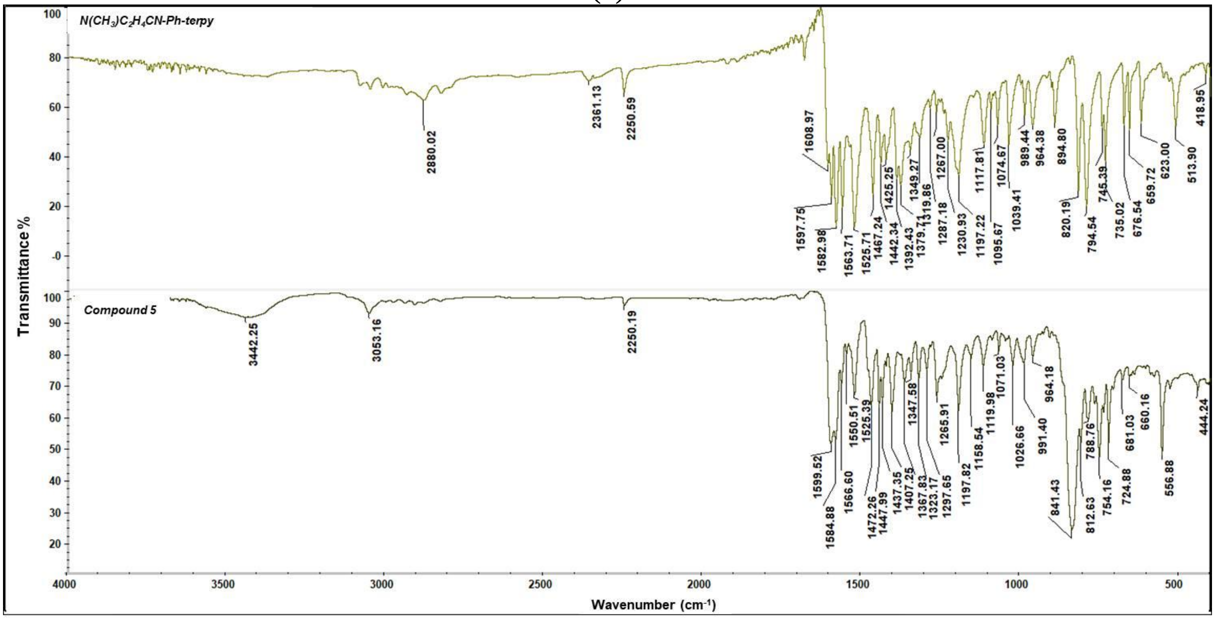
N(CH3)C2H4CN-Ph-terpy
Compound 5
Transmittance %
Wavenumber (cm-1)

(f)

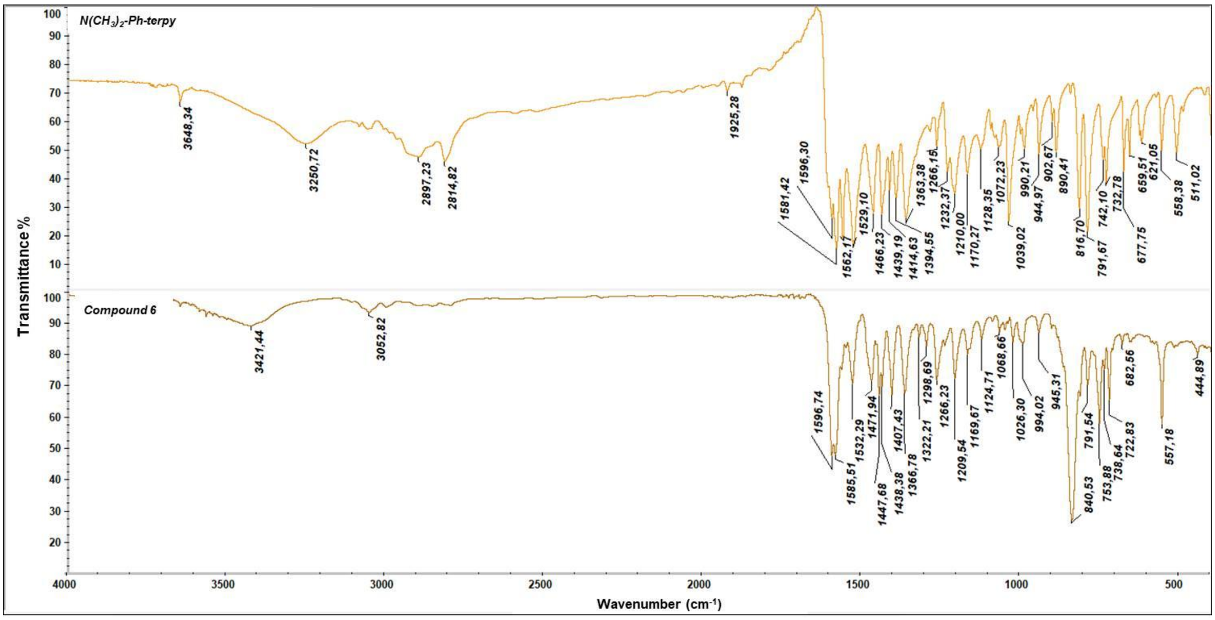

(g)

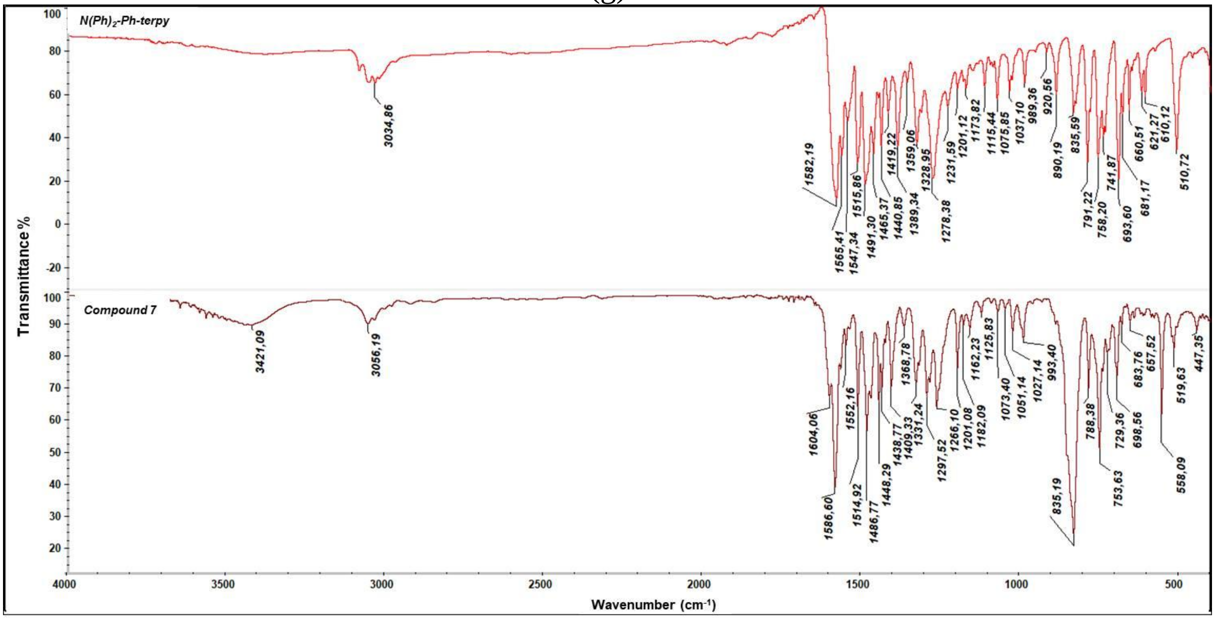

(h)

Figure S3. **FT-**IR spectra of Ph-btz (a) and **1**–**7** along with those for the free ligands (b–h).

X-Ray analysis

Table S1. Crystal data and structure refinement of complexes **2** and **6**.

	**2**	**6**
Empirical formula	$C_{47}H_{31}F_6IrN_5PS_2$	$C_{50}H_{40}F_6IrN_6OPS_2$
Formula weight	1067.06	1142.17
Temperature [K]	293.0(2)	293.0(2)
Wavelength [Å]	0.71073	0.71073
Crystal system	*Monoclinic*	*Triclinic*
Space group	$P2_1/c$	$P\bar{1}$
Unit cell dimensions [Å, °]	a = 17.2520(8)	a = 12.2671(5)
	b = 12.7739(4)	b = 14.2951(6)
	c = 21.2498(8)	c = 14.5500(6)

	β = 110.446(5)	α = 101.517(4)<br>β = 95.605(4)<br>γ = 109.851(4)
Volume [Å ^{3}]	4387.9(3)	2313.34(18)
Z	4	2
Density (calculated) [g/cm^3]	1.615	1.640
Absorption coefficient [mm^{-1}]	3.240	3.081
F(000)	2104.0	1136.0
Crystal size [mm]	0.195×0.155×0.09	0.210 × 0.145 × 0.095
θ range for data collection [°]	3.43 to 29.42	3.41 to 29.39
Index ranges	–23 ≤ h ≤ 21<br>–17 ≤ k ≤ 12<br>–26 ≤ l ≤ 26	–14 ≤ h ≤ 15<br>–19 ≤ k ≤ 19<br>–20 ≤ l ≤ 17
Reflections collected	24250	20900
Independent reflections	10438 (R_{int} = 0.0384)	10893 (R_{int} = 0.0453)
Completeness to 2θ=50.5º [%]	99.5	99.6
Max. and min. transmission	0.815 and 1.000	0.666 and 1.000
Data / restraints / parameters	10438/0/559	10893/0/608
Goodness-of-fit on F^2	1.027	1.021
Final R indices [I>2σ(I)]	R_1 = 0.0437<br>wR_2 = 0.0961	R_1 = 0.0486<br>wR_2 = 0.0997
R indices (all data)	R_1 = 0.0707<br>wR_2 = 0.1104	R_1 = 0.0687<br>wR_2 = 0.1116
Largest diff. peak and hole [eÅ^{-3}]	1.62/–1.86	2.20/–1.34

Table S2. Experimental bond lengths [Å] and angles [°] for **2** and **6**.

	**2**	**6**
**Bond lengths [Å]**		
Ir(1)–C(1)	2.025(5)	2.037(5)
Ir(1)–C(14)	2.006(5)	2.026(5)
Ir(1)–N(1)	2.051(4)	2.058(4)
Ir(1)–N(2)	2.064(4)	2.049(4)
Ir(1)–N(3)	2.145(4)	2.129(4)
Ir(1)–N(4)	2.218(4)	2.206(4)
**Bond angles [°]**		
N(1)–Ir(1)–N(2)	169.34(17)	169.25(15)
N(1)–Ir(1)–N(3)	103.06(16)	102.58(16)
N(1)–Ir(1)–N(4)	87.97(16)	86.62(15)
N(2)–Ir(1)–N(3)	84.81(17)	85.84(16)
N(2)–Ir(1)–N(4)	101.09(16)	102.04(16)
N(3)–Ir(1)–N(4)	75.55(15)	76.18(15)
C(1)–Ir(1)–N(1)	79.40(19)	80.24(19)
C(1)–Ir(1)–N(2)	92.44(19)	91.05(19)
C(1)–Ir(1)–N(3)	176.50(18)	176.04(17)
C(1)–Ir(1)–N(4)	107.17(17)	106.92(17)
C(14)–Ir(1)–N(1)	92.78(19)	92.3(2)
C(14)–Ir(1)–N(2)	79.6(2)	80.1(2)
C(14)–Ir(1)–N(3)	92.10(19)	95.41(17)
C(14)–Ir(1)–N(4)	167.46(18)	171.03(18)
C(1)–Ir(1)–C(14)	85.3(2)	81.62(19)

Table S3. Short intra- and intermolecular contacts in the crystal structures of **2** and **6**.

D	A[a]	D—H [Å]	H•••A [Å]	D•••A [Å]	D—H•••A [°]
			**2**		
C(12)	N(3)	0.93	2.54	3.318(8)	141.0
C(25)	N(4)	0.93	2.53	3.305(8)	141.0
C(45)	F(4a)	0.93	2.53	3.311(13)	142.0
			**6**		
O(1)	N(5)	0.82	2.32	2.961(11)	136.0
C(2)	N(2)	0.93	2.61	3.090(8)	113.0
C(12)	N(3)	0.93	2.60	3.365(8)	140.0
C(25)	N(4)	0.93	2.53	3.328(8)	144.0
C(30)	F(4b)	0.93	2.45	3.367(7)	168.0
C(43)	F(4b)	0.93	2.53	3.458(9)	174.0

[a]Symmetry code: (a) = x, 3/2-y, -1/2+z; (b) = -x, 1-y, 1-z.

Table S4. Short π•••π interactions (with Cg•••Cg distances shorter than 4 Å) in the crystal structures of **2** and **6**.

Cg(I)•••Cg(J)	Cg(I)•••Cg(J) [Å]	α[°]	β[°]	γ [°]	Cg(I)-Perp [Å]	Cg(J)-Perp [Å]
			**2**			
Cg(8)•••Cg(9)	3.474(4)	9.7(3)	21.11	17.81	-3.308(3)	3.241(2)
			**6**			
Cg(5)•••Cg(12)[a]	3.930(4)	1.9(3)	24.87	25.87	-3.537(2)	-3.566(3)
Cg(8)•••Cg(9)	3.452(4)	13.0(3)	14.35	15.43	3.328(3)	-3.344(3)
Cg(10)•••Cg(10)[b]	3.938(4)	0	25.46	25.46	-3.556(3)	-3.556(3)
Cg(12)•••Cg(12)[a]	3.979 (4)	0	26.30	26.30	-3.568(3)	-3.567(3)

Symmetry code (a) -x,2-y,1-z; (b) -x,1-y,-z;

α = dihedral angle between *Cg*(I) and *Cg*(J);

β = angle *Cg*(I)→*Cg*(J) vector and normal to ring I;

γ = angle between the *Cg*(I) →*Cg*(J) vector and the normal to plane J;

Cg(I)-Perp = Perpendicular distance of *Cg*(I) on ring J;

[f]*Cg*(J)-Perp = perpendicular distance of *Cg*(J) on ring I

Cg(5) is the centroid of the S(2)/C(20)/N(2)/C(26)/C(21); Cg(8) is the centroid of the N(5)/C(37)/C(38)/C(39)/C(40)/C(41) set of atoms; Cg(9) is the centroid of the C(1)/C(2)/C(3)/C(4)/C(5)/C(6) set of atoms; Cg(10) is the centroid of the C(8)/C(9)/C(10)/C(11)/C(12)/C(13) set of atoms; Cg(12) is the centroid of the C(21)/C(22)/C(23)/C(24)/C(25)/C(26) set of atoms.

Table S5. X–H•••Cg(J)(π-ring) interactions (with with H•••Cg distances shorter than 3 Å and γ < 30°**)** in the crystal structures of **2** and **6**.

**X–H•••Cg(J)**	**H(I)•••Cg(J) [Å]**	**X–Perp [Å]**	**γ [°]**	**X–H(I)•••Cg(J) [°]**
		**2**		
C(15)–H(15)•••Cg(4)	2.91	2.65	24.28	137
C(27)–H(27)•••Cg(11)	2.88	2.55	27.50	144
C(29)–H(29)•••Cg(5)[a]	2.81	2.56	24.02	169
C(29)–H(29)•••Cg(12)[a]	2.78	2.56	23.30	141
		**6**		
C(2)–H(2)•••Cg(5)	2.82	2.61	22.04	135
C(15)–H(15)•••Cg(4)	2.93	-2.64	25.42	135
C(17)–H(17)•••Cg(9)[b]	2.67	-2.57	15.16	179
C(22)–H(22)•••Cg(6)[c]	2.77	2.68	14.38	150

C(24)–H(24)•••Cg(8)[d]	2.62	2.61	6.02	168

Symmetry codes: (a) = -x,1-y,1-z; (b) = -x, 2-y, -z; (c) = -x, 2-y,1-z; (c) = 1-x, 2-y,1-z.
γ = angle X(I)→Cg(J) vector and normal to plane J.
Cg(4) is the centroid of the ring: S(2)/C(7)/N(1)/C(13)/C(8); Cg(5) is the centroid of the ring: S(2)/C(20)/N(2)/C(26)/C(21); Cg(6) is the centroid of the ring: N(3)/C(27)/C(28)/C(29)/C(30)/C(31); Cg(8) is the centroid of the ring: N(5)/C(37)/C(38)/C(39)/C(40)/C(41); Cg(9) is the centroid of the ring: C(1)/C(2)/C(3)/C(4)/C(5)/C(6); Cg(11) is the centroid of the ring: C(14)/C(15)/C(16)/C(17)/C(18)/C(19); Cg(12) is the centroid of the ring: C(21)/C(22)/C(23)/C(24)/C(25)/C(26).

Table S6. Y–X•••Cg(J)(π-ring) interactions (with X•••Cg distances shorter than 4 Å and **γ** < 30°) in the crystal structures of **2** and **6**.

Y–X•••Cg(J)	X•••Cg(J) [Å]	X–Perp [Å]	γ [°]	Y–X(I)•••Cg(J) [°]
**1**				
P(1)–F(4)•••Cg(6)	3.84 (2)	3.430	26.75	132.1(9)
P(1)–F(3)•••Cg(12)	3.192(18)	-3.164	7.55	161.1(11)
**6**				
not detected				

γ = angle Y(I)→Cg(J) vector and normal to plane J.
Cg(6) is the centroid of atoms = N(3)/C(27)/C(28)/C(29)/C(30)/C(31); Cg(12) is the centroid of the ring: C(21)/C(22)/C(23)/C(24)/C(25)/C(26).

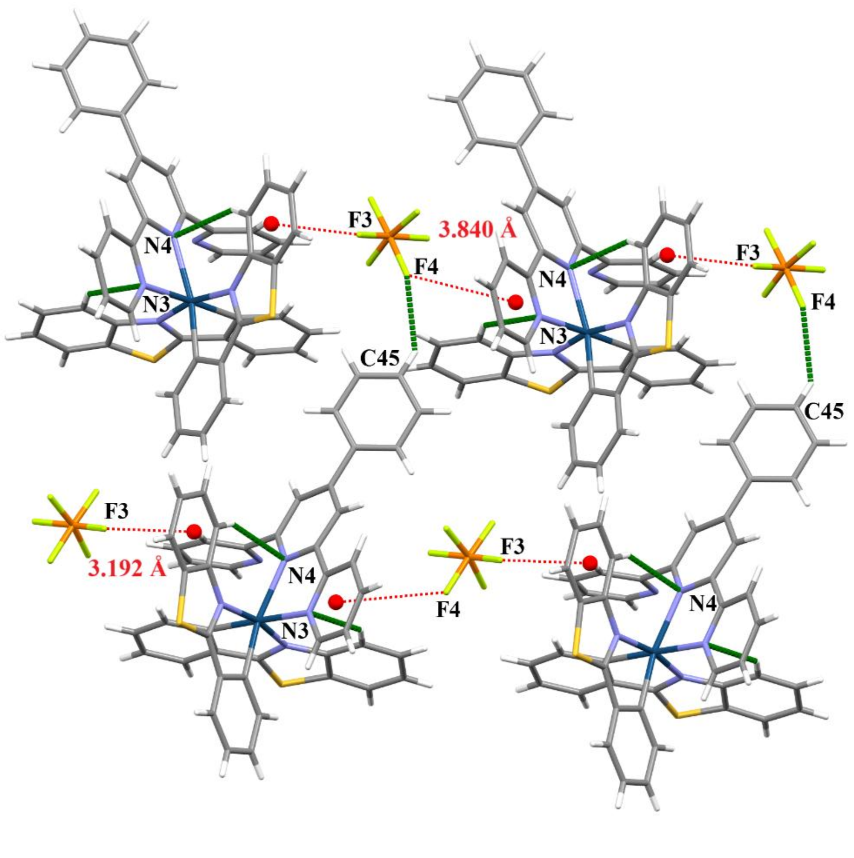

(a)

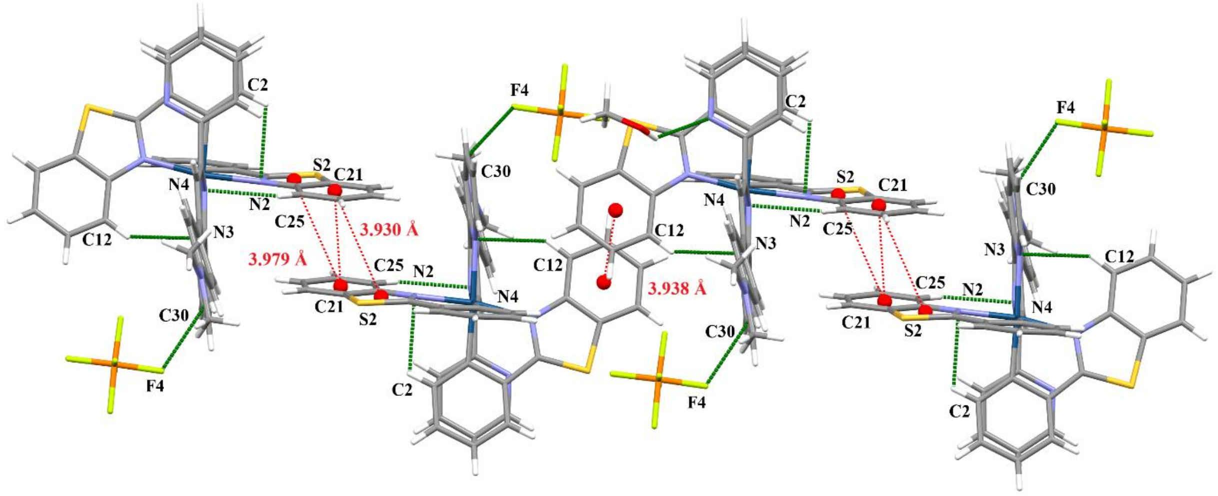

(b)

Figure S4. Crystal packing arrangement of **2** (a) and **6** (b) along with the selected intermolecular contacts.

Electrochemical charcterization

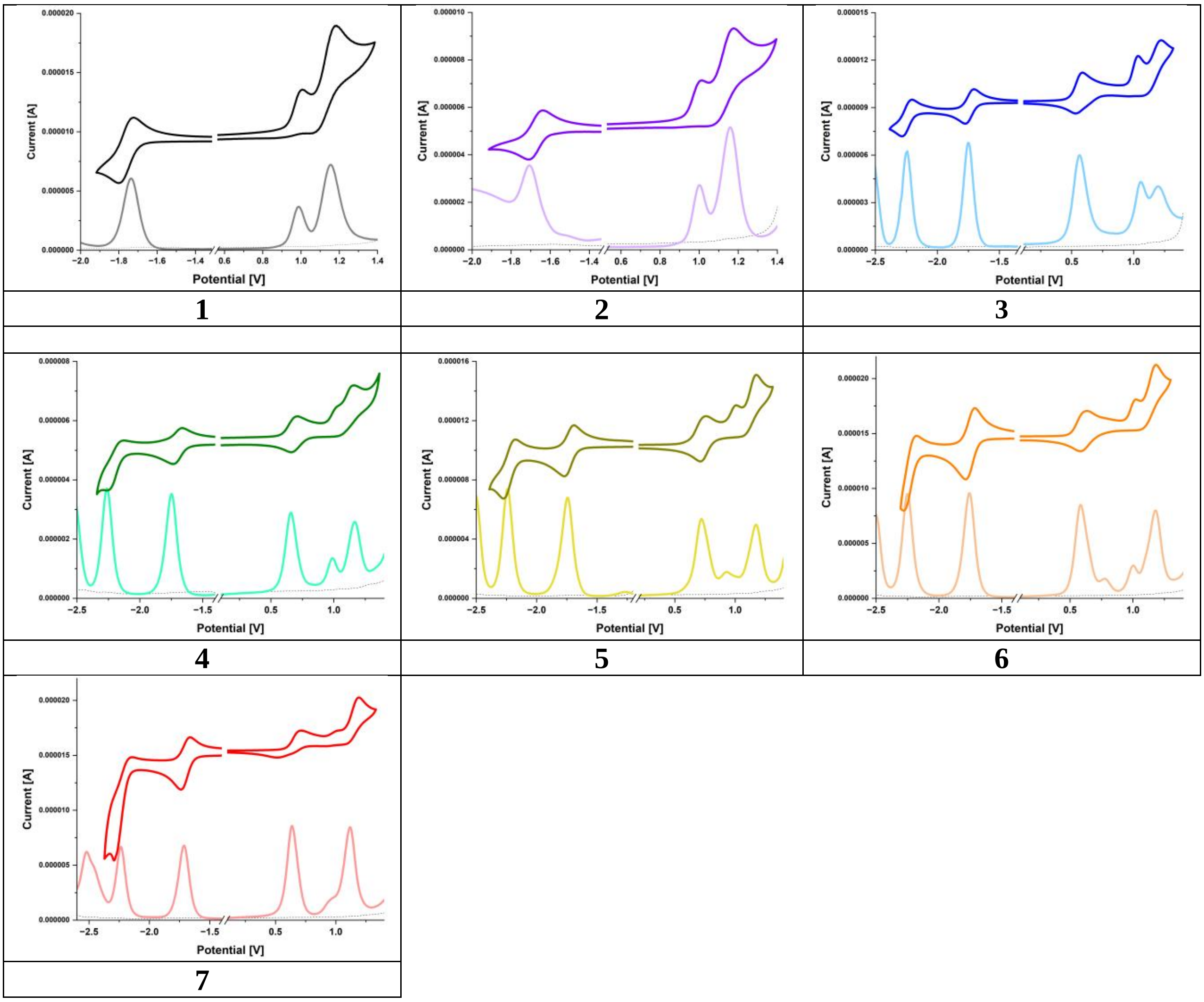

Figure S5. The cyclic voltammetry (CV) and differential pulse voltammetry (DPV) curves of complexes **1–7**.

UV-Vis spectroscopy

Table S7. The absorption maxima and molar extinction coefficient values for complexes **1–7**.

**Compound**	**Medium**	**λ/nm (10^4 ε/ $dm^3 \cdot mol^{-1} \cdot cm^{-1}$)**
**1**	$CHCl_3$	440.6 (0.63), 411.8 (0.81), 377.4 (1.04), 353.2 (1.73), 316.0 (4.86), 272.8 (3.69), 252.8 (4.25)
	CH_3CN	440.6 (0.47), 412.7 (0.64), 374.0 (0.76), 348.3 (1.26), 314.6 (3.25), 269.4 (2.85), 247.2 (3.04)
**2**	$CHCl_3$	440.9 (0.44), 408.9 (0.57), 370.5 (0.90), 344.7 (1.71), 316.9 (3.91), 280.8 (3.87)
	CH_3CN	439.4 (0.56), 408.4 (0.78), 371.0 (1.20), 343.9 (2.15), 316.2 (4.96), 276.4 (5.43)
**3**	$CHCl_3$	423.66 (2.41), 377.2 (1.97), 352.8 (2.06), 315.1 (4.04), 263.5 (4.20), 249.4 (4.40)

	CH_3CN	409.0 (2.94), 382.3 (2.68), 354.2 (2.58), 313.4 (4.65), 254.4 (4.52)
**4**	$CHCl_3$	407.8 (4.08), 387.7 (3.91), 351.4 (3.73), 315.8 (6.72), 260.2 (6.16), 251.2 (6.60)
	CH_3CN	382.8 (3.85), 352.6 (3.75), 312.8 (6.06), 251.0 (5.82)
**5**	$CHCl_3$	412.2 (3.68), 355.8 (2.97), 314.3 (5.56), 275.6 (5.05), 249.6 (6.11)
	CH_3CN	406.3 (2.91), 349.1 (2.46), 313.5 (4.23), 252.5 (4.12)
**6**	$CHCl_3$	429.8 (2.07), 380.6 (1.57), 355.0 (1.72), 315.4 (3.49), 269.2 (3.18), 246.5 (3.47)
	CH_3CN	414.4 (2.33), 358.7 (1.80), 344.6 (1.80), 313.4 (3.30), 253.5 (3.07)
**7**	$CHCl_3$	441.5 (3.12), 375.5 (1.88), 346.2 (2.35), 315.4 (5.05), 288.3 (4.89), 265.5 (4.97)
	CH_3CN	416.6 (3.25), 353.5 (2.27), 313.9 (4.82), 277.3 (4.63)

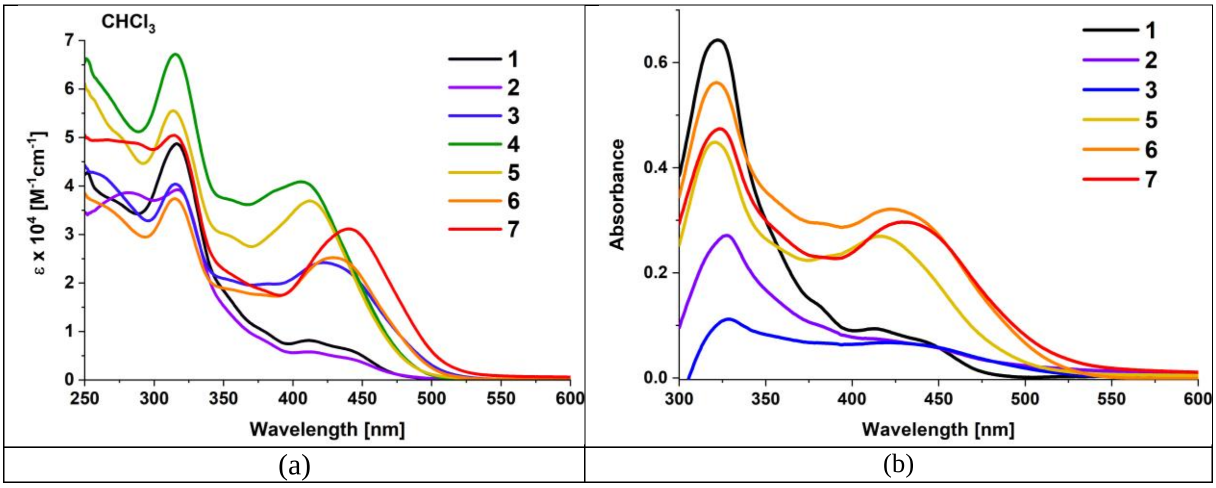

(a) (b)

Figure S6. UV-Vis spectra of **1**–**7** in chloroform solution (a) solid state as film on glass substrate (b).

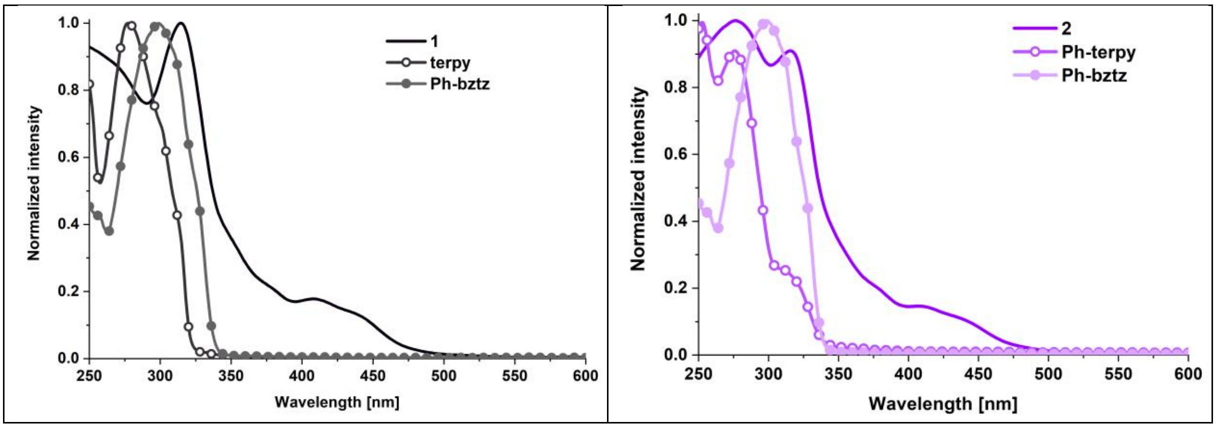

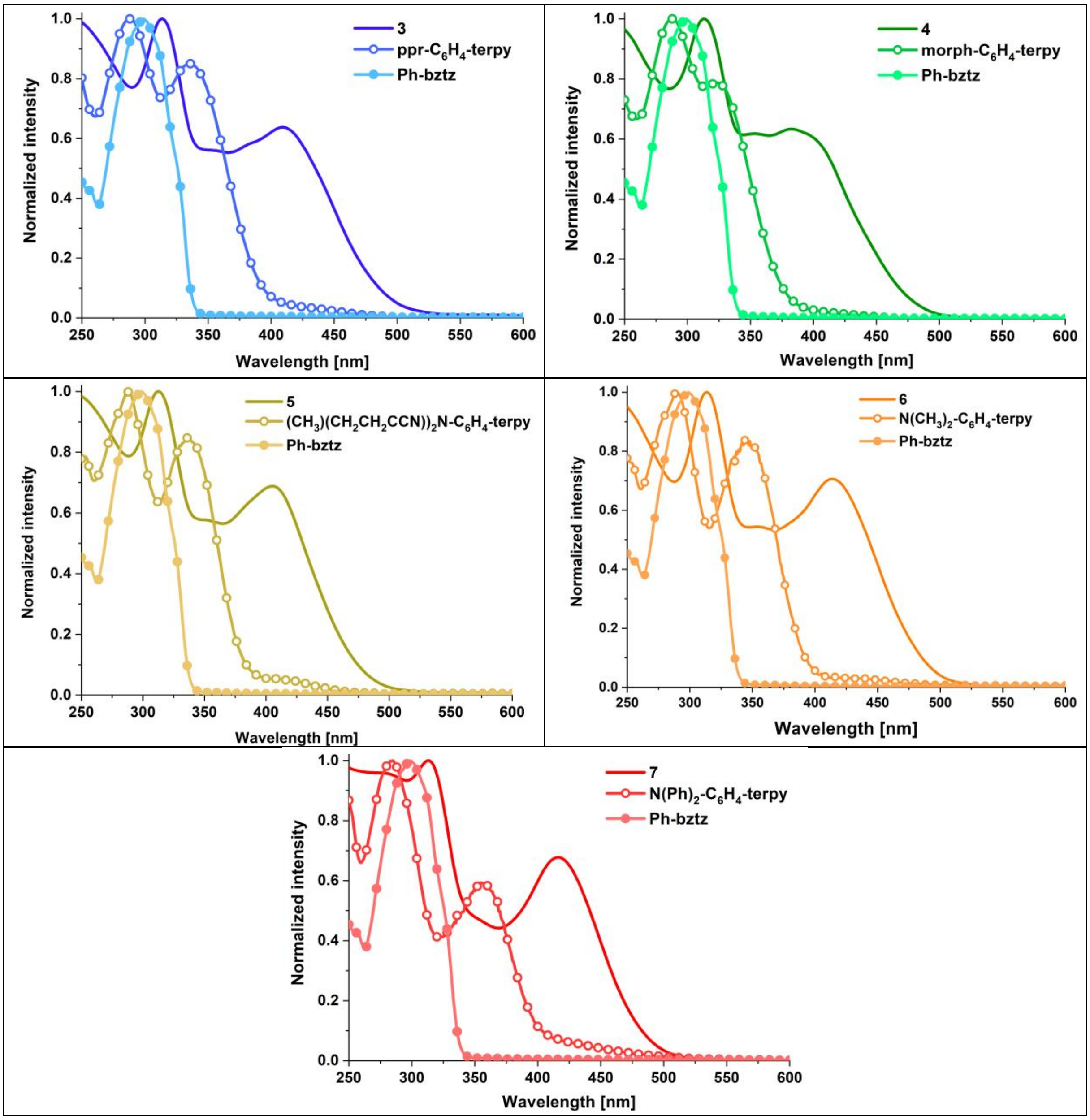

Figure S7. UV-Vis spectra of **1–7** in CH_3CN alongside electronic absorption spectra of 2-phenylbenzothiazole and the appropriate terpy derivative.

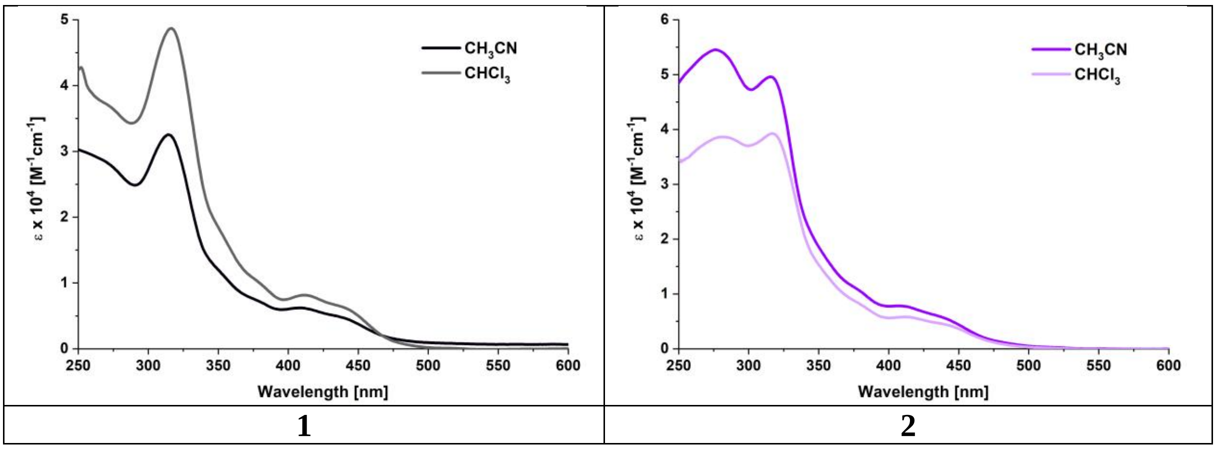

1	2

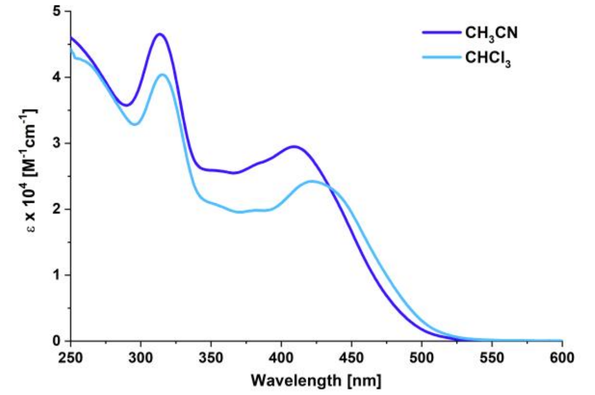

3

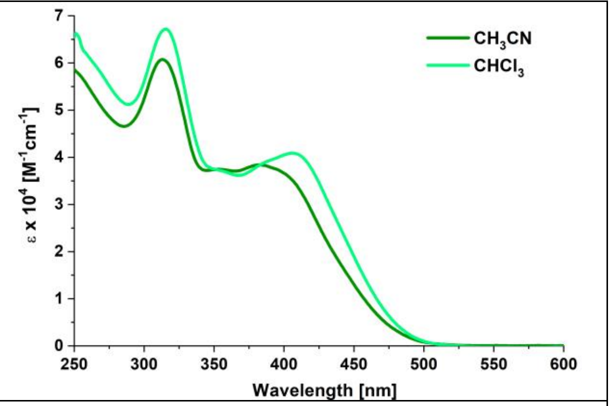

4

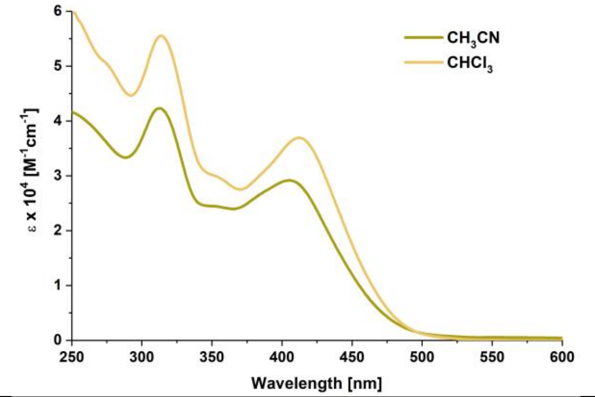

5

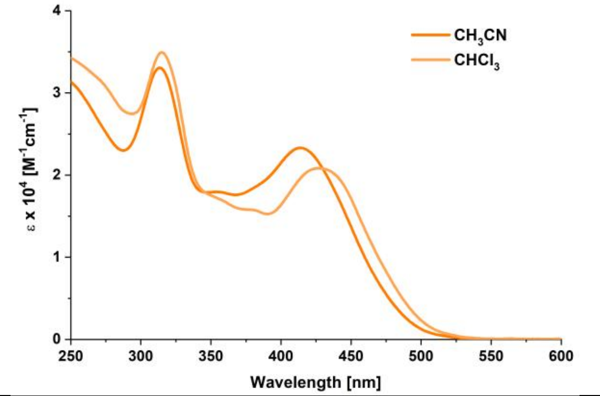

6

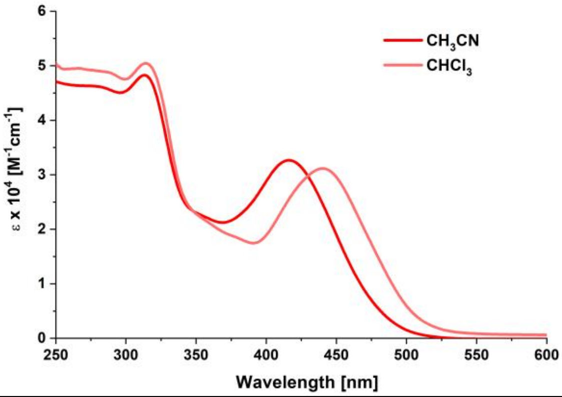

7

Figure S8. Solvatochromic impact on the absorbance behavior of **1**–**7**.

Compound	Stability	Fotostability
**1**	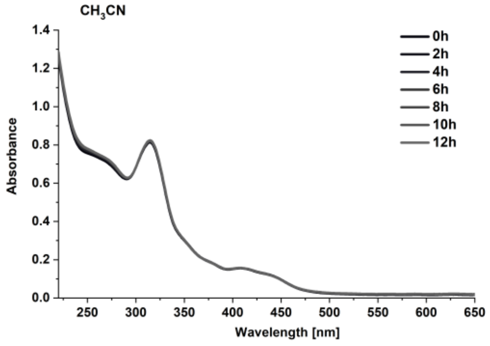	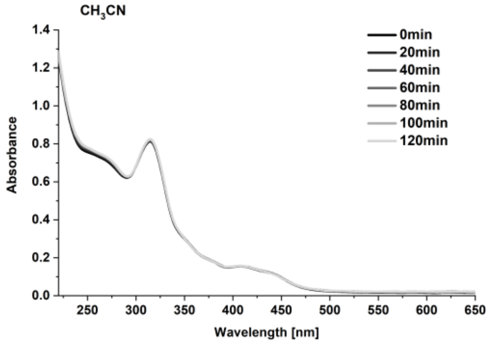

<table>
<tr>
<td></td>
<td>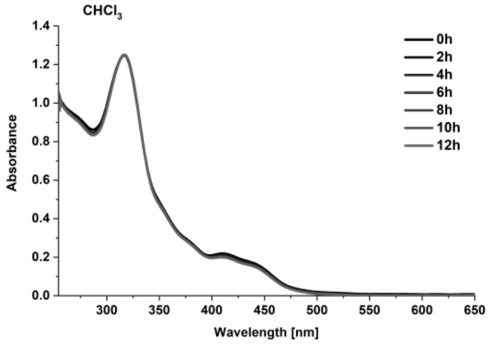
</td>
<td>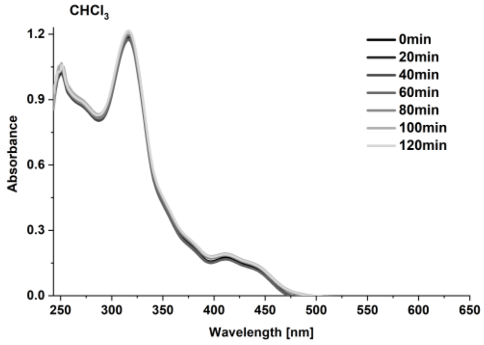
</td>
</tr>
<tr>
<td>2</td>
<td>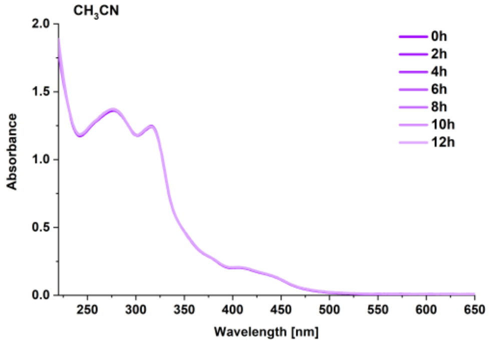

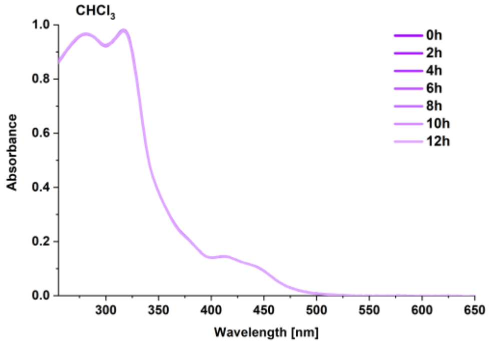
</td>
<td>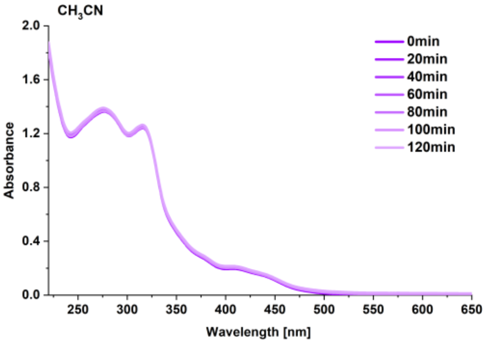
</td>
</tr>
<tr>
<td>3</td>
<td>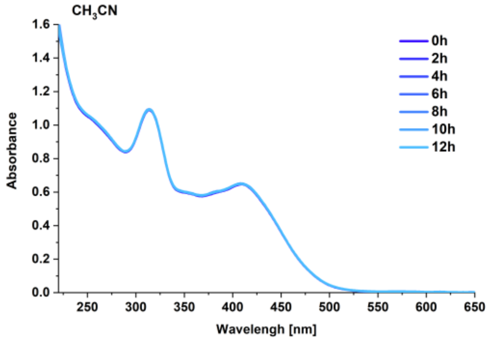

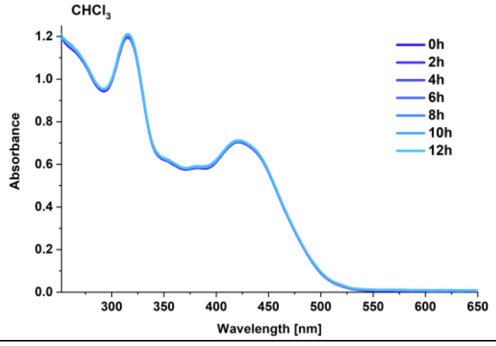
</td>
<td>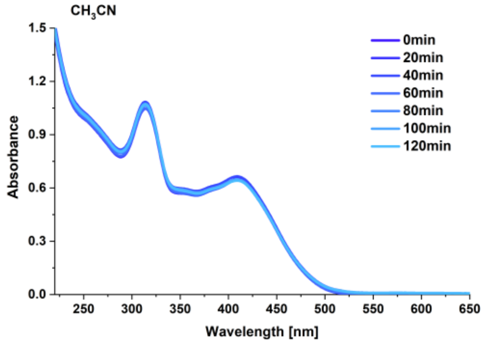
</td>
</tr>
</table>

4

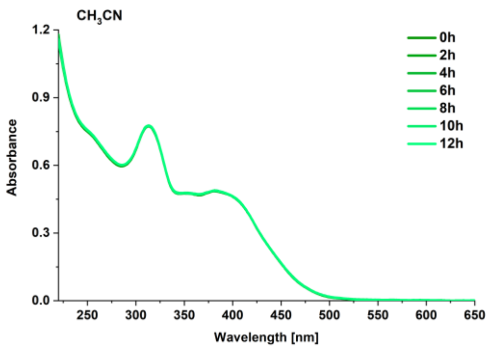
CH_3CN
Absorbance
Wavelength [nm]
0h
2h
4h
6h
8h
10h
12h

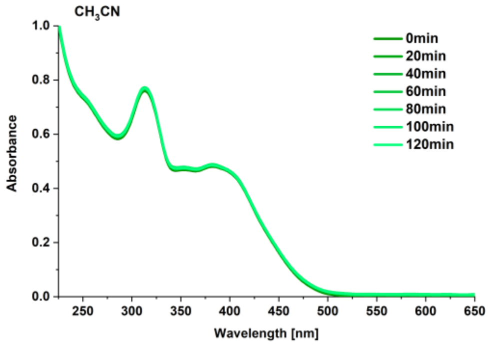
CH_3CN
Absorbance
Wavelength [nm]
0min
20min
40min
60min
80min
100min
120min

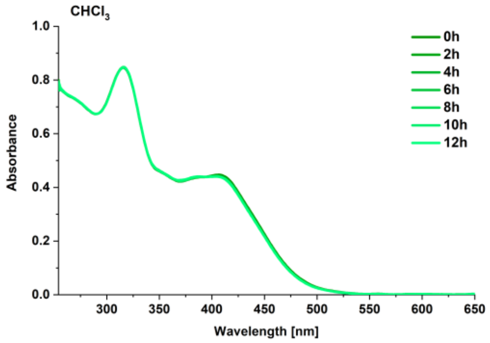
$CHCl_3$
Absorbance
Wavelength [nm]
0h
2h
4h
6h
8h
10h
12h

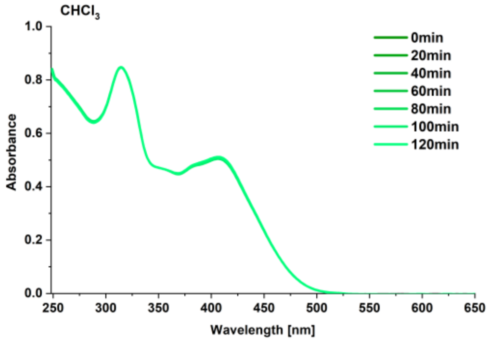
$CHCl_3$
Absorbance
Wavelength [nm]
0min
20min
40min
60min
80min
100min
120min

5

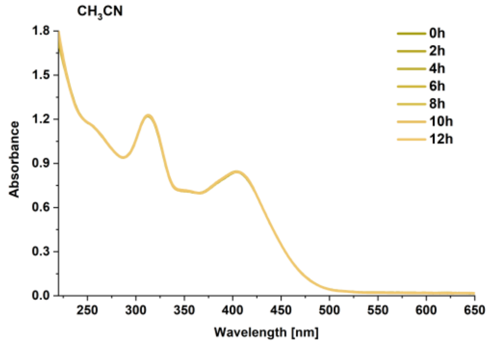
CH_3CN
Absorbance
Wavelength [nm]
0h
2h
4h
6h
8h
10h
12h

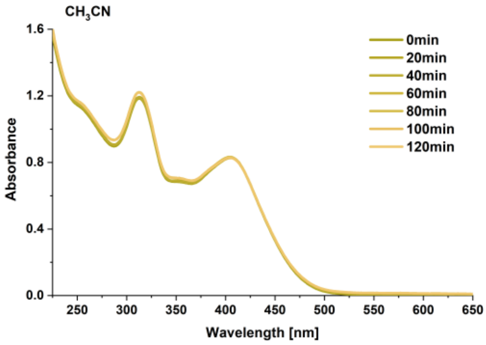
CH_3CN
Absorbance
Wavelength [nm]
0min
20min
40min
60min
80min
100min
120min

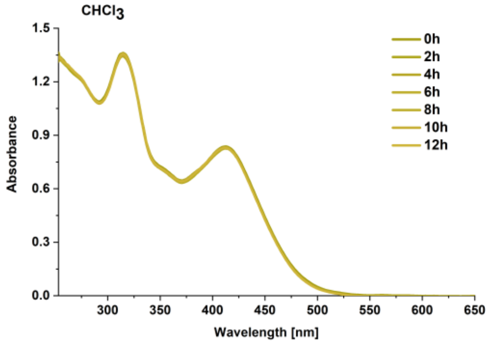
$CHCl_3$
Absorbance
Wavelength [nm]
0h
2h
4h
6h
8h
10h
12h

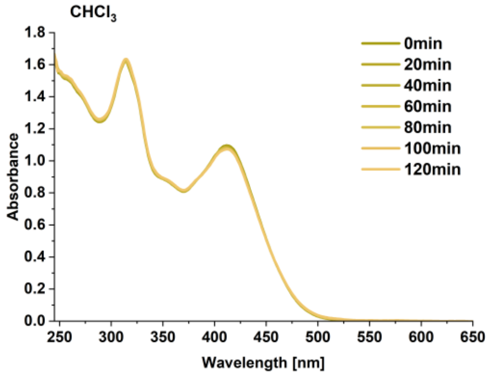
$CHCl_3$
Absorbance
Wavelength [nm]
0min
20min
40min
60min
80min
100min
120min

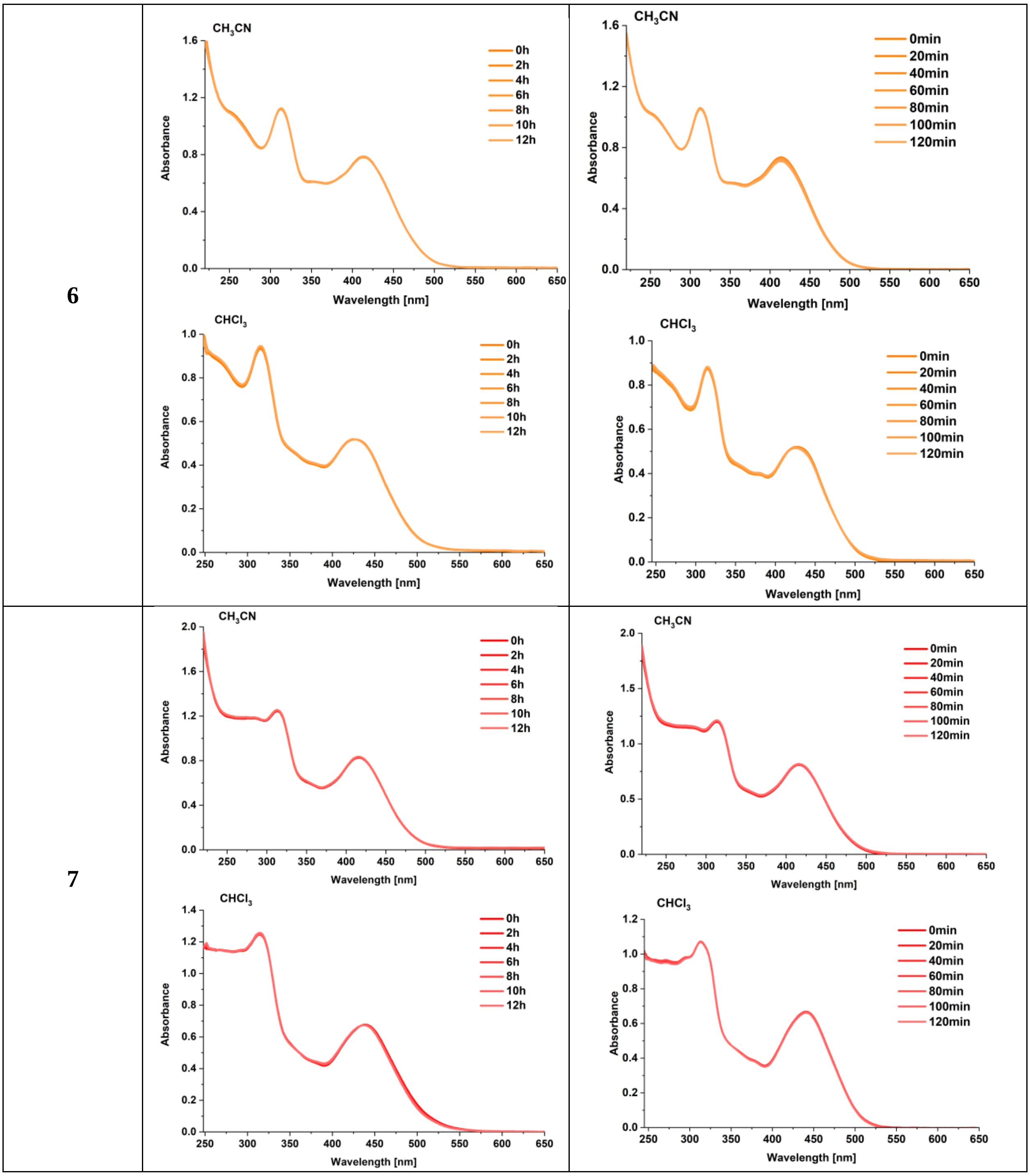

Figure S9. UV-Vis photostability of Ir(III) complexes upon 420 nm light irradiation in CH_3CN and $CHCl_3$; $c = 10^{-5}$ M.

Luminescence properties

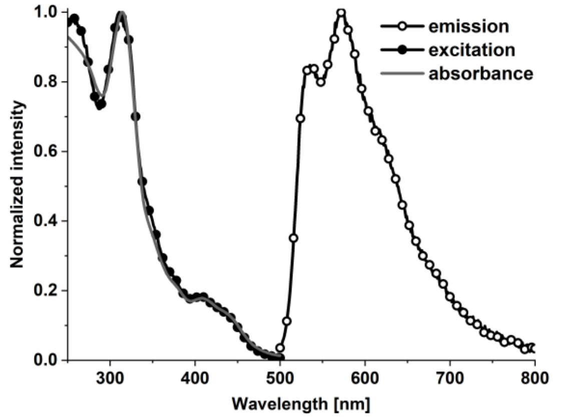

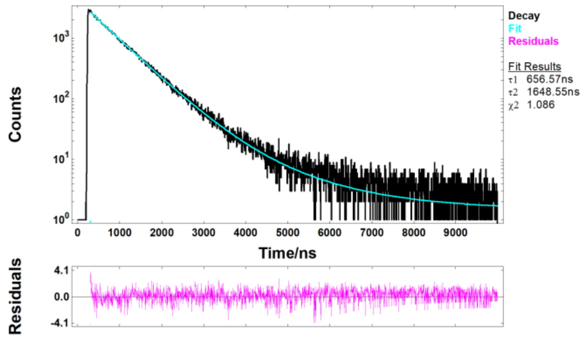

Fit results: τ_1 = 656.57 ns ± 4.40 ns (93.61%)
τ_2 = 1648.55 ns ± 148.60 ns (6.39%)

CH_3CN

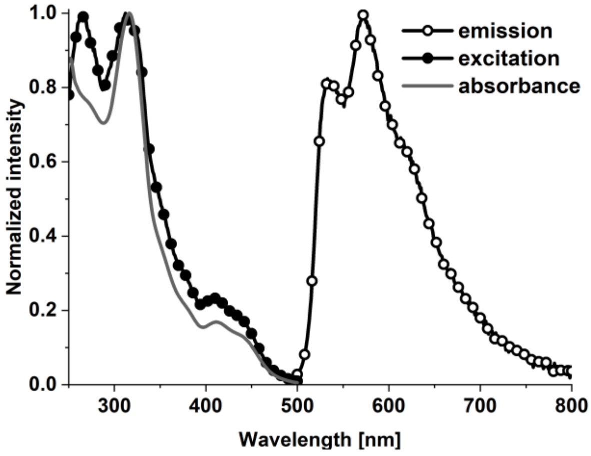

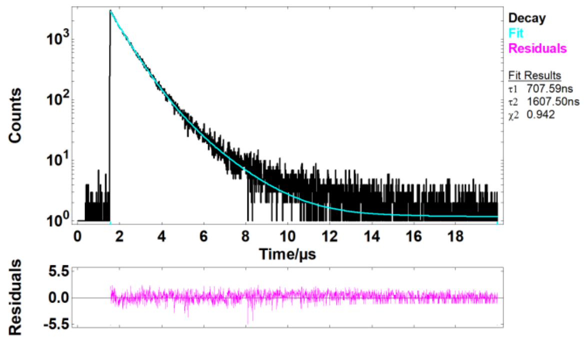

Fit results: τ_1 = 707.59 ns ± 7.17 ns (79.01%)
τ_2 = 1607.50 ns ± 44.34 ns (20.99%)

$CHCl_3$

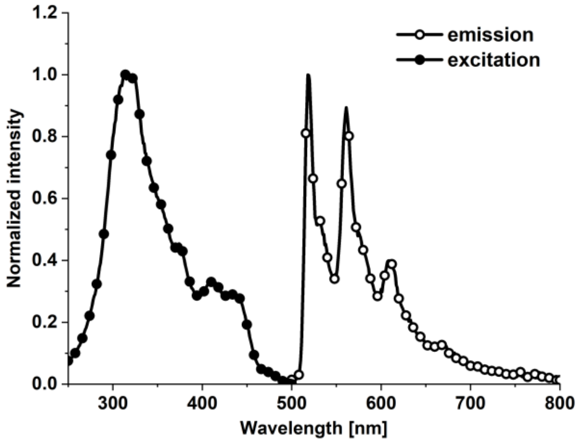

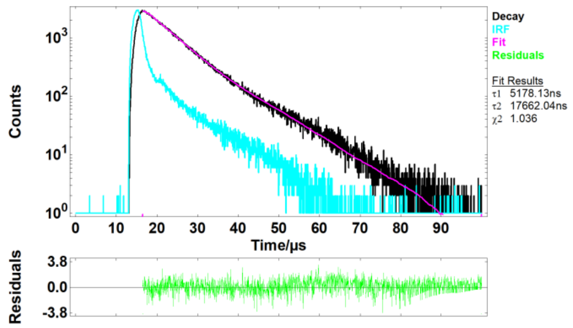

Fit results: τ_1 = 5.18 µs ± 0.04 µs (94.31%)
τ_2 = 17.66 µs ± 1.45 µs (5.69%)

77 K (EtOH:MeOH 4:1 v/v)

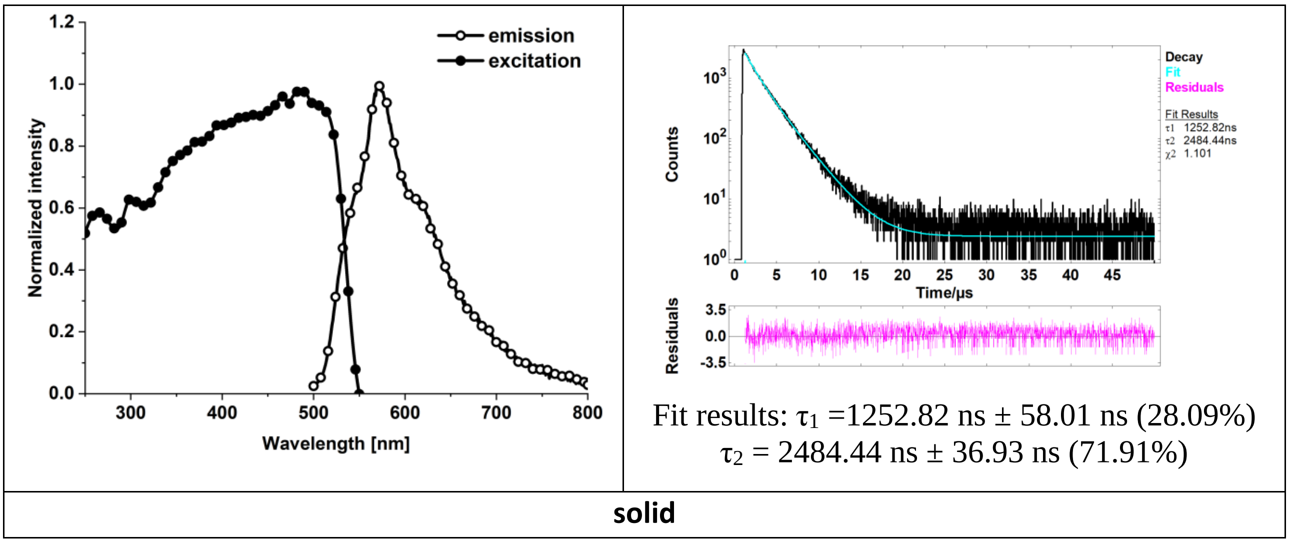

Figure S10. Summary of photoluminescence properties of complex **1**.

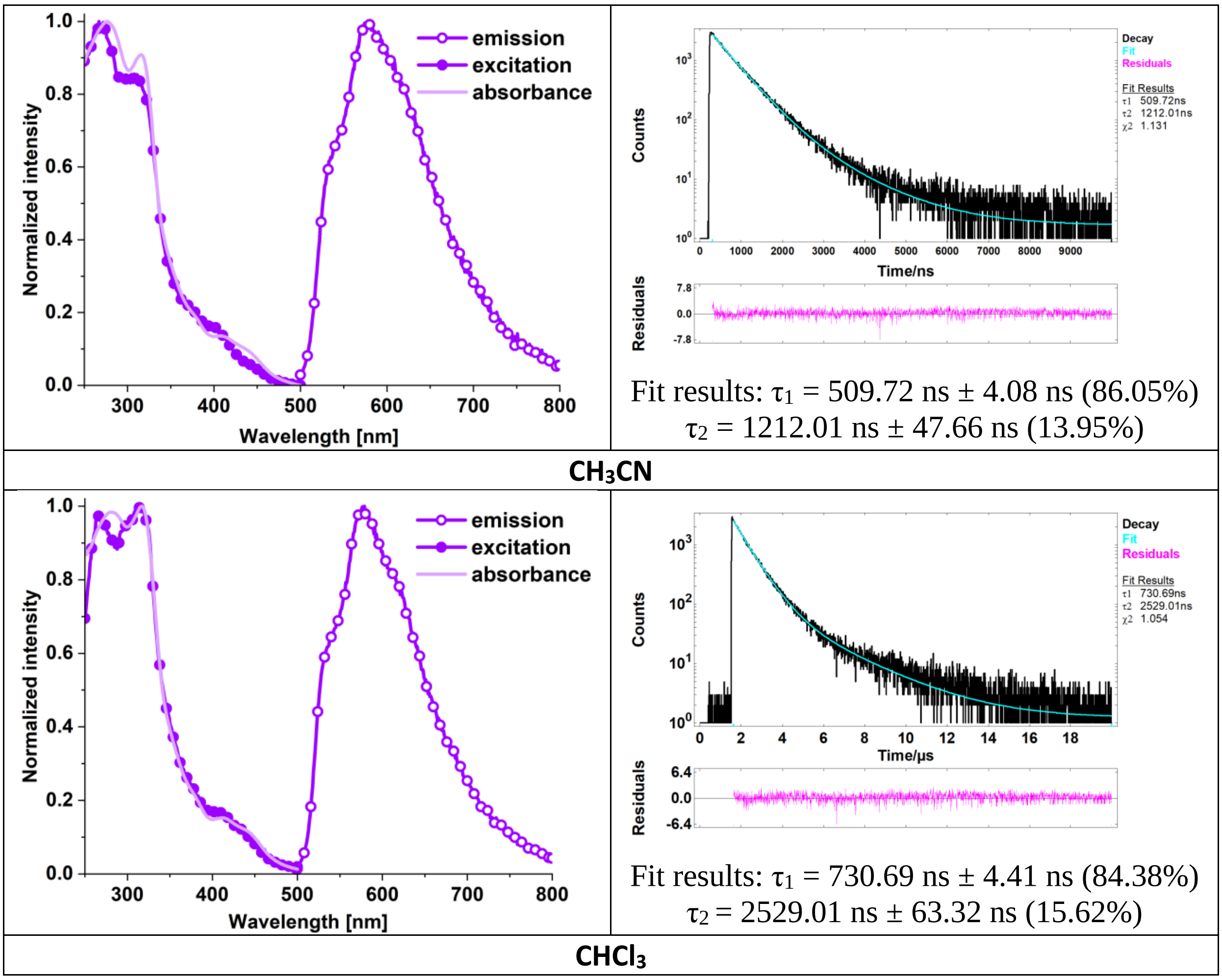

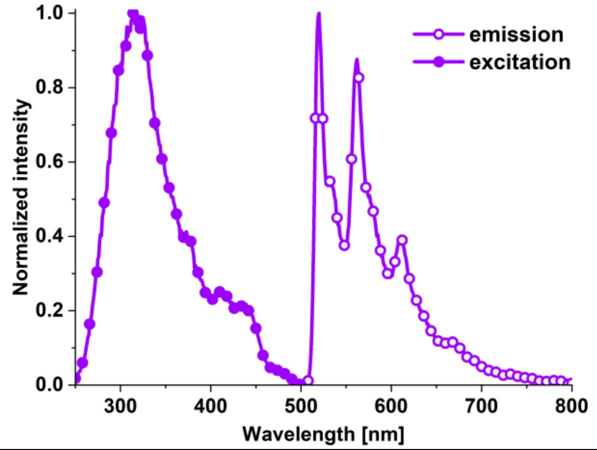

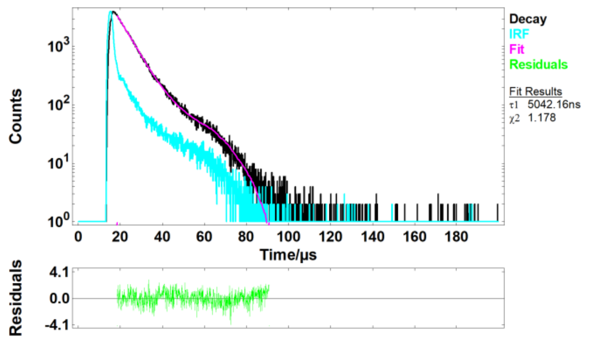

Fit results: τ_1 = 5042.16 ns ± 18.03 ns (100%)

77 K (EtOH:MeOH 4:1 v/v)

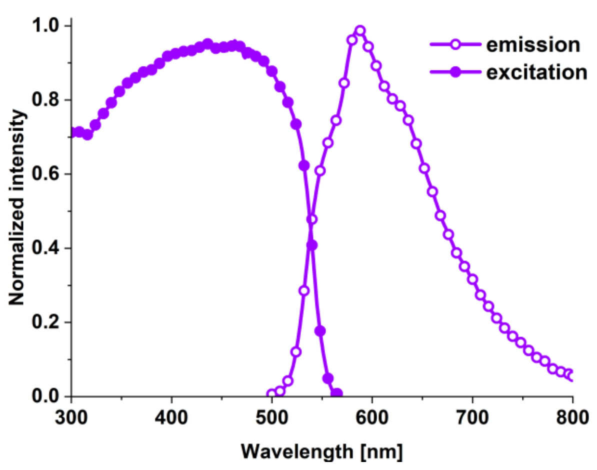

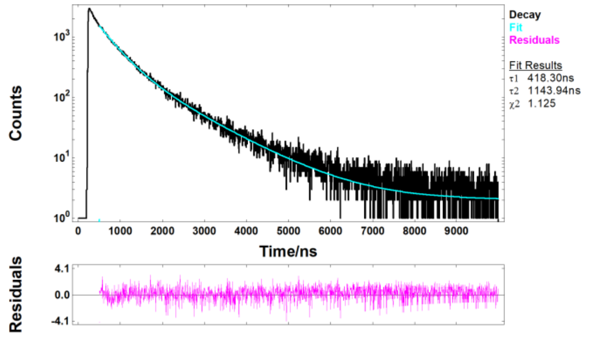

Fit results: τ_1 = 418.30 ns ± 6.74 ns (51.19%)
τ_2 = 1143.94 ns ± 17.42 ns (48.81%)

solid

Figure S11. Summary of photoluminescence properties of complex **2**.

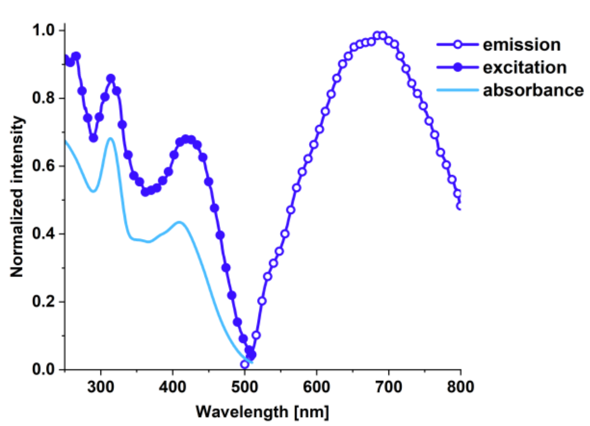

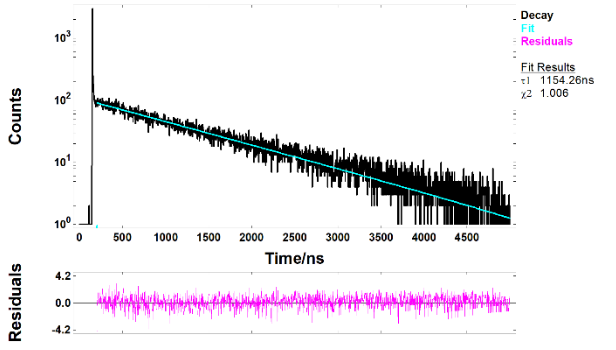

Fit results: τ_1 = 1.15 μs ± 0.01 μs

CH_3CN

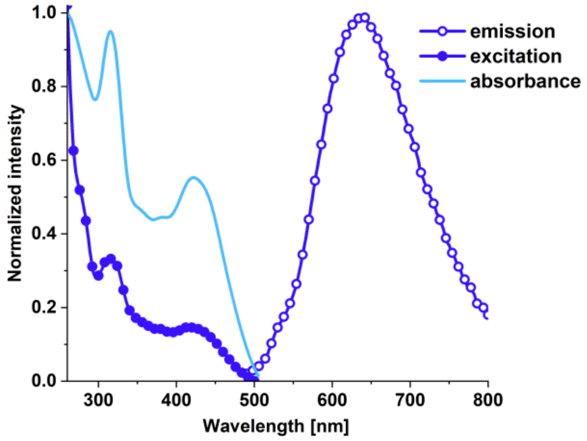

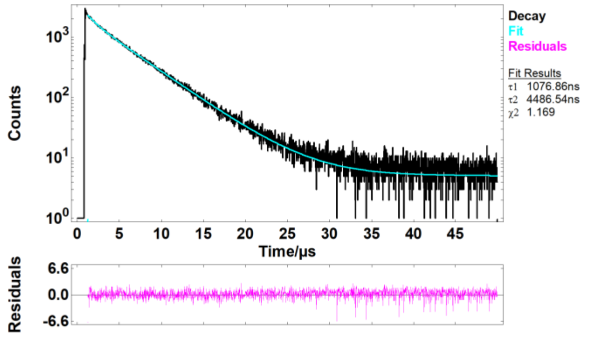

Fit results: τ_1 = 1076.86 ns ± 73.29 ns (5.23%)
τ_2 = 4486.54 ns ± 16.98 ns (94.77%)

$CHCl_3$

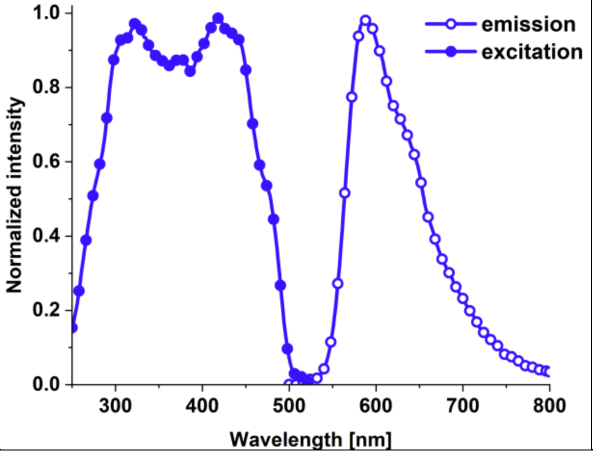

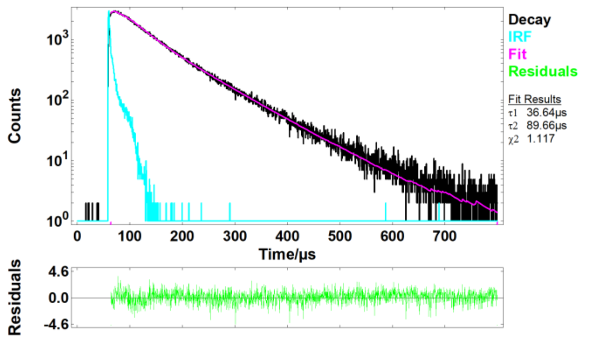

Fit results: τ_1 = 36.64 μs ± 0.88 μs (33.39%)
τ_2 = 89.66 ± 0.70 μs (66.61%)

77 K (EtOH:MeOH 4:1 v/v)

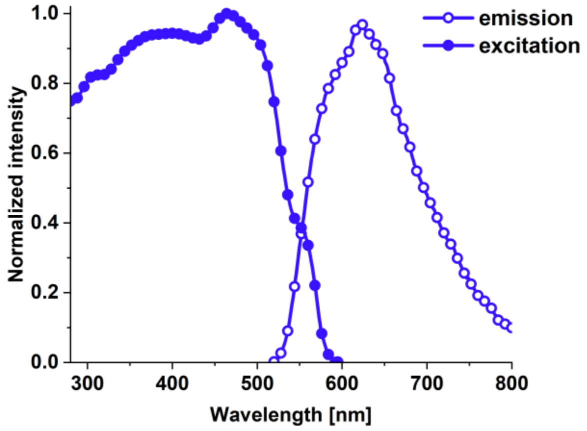

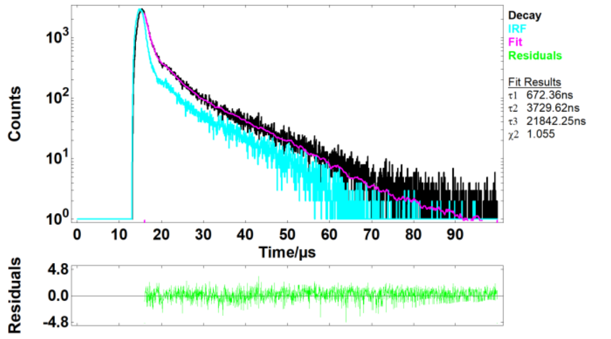

Fit results: τ_1 = 0.67 μs ± 0.02 μs (82.82%)
τ_2 = 3.723 μs ± 0.25 μs (13.81%)
τ_3 = 21.84 μs ± 3.51 μs (3.37%)

solid

Figure S12. Summary of photoluminescence properties of complex **3**.

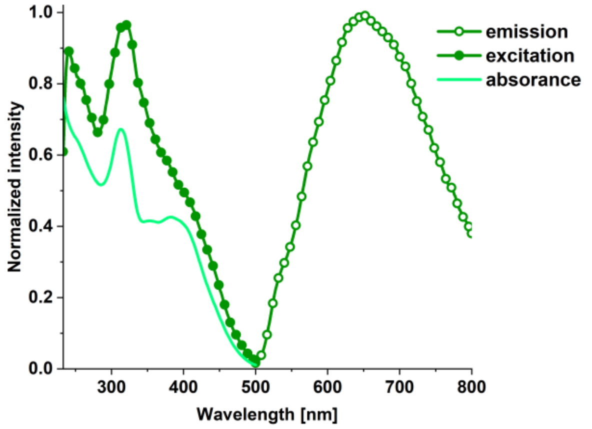

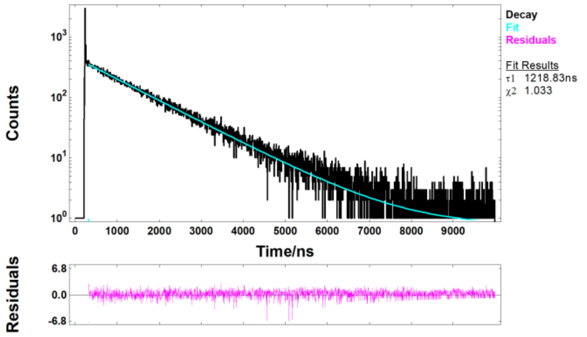

Fit results: τ_1 = 1218.83 ns ± 5.15 ns (100%)

CH_3CN

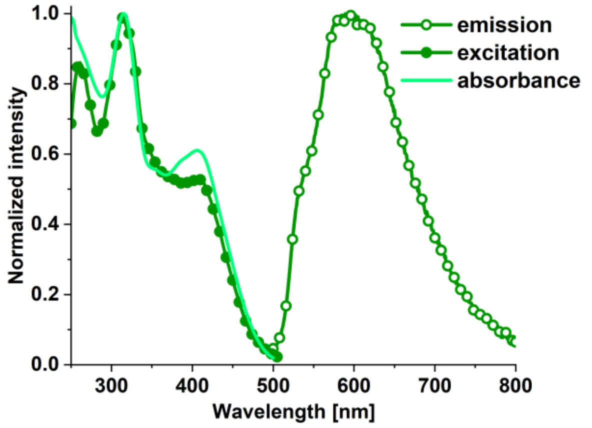

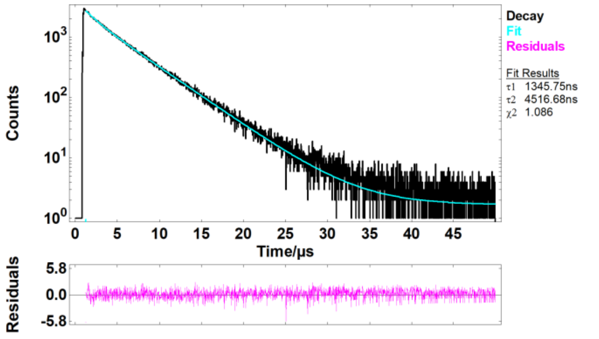

Fit results: τ_1 = 1345.75 ns ± 82.31 ns (6.54%) τ_2 = 4516.68 ns ± 17.36 ns (93.46%)

$CHCl_3$

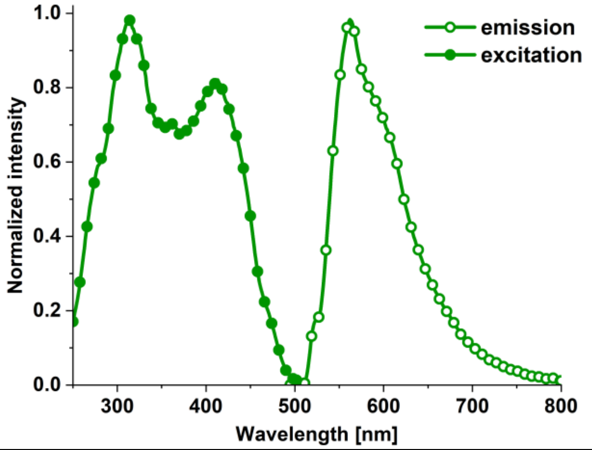

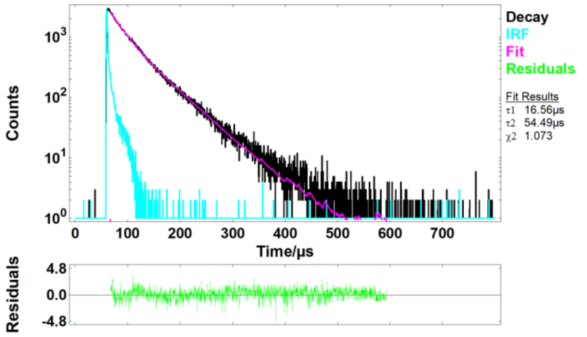

Fit results: τ_1 = 16.56 μs ± 0.39 μs (35.86%) τ_2 = 54.49 μs ± 0.38 μs (64.14%)

77 K (EtOH:MeOH 4:1 v/v)

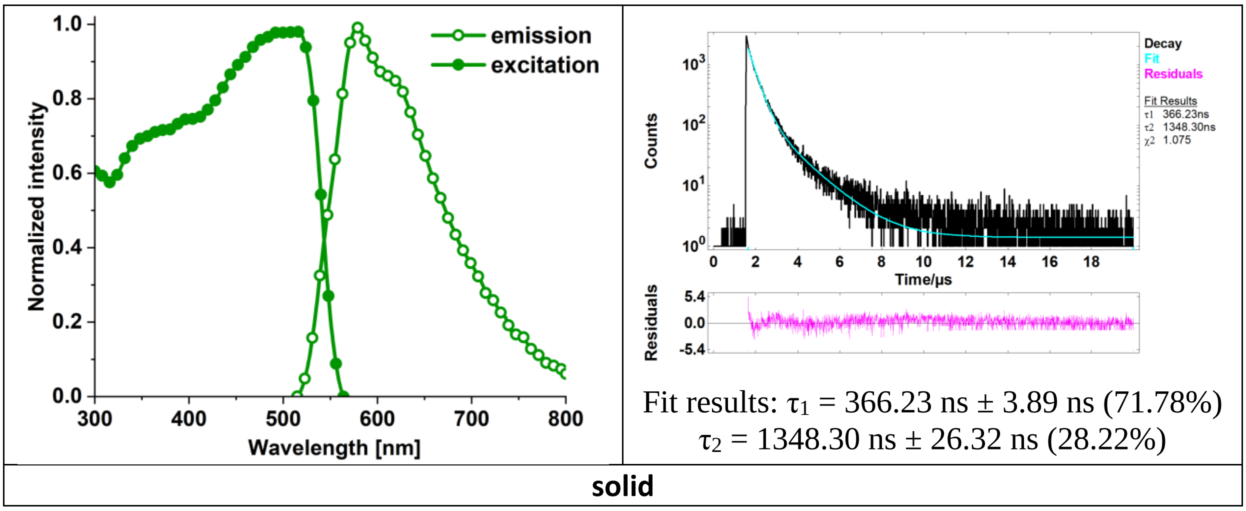

Figure S13. Summary of photoluminescence properties of complex **4**.

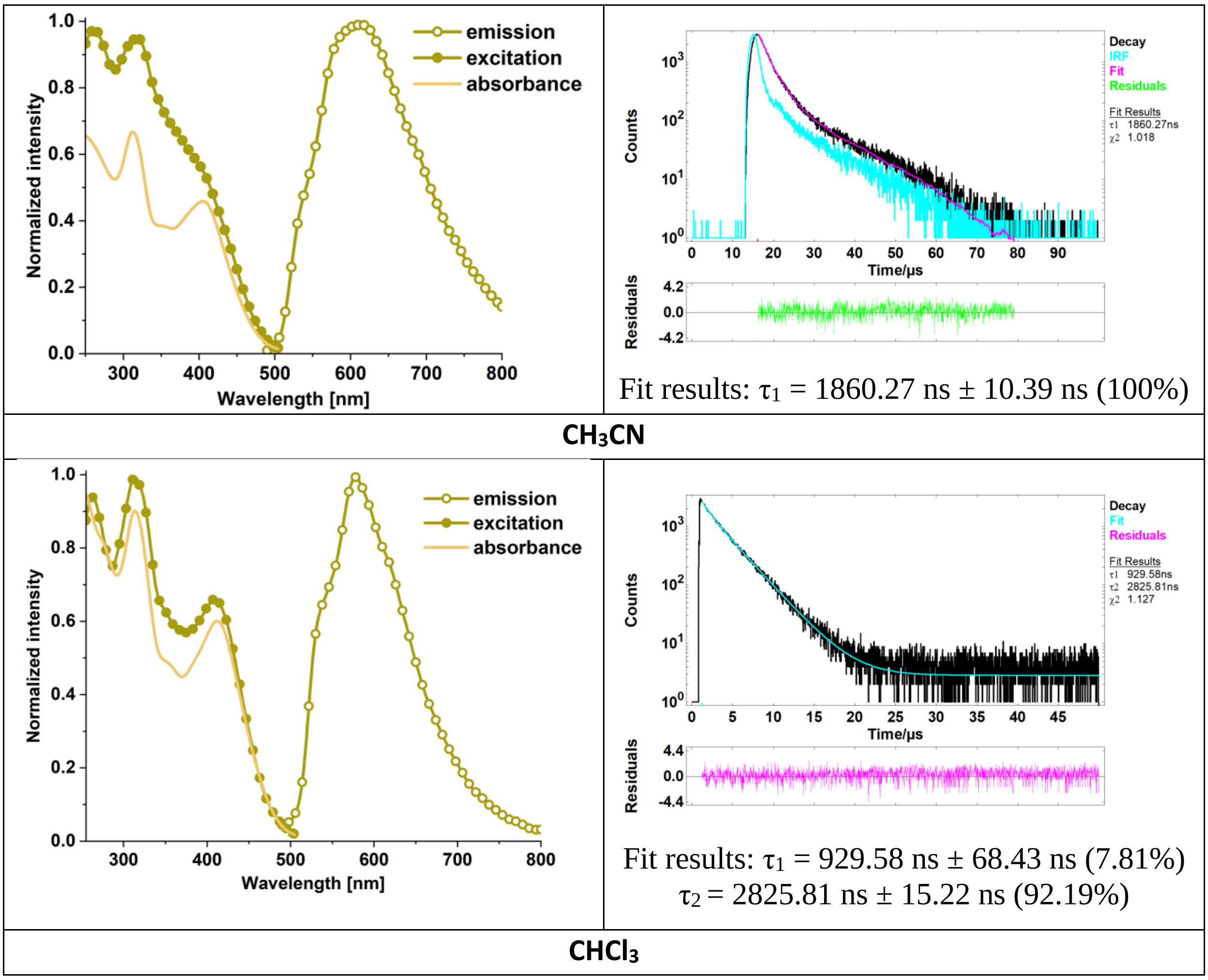

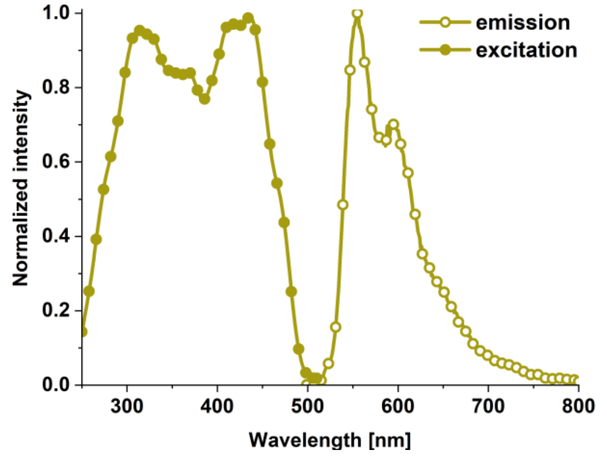

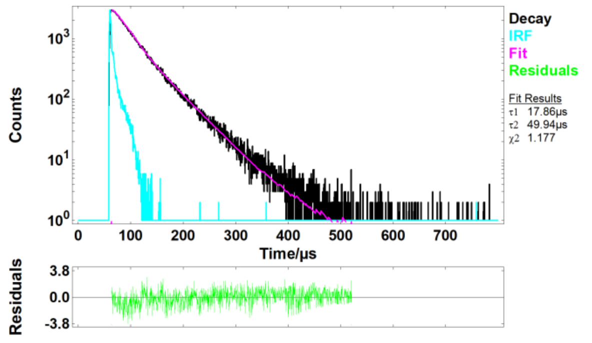

Fit results: τ_1 = 17.86 μs ± 0.44 μs (41.50%) τ_1 = 49.94 μs ± 0.43 μs (58.50%)

77 K (EtOH:MeOH 4:1 v/v)

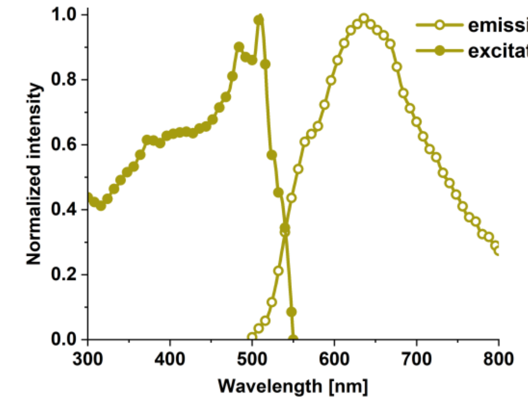

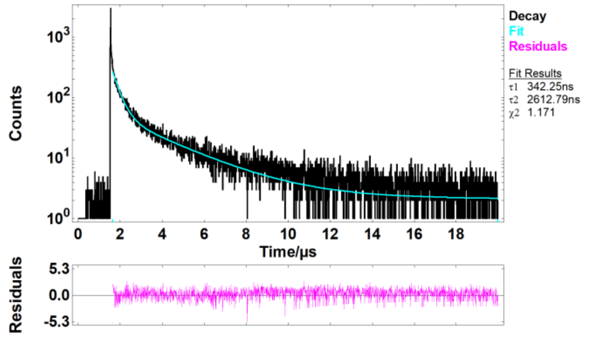

Fit results: τ_1 = 342.25 ns ± 10.36 ns (37.13%) τ_2 = 2612.79 ns ± 64.33 ns (62.87%)

solid

Figure S14. Summary of photoluminescence properties of complex **5**.

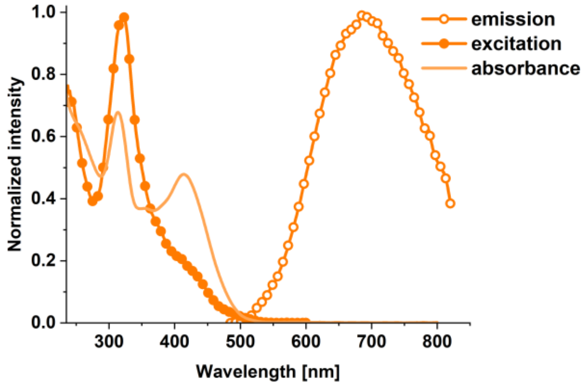

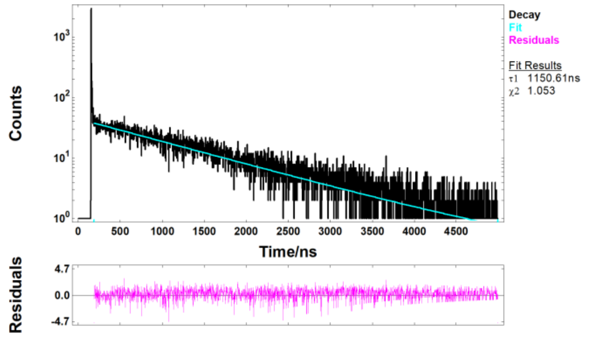

Fit results: τ_1 = 1150.61 ns ± 19.81 ns (100%)

CH_3CN

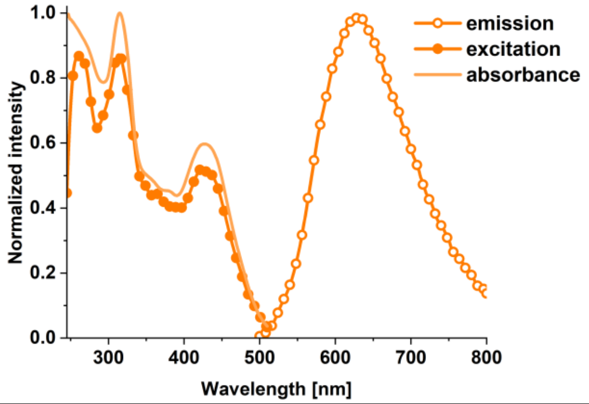

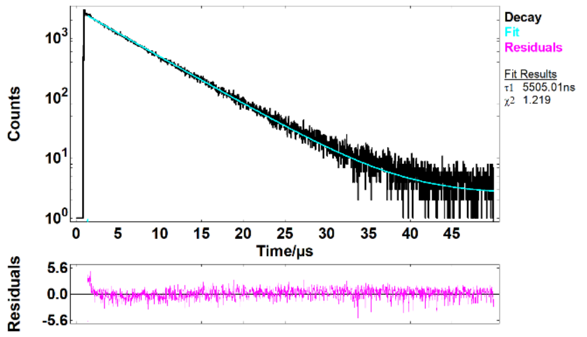

Fit results: τ_1 = 5505.01 ns ± 8.72 ns (100%)

$CHCl_3$

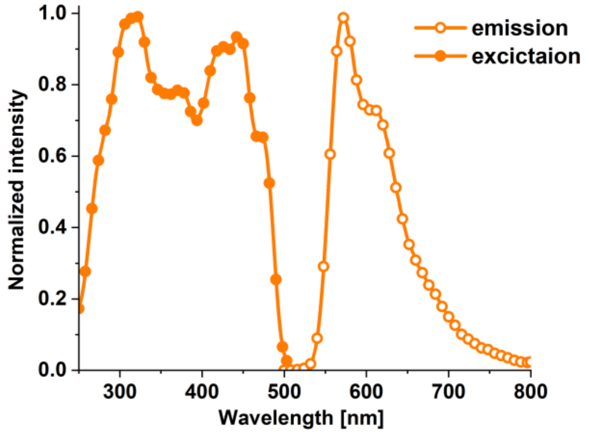

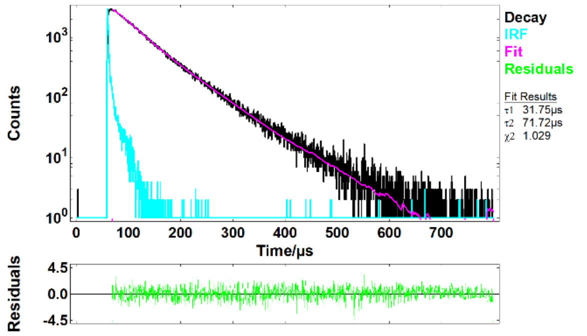

Fit results: τ_1 = 31.75 μs ± 0.91 μs (29.95%)
τ_2 = 71.72 μs ± 0.60 μs (70.05%)

77 K (EtOH:MeOH 4:1 v/v)

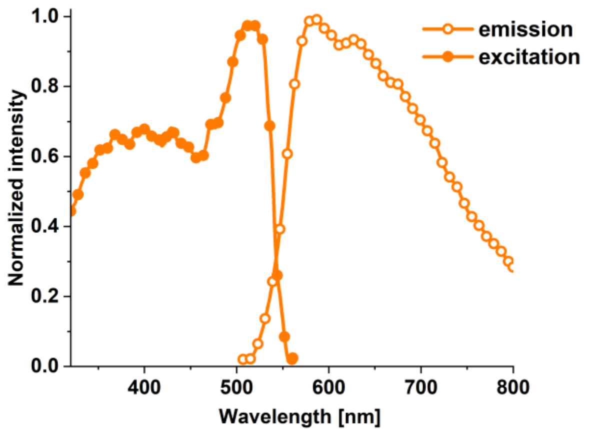

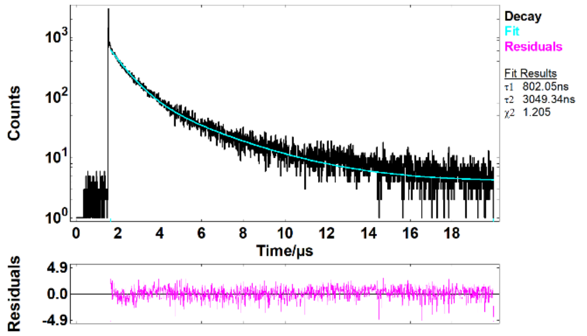

Fit results: τ_1 = 802.05 ns ± 15.59 ns (52.66%) τ_2 = 3049.34 ns ± 82.92 ns (47.34%)

solid

Figure S15. Summary of photoluminescence properties of complex **6**.

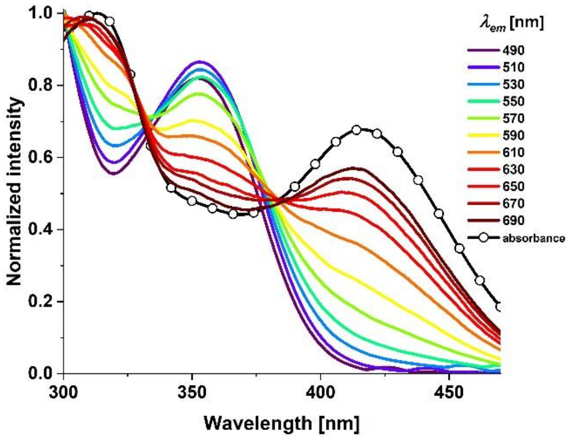

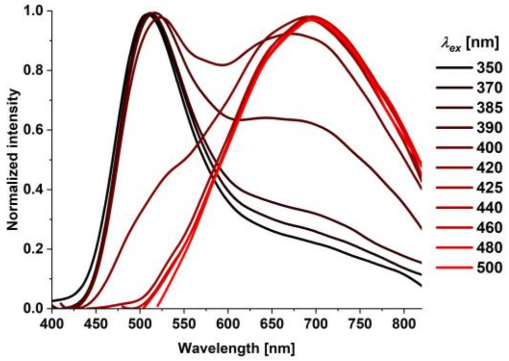

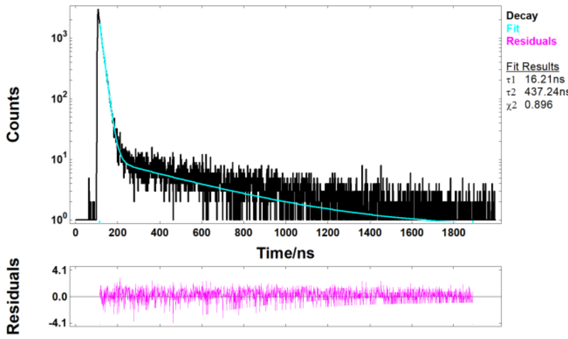

λ_{em} = 510 nm

Fit results: τ_1 = 16.21 ns ± 0.11 ns (87.40%)

τ_2 = 438.24 ns ± 24.96 ns (12.60%)

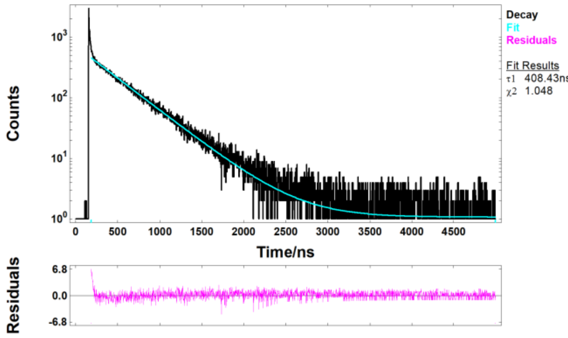

λ_{em} = 695 nm

Fit results: τ_1 = 408.43 ns ± 1.71 ns (100%)

CH_3CN

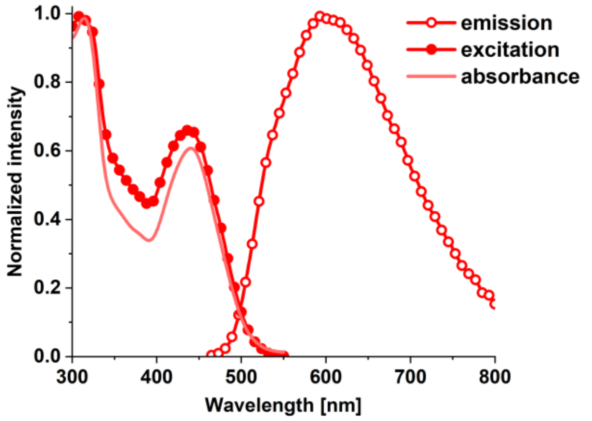

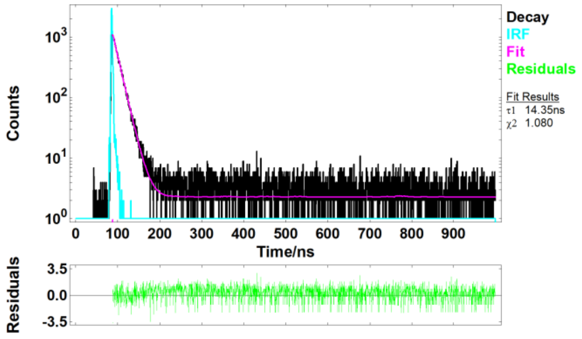

λ_{em} = 510 nm

Fit results: τ_1 = 14.35 ns ± 0.10 ns (100%)

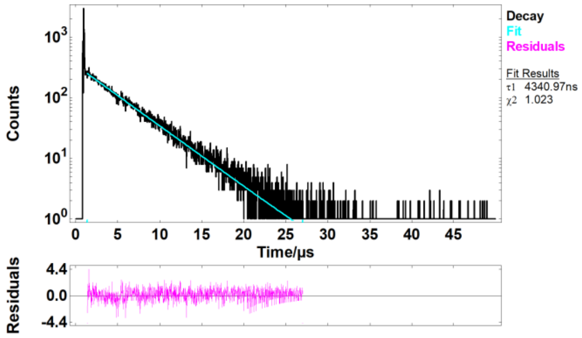

λ_{em} = 610 nm

Fit results: τ_1 = 4340.97 ns ± 30.02 ns (100%)

$CHCl_3$

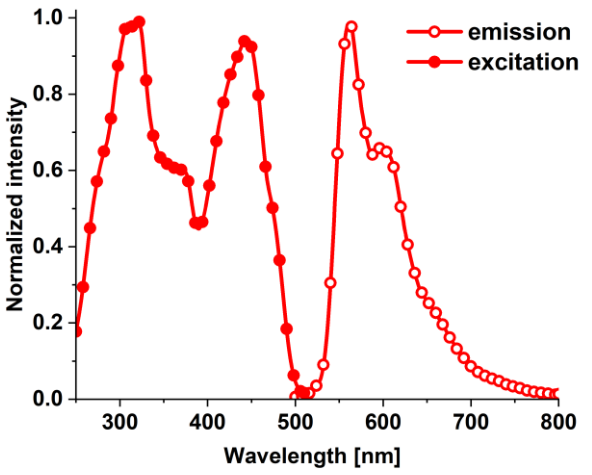

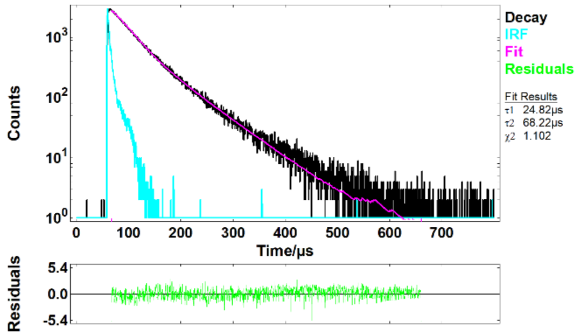

Fit results: τ_1 = 24.82 μs ± 0.52 μs (46.27%)

τ_2 = 68.22 μs ± 0.62 μs (53.73%)

77 K (EtOH:MeOH 4:1 v/v)

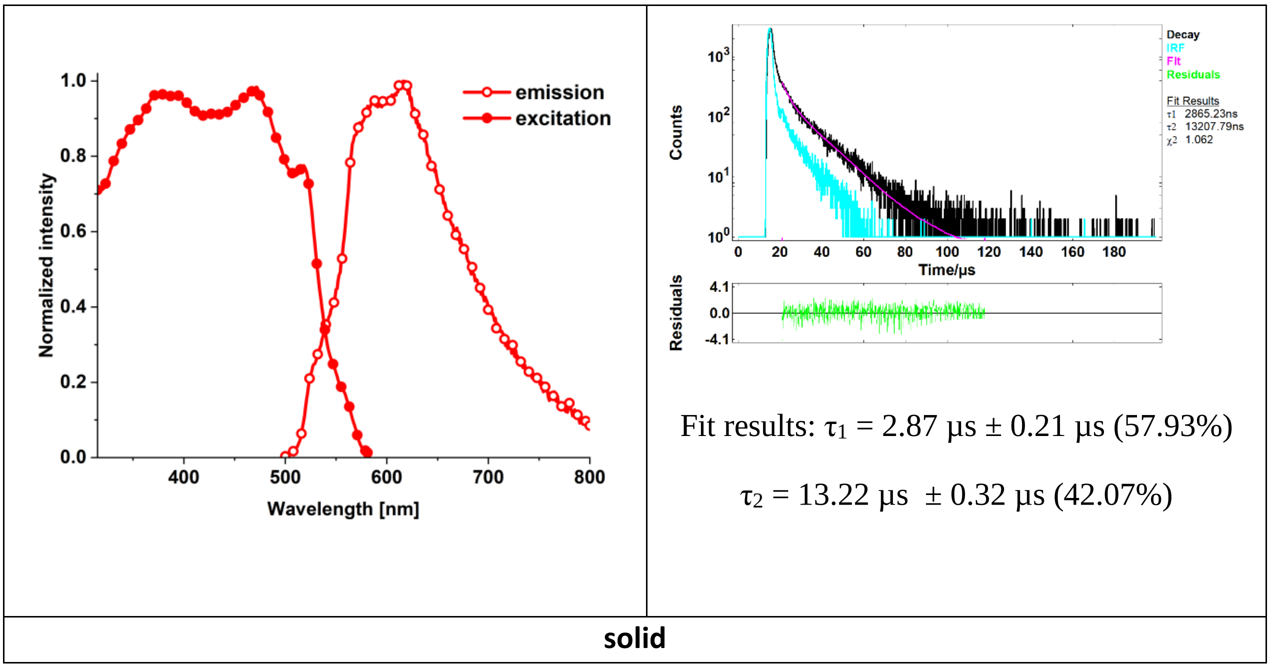

Figure S16. Summary of photoluminescence properties of complex **7**.

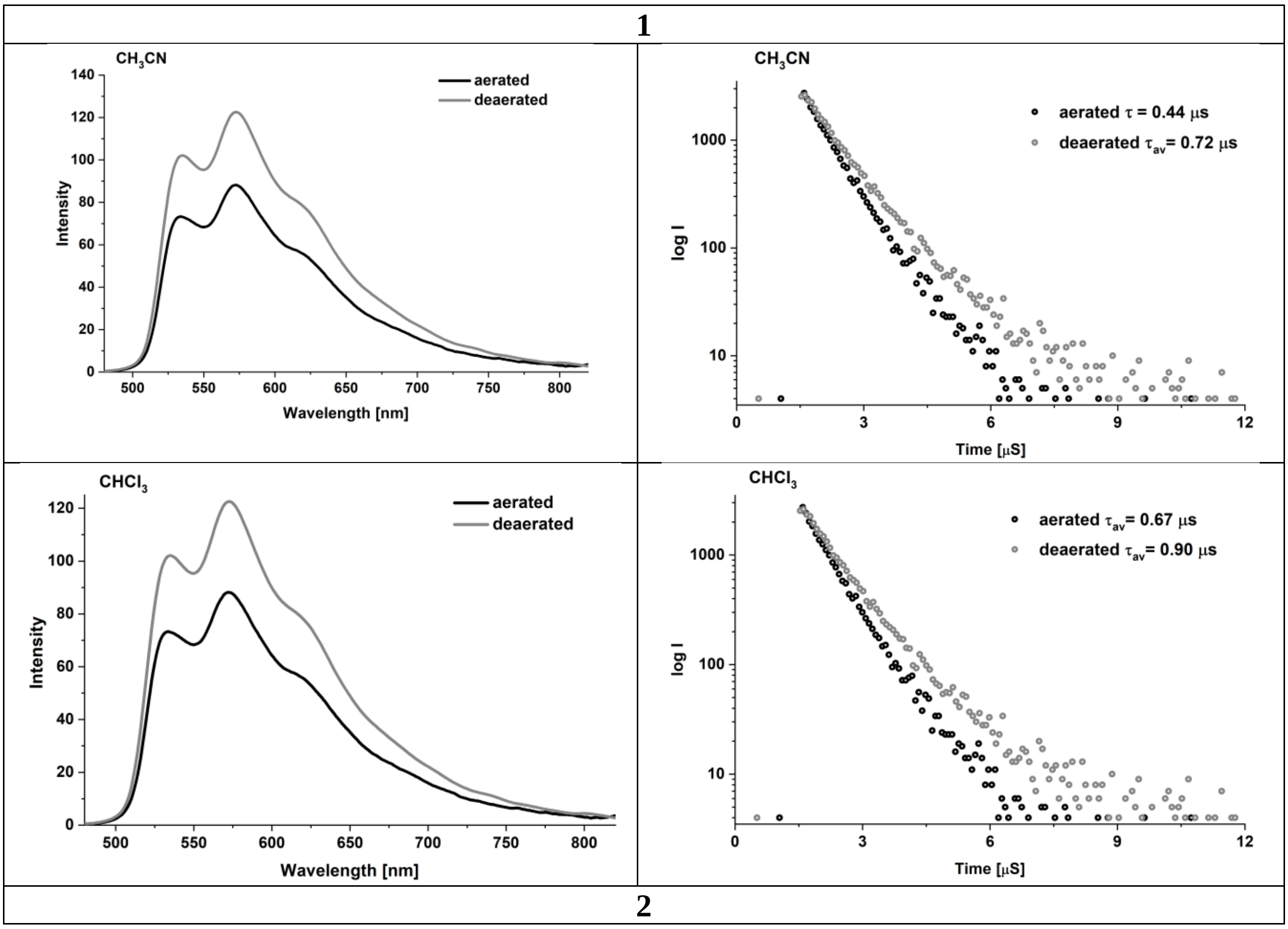

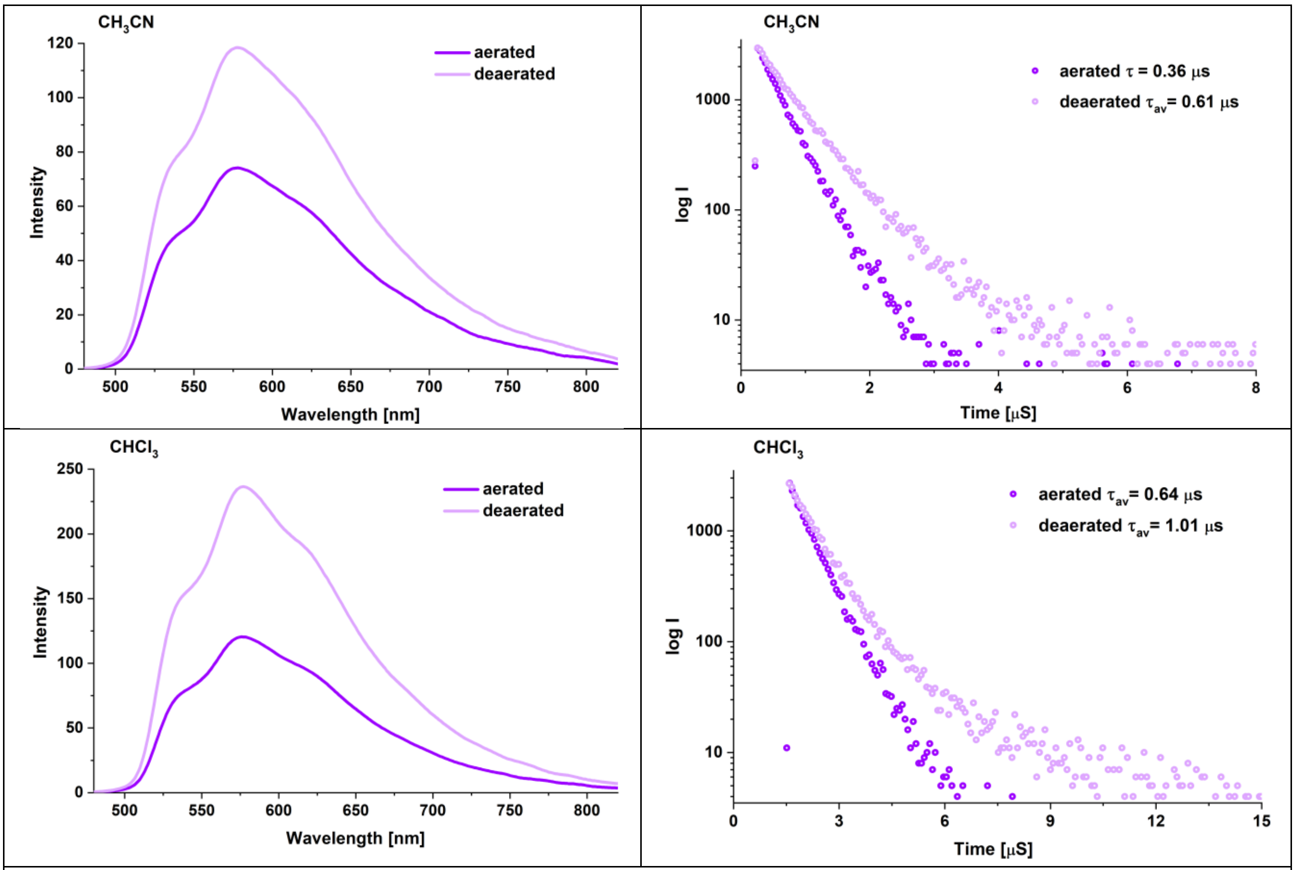
CH$_3$CN
aerated
deaerated
Intensity
Wavelength [nm]
aerated τ = 0.36 μs
deaerated τ$_{av}$= 0.61 μs
log I
Time [μS]
CHCl$_3$
aerated
deaerated
Intensity
Wavelength [nm]
aerated τ$_{av}$= 0.64 μs
deaerated τ$_{av}$= 1.01 μs
log I
Time [μS]

3

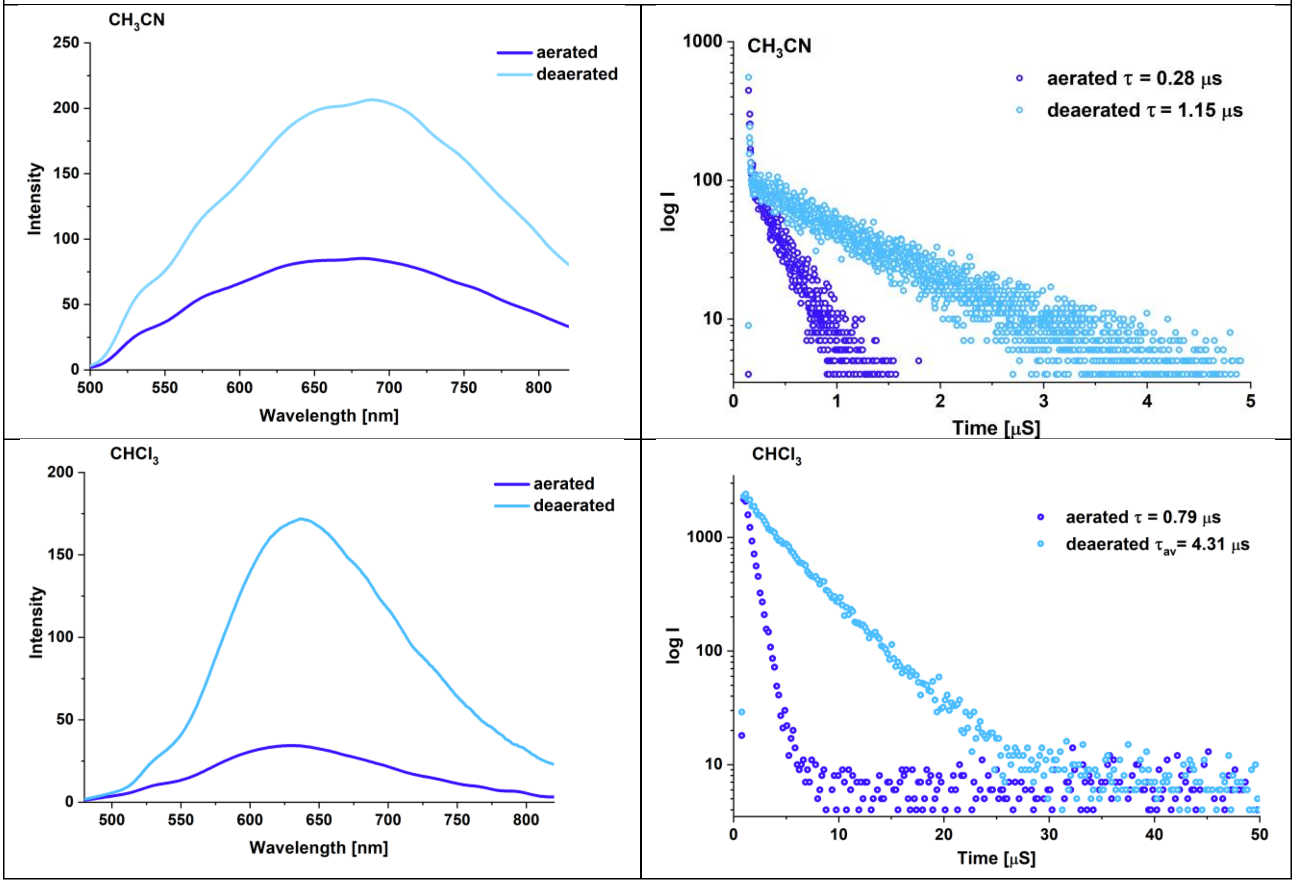
CH$_3$CN
aerated
deaerated
Intensity
Wavelength [nm]
aerated τ = 0.28 μs
deaerated τ = 1.15 μs
log I
Time [μS]
CHCl$_3$
aerated
deaerated
Intensity
Wavelength [nm]
aerated τ = 0.79 μs
deaerated τ$_{av}$= 4.31 μs
log I
Time [μS]

4

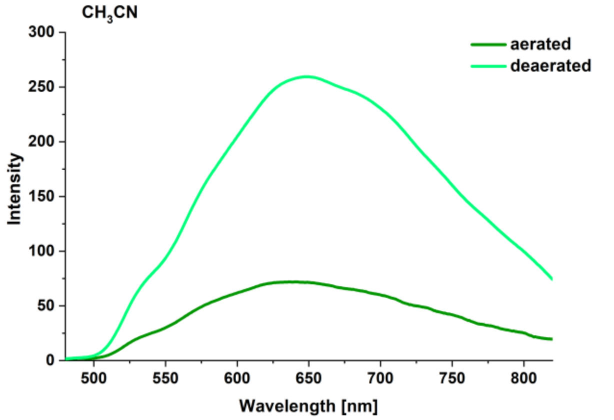
CH_3CN
aerated
deaerated
Intensity
Wavelength [nm]

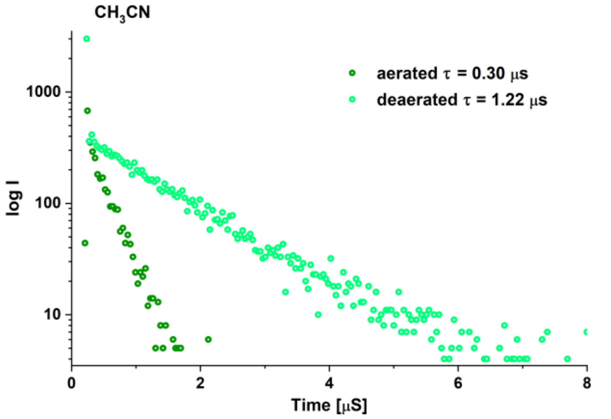
CH_3CN
aerated τ = 0.30 μs
deaerated τ = 1.22 μs
log I
Time [μS]

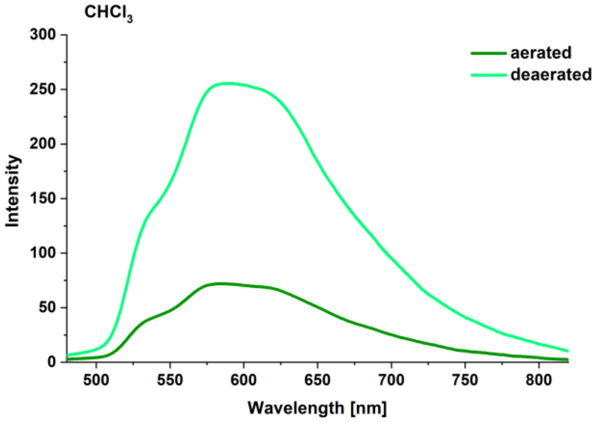
$CHCl_3$
aerated
deaerated
Intensity
Wavelength [nm]

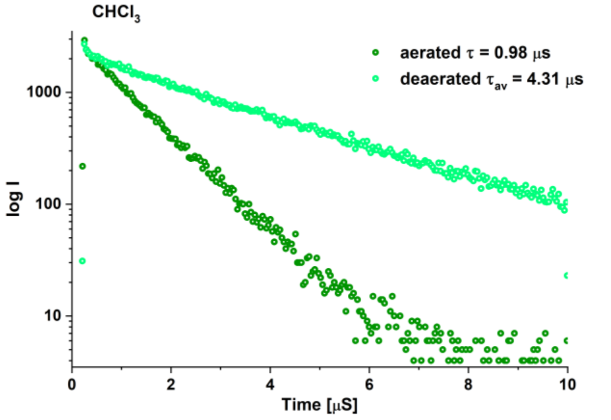
$CHCl_3$
aerated τ = 0.98 μs
deaerated τ_{av} = 4.31 μs
log I
Time [μS]

5

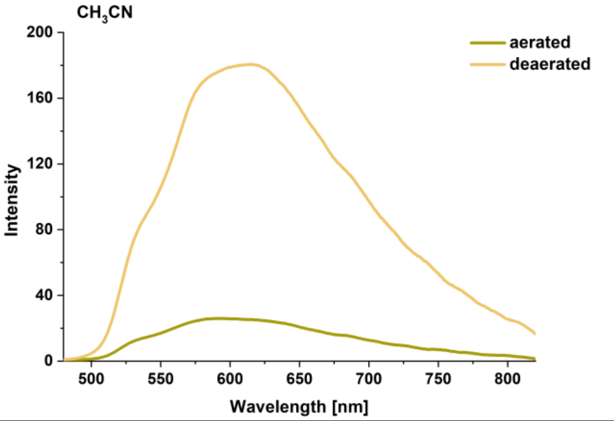
CH_3CN
aerated
deaerated
Intensity
Wavelength [nm]

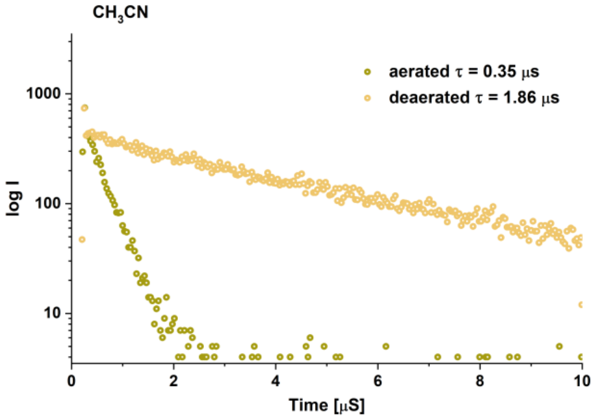
CH_3CN
aerated τ = 0.35 μs
deaerated τ = 1.86 μs
log I
Time [μS]

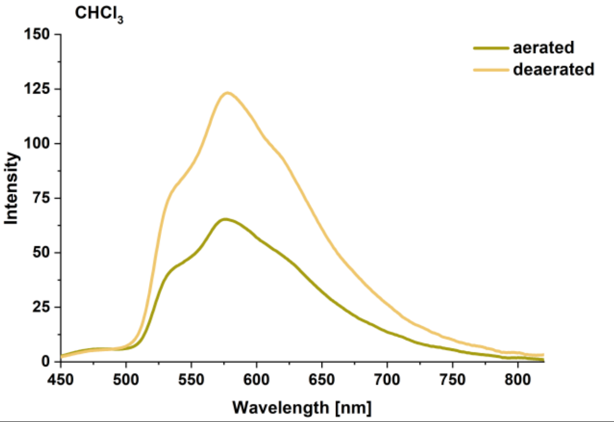
$CHCl_3$
aerated
deaerated
Intensity
Wavelength [nm]

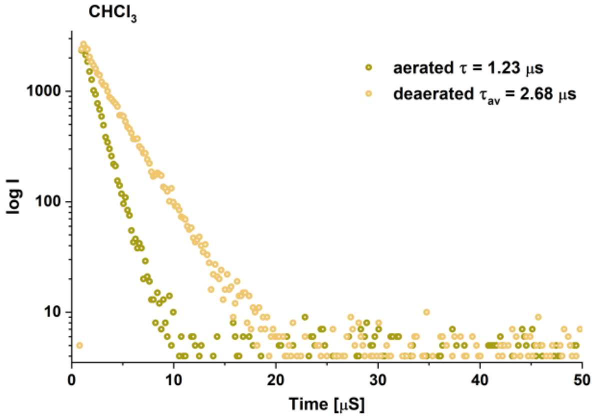
$CHCl_3$
aerated τ = 1.23 μs
deaerated τ_{av} = 2.68 μs
log I
Time [μS]

<table>
<tr><th colspan="2">6</th></tr>
<tr><td>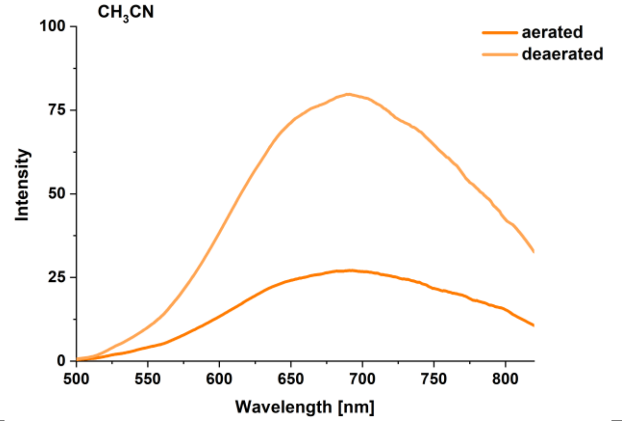
</td><td>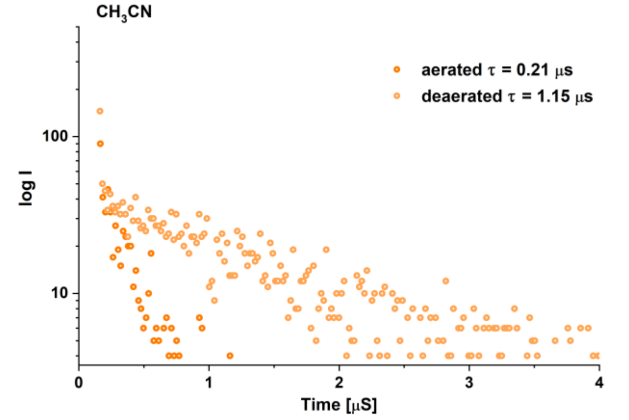
</td></tr>
<tr><td>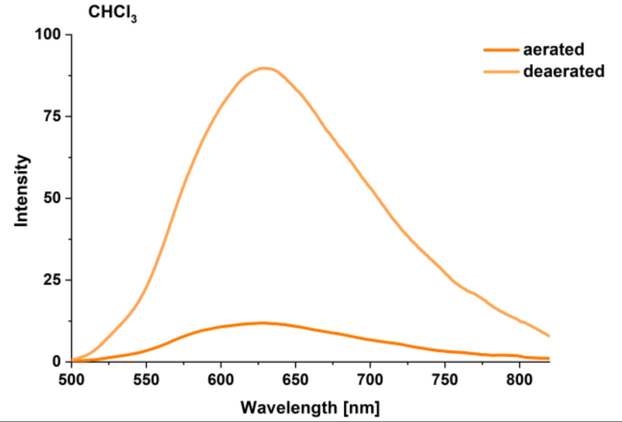
</td><td>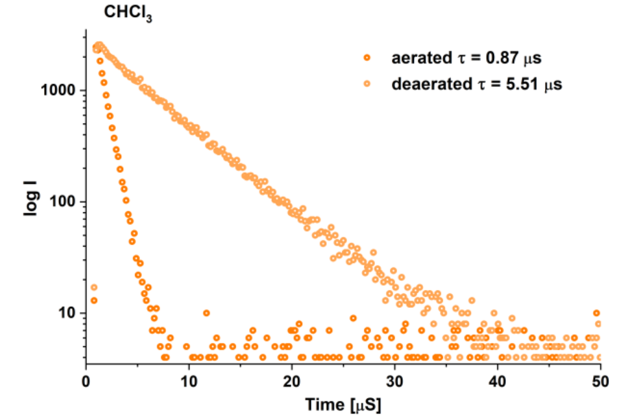
</td></tr>
<tr><th colspan="2">7</th></tr>
<tr><td>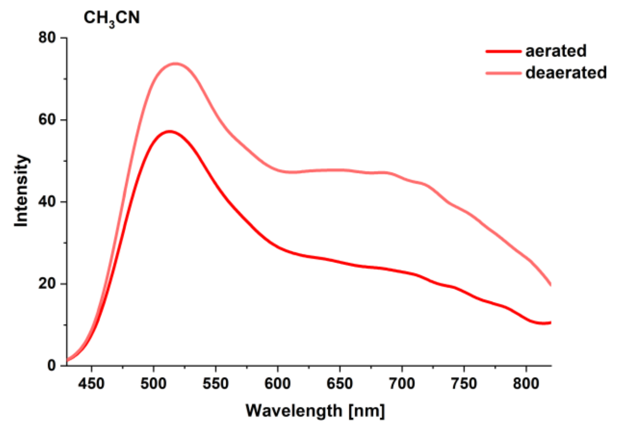
</td><td>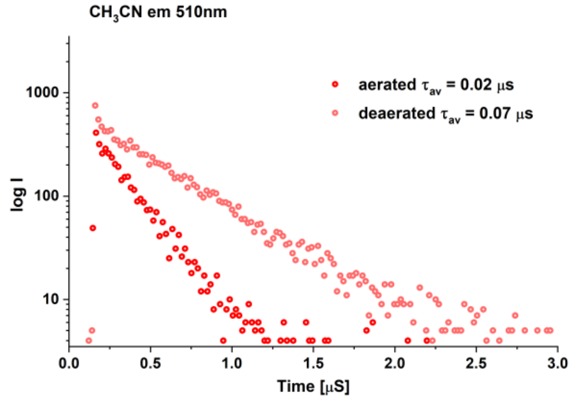

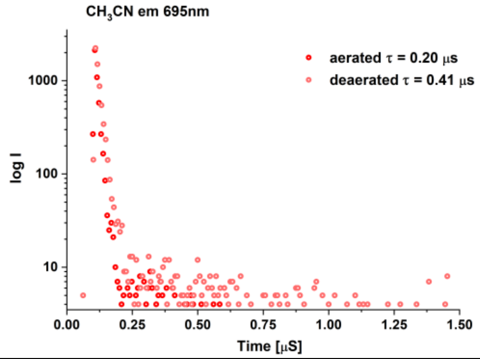
</td></tr>
</table>

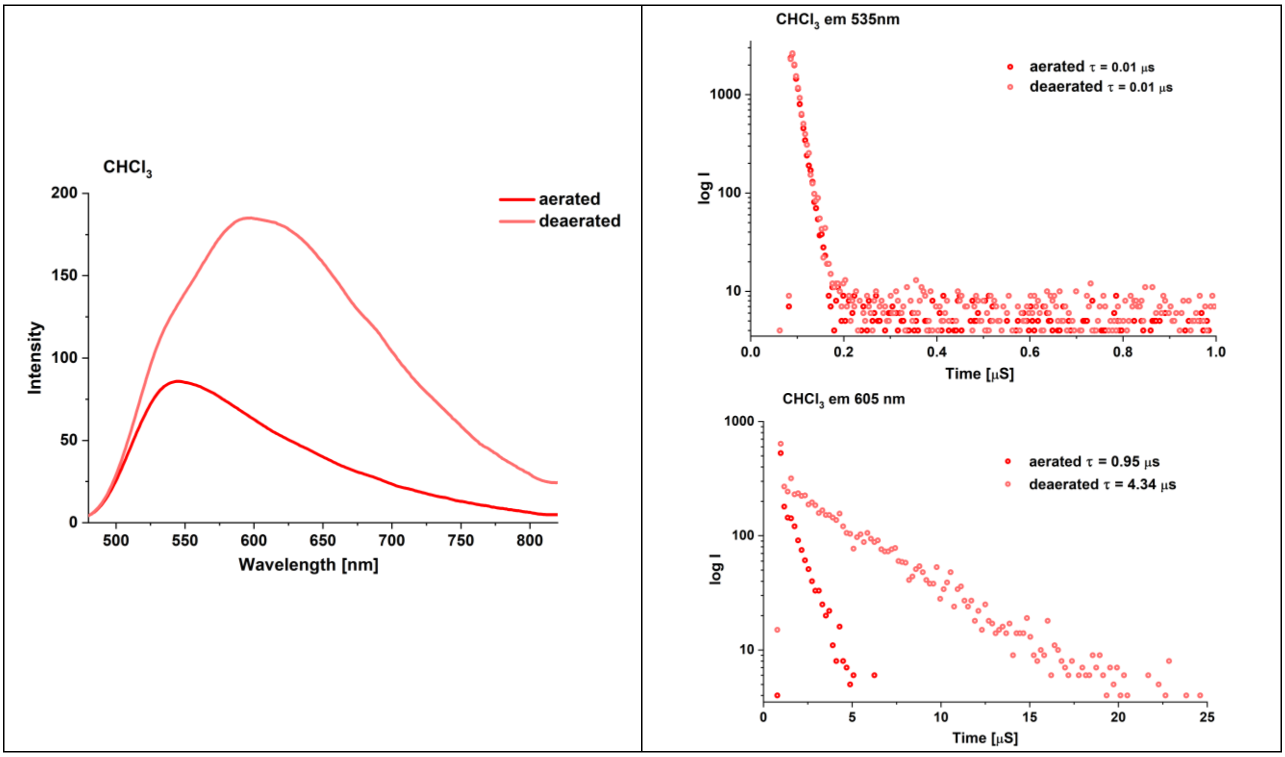

Figure S17. Emission spectra and decay curves of **1–7** in $CHCl_3$ and CH_3CN in argon-saturated and air-equilibrated samples.

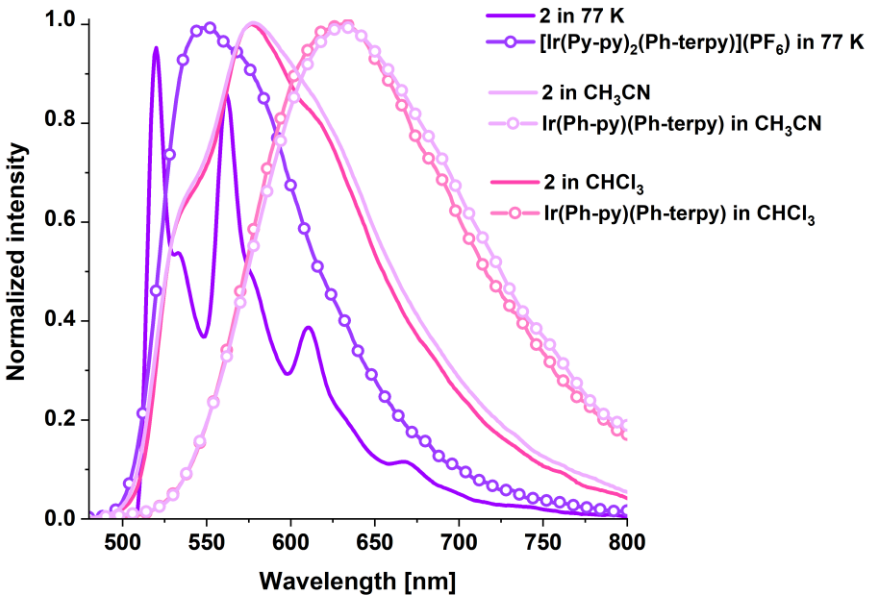

Figure S18. The phosphorescence spectra of [Ir(Ph-btz)$_2$(Ph-terpy)](PF_6) (**2**) compared to those of [Ir(Py-py)$_2$(Ph-terpy)](PF_6).

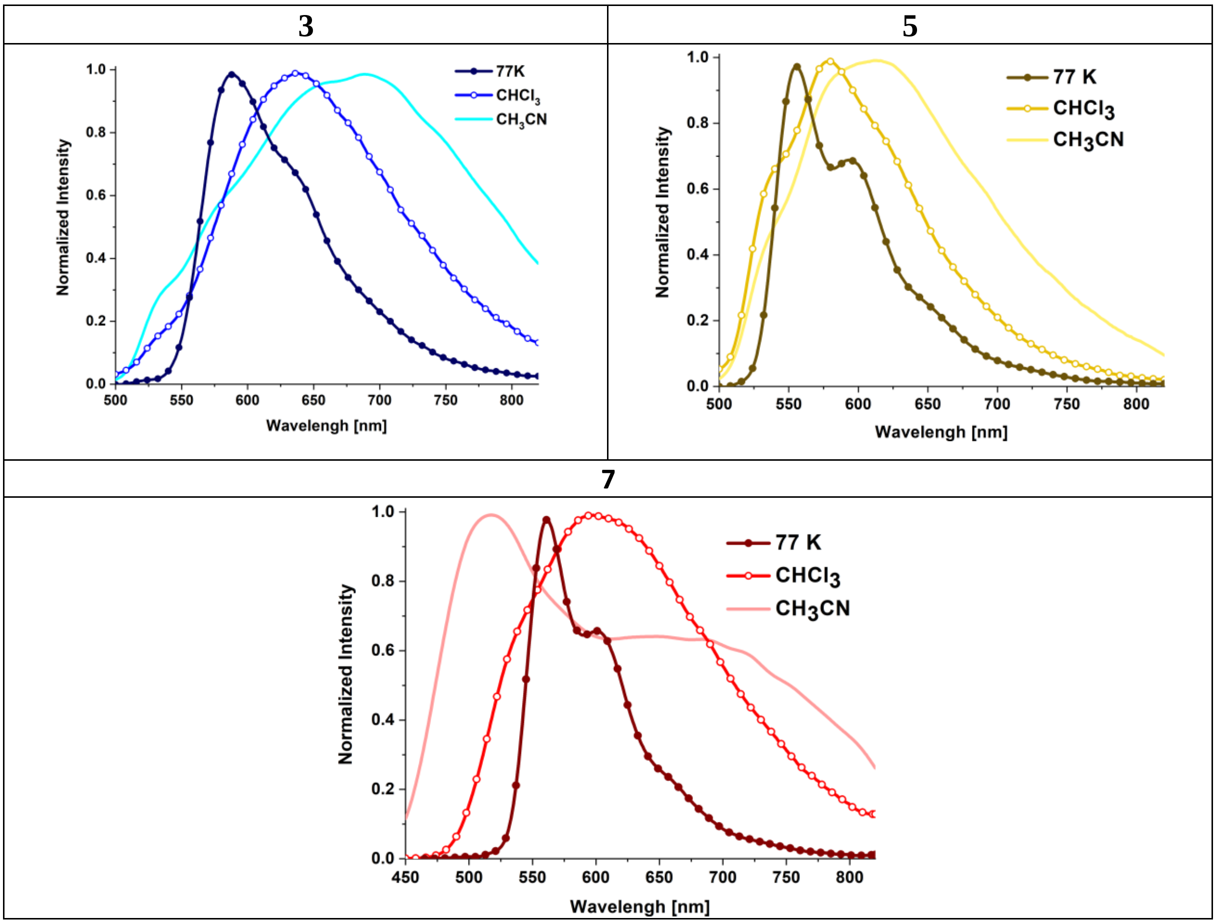

Figure S19. Normalized emission spectra of **3**, **5** and **7** in the matrix at 77 K and solution at room temperature.

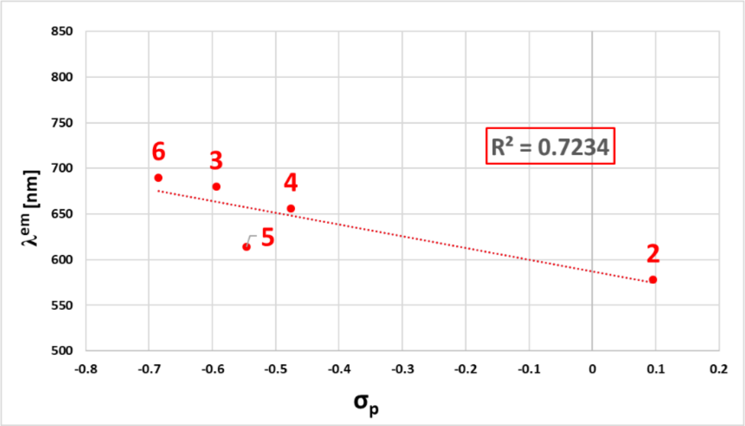

Figure S20. Linear correlation of λ^{em} of **2–6** with the calculated σ_p parameter of the R–group.

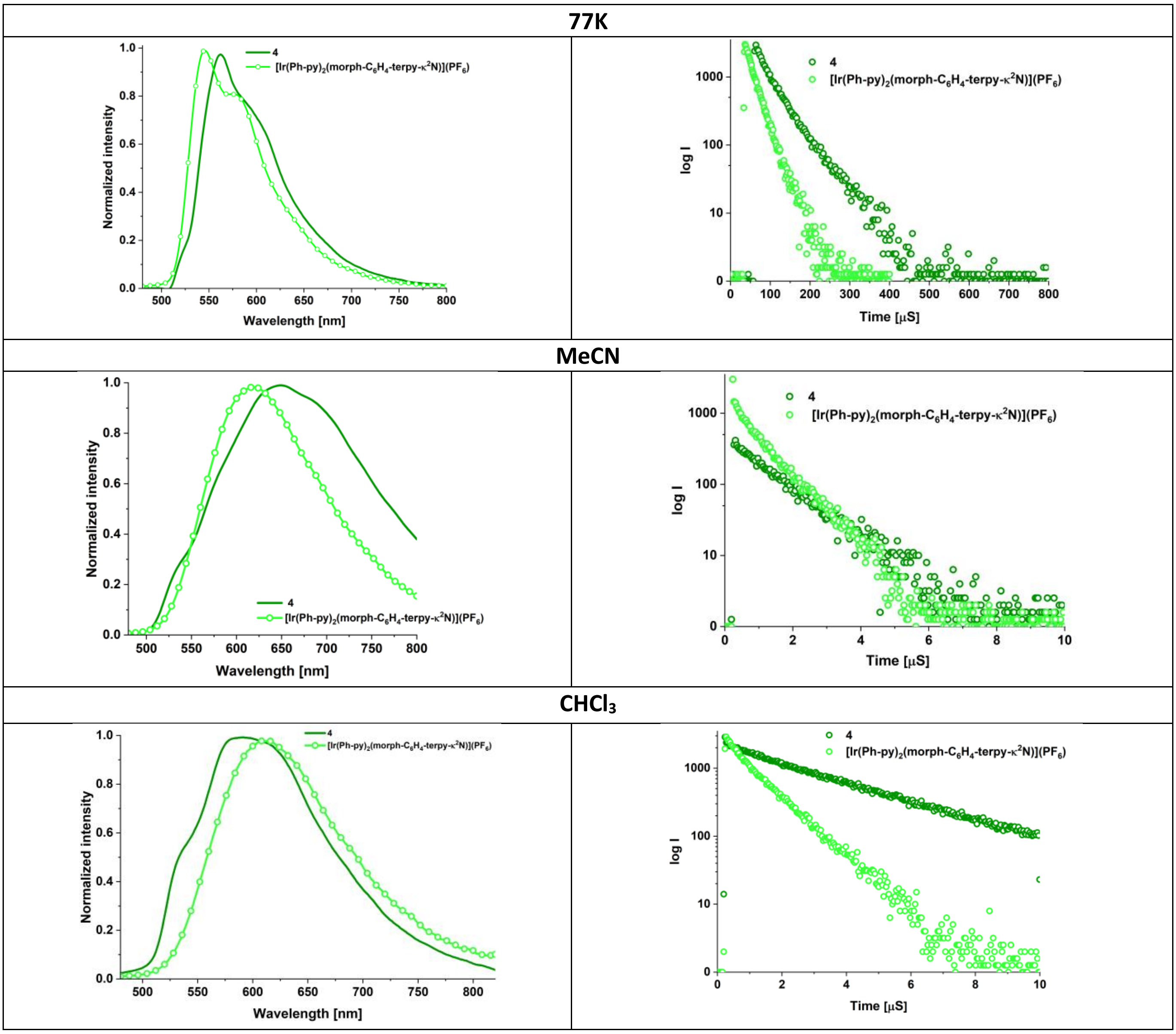

Figure S21. Comparative analysis of photoluminescent data of **4** with those for its analogue [Ir(Ph-py)$_2$(morph-C$_6$H$_4$-terpy-κ^{2}N)]PF$_6$.[16] Reproduced from 16 Copyright 2025 American Chemical Society.

1

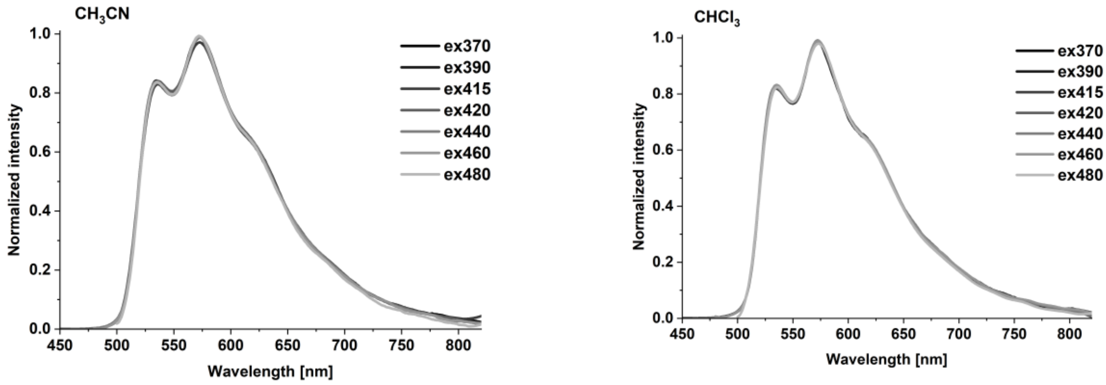

2

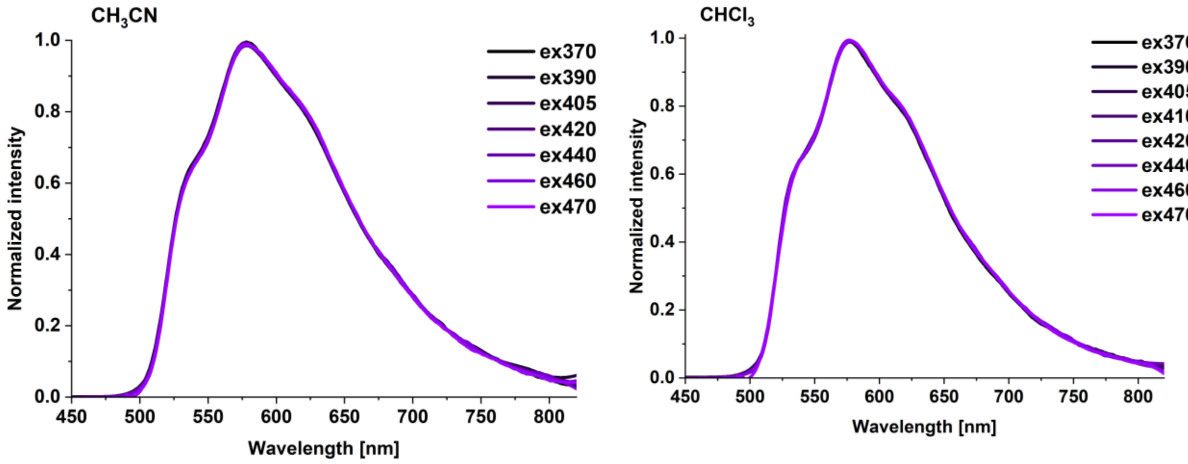

CH_3CN
Normalized intensity
Wavelength [nm]
ex370
ex390
ex405
ex420
ex440
ex460
ex470
$CHCl_3$
Normalized intensity
Wavelength [nm]
ex370
ex390
ex405
ex410
ex420
ex440
ex460
ex470

3

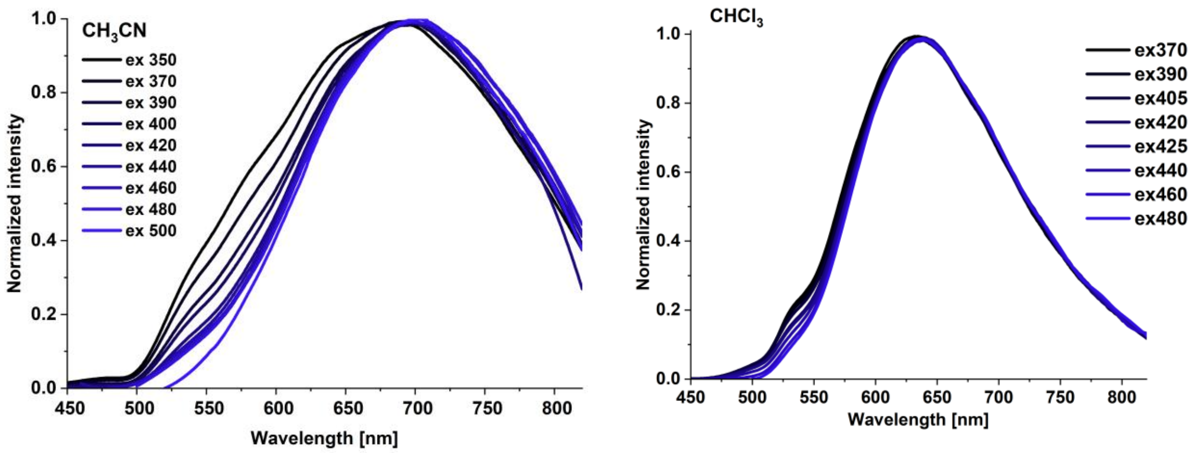

CH_3CN
Normalized intensity
Wavelength [nm]
ex 350
ex 370
ex 390
ex 400
ex 420
ex 440
ex 460
ex 480
ex 500
$CHCl_3$
Normalized intensity
Wavelength [nm]
ex370
ex390
ex405
ex420
ex425
ex440
ex460
ex480

4

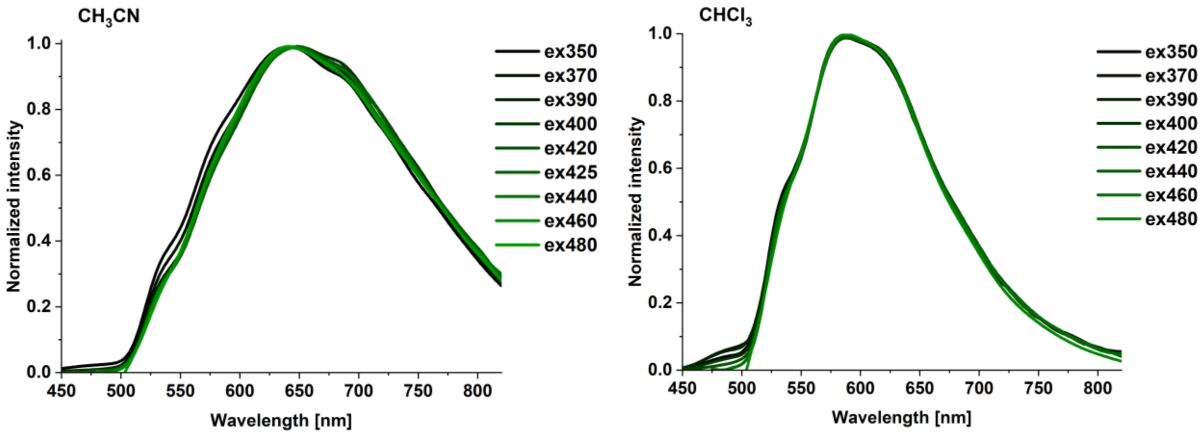

CH_3CN
Normalized intensity
Wavelength [nm]
ex350
ex370
ex390
ex400
ex420
ex425
ex440
ex460
ex480
$CHCl_3$
Normalized intensity
Wavelength [nm]
ex350
ex370
ex390
ex400
ex420
ex440
ex460
ex480

5

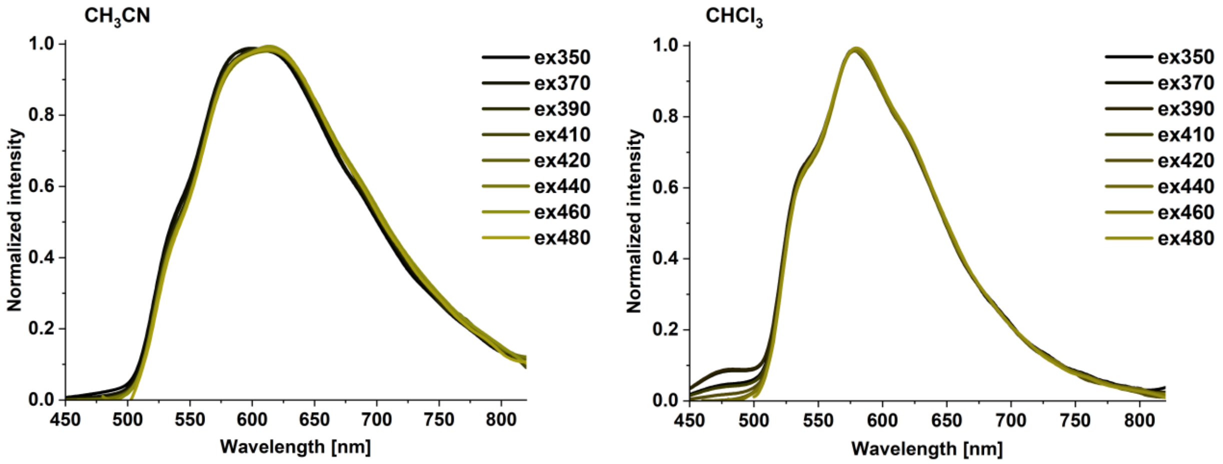

6

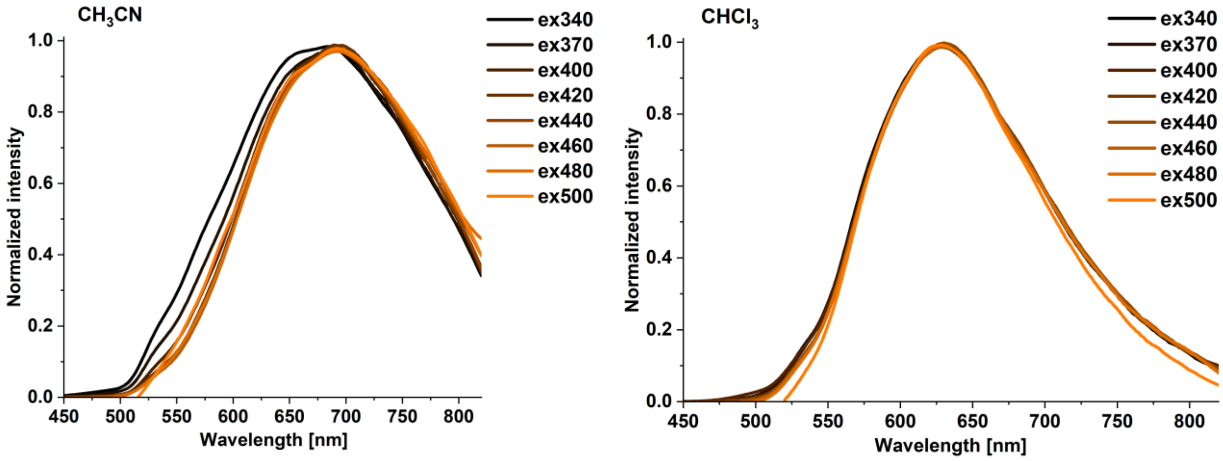

7

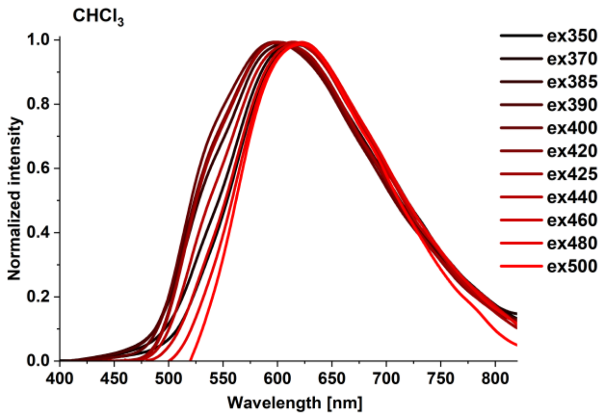

Figure S22. Emission spectra of **1**–**7** upon different excitation wavelengths.

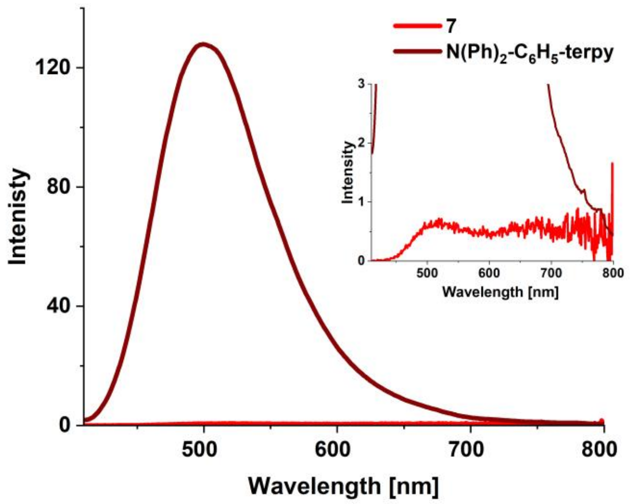

Figure S23. Emission spectra of N(Ph)$_2$-C$_6$H$_5$-terpy and complex 7 in CH_3CN.

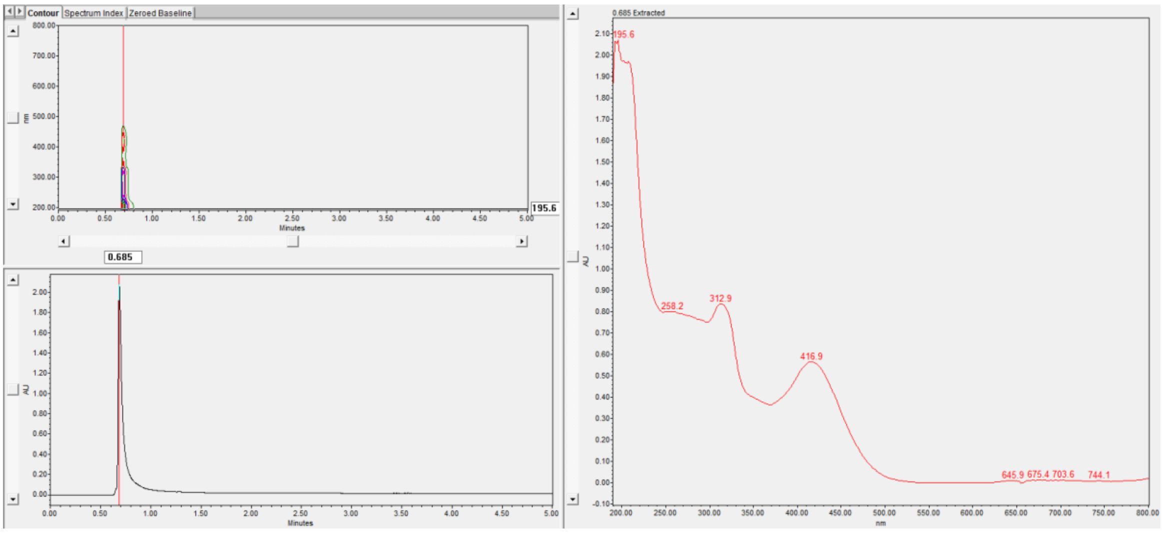

Figure S24. UPLC spectra for **7.**

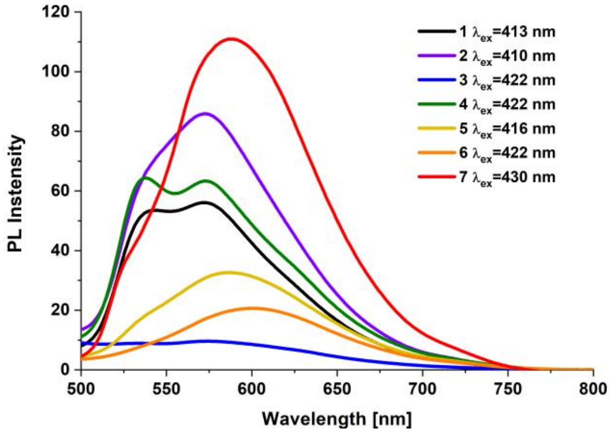

Figure S25. Emission spectra of **1–7** in the solid state as thin films deposited on a glass substrate.

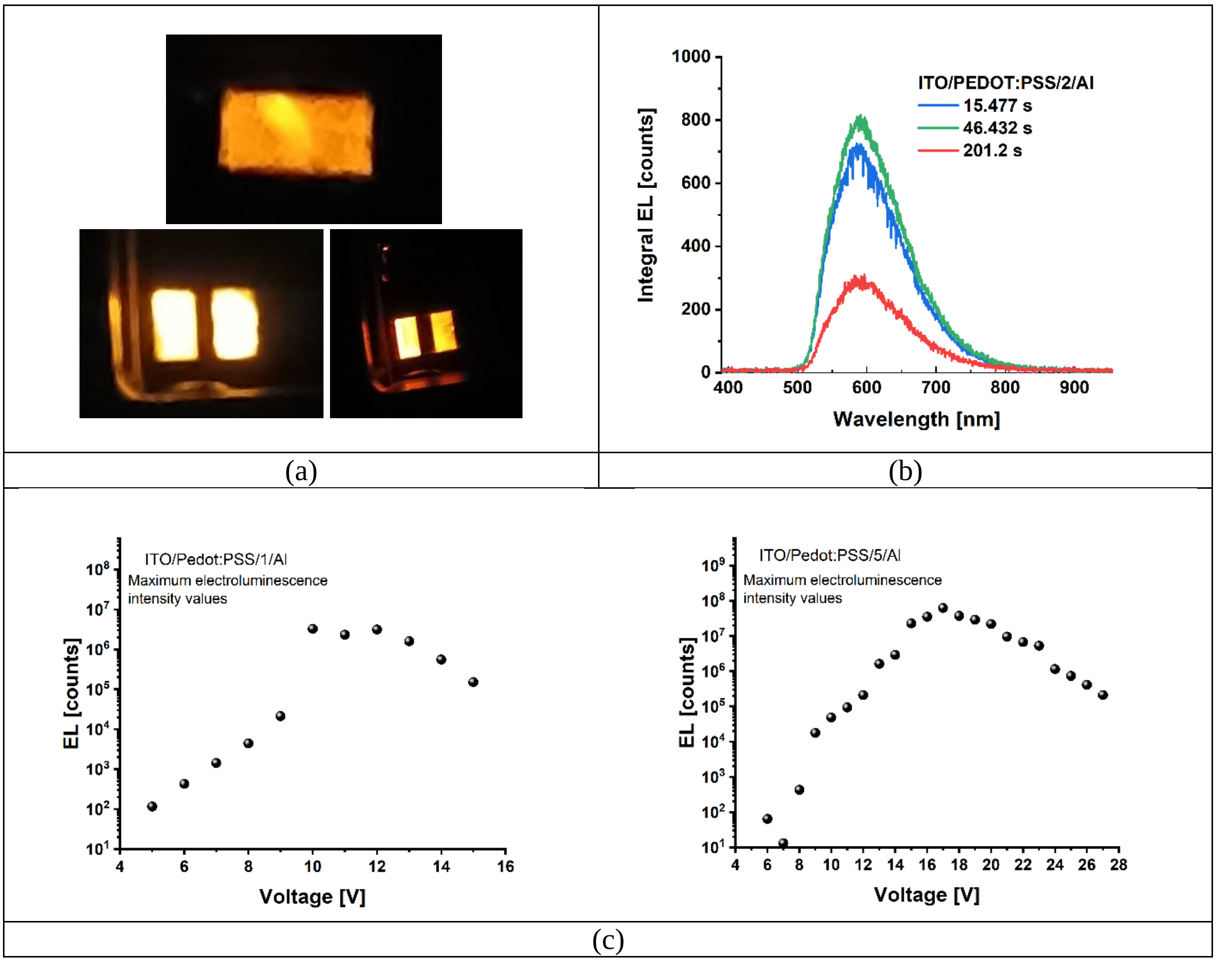

Figure S26. Photos of exemplary diodes **3**, **5** and **6** (a), the EL intensity recorded as a function of time and EL spectra of ITO/PEDOT:PSS/2/Al (b), effect of applied voltage on EL intensity of diode ITO/PEDOT:PSS/1/Al and ITO/PEDOT:PSS/5/Al (c)

Table S8. EL data of the fabricated diodes ITO/PEDOT:PSS/ complex/Al and ITO/PEDOT:PSS/PVK:PBD:complex/Al obtained under external voltage 11 V.

	**Active layer composition**						
	**1**	**2**	**3**	**4**	**5**	**6**	**7**
λ_{EL}[nm]	584	588	624	600	611	638	610
Intensity [counts]	2 306 800	2 708 100	237 650	23 720	93 230	172	1 260 700
	**PVK:PBD: 2 wt% of complex**						
λ_{EL}[nm]	576	578	581	580	578	578	576
Intensity [counts]	1 623	295	91	616	1 140	605	52
	**PVK:PBD: 15 wt% of complex**						
λ_{EL}[nm]	578	580	598	575	578	590	582
Intensity [counts]	9 328	1 726	130	5 144	2 760	1 016	1 693

Transient absorption spectroscopy

1

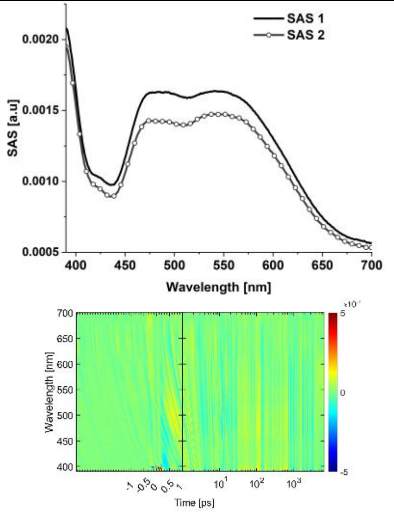

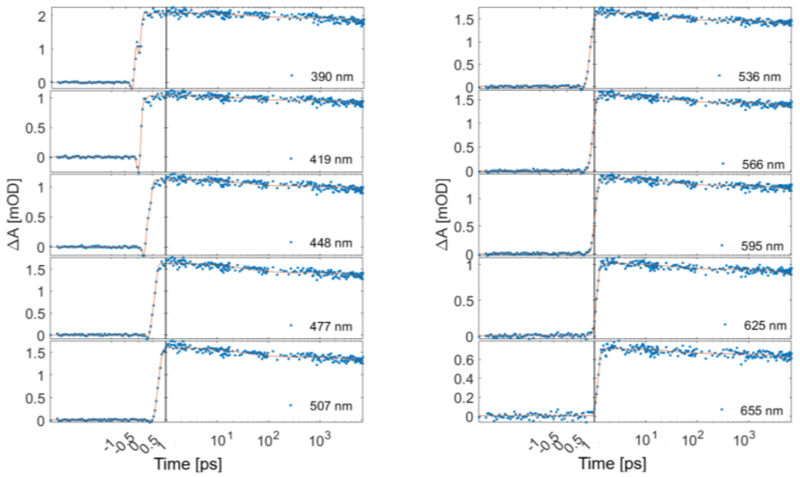

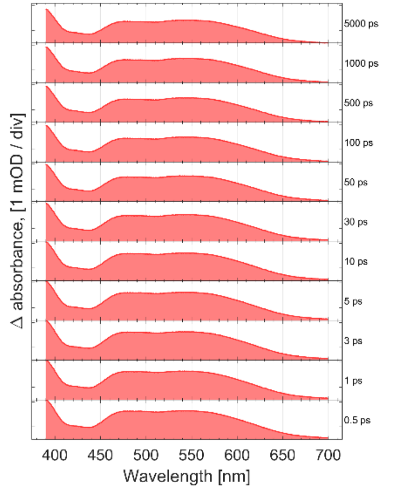

2

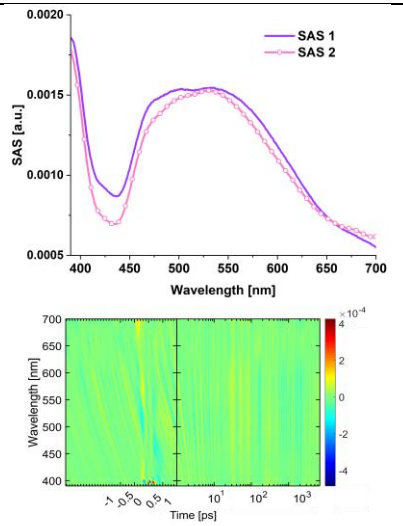

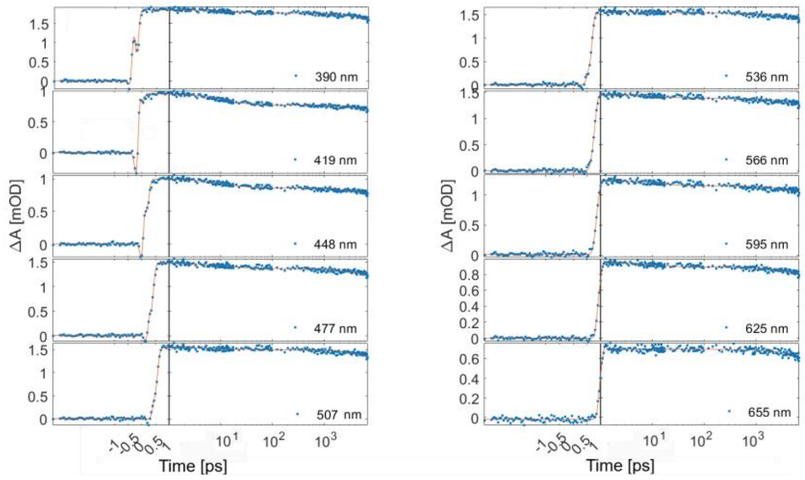

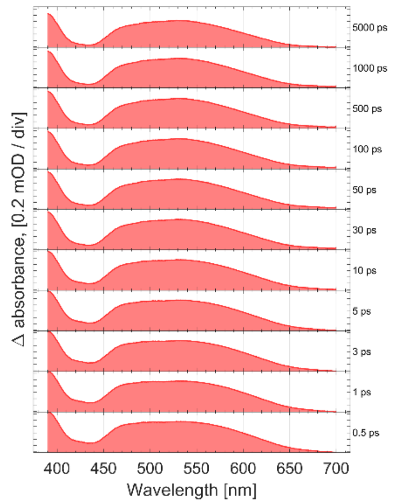

3

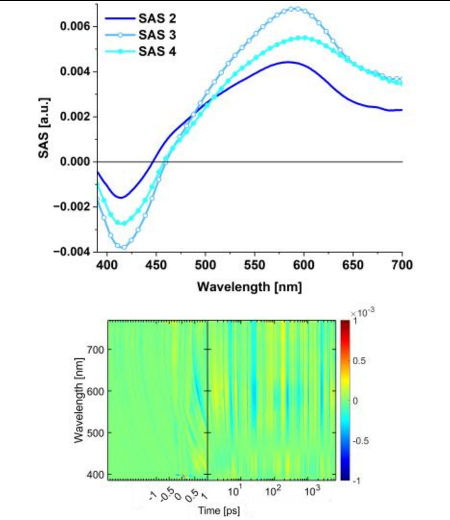
SAS 2
SAS 3
SAS 4
SAS [a.u.]
Wavelength [nm]
Wavelength [nm]
Time [ps]

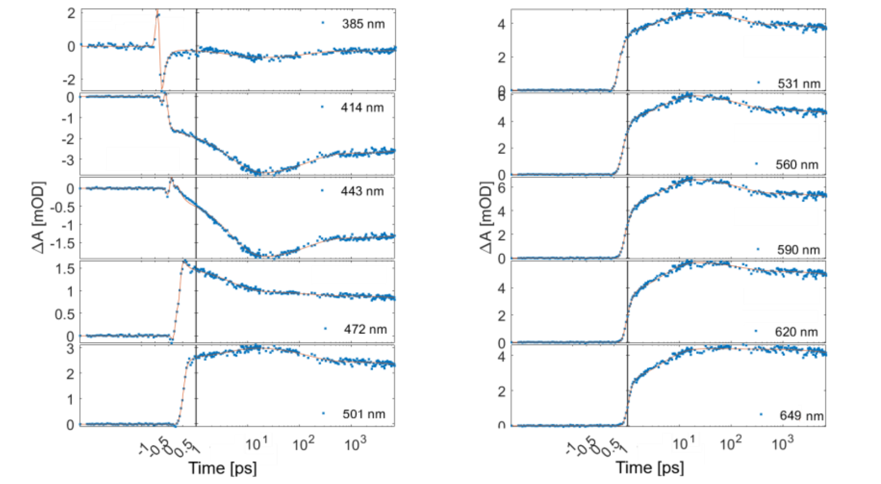
385 nm
414 nm
443 nm
472 nm
501 nm
531 nm
560 nm
590 nm
620 nm
649 nm
ΔA [mOD]
ΔA [mOD]
Time [ps]
Time [ps]

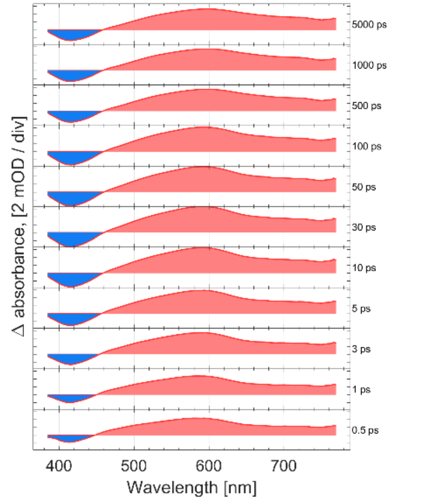
5000 ps
1000 ps
500 ps
100 ps
50 ps
30 ps
10 ps
5 ps
3 ps
1 ps
0.5 ps
Δ absorbance, [2 mOD / div]
Wavelength [nm]

4

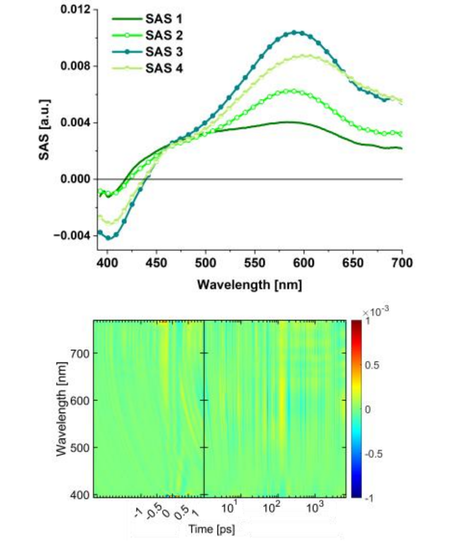
SAS 1
SAS 2
SAS 3
SAS 4
SAS [a.u.]
Wavelength [nm]
Wavelength [nm]
Time [ps]

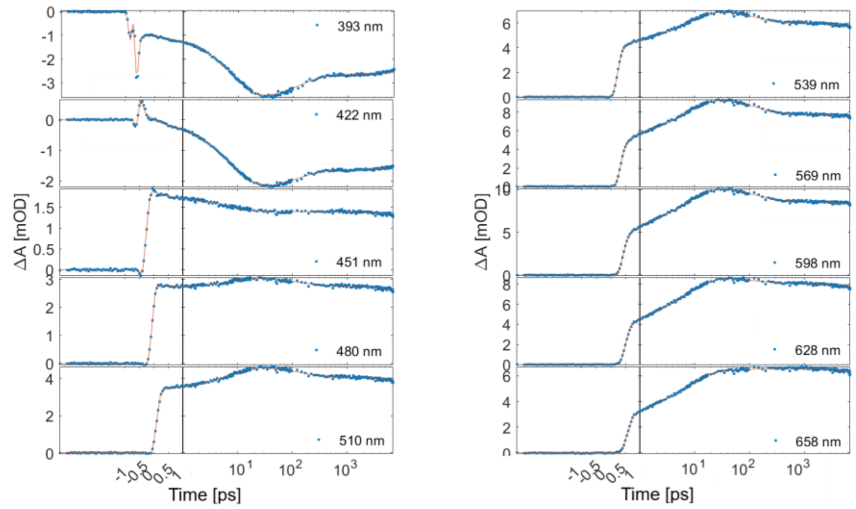
393 nm
422 nm
451 nm
480 nm
510 nm
539 nm
569 nm
598 nm
628 nm
658 nm
ΔA [mOD]
ΔA [mOD]
Time [ps]
Time [ps]

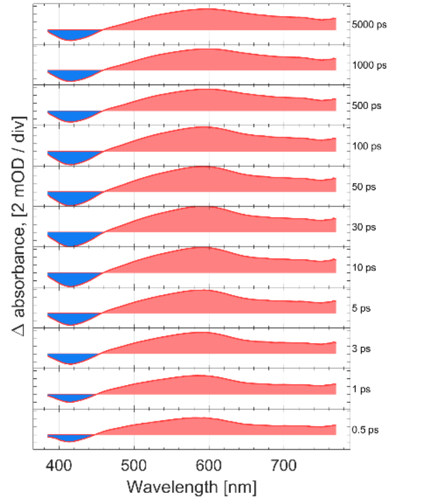
5000 ps
1000 ps
500 ps
100 ps
50 ps
30 ps
10 ps
5 ps
3 ps
1 ps
0.5 ps
Δ absorbance, [2 mOD / div]
Wavelength [nm]

5

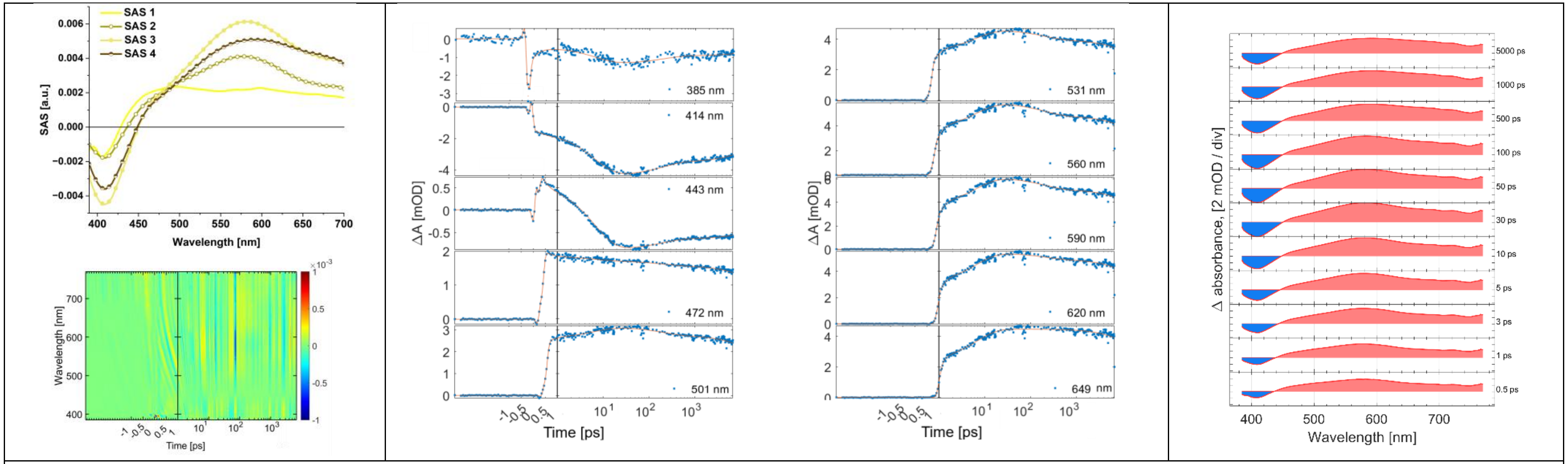

SAS 1
SAS 2
SAS 3
SAS 4
SAS [a.u.]
Wavelength [nm]
Wavelength [nm]
Time [ps]
ΔA [mOD]
385 nm
414 nm
443 nm
472 nm
501 nm
531 nm
560 nm
590 nm
620 nm
649 nm
Time [ps]
Δ absorbance, [2 mOD / div]
5000 ps
1000 ps
500 ps
100 ps
50 ps
30 ps
10 ps
5 ps
3 ps
1 ps
0.5 ps
Wavelength [nm]

6

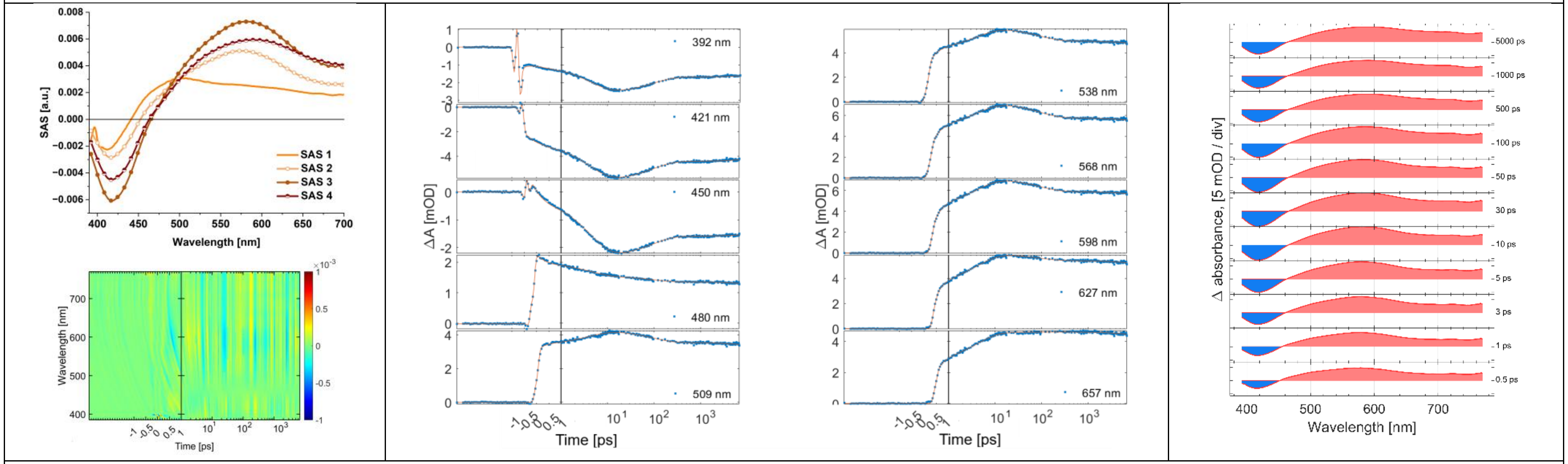

SAS 1
SAS 2
SAS 3
SAS 4
SAS [a.u.]
Wavelength [nm]
Wavelength [nm]
Time [ps]
ΔA [mOD]
392 nm
421 nm
450 nm
480 nm
509 nm
538 nm
568 nm
598 nm
627 nm
657 nm
Time [ps]
Δ absorbance, [5 mOD / div]
5000 ps
1000 ps
500 ps
100 ps
50 ps
30 ps
10 ps
5 ps
3 ps
1 ps
0.5 ps
Wavelength [nm]

7

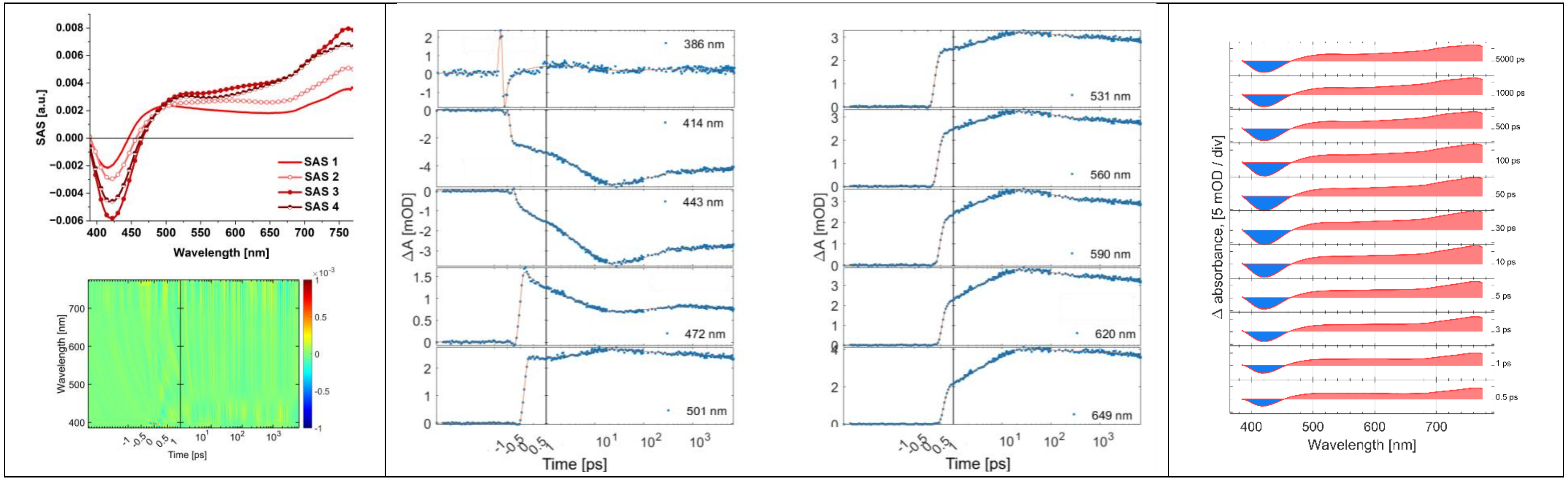

Figure S27. Summary of the global lifetime analysis of **1-7** (acetonitrile, pump wavelength 355 nm; pump power 0.24 μJ per pulse) containing, evolution associated spectra, residual map, time traces at several wavelength and transient spectra.

1

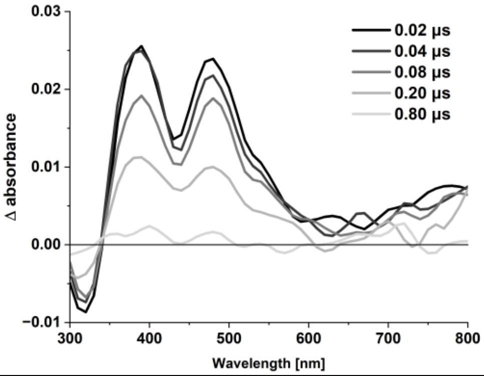
0.02 μs
0.04 μs
0.08 μs
0.20 μs
0.80 μs
Δ absorbance
Wavelength [nm]

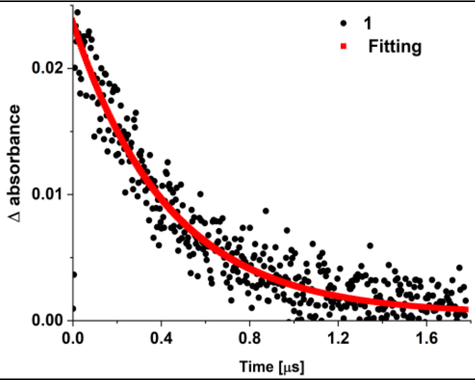
1
Fitting
Δ absorbance
Time [μs]

2

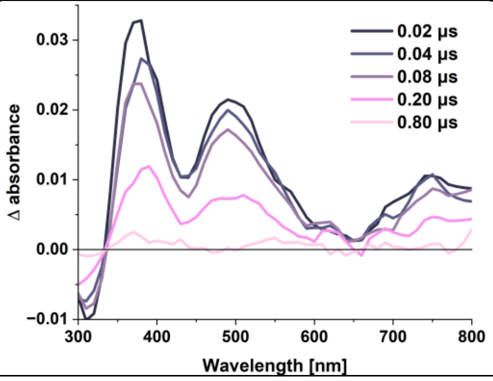
0.02 μs
0.04 μs
0.08 μs
0.20 μs
0.80 μs
Δ absorbance
Wavelength [nm]

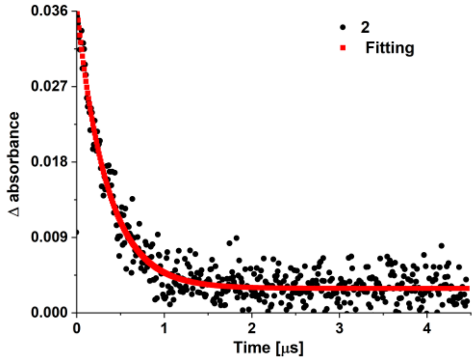
2
Fitting
Δ absorbance
Time [μs]

3

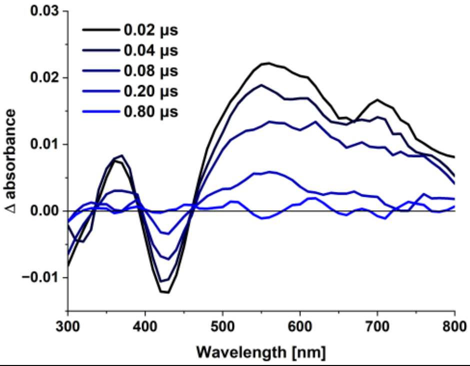
0.02 μs
0.04 μs
0.08 μs
0.20 μs
0.80 μs
Δ absorbance
Wavelength [nm]

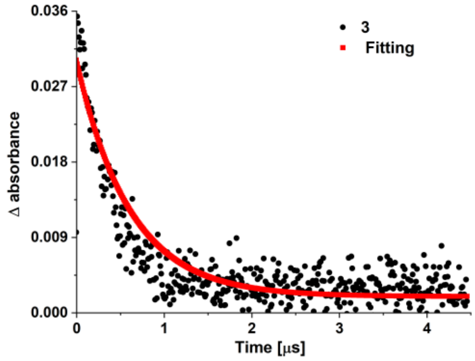
3
Fitting
Δ absorbance
Time [μs]

4

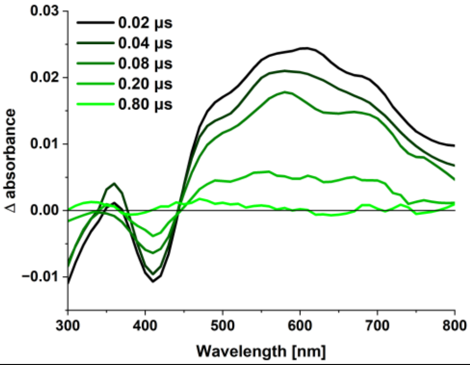
0.02 μs
0.04 μs
0.08 μs
0.20 μs
0.80 μs
Δ absorbance
Wavelength [nm]

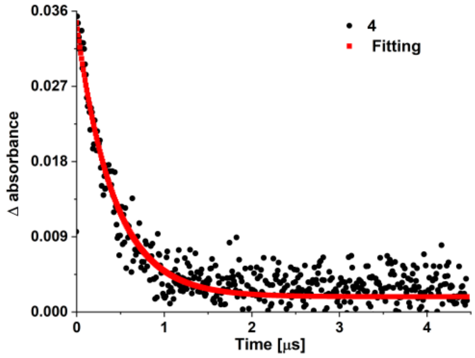
4
Fitting
Δ absorbance
Time [μs]

5

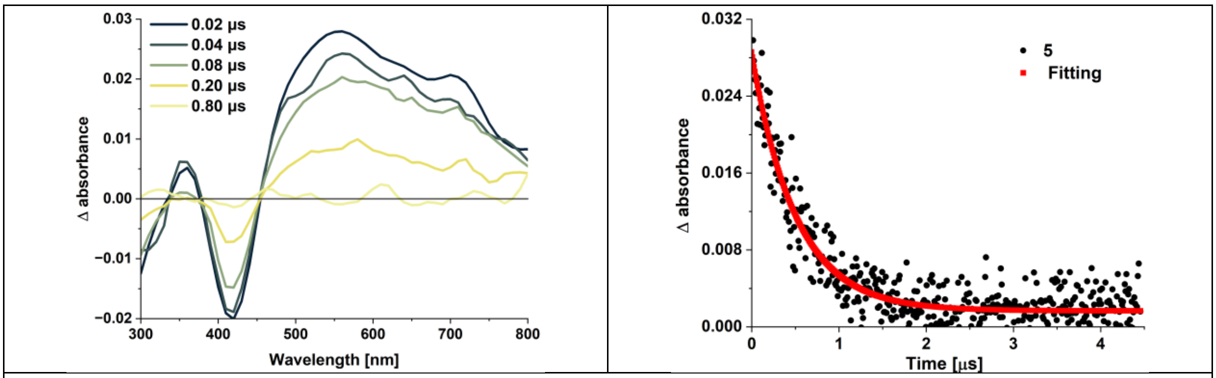

6

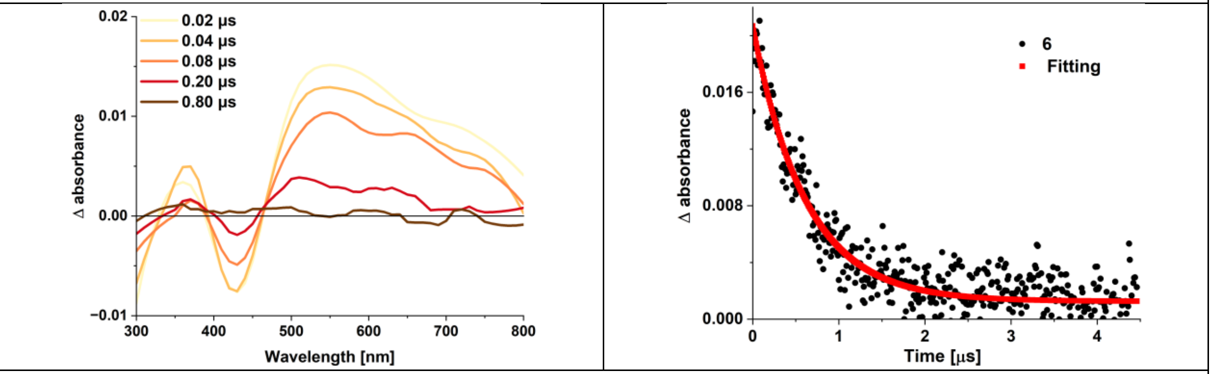

7

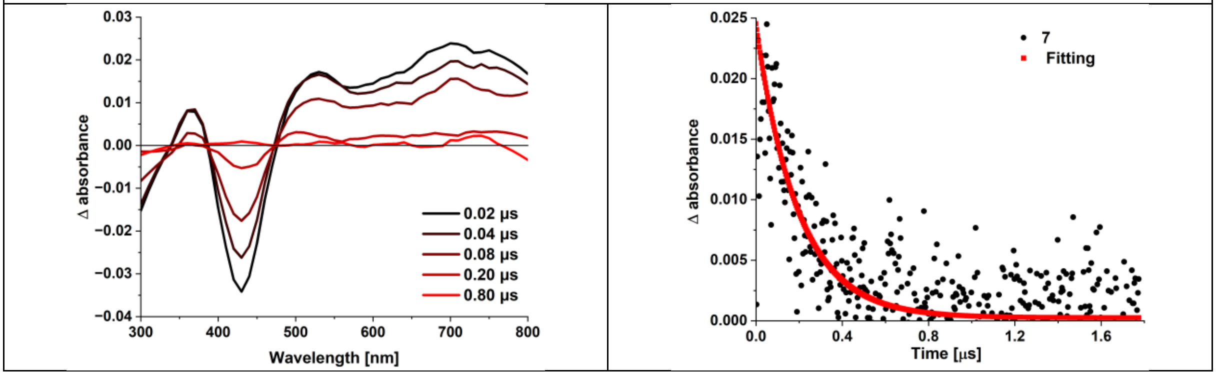

Figure S28. Nanosecond transient absorption (ns-TA) spectra obtained using laser flash photolysis (LFP) in degassed CH_3CN, together with the corresponding decay traces.

3	4
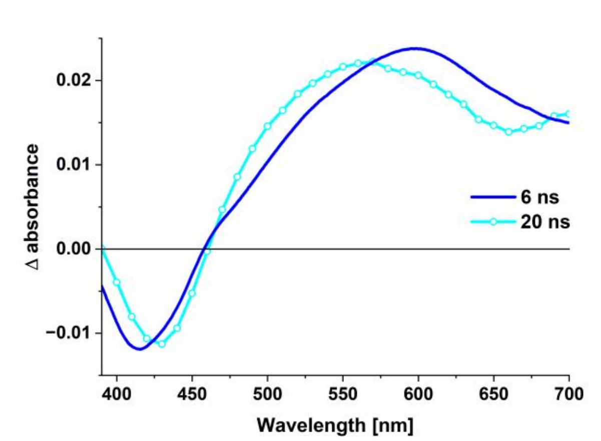	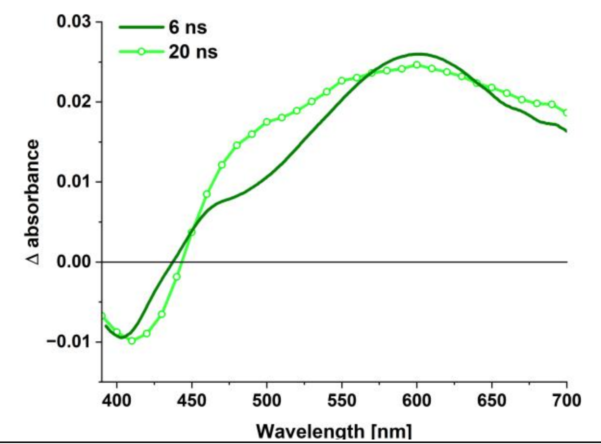

5

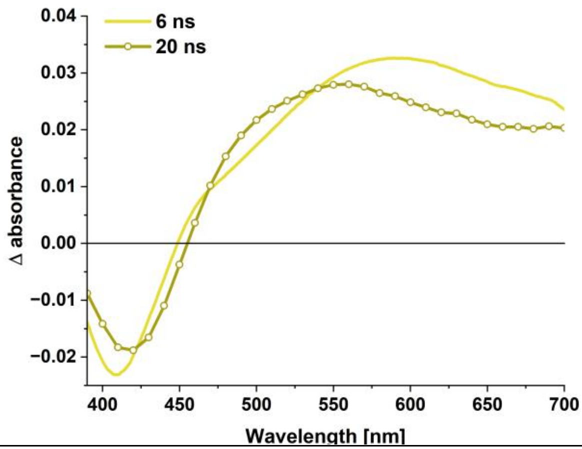

Figure S29. Comparison of the TA spectral profiles of the complexes **3–5** recorded at 6 ns delay time in the fs-TA experiments with their earliest delay-time spectra obtained by laser flash photolysis.

1

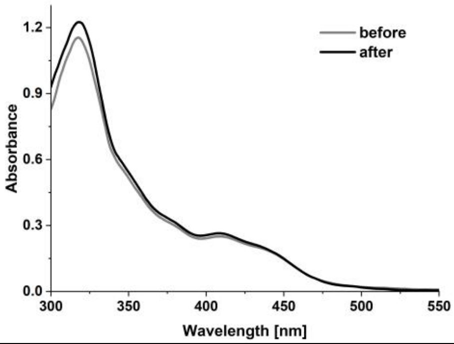

2

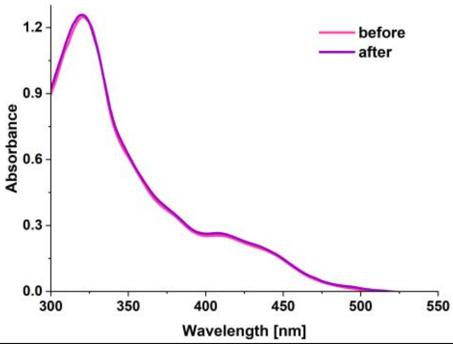

3

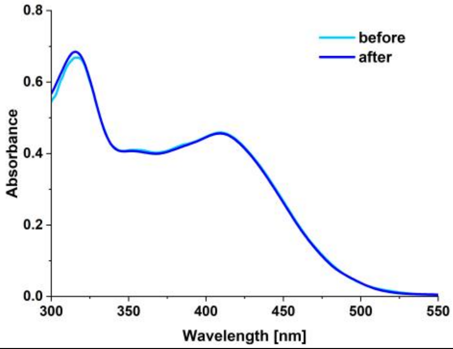

4

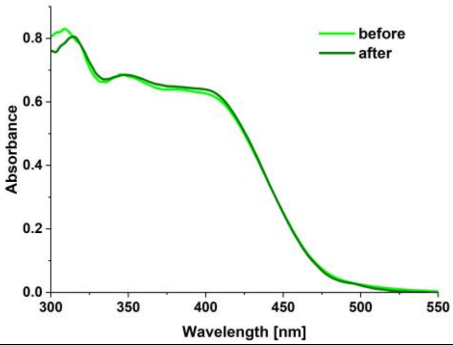

5

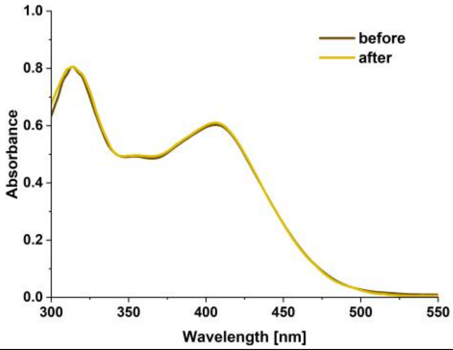

6

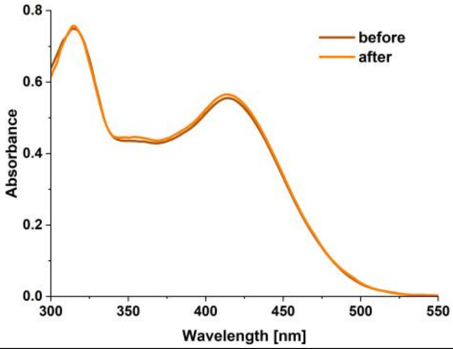

7
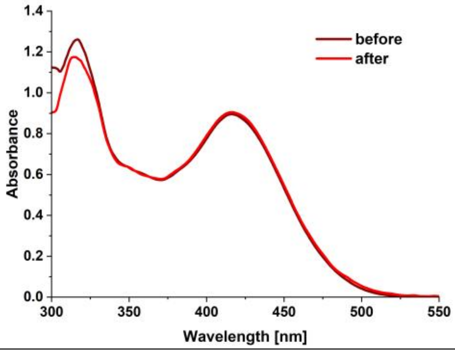

Figure S30. UV–Vis absorption spectra of complexes **1**–**7** recorded before and after laser irradiation.

Table S9. Triplet state maximum absorption (λ_{max}) and average lifetimes deaerated ($\boldsymbol{\tau_{T,N_2}}$) environments obtained for all tested compounds dissolved in acetonitrile

Compound	λ_{max} [nm]	$\tau_T \pm \Delta\tau_T$ [μs]
**1**	490	0.44 ± 0.01
**2**	380	0.36 ± 0.01
**3**	560	0.58 ± 0.02
**4**	600	0.88 ± 0.03
**5**	560	2.02 ± 0.05
**6**	530	0.64 ± 0.02
**7**	730	0.24 ± 0.01

Theoretical calculations

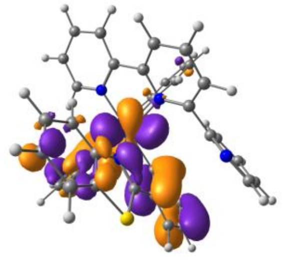	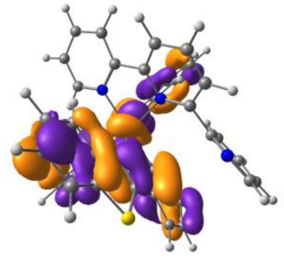	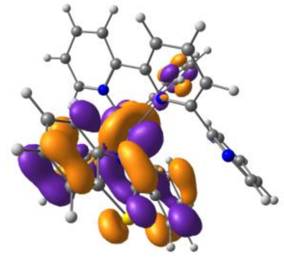	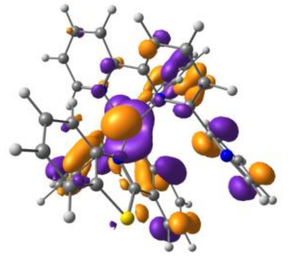
**HOMO**	**H–1**	**H–2**	**H–3**
	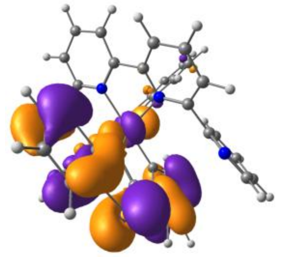	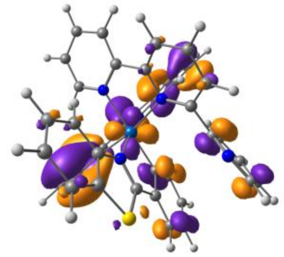	
	**H–4**	**H–5**	

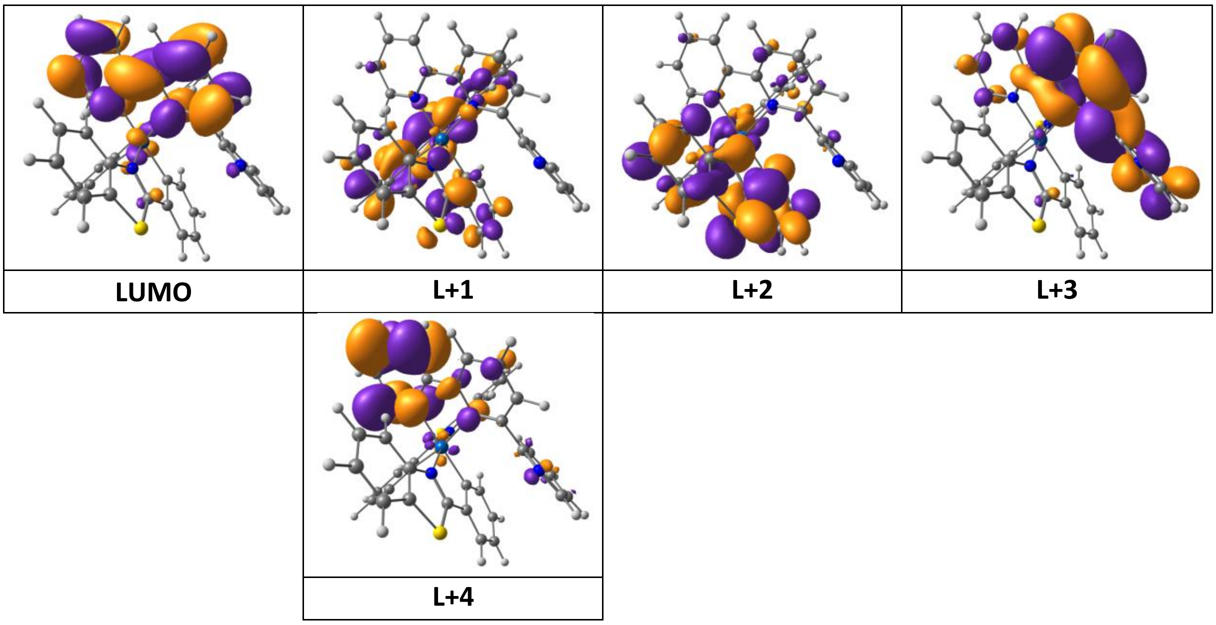
LUMO
L+1
L+2
L+3
L+4

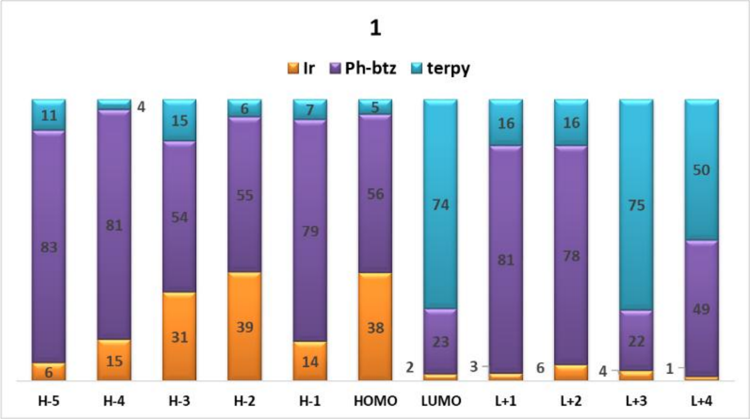
1
Ir
Ph-btz
terpy
H-5 6 83 11
H-4 15 81 4
H-3 31 54 15
H-2 39 55 6
H-1 14 79 7
HOMO 38 56 5
LUMO 2 23 74
L+1 3 81 16
L+2 6 78 16
L+3 4 22 75
L+4 1 49 50

1 (a)

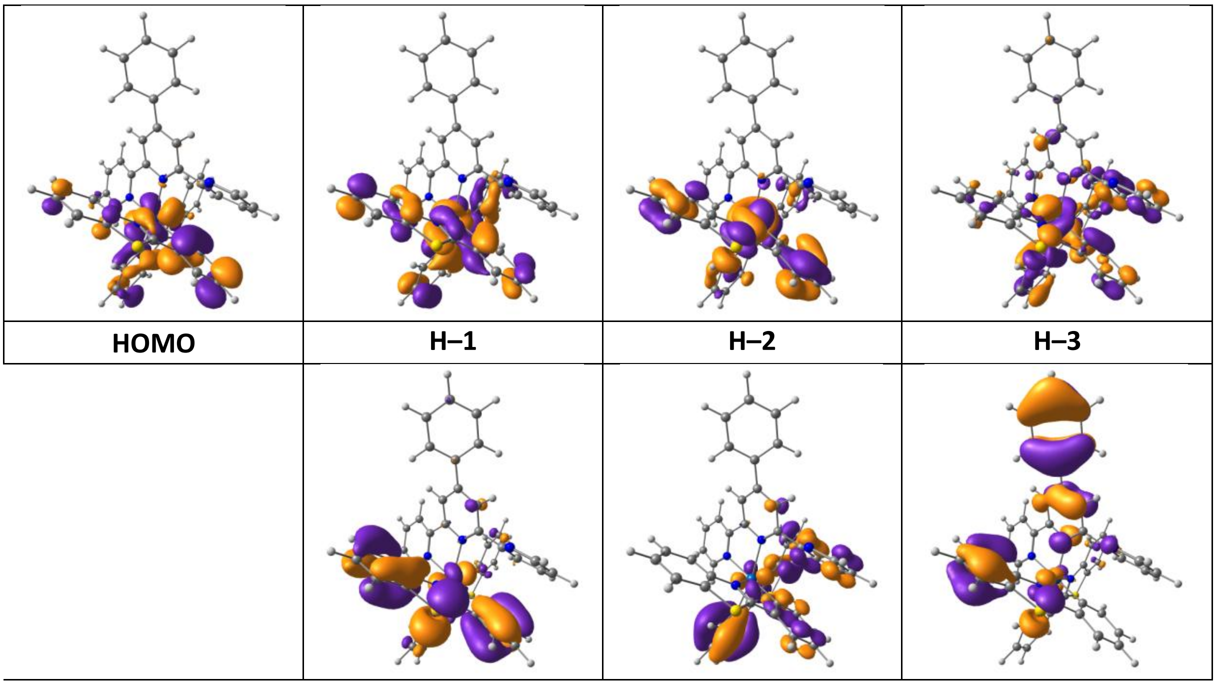
HOMO
H–1
H–2
H–3

	H–4	H–5	H–6
	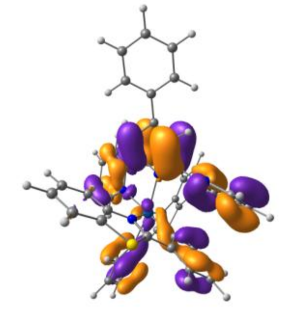		
	H–7		
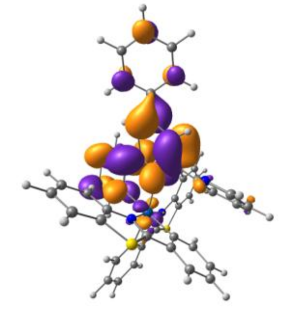	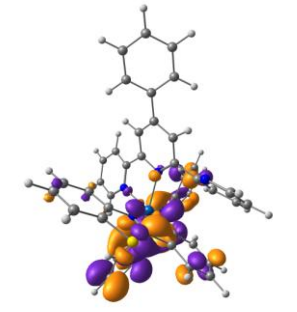	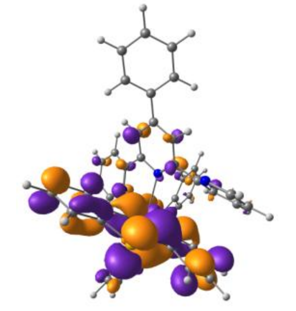	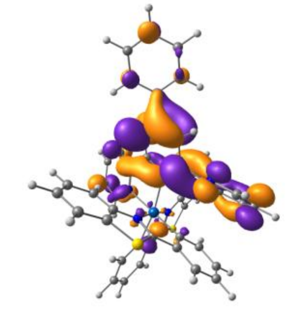
LUMO	L+1	L+2	L+3
	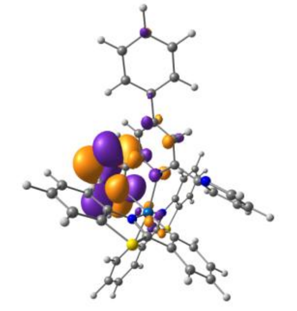		
	L+4		

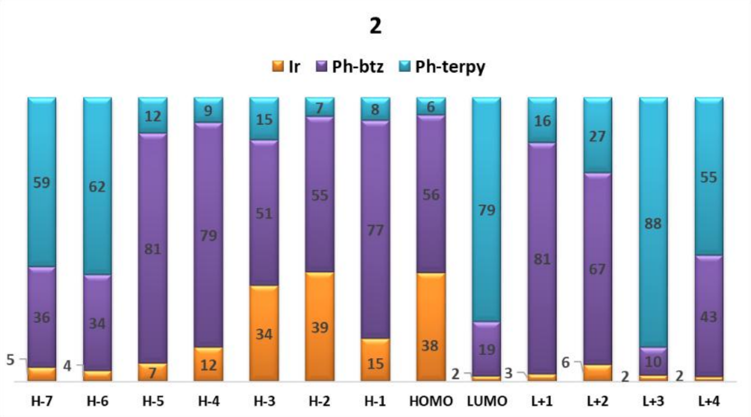

2 (b)

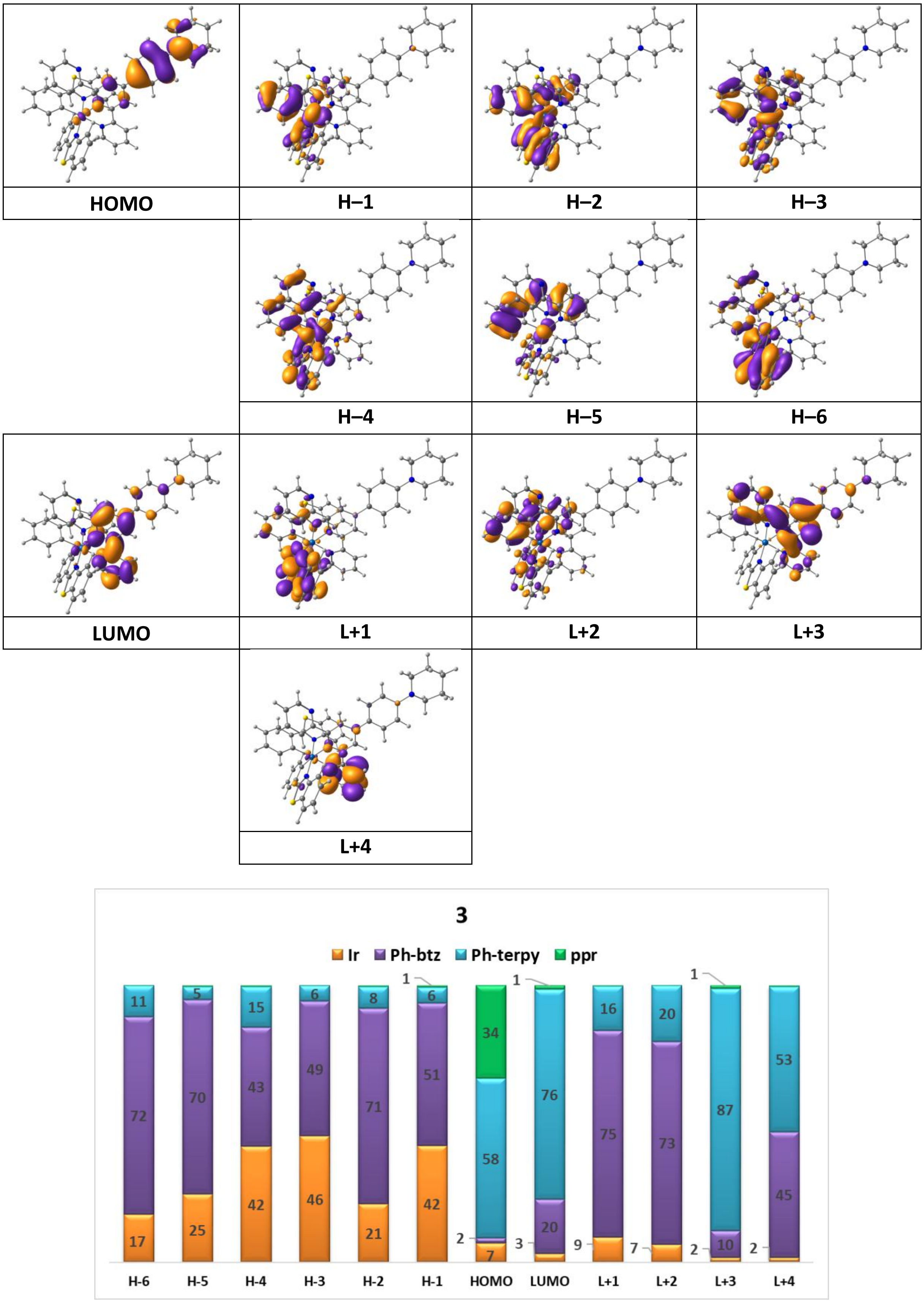
HOMO
H–1
H–2
H–3
H–4
H–5
H–6
LUMO
L+1
L+2
L+3
L+4
3
Ir
Ph-btz
Ph-terpy
ppr
H-6: 17, 72, 11
H-5: 25, 70, 5
H-4: 42, 43, 15
H-3: 46, 49, 6
H-2: 21, 71, 8
H-1: 42, 51, 6, 1
HOMO: 7, 2, 58, 34
LUMO: 3, 20, 76, 1
L+1: 9, 75, 16
L+2: 7, 73, 20
L+3: 2, 10, 87, 1
L+4: 2, 45, 53
H-6
H-5
H-4
H-3
H-2
H-1
HOMO
LUMO
L+1
L+2
L+3
L+4

3 (c)

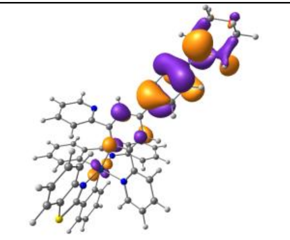	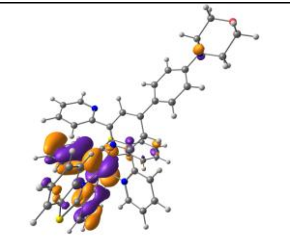	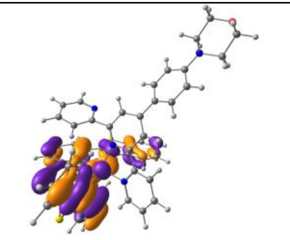	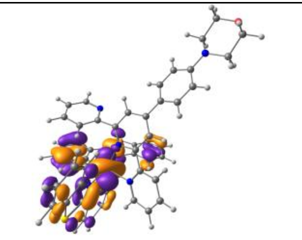
**HOMO**	**H–1**	**H–2**	**H–3**
	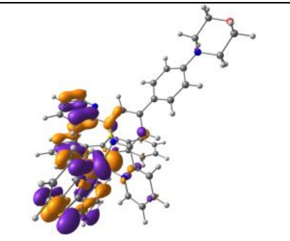	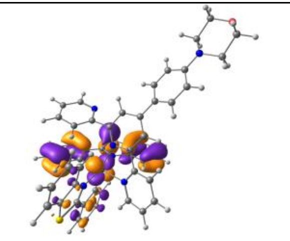	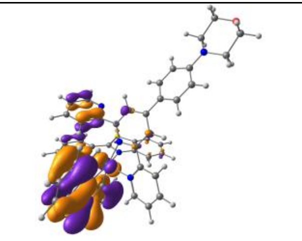
	**H–4**	**H–5**	**H–6**
	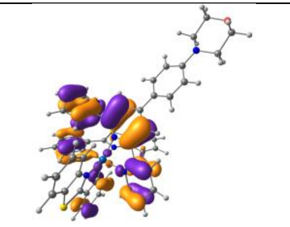		
	**H–7**		
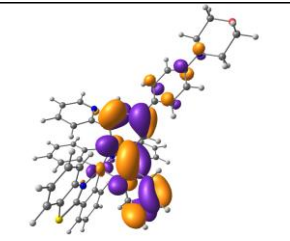	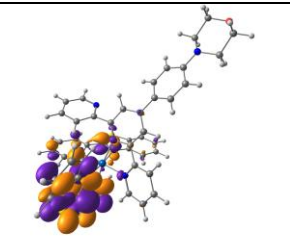	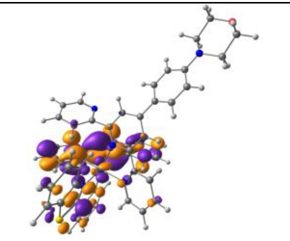	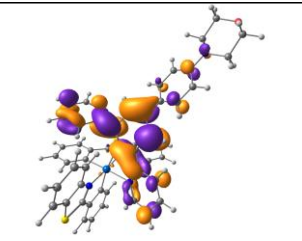
**LUMO**	**L+1**	**L+2**	**L+3**
	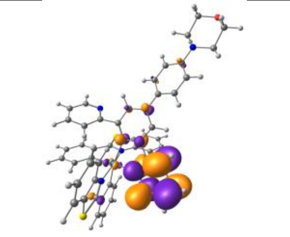		
	**L+4**		

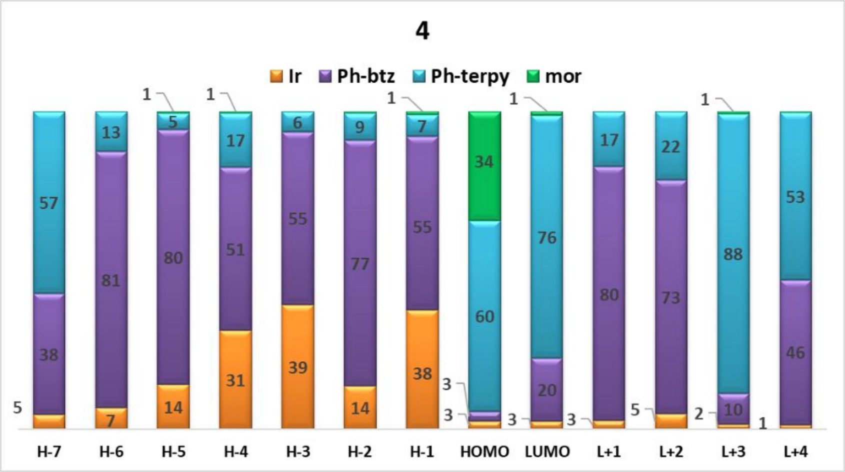
4
Ir
Ph-btz
Ph-terpy
mor
H-7
H-6
H-5
H-4
H-3
H-2
H-1
HOMO
LUMO
L+1
L+2
L+3
L+4

4 (d)

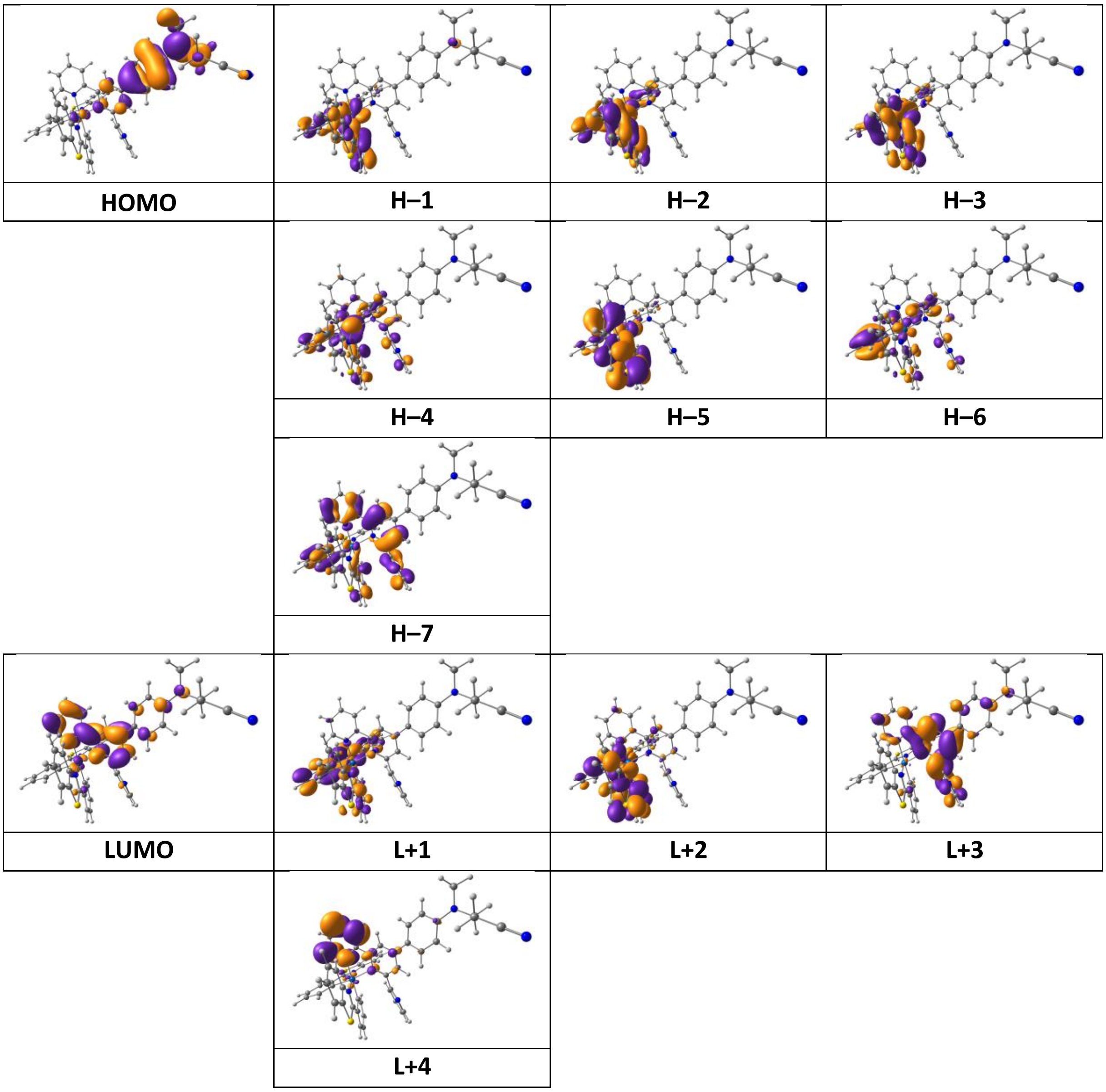
HOMO
H–1
H–2
H–3
H–4
H–5
H–6
H–7
LUMO
L+1
L+2
L+3
L+4

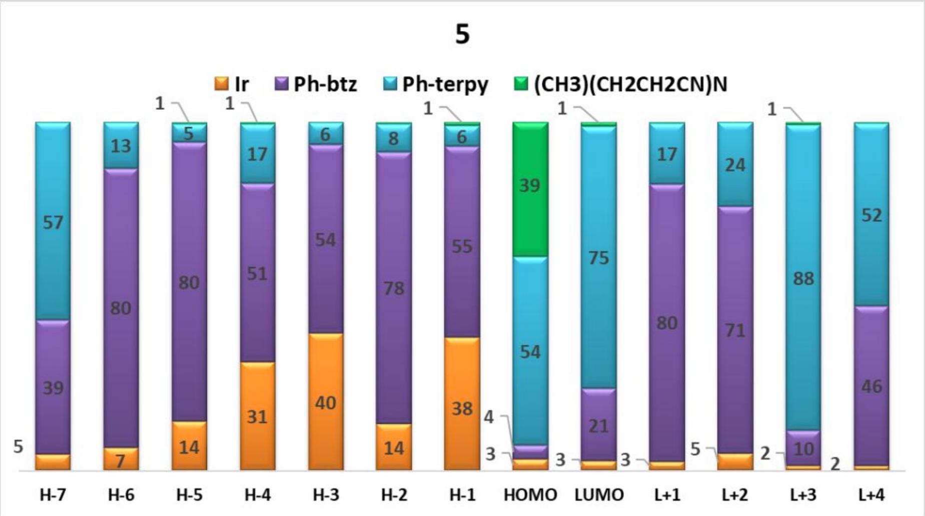
5
Ir
Ph-btz
Ph-terpy
(CH3)(CH2CH2CN)N
H-7
H-6
H-5
H-4
H-3
H-2
H-1
HOMO
LUMO
L+1
L+2
L+3
L+4

5 (e)

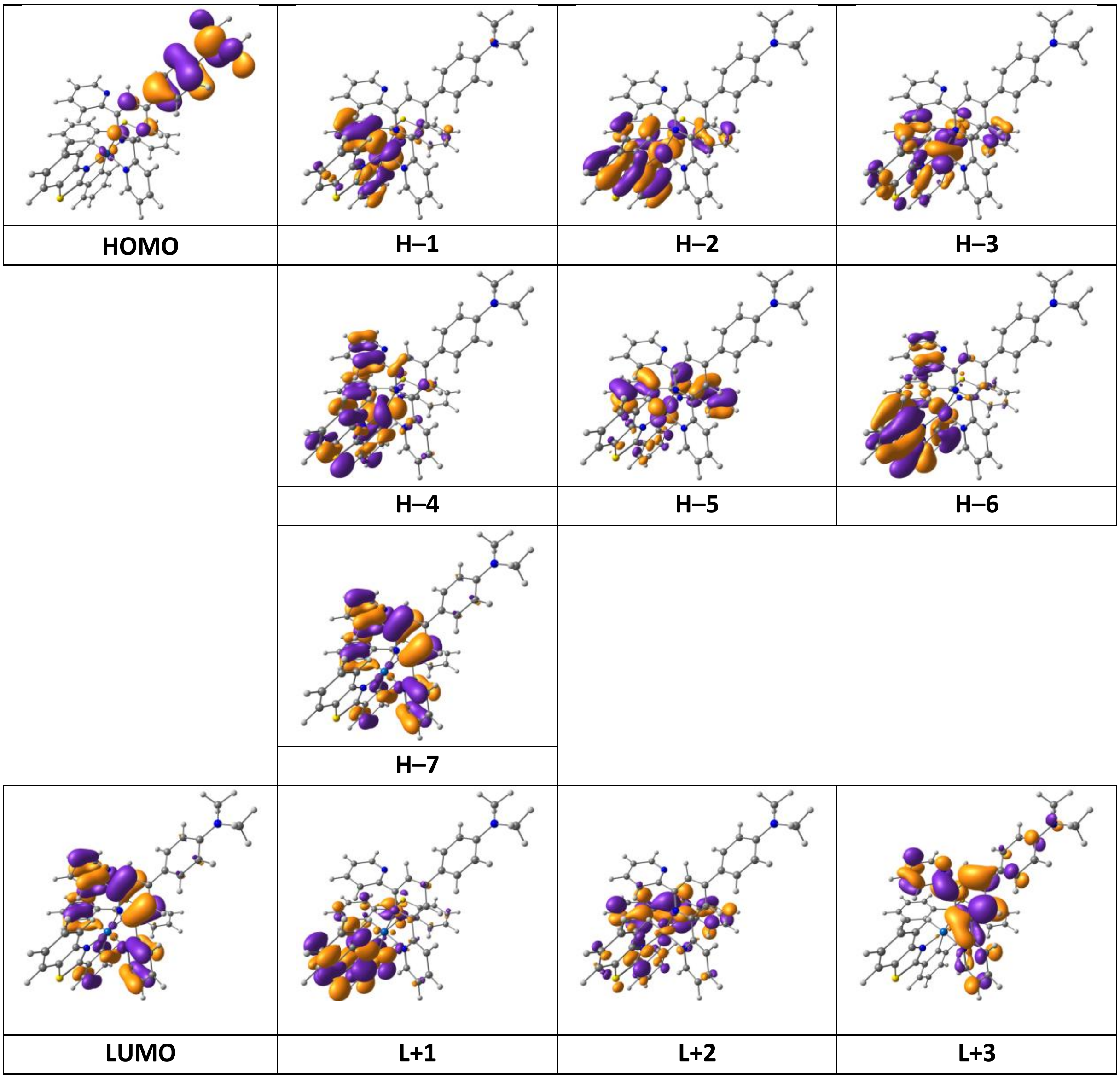
HOMO
H–1
H–2
H–3
H–4
H–5
H–6
H–7
LUMO
L+1
L+2
L+3

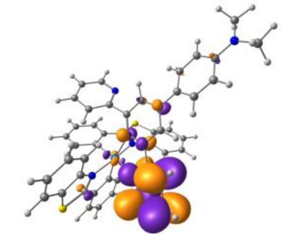	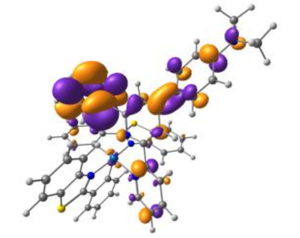
**L+4**	**L+5**

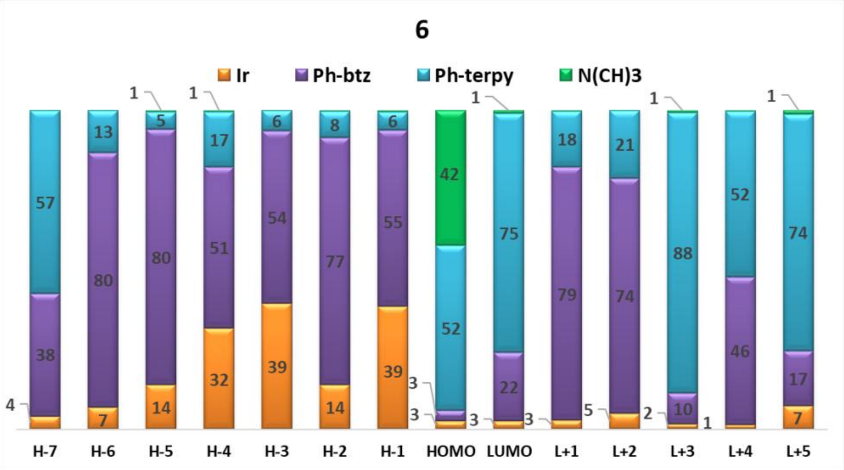

6 (f)

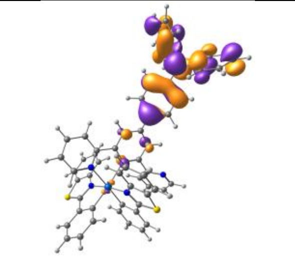	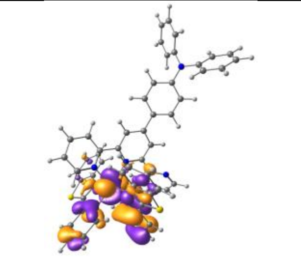	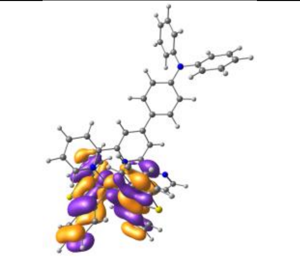	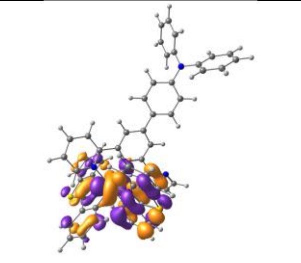
**HOMO**	**H–1**	**H–2**	**H–3**
	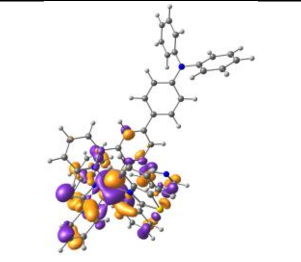	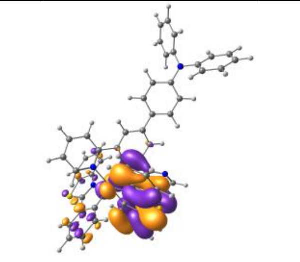	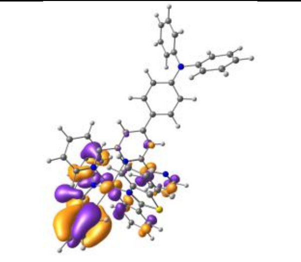
	**H–4**	**H–5**	**H–6**
	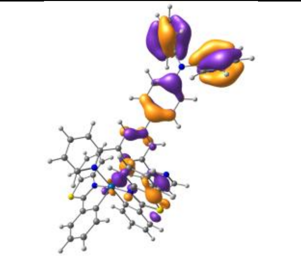	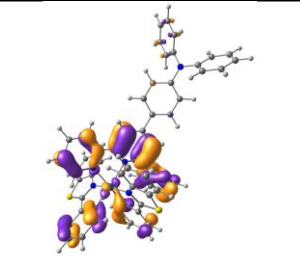	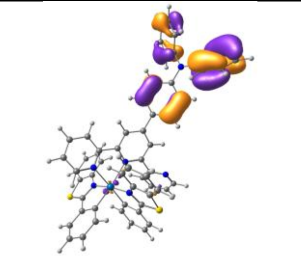
	**H–7**	**H–8**	**H–9**

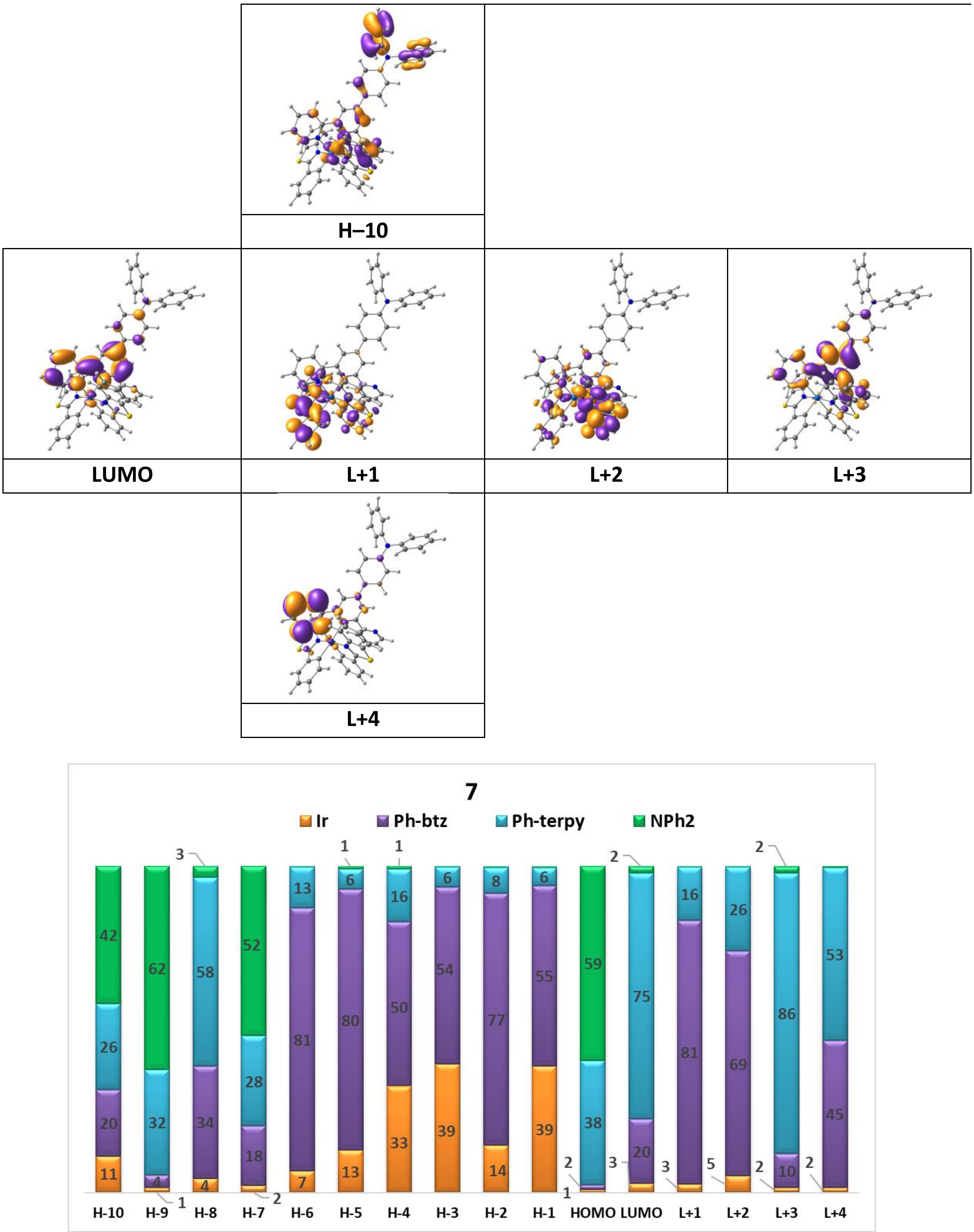

7 (g)

Figure S31. Frontier molecular orbitals of and major electron density distributions of complexes **1–7** (a–g, respectively).

Table S10. Assignment of calculated singlet excited states to the UV–Vis spectra and TD-DFT parameters of electronic transitions for **1** in acetonitrile (PCM); Gaussian 16, PBE0; Stuttgart/Dresden small-core ECP (Ir) and def2-TZVP (all other atoms).

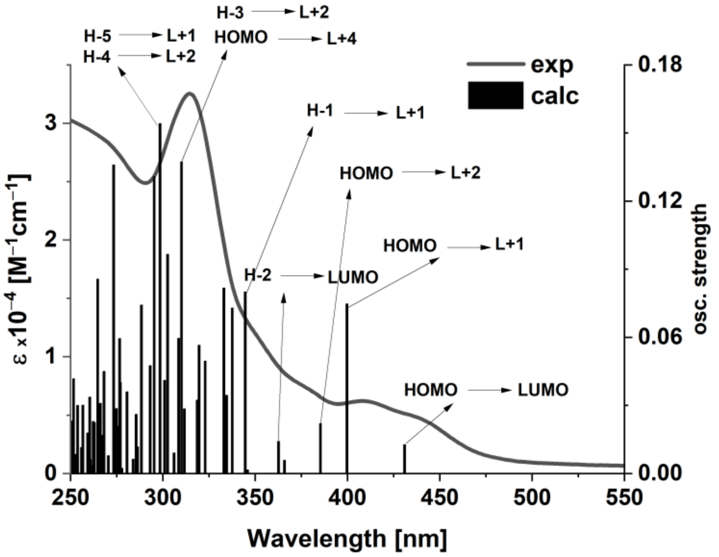

Exp. absorption λ; nm (10^{-4} ε; $M^{-1}cm^{-1}$)	Calculated transitions				
	Major contribution (%)	E [eV]	λ [nm]	Oscillator strength	transition No.
440.6 (0.63)	HOMO→LUMO (94 %)	2.88	431.0	0.0127	1
411.8 (0.81)	HOMO→L+1 (93 %)	3.10	399.8	0.0747	2
377.4 (1.04)	HOMO→L+2 (93 %)	3.22	385.3	0.0220	3
	H–1→LUMO (81 %)	3.39	365.9	0.0058	4
	H–2→LUMO (83 %)	3.42	362.7	0.0141	5
353.2 (1.73)	H–1→L+1 (82 %)	3.60	344.6	0.0800	7
	H–2→L+1 (66 %)<br>H–1→L+2 (15 %)	3.67	337.6	0.0729	8
	H–3→L+1 (62 %)	3.71	334.5	0.0345	9
	H–1→L+2 (67 %)<br>H–2→L+1 (21 %)	3.72	333.1	0.0817	10
316.0 (4.86)	H–2→L+2 (74 %)	3.84	322.9	0.0496	11
	H–4→LUMO (79 %)	3.88	319.6	0.0565	12
	H–3→L+1 (80 %)	3.89	318.6	0.0324	13
	HOMO→L+4 (79 %)	3.97	311.6	0.0285	14
	H–3→L+2 (65 %)<br>HOMO→L+4 (13 %)	4.00	310.1	0.1373	15
	H–5→LUMO (69 %)<br>H–1→L+3 (13 %)	4.02	308.5	0.0596	16
	H–1→L+3 (69 %)<br>H–5→LUMO (11 %)	4.05	306.1	0.0091	17
	H–4→L+1 (60 %)<br>H–4→L+2 (10 %)	4.10	302.5	0.0966	18
	H–2→L+3 (77 %)	4.12	300.9	0.0411	19
	H–5→L+1 (41 %)<br>H–4→L+2 (36 %)	4.15	298.5	0.1542	20

Table S11. Assignment of calculated singlet excited states to the UV–Vis spectra and TD-DFT parameters of electronic transitions for **2** in acetonitrile (PCM); Gaussian 16, PBE0; Stuttgart/Dresden small-core ECP (Ir) and def2-TZVP (all other atoms).

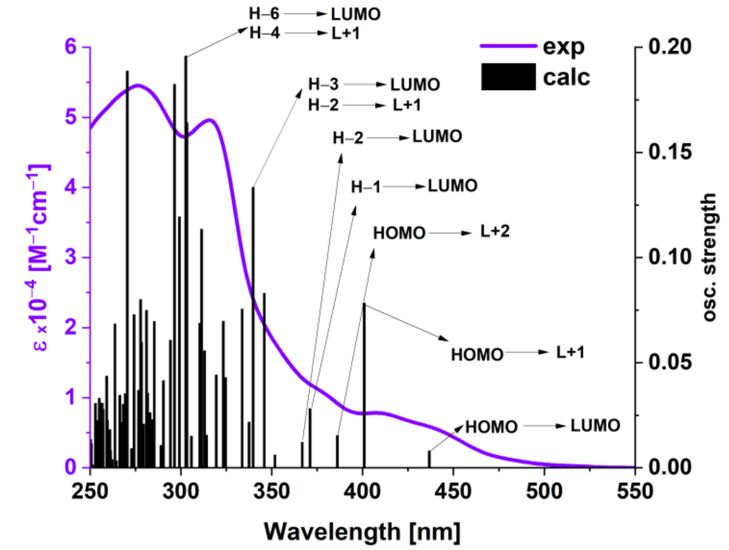

**Exp. absorption λ; nm (10^{-4} ε; $M^{-1}cm^{-1}$)**	**Calculated transitions**				
	**Major contribution (%)**	**E [eV]**	**λ [nm]**	**Oscillator strength**	**transition No.**
440.6 (0.47)	HOMO→LUMO (95 %)	2.84	436.8	0.0081	1
412.7 (0.64)	HOMO→L+1 (93 %)	3.09	400.9	0.0783	2
374.0 (0.76)	HOMO→L+2 (90 %)	3.21	386.1	0.0154	3
	H–1→LUMO (79 %)	3.34	371.1	0.0282	4
	H–2→LUMO (83 %)	3.38	366.8	0.0122	5
348.3 (1.73)	HOMO→L+3 (83 %)<br>H–3→LUMO (11 %)	3.52	351.8	0.0062	6
	H–1→L+1 (84 %)	3.58	345.9	0.0831	7
	H–3→LUMO (62 %)<br>H–2→L+1 (10 %)	3.65	339.7	0.1334	8
	H–2→L+1 (55 %)<br>H–1→L+2 (15 %)<br>H–3→LUMO (10 %)	3.67	337.5	0.0217	9
	H–1→L+2 (63 %)<br>H–2→L+1 (24 %)	3.71	333.8	0.0755	10
314.6 (3.25)	H–4→LUMO (86 %)	3.82	324.6	0.0429	11
	H–2→L+2 (73 %)	3.83	323.4	0.0697	12
	H–3→L+1 (81 %)	3.88	319.5	0.0442	13
	H–1→L+3 (12 %)<br>HOMO→L+4 (53 %)<br>H–5→LUMO (19 %)<br>H–1→L+3 (12 %)	3.95	314.2	0.0155	14
	H–1→L+3 (36 %)<br>HOMO→L+4 (31 %)<br>H–5→LUMO (18 %)	3.96	313.1	0.0557	15
	H–3→L+2 (31 %)<br>H–5→LUMO (30 %)<br>H–1→L+3 (23 %)	3.98	311.4	0.1134	16
	H–3→L+2 (49 %)<br>H–5→LUMO (18 %)<br>H–1→L+3 (12 %)	3.99	310.5	0.0688	17
	H–2→L+3 (79 %)	4.05	305.8	0.0151	18
	H–4→L+1 (48 %)<br>H–6→LUMO (15 %)<br>H–7→LUMO (14 %)	4.09	303.3	0.1641	19
	H–6→LUMO (55 %)<br>H–4→L+1 (19 %)	4.10	302.7	0.1958	20

	H–5→L+1 (35 %)<br>H–4→L+2 (23 %)<br>H–7→LUMO (13 %)<br>H–4→L+1 (11 %)	4.14	299.2	0.1194	21
269.4 (2.85)	H–7→LUMO (39 %)<br>H–4→L+2 (24 %)<br>H–6→LUMO (17 %)	4.18	296.5	0.1824	22

Table S12. Assignment of calculated singlet excited states to the UV–Vis spectra and TD-DFT parameters of electronic transitions for **3** in acetonitrile (PCM); Gaussian 16, PBE0; Stuttgart/Dresden small-core ECP (Ir) and def2-TZVP (all other atoms).

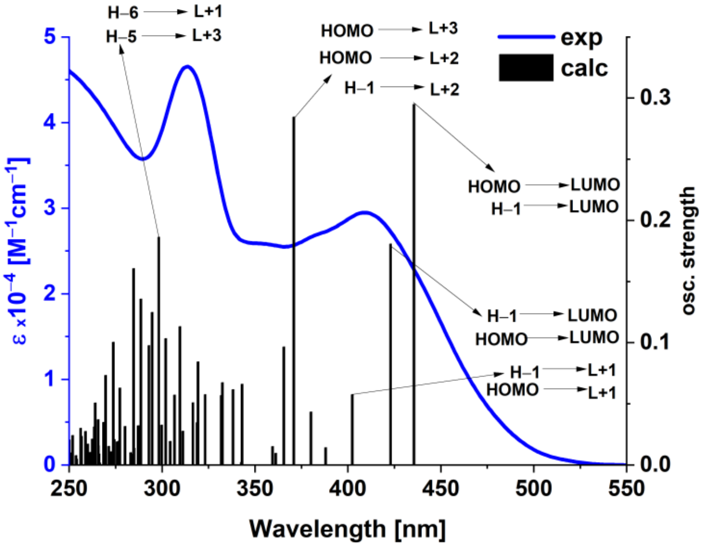

7

Exp. absorption λ; nm (10^{-4} ε; $M^{-1}cm^{-1}$)	Calculated transitions				
	Major contribution (%)	E [eV]	λ [nm]	Oscillator strength	transition No.
409.0 (2.94)	HOMO→LUMO (75 %)<br>H–1→LUMO (21%)	2.85	435.6	0.2950	1
	H–1→LUMO (68 %)<br>HOMO→LUMO (22 %)	2.93	423.0	0.1807	2
	H–1→L+1 (78 %)<br>HOMO→L+1 (10 %)	3.08	402.4	0.0576	3
382.3 (2.68)	H–1→L+2 (76 %)<br>HOMO→L+2 (18 %)	3.20	388.1	0.0141	4
	HOMO→L+1 (81 %)<br>H–1→L+1 (10 %)	3.26	380.2	0.0433	5
354.2 (2.58)	HOMO→L+3 (50 %)<br>HOMO→L+2 (33 %)<br>H–1→L+2 (11 %)	3.34	370.9	0.2845	6
	HOMO→L+2 (40 %)<br>HOMO→L+3 (41 %)	3.39	365.6	0.0965	7
	H–2→LUMO (76 %)	3.43	361.2	0.0095	8
	H–3→LUMO (85 %)	3.45	359.5	0.0151	9
	H–2→L+1 (84 %)	3.61	343.1	0.0660	10
	H–1→L+3 (86 %)	3.62	342.7	0.0021	11
	H–3→L+1(83 %)	3.67	338.2	0.0617	12
	H–4→LUMO (37 %)<br>H–2→L+2 (28 %)<br>H–3→L+2 (12 %)	3.73	332.5	0.0673	13
	H–2→L+2 (48 %)<br>H–4→LUMO (29 %)	3.74	331.9	0.0568	14
313.4 (4.65)	H–3→L+2 (66 %)	3.84	323.1	0.0575	15
	HOMO→L+4 (63 %)		319.4	0.0844	16

	H–4→L+2 (62 %)<br>H–1→L+4 (18 %)	3.88	309.6	0.1132	20
	H–6→L+1 (43 %)<br>H–5→L+3 (12 %)	4.16	298.3	0.1863	25

Table S13. Assignment of calculated singlet excited states to the UV–Vis spectra and TD-DFT parameters of electronic transitions for **4** in acetonitrile (PCM); Gaussian 16, PBE0; Stuttgart/Dresden small-core ECP (Ir) and def2-TZVP (all other atoms).

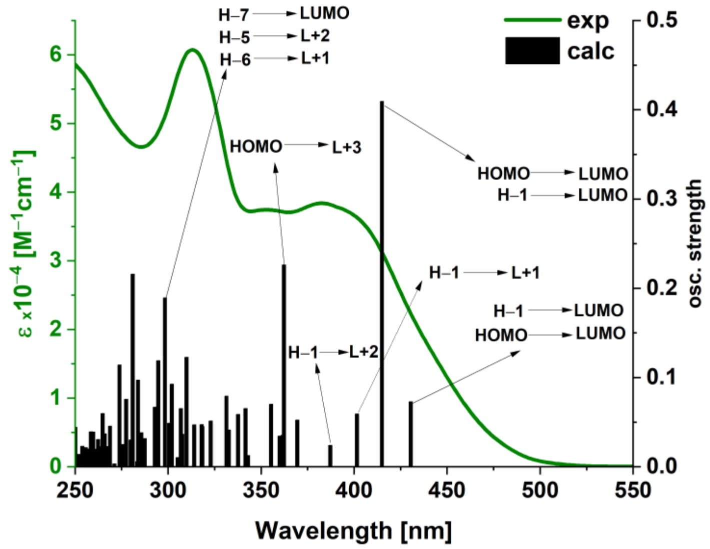

Exp. absorption λ; nm (10^{-4} ε; $M^{-1}cm^{-1}$)	Calculated transitions				
	**Major contribution (%)**	**E [eV]**	**λ [nm]**	**Oscillator strength**	**transition No.**
382.8 (3.85)	H–1→LUMO (65%)<br>HOMO→LUMO (29 %)	2.88	430.5	0.073	1
	HOMO→LUMO (67 %)<br>H–1→LUMO (24 %)	2.99	415.0	0.4094	2
	H–1→L+1 (80 %)	3.09	401.6	0.0593	3
	H–1→L+2 (82%)	3.20	387.3	0.0241	4
352.6 (3.75)	HOMO→L+1 (76 %)	3.36	369.5	0.0524	5
	HOMO→L+3 (41 %)<br>HOMO→L+2 (33 %)	3.42	362.3	0.2264	6
	H–2→LUMO (75 %)<br>HOMO→L+3 (11%)	3.43	361.7	0.0356	7
	H–3→LUMO (77%)	3.44	360.0	0.0345	8
	HOMO→L+2 (45 %)<br>HOMO→L+3 (32%)	3.49	355.4	0.0702	9
312.8 (6.06)	H–1→L+3 (85 %)	3.61	343.1	0.0128	10
	H–2→L+1 (82 %)	3.63	341.6	0.0653	11
	H–3→L+1 (84 %)	3.67	337.6	0.0588	12
	H–4→LUMO (60 %)<br>H–3→L+2 (11 %)	3.73	332.7	0.0414	13
	H–2→L+2 (70 %)	3.74	331.4	0.0792	14
	H–4→L+2 (76 %)	4.00	309.9	0.1229	19
	H–7→LUMO (34 %)<br>H–5→L+2 (31 %)<br>H–6→L+1 (10 %)	4.16	298.3	0.1894	25

Table S14. Assignment of calculated singlet excited states to the UV–Vis spectra and TD-DFT parameters of electronic transitions for **5** in acetonitrile (PCM); Gaussian 16, PBE0; Stuttgart/Dresden small-core ECP (Ir) and def2-TZVP (all other atoms).

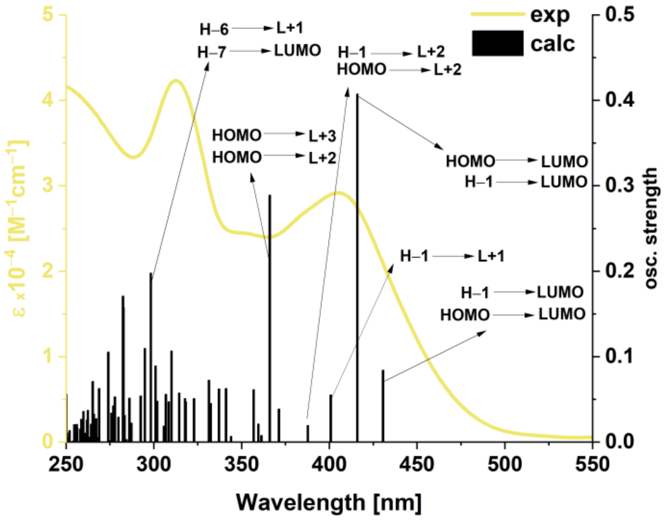

Exp. absorption λ; nm (10^{-4} ε; $M^{-1}cm^{-1}$)	Calculated transitions				
	Major contribution (%)	E [eV]	λ [nm]	Oscillator strength	transition No.
406.36 (2.91)	H–1→LUMO (61%)<br>HOMO→LUMO (32 %)	2.88	430.6	0.0842	1
	HOMO→LUMO (63 %)<br>H–1→LUMO (27 %)	2.98	416.0	0.4077	2
	H–1→L+1 (79 %)	3.09	400.9	0.0522	3
	H–1→L+2 (80 %)<br>HOMO→L+2 (14 %)	3.20	387.8	0.0195	4
349.1 (2.46)	HOMO→L+1 (77 %)	3.34	371.3	0.0387	5
	HOMO→L+3 (52 %)<br>HOMO→L+2 (33 %)	3.39	366.1	0.2890	6
	H–2→LUMO (73 %)	3.43	361.3	0.0077	7
	H–3→LUMO (76 %)<br>H–2→LUMO (10 %)	3.45	359.6	0.0212	8
	HOMO→L+2 (39 %)<br>HOMO→L+3 (31 %)	3.47	356.9	0.0614	9
	H–1→L+3 (87 %)	3.60	344.0	0.0066	10
	H–2→L+1 (83 %)	3.63	341.1	0.0626	11
	H–3→L+1 (83 %)	3.68	337.2	0.0622	12
	H–4→LUMO (57 %)<br>H–3→L+2 (13 %)	3.73	332.5	0.0452	13
	H–2→L+2 (67 %)<br>H–4→LUMO (10 %)	3.74	331.5	0.0724	14
313.5 (4.23)	H–4→L+2 (69 %)	4.00	310.1	0.1066	19
	H–6→L+1 (40 %)<br>H–7→LUMO (10 %)	4.16	298.3	0.1980	25

Table S15. Assignment of calculated singlet excited states to the UV–Vis spectra and TD-DFT parameters of electronic transitions for **6** in acetonitrile (PCM); Gaussian 16, PBE0; Stuttgart/Dresden small-core ECP (Ir) and def2-TZVP (all other atoms).

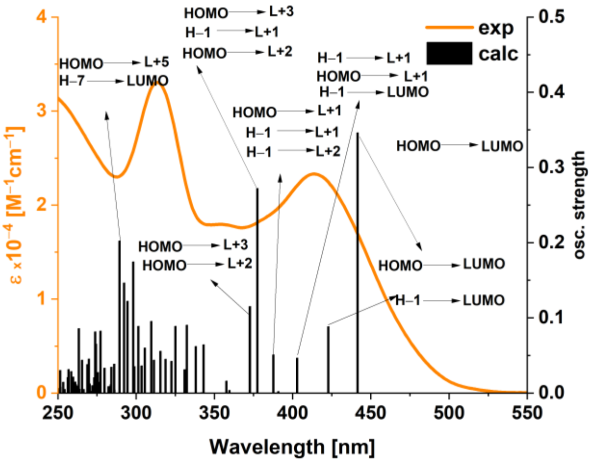

Exp. absorption λ; nm (10^{-4} ε; $M^{-1}cm^{-1}$)	Calculated transitions				
	Major contribution (%)	E [eV]	λ [nm]	Oscillator strength	transition No.
414.4 (2.33)	HOMO→LUMO (90 %)	2.80	441.5	0.3462	1
	H–1→LUMO (80 %)	2.93	422.8	0.0885	2
	H–1→L+1 (65 %)<br>HOMO→L+1 (22 %)<br>H–1→LUMO (10 %)	3.08	402.9	0.0466	3
	H–1→L+2 (46 %)<br>HOMO→L+2 (34 %)<br>HOMO→L+1 (10 %)	3.17	390.9	0.0023	4
	HOMO→L+1 (61 %)<br>H–1→L+1 (18 %)<br>H–1→L+2 (14 %)	3.20	387.7	0.0509	5
	HOMO→L+3 (43 %)<br>H–1→L+2 (27 %)<br>HOMO→L+2 (25 %)	3.28	377.5	0.2721	6
	HOMO→L+3 (50 %)<br>HOMO→L+2 (36 %)	3.33	372.7	0.1152	7
349.1 (2.46)	H–2→LUMO (80 %)	3.45	359.7	0.0038	8
	H–3→LUMO (84 %)	3.47	357.7	0.0160	9
	H–2→L+1 (83 %)	3.61	343.1	0.0643	10
	H–1→L+3 (87 %)	3.63	341.7	0.0007	11
	H–3→L+1 (82 %)	3.67	338.1	0.0621	12
313.5 (4.23)	H–2→L+2 (70 %)	3.73	332.4	0.0905	13
	H–4→LUMO (60 %)	3.75	331.0	0.0313	14
	HOMO→L+4 (79 %)<br>H–3→L+2 (11 %)	3.81	325.1	0.0889	15
	H–4→L+2 (59 %)<br>H–1→L+4 (21 %)	4.00	309.7	0.0956	20
	H–6→L+1 (39 %)<br>H–5→L+2 (32 %)	4.16	298.1	0.1745	25
	HOMO→L+5 (74 %)<br>H–7→LUMO (14 %)	4.28	289.4	0.2023	28

Table S16. Assignment of calculated singlet excited states to the UV–Vis spectra and TD-DFT parameters of electronic transitions for **7** in acetonitrile (PCM); Gaussian 16, PBE0; Stuttgart/Dresden small-core ECP (Ir) and def2-TZVP (all other atoms).

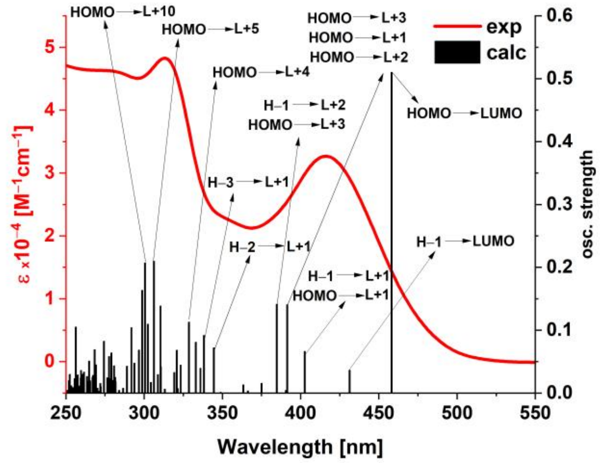

Exp. absorption λ; nm (10^{-4} ε; $M^{-1}cm^{-1}$)	Calculated transitions				
	Major contribution (%)	E [eV]	λ [nm]	Oscillator strength	transition No.
	HOMO→LUMO (96 %)	2.71	458.2	0.5103	1
	H–1→LUMO (91 %)	2.93	431.3	0.0364	2
	H–1→L+1 (75 %)<br>HOMO→L+1 (16 %)	3.08	402.6	0.0663	3
416.3 (3.25)	HOMO→L+3 (36 %)<br>HOMO→L+1 (21 %)<br>HOMO→L+2 (21 %)<br>H–1→L+2 (13 %)	3.17	391.5	0.1408	4
	H–1→L+2 (53 %)<br>HOMO→L+3 (34 %)	3.22	384.8	0.1415	6
	HOMO→L+2 (57 %)<br>HOMO→L+3(25 %)<br>H–1→L+2 (11 %)	3.31	375.1	0.0156	7
	H–2→LUMO (79 %)	3.38	366.4	0.0035	8
	H–3→LUMO (82 %)	3.42	363.4	0.0132	9
	H–1→L+3 (86 %)	3.55	348.9	0.0013	10
	H–2→L+1 (84 %)	3.60	344.6	0.0722	11
	H–3→L+1 (69 %)	3.67	338.2	0.0918	12
	H–4→LUMO (63 %)	3.69	336.0	0.0395	13
	H–2→L+2 (73 %)<br>H–3→L+1 (13 %)	3.72	333.0	0.0811	14
353.5 (2.27)	HOMO→L+4 (85 %)	3.77	328.7	0.1131	15
	H–3→L+2 (73 %)	3.83	323.4	0.0447	16
	HOMO→L+6 (67 %)<br>H–5→LUMO (20 %)	3.86	321.5	0.0071	17
	H–5→LUMO (63 %)<br>HOMO→L+6 (20 %)	3.86	320.9	0.0684	18
	H–4→L+1 (81 %)	3.88	319.3	0.0328	19
	H–1→L+4 (77 %)	3.96	313.2	0.0062	20
	H–2→L+3 (39 %)<br>H–6→LUMO (37 %)	3.99	310.7	0.0420	21
	H–4→L+2 (73 %)	3.99	310.5	0.1388	22
313.9 (4.82)	H–2→L+3 (39 %)<br>H–6→LUMO (34 %)	4.01	308.9	0.0297	23
	HOMO→L+5 (68 %)	4.05	306.3	0.2098	24
	H–3→L+3 (77 %)	4.07	304.4	0.0171	25
	H–5→L+1 (66 %)	4.10	302.5	0.1096	26
	HOMO→L+10 (97 %)	4.12	300.6	0.2070	27
	H–6→L+1 (40 %)<br>H–5→L+2 (66 %)	4.15	298.8	0.1636	28

	H–8→LUMO (12 %)				
	H–8→LUMO (39 %)<br>H–5→L+2 (24 %)<br>H–7→LUMO (14 %)	4.18	296.7	0.0687	29
	H–6→L+1 (43 %)<br>H–5→L+2 (15 %)<br>H–8→LUMO (11 %)	4.22	293.8	0.0477	30
	H–7→LUMO (35 %)<br>H–10→LUMO (28 %)	4.25	291.9	0.1041	31
	H–4→L+3 (77 %)	4.29	289.0	0.0428	32

1	
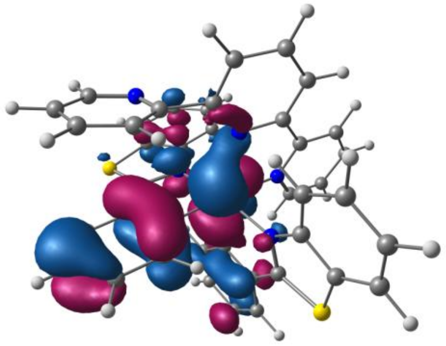	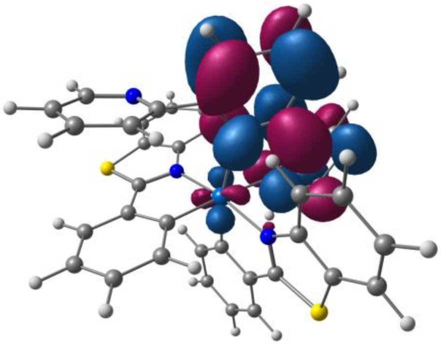
**LSOMO**	**HSOMO**
**2**	
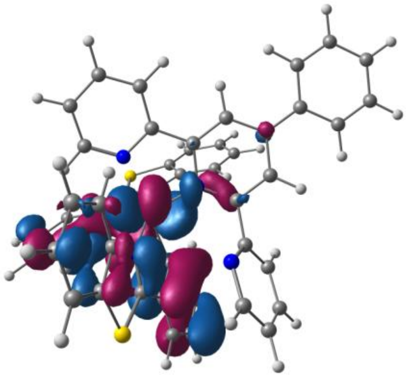	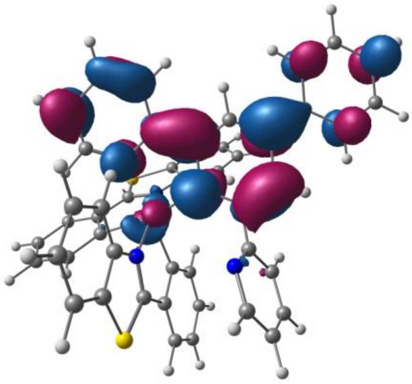
**LSOMO**	**HSOMO**
**4**	
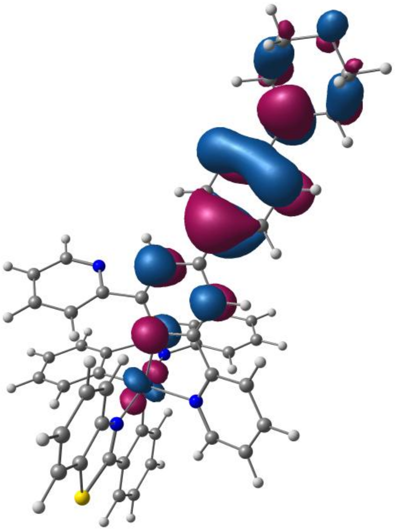	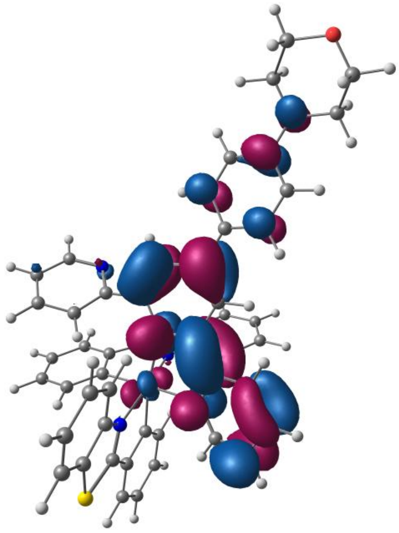
**LSOMO**	**HSOMO**

5	
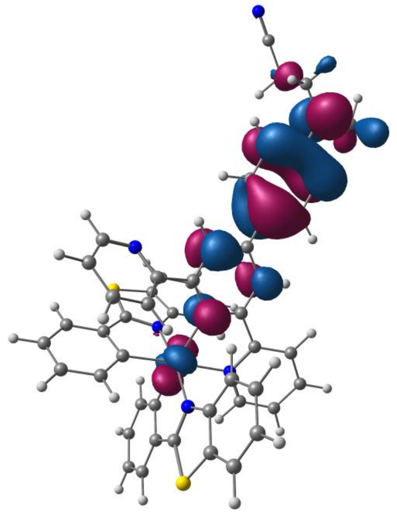	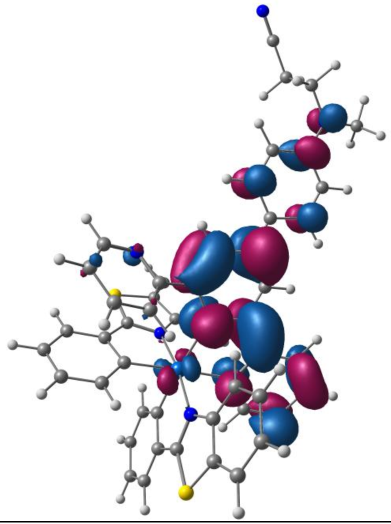
**LSOMO**	**HSOMO**
**6**	
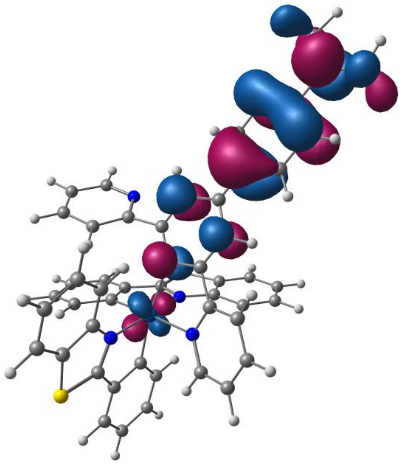	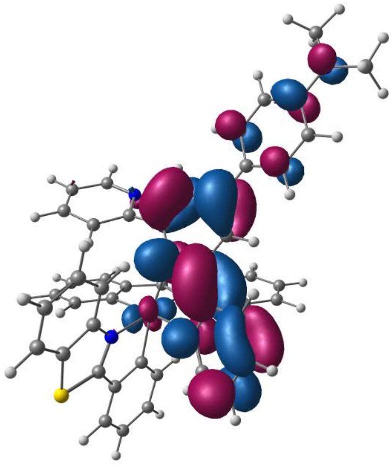
**LSOMO**	**HSOMO**
**7**	
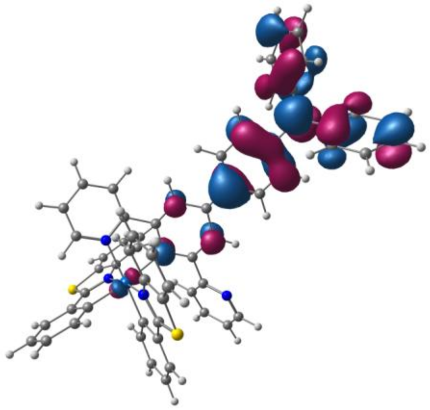	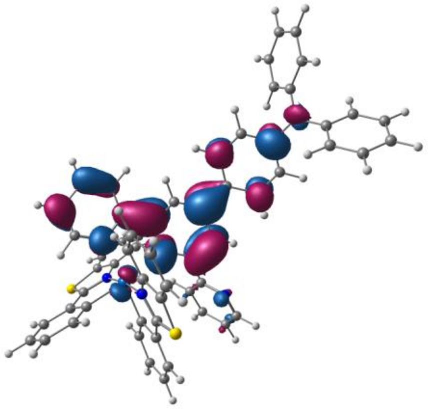
**LSOMO**	**HSOMO**

Figure S32. The isodensity surface plots of the LSOMO and HSOMO of **1**–**7**.

References

(1) Sheldrick, G. M. Crystal Structure Refinement with SHELXL. *Acta Cryst C* **2015**, *71* (1), 3–8. https://doi.org/10.1107/S2053229614024218.

(2) J. Frisch, G. W. Trucks, H. B. Schlegel, G. E. Scuseria, M. A. Robb, J. R. Cheeseman, G. Scalmani, V. Barone, G. A. Petersson, H. Nakatsuji, X. Li, M. Caricato, A. V. Marenich, J. Bloino, B. G. Janesko, R. Gomperts, B. Mennucci, H. P. Hratchian, J. V. Ortiz, A. F. Izmaylov, J. L. Sonnenberg, Williams, F. Ding, F. Lipparini, F. Egidi, J. Goings, B. Peng, A. Petrone, T. Henderson, D. Ranasinghe, V. G. Zakrzewski, J. Gao, N. Rega, G. Zheng, W. Liang, M. Hada, M. Ehara, K. Toyota, R. Fukuda, J. Hasegawa, M. Ishida, T. Nakajima, Y. Honda, O. Kitao, H. Nakai, T. Vreven, K. Throssell, J. A. Montgomery Jr., J. E. Peralta, F. Ogliaro, M. J. Bearpark, J. J. Heyd, E. N. Brothers, K. N. Kudin, V. N. Staroverov, T. A. Keith, R. Kobayashi, J. Normand, K. Raghavachari, A. P. Rendell, J. C. Burant, S. S. Iyengar, J. Tomasi, M. Cossi, J. M. Millam, M. Klene, C. Adamo, R. Cammi, J. W. Ochterski, R. L. Martin, K. Morokuma, O. Farkas, J. B. Foresman and D. J. Fox. Gaussian 16 Rev. C.01, 2016.

(3) Adamo, C.; Barone, V. Toward Reliable Density Functional Methods without Adjustable Parameters: The PBE0 Model. *The Journal of Chemical Physics* **1999**, *110* (13), 6158–6170. https://doi.org/10.1063/1.478522.

(4) Ernzerhof, M.; Scuseria, G. E. Assessment of the Perdew–Burke–Ernzerhof Exchange-Correlation Functional. *J. Chem. Phys.* **1999**, *110* (11), 5029–5036. https://doi.org/10.1063/1.478401.

(5) Andrae, D.; Häußermann, U.; Dolg, M.; Stoll, H.; Preuß, H. Energy-Adjustedab Initio Pseudopotentials for the Second and Third Row Transition Elements. *Theoret. Chim. Acta* **1990**, *77* (2), 123–141. https://doi.org/10.1007/BF01114537.

(6) Martin, J. M. L.; Sundermann, A. Correlation Consistent Valence Basis Sets for Use with the Stuttgart–Dresden–Bonn Relativistic Effective Core Potentials: The Atoms Ga–Kr and In–Xe. *J. Chem. Phys.* **2001**, *114* (8), 3408–3420. https://doi.org/10.1063/1.1337864.

(7) Weigend, F.; Ahlrichs, R. Balanced Basis Sets of Split Valence, Triple Zeta Valence and Quadruple Zeta Valence Quality for H to Rn: Design and Assessment of Accuracy. *Phys. Chem. Chem. Phys.* **2005**, *7* (18), 3297–3305. https://doi.org/10.1039/B508541A.

(8) Weigend, F. Accurate Coulomb-Fitting Basis Sets for H to Rn. *Phys. Chem. Chem. Phys.* **2006**, *8* (9), 1057–1065. https://doi.org/10.1039/B515623H.

(9) Rappoport, D.; Furche, F. Property-Optimized Gaussian Basis Sets for Molecular Response Calculations. *The Journal of Chemical Physics* **2010**, *133* (13), 134105. https://doi.org/10.1063/1.3484283.

(10) Cancès, E.; Mennucci, B.; Tomasi, J. A New Integral Equation Formalism for the Polarizable Continuum Model: Theoretical Background and Applications to Isotropic and Anisotropic Dielectrics. *The Journal of Chemical Physics* **1997**, *107* (8), 3032–3041. https://doi.org/10.1063/1.474659.

(11) Cossi, M.; Barone, V.; Mennucci, B.; Tomasi, J. Ab Initio Study of Ionic Solutions by a Polarizable Continuum Dielectric Model. *Chemical Physics Letters* **1998**, *286* (3), 253–260. https://doi.org/10.1016/S0009-2614(98)00106-7.

(12) Palion-Gazda, J.; Machura, B.; Szłapa-Kula, A.; Maroń, A. M.; Nycz, J. E.; Ledwon, P.; Schab-Balcerzak, E.; Siwy, M.; Grzelak, J.; Maćkowski, S. Effect of Carbazole and Pyrrolidine Functionalization of Phenanthroline Ligand on Ground- and Excited-State Properties of Rhenium(I) Complexes. Interplay between 3MLCT and 3IL/3ILCT. *Dyes and Pigments* **2022**, *200*, 110113. https://doi.org/10.1016/j.dyepig.2022.110113.

(13) Szlapa-Kula, A.; Małecka, M.; Maroń, A. M.; Janeczek, H.; Siwy, M.; Schab-Balcerzak, E.; Szalkowski, M.; Maćkowski, S.; Pedzinski, T.; Erfurt, K.; Machura, B. In-Depth Studies of Ground- and Excited-State Properties of Re(I) Carbonyl Complexes Bearing 2,2′:6′,2″-Terpyridine and 2,6-Bis(Pyrazin-2-Yl)Pyridine Coupled with π-Conjugated Aryl Chromophores. *Inorg. Chem.* **2021**, *60* (24), 18726–18738. https://doi.org/10.1021/acs.inorgchem.1c02151.

(14) Małecka, M.; Szlapa-Kula, A.; Maroń, A. M.; Ledwon, P.; Siwy, M.; Schab-Balcerzak, E.; Sulowska, K.; Maćkowski, S.; Erfurt, K.; Machura, B. Impact of the Anthryl Linking Mode on the Photophysics and Excited-State Dynamics of Re(I) Complexes [ReCl(CO)3(4′-An-Terpy-κ2N)]. *Inorg. Chem.* **2022**, *61* (38), 15070–15084. https://doi.org/10.1021/acs.inorgchem.2c02160.

(15) Slavov, C.; Hartmann, H.; Wachtveitl, J. Implementation and Evaluation of Data Analysis Strategies for Time-Resolved Optical Spectroscopy. *Anal. Chem.* **2015**, *87* (4), 2328–2336. https://doi.org/10.1021/ac504348h.

(16) Palion-Gazda, J.; Kwiecień, A.; Choroba, K.; Penkala, M.; Erfurt, K.; Machura, B. Effect of the Appended Morpholinyl Group on Photophysical Behavior of Mono- and Bis-Cyclometalated Terpyridine Iridium(III) Chromophores. *Inorg. Chem.* **2025**, *64* (1), 646–661. https://doi.org/10.1021/acs.inorgchem.4c03769.
